# Supplementary figures and images for: SPOP targets the immune transcription factor IRF1 for proteasomal degradation (part 1 of 2)
Source: eLife. 2023 Aug 25;12:e89951. doi: 10.7554/eLife.89951 (PMC10491434; doi:10.7554/eLife.89951)

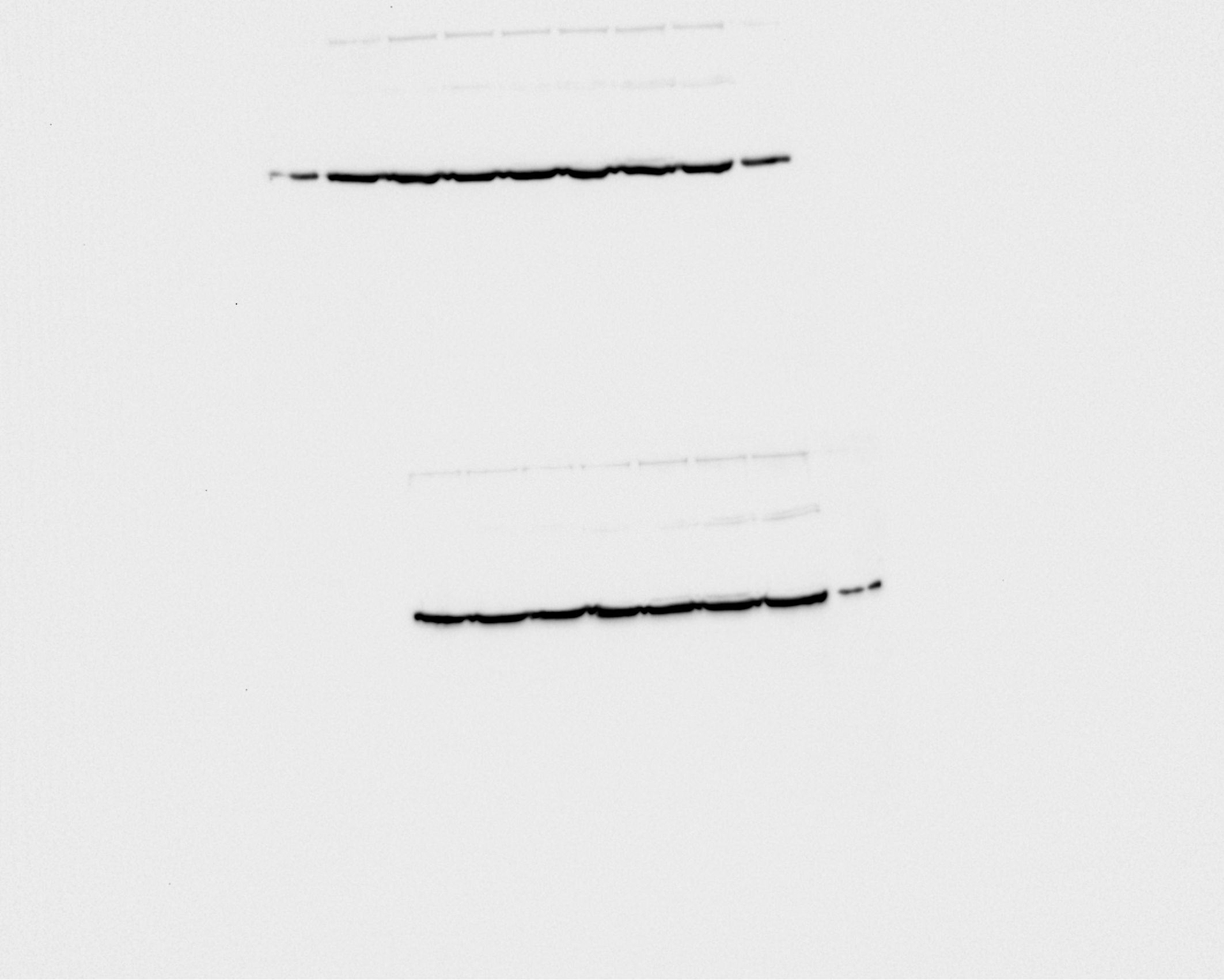

Supplement: Figure 1—source data 1. [file elife-89951-fig1-data1.zip › Figure 1-source data 1/ACTIN_Figure 1-source data 1/Run 2018-10-16 16h34m57s 29.736s(Chemiluminescence).tif]

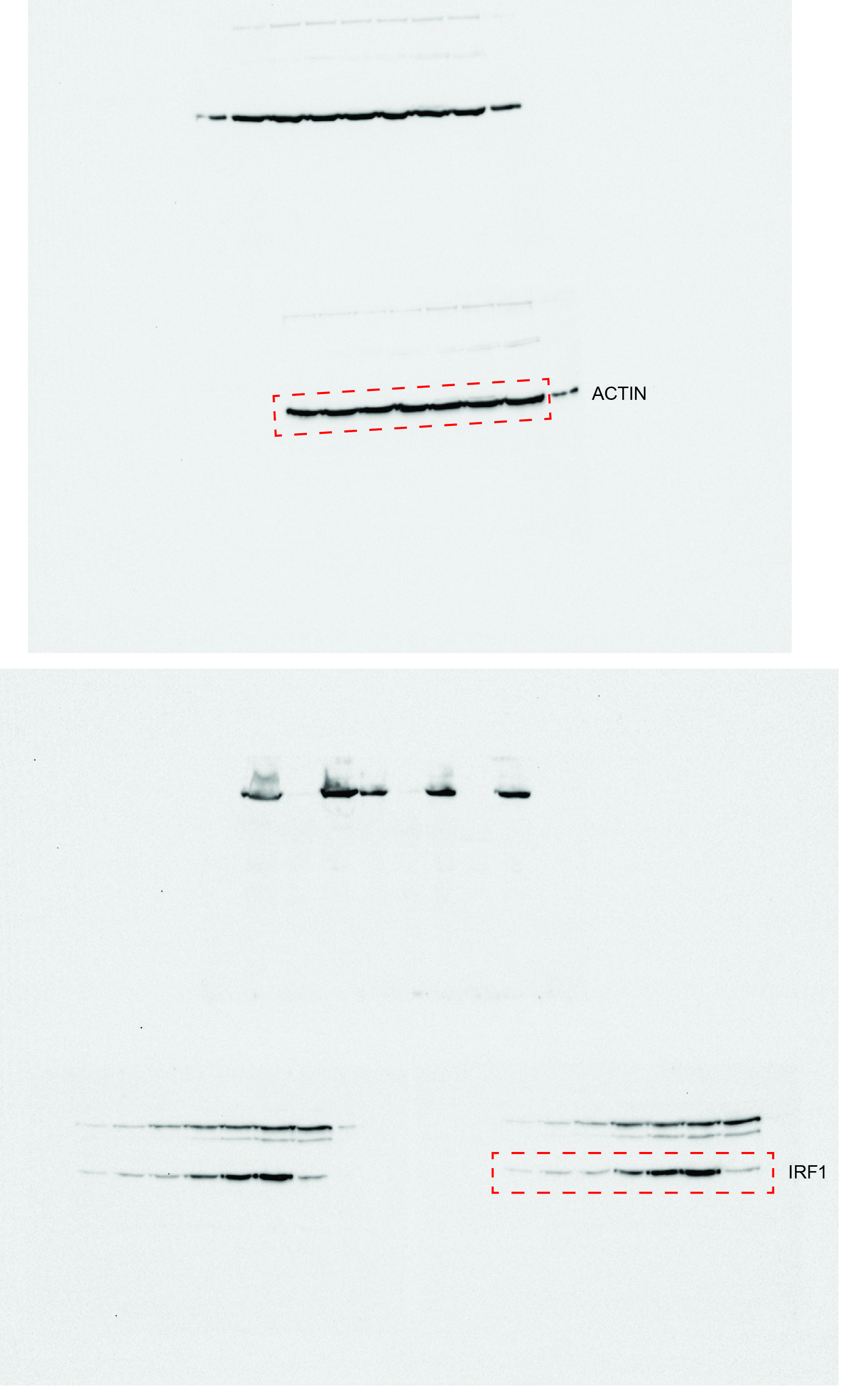

Supplement: Figure 1—source data 1. [file elife-89951-fig1-data1.zip › Figure 1-source data 1/Figure 1-source data 1.jpg]

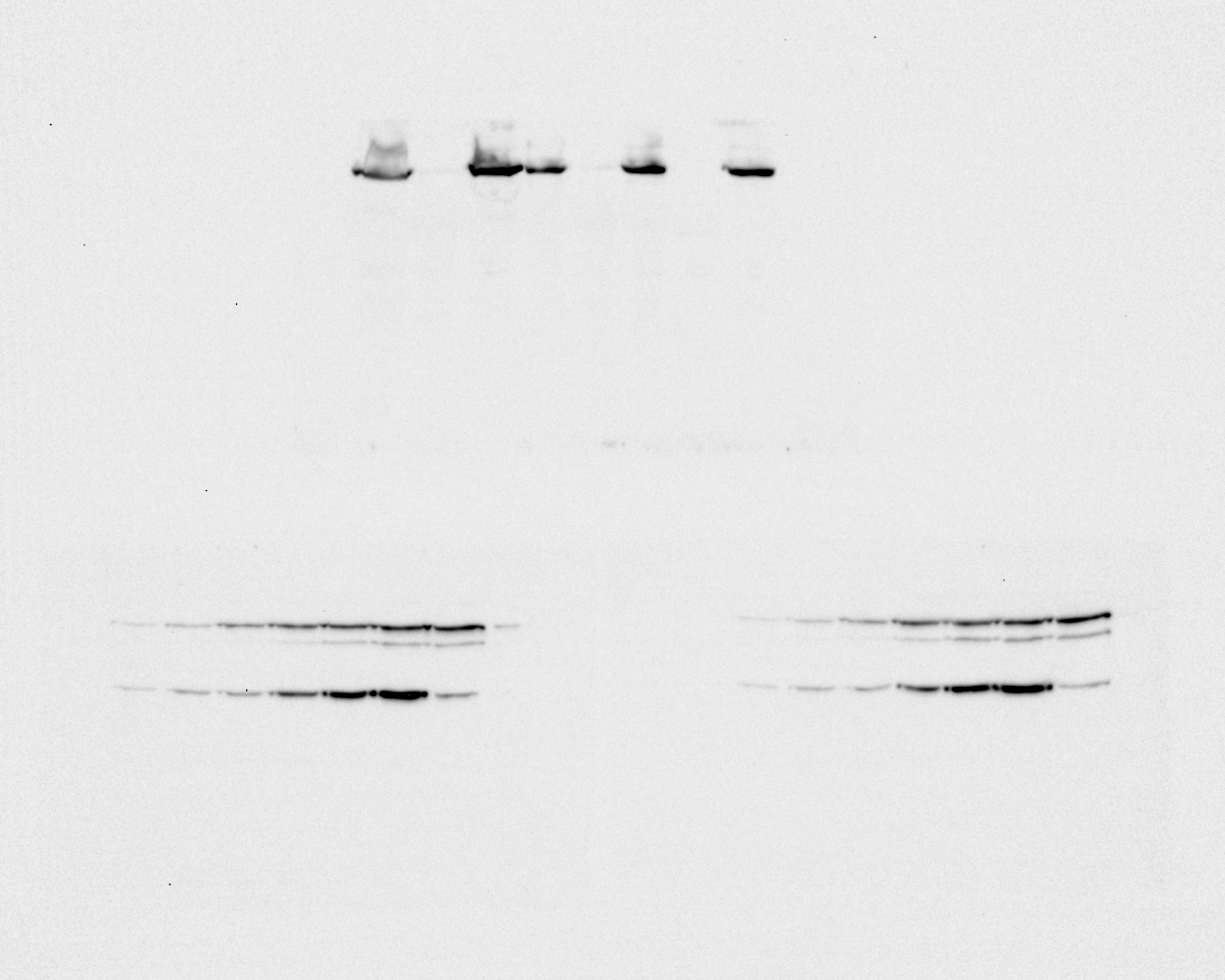

Supplement: Figure 1—source data 1. [file elife-89951-fig1-data1.zip › Figure 1-source data 1/IRF1_Figure 1-source data 1/Adriana 2018-10-16 13h22m36s 177.170s(Chemiluminescence).tif]

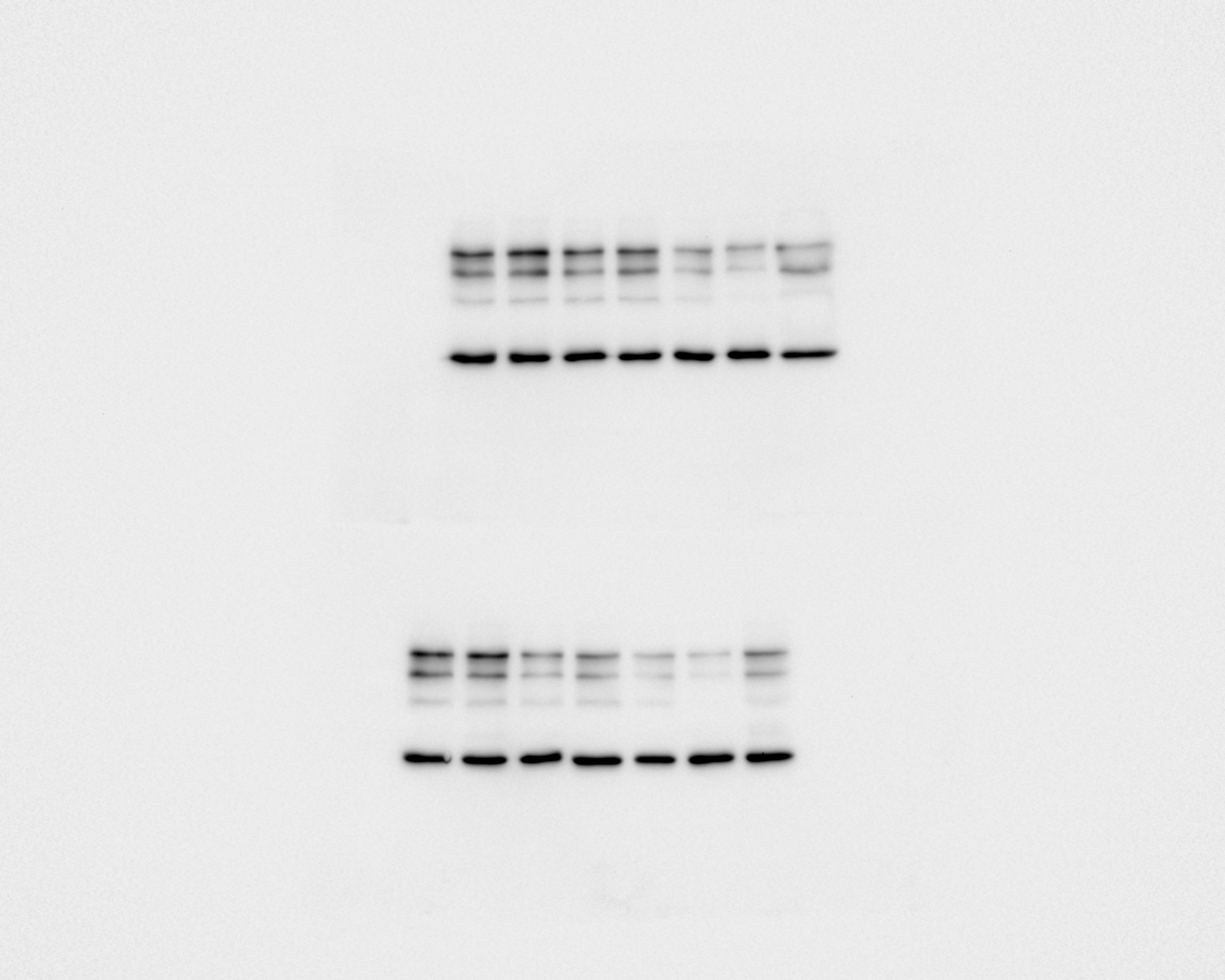

Supplement: Figure 1—source data 2. [file elife-89951-fig1-data2.zip › Figure 1-source data 2/ACTIN_Figure 1-source data 2/Versteeg 2022-02-15 12h33m15s 38.578s(Chemiluminescence).jpg]

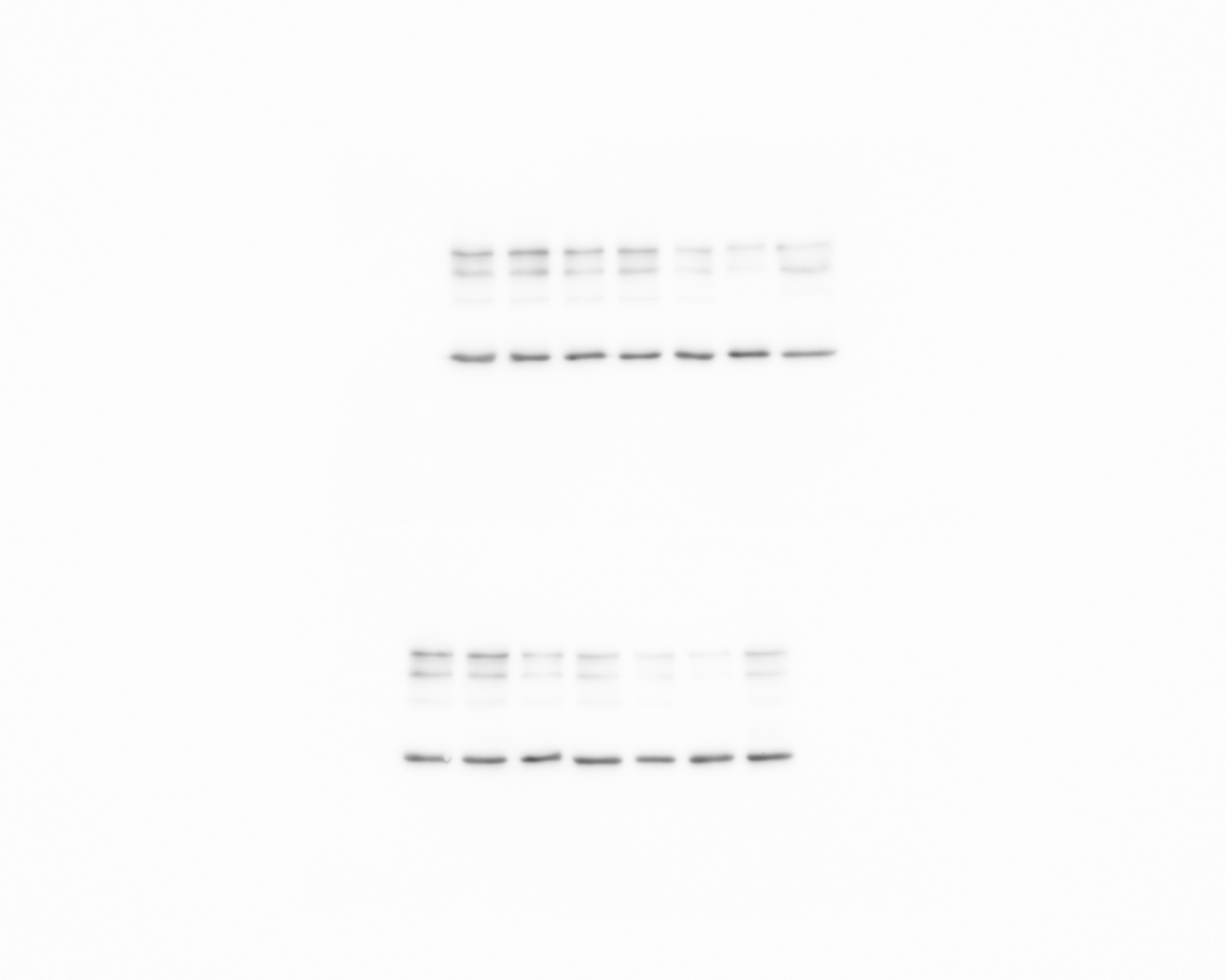

Supplement: Figure 1—source data 2. [file elife-89951-fig1-data2.zip › Figure 1-source data 2/ACTIN_Figure 1-source data 2/Versteeg 2022-02-15 12h33m15s 38.578s(Chemiluminescence).raw16.tif]

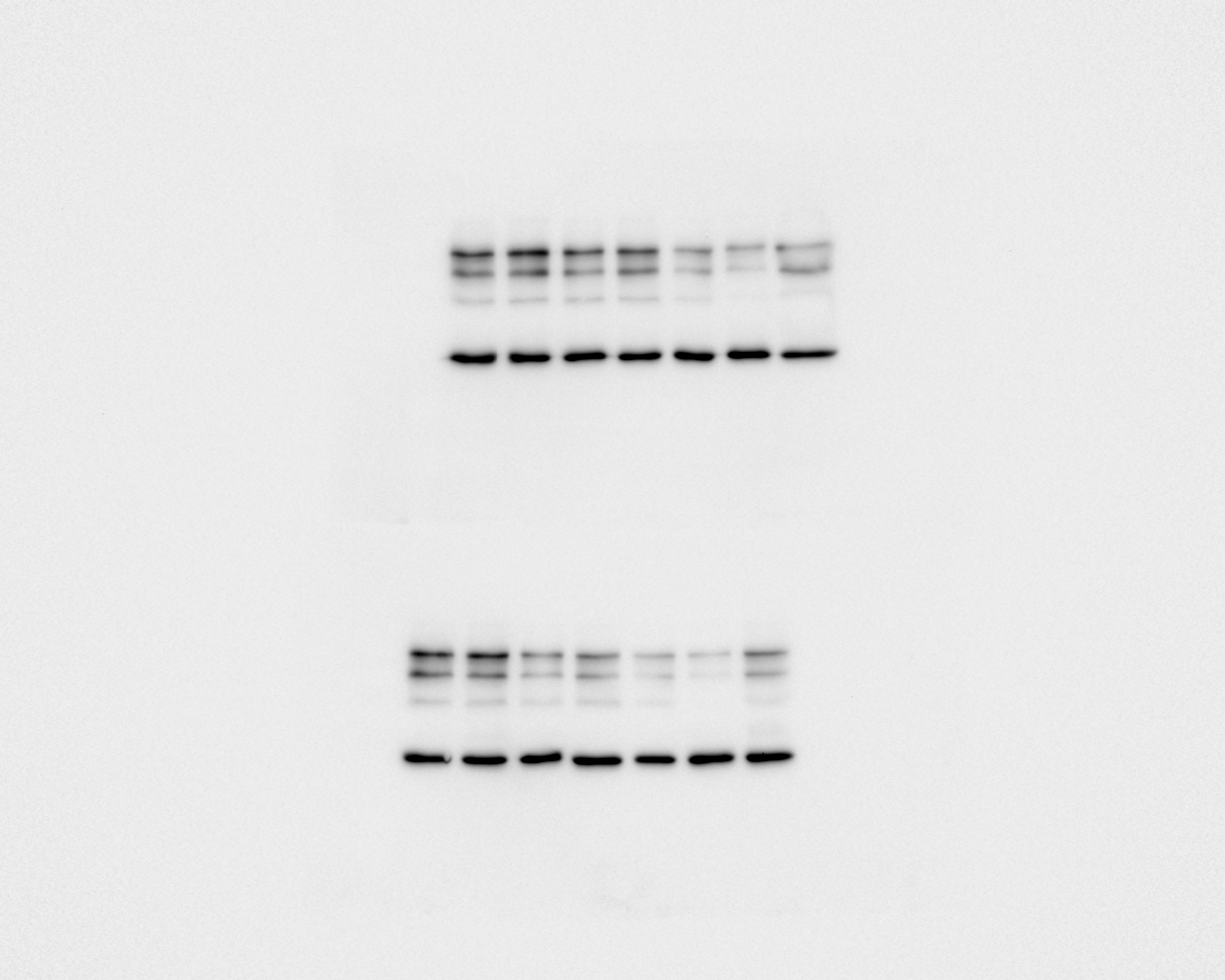

Supplement: Figure 1—source data 2. [file elife-89951-fig1-data2.zip › Figure 1-source data 2/ACTIN_Figure 1-source data 2/Versteeg 2022-02-15 12h33m15s 38.578s(Chemiluminescence).tif]

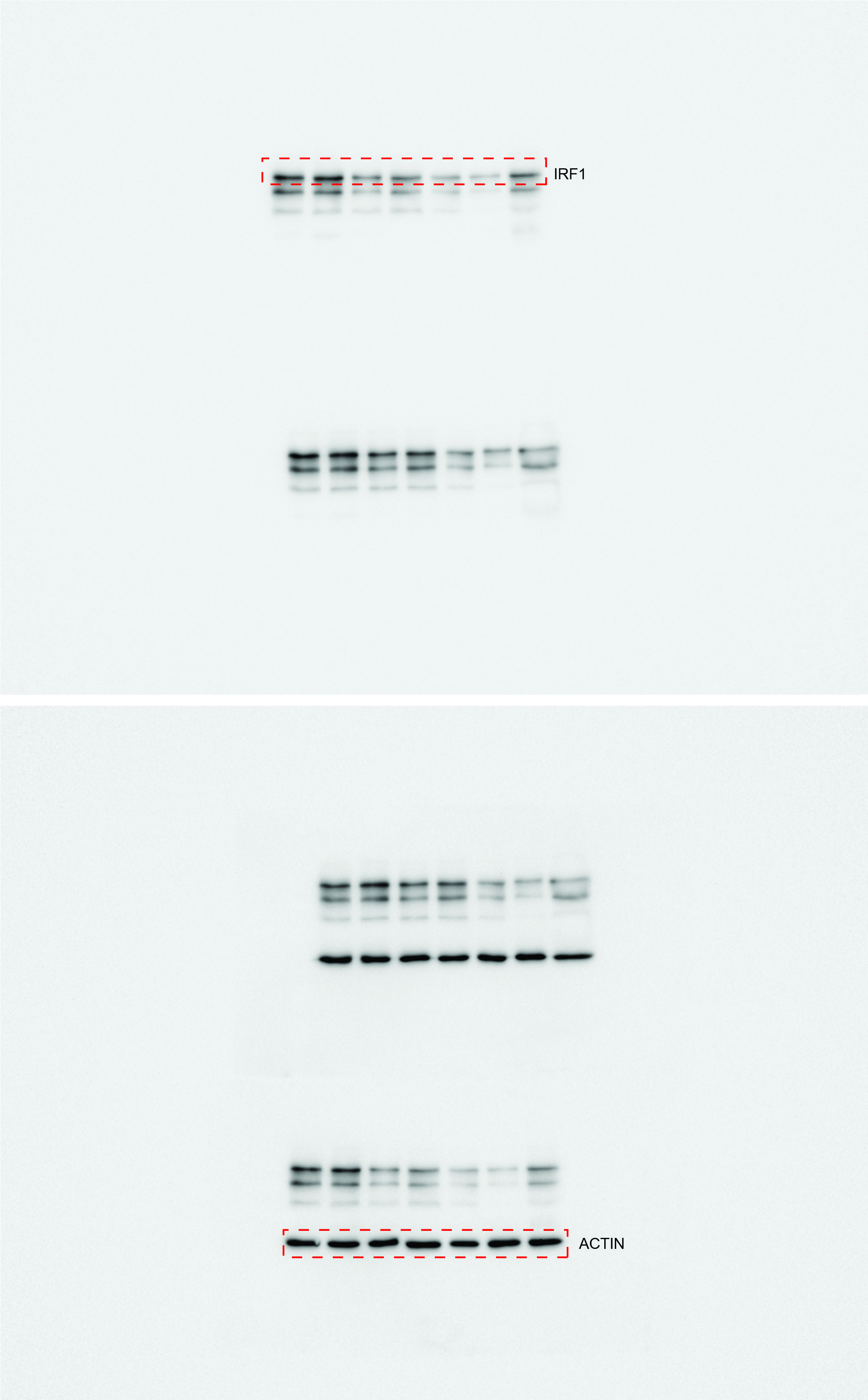

Supplement: Figure 1—source data 2. [file elife-89951-fig1-data2.zip › Figure 1-source data 2/Figure 1-source data 2.jpg]

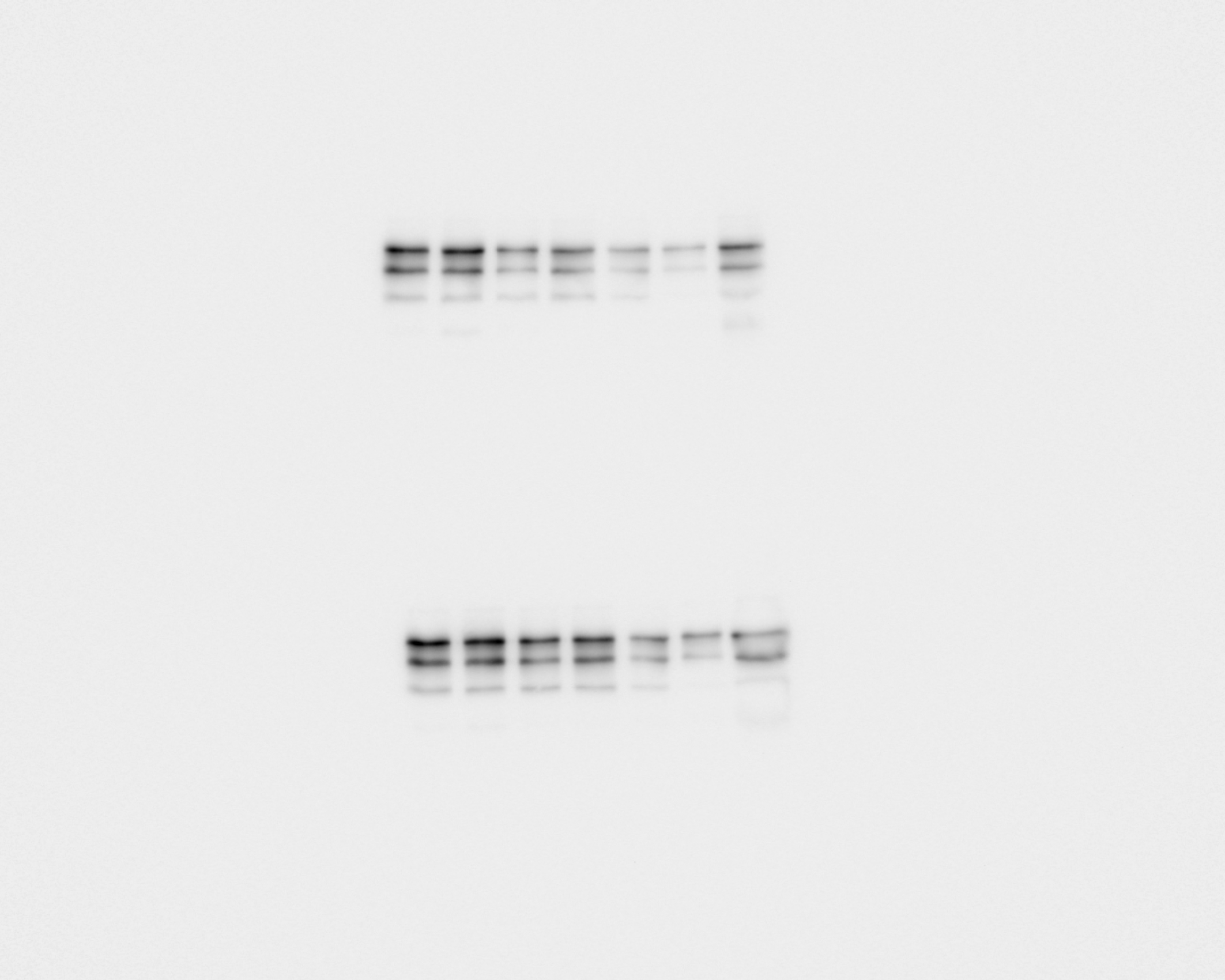

Supplement: Figure 1—source data 2. [file elife-89951-fig1-data2.zip › Figure 1-source data 2/IRF1_Figure 1-source data 2/Versteeg 2022-02-15 10h27m33s 32.472s(Chemiluminescence).jpg]

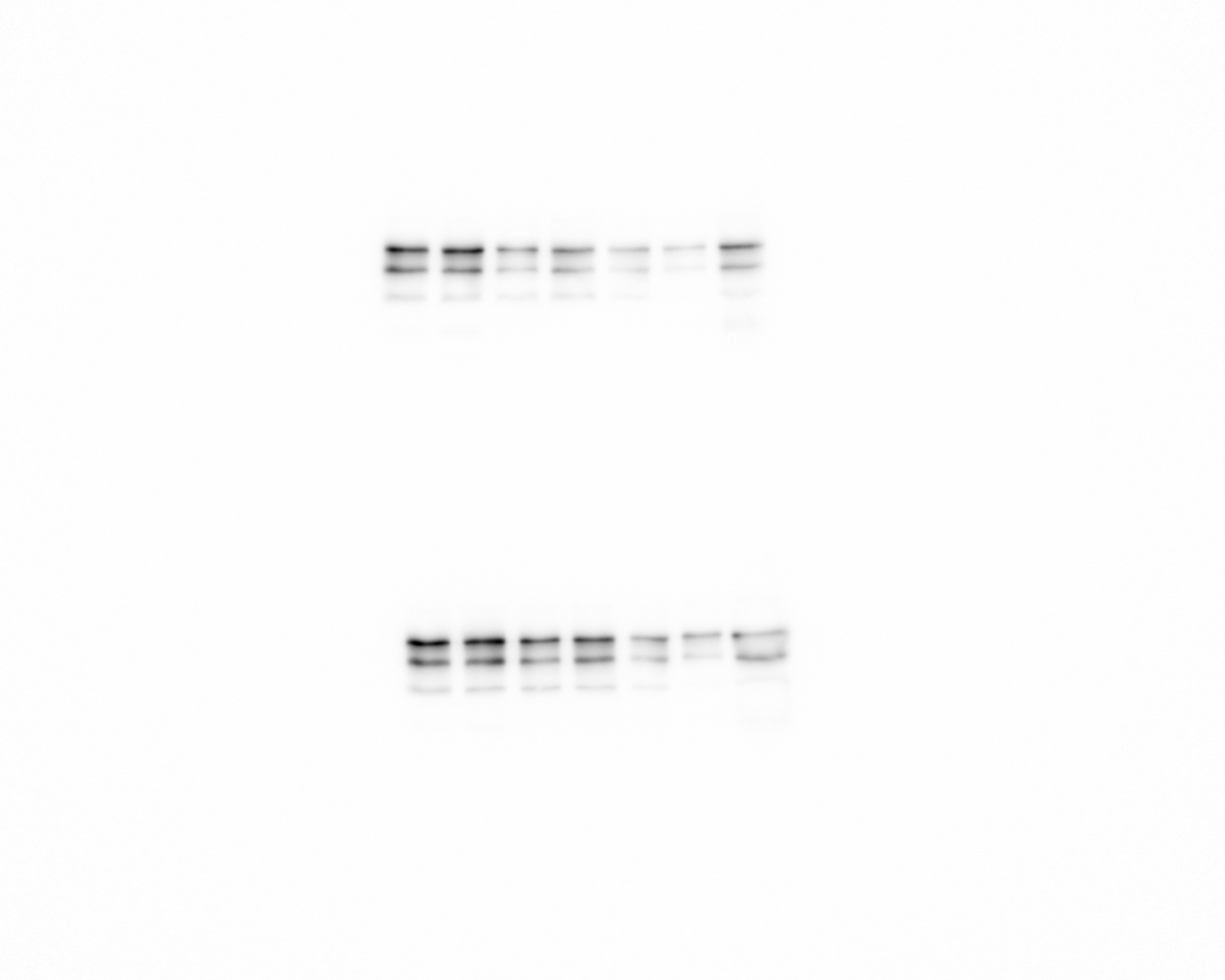

Supplement: Figure 1—source data 2. [file elife-89951-fig1-data2.zip › Figure 1-source data 2/IRF1_Figure 1-source data 2/Versteeg 2022-02-15 10h27m33s 32.472s(Chemiluminescence).raw16.tif]

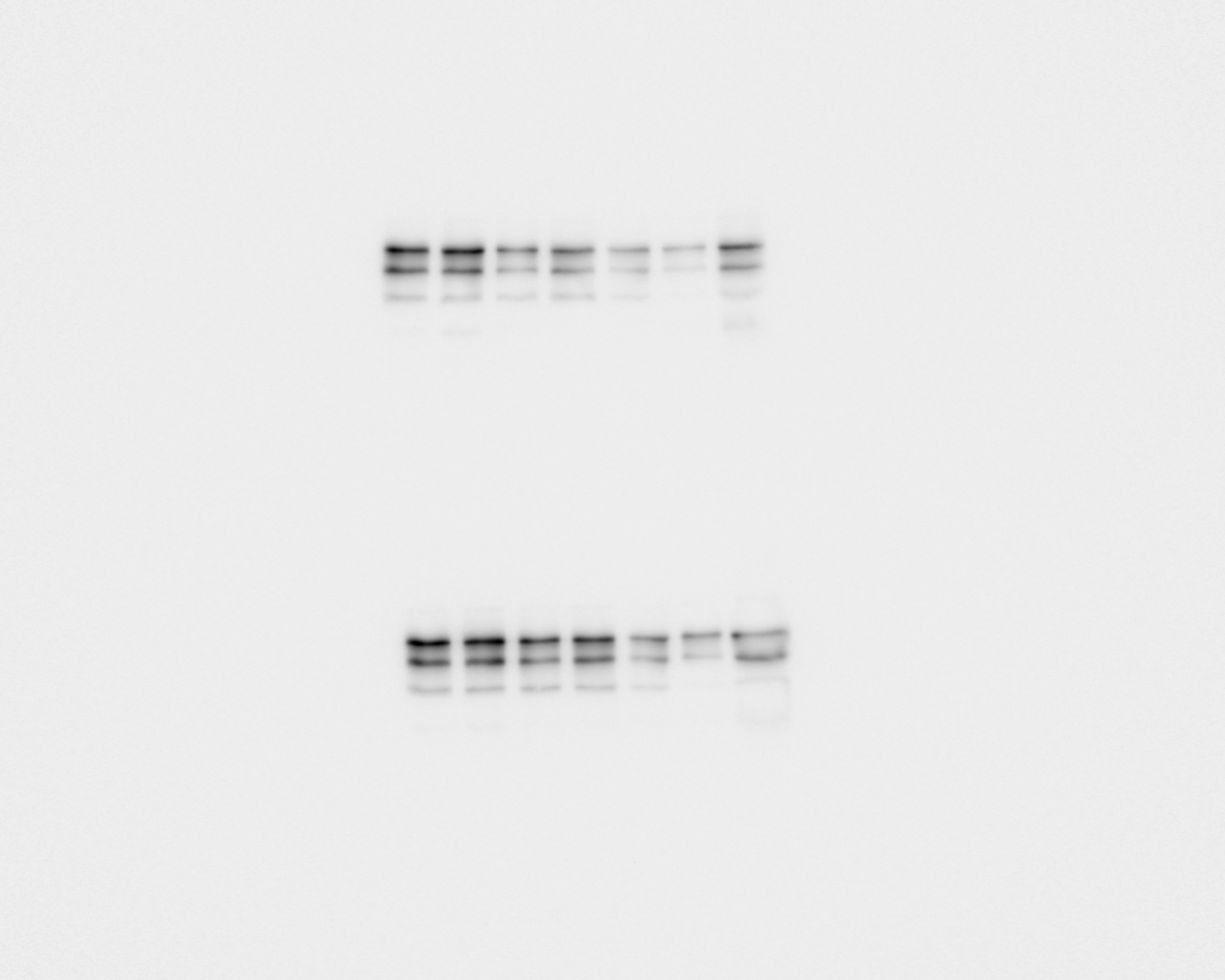

Supplement: Figure 1—source data 2. [file elife-89951-fig1-data2.zip › Figure 1-source data 2/IRF1_Figure 1-source data 2/Versteeg 2022-02-15 10h27m33s 32.472s(Chemiluminescence).tif]

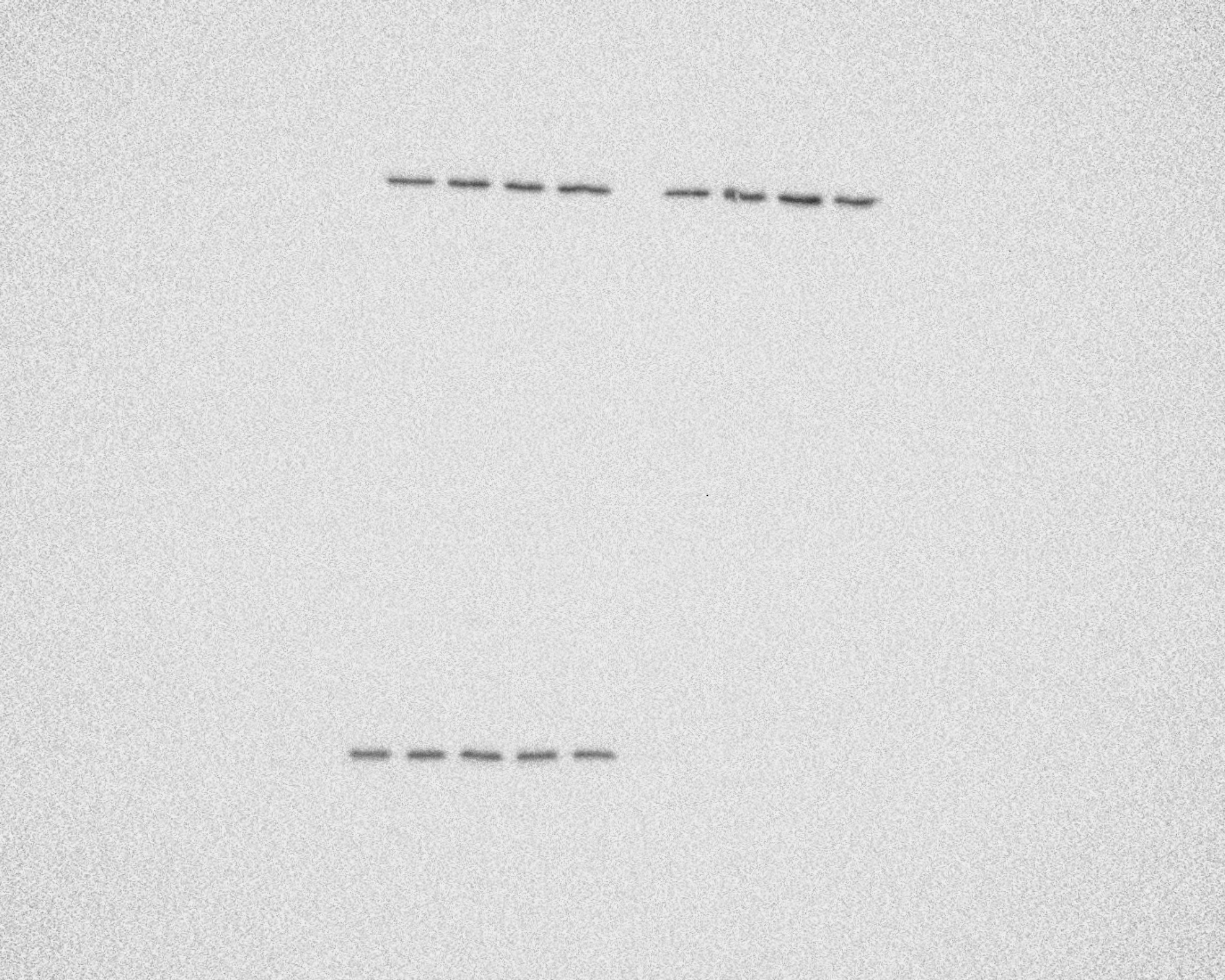

Supplement: Figure 1—source data 3. [file elife-89951-fig1-data3.zip › Figure 1-source data 3/ACTIN_Figure 1-source data 3/Versteeg 2022-02-23 13h54m07s 119.944s(Chemiluminescence).jpg]

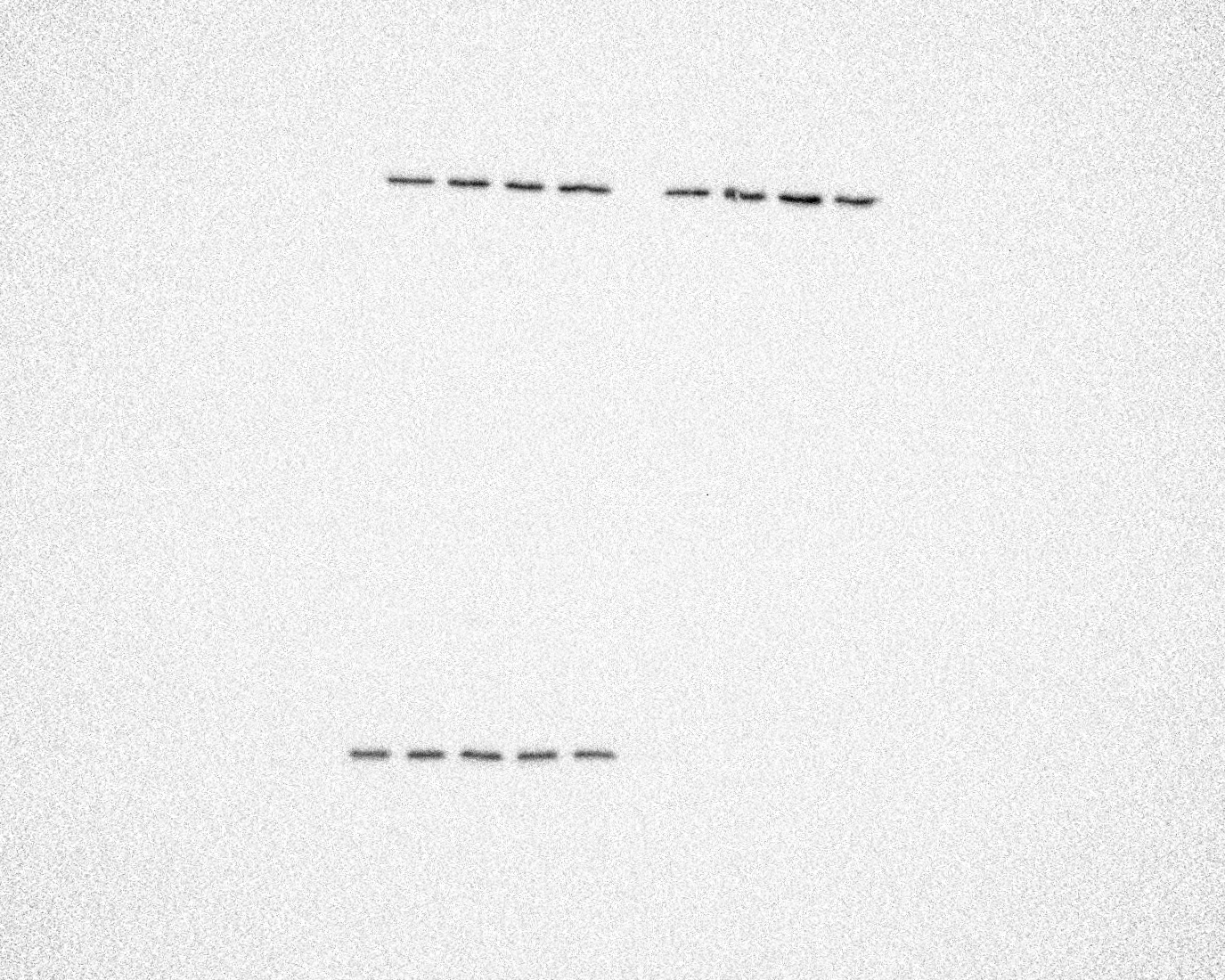

Supplement: Figure 1—source data 3. [file elife-89951-fig1-data3.zip › Figure 1-source data 3/ACTIN_Figure 1-source data 3/Versteeg 2022-02-23 13h54m07s 119.944s(Chemiluminescence).raw16.tif]

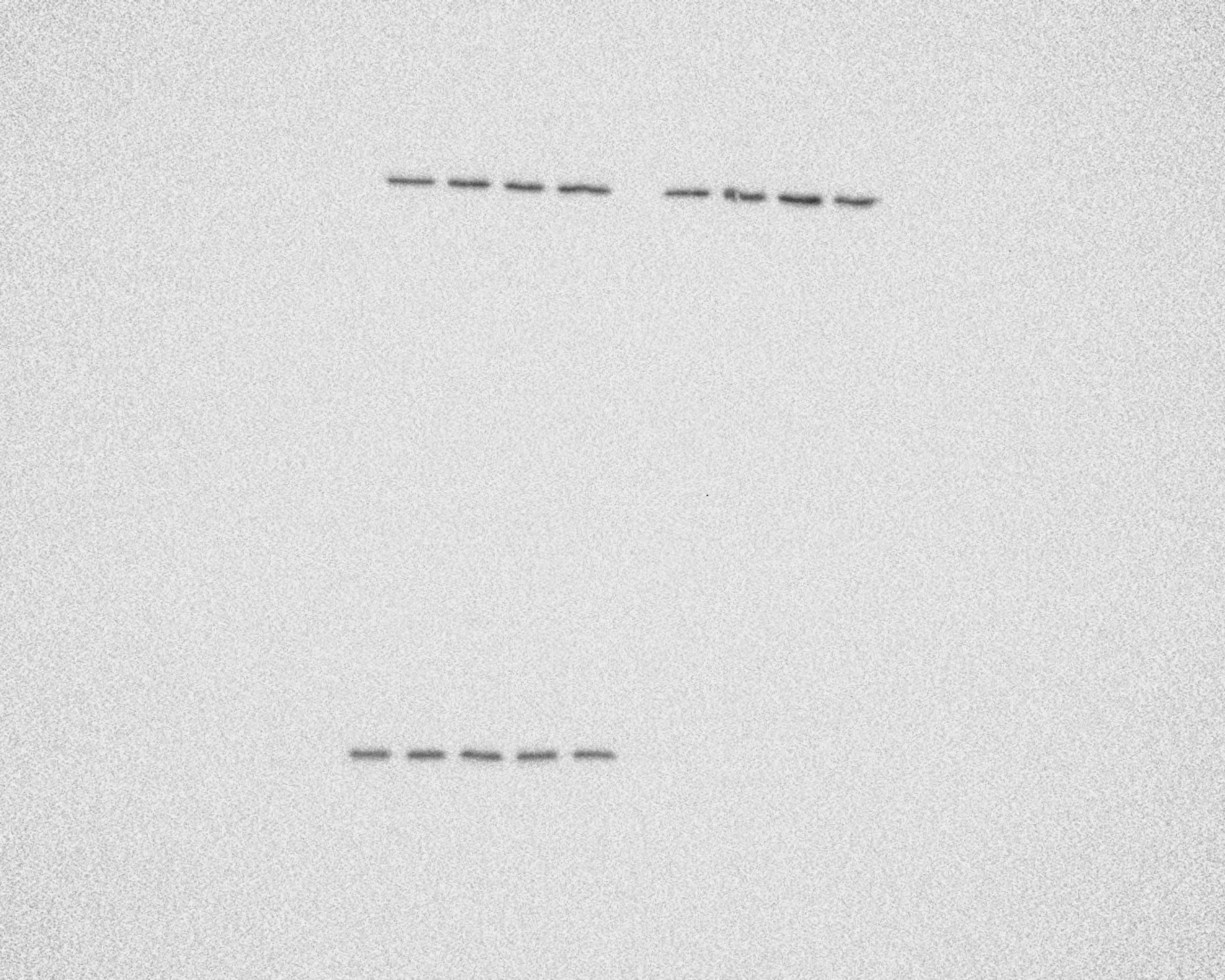

Supplement: Figure 1—source data 3. [file elife-89951-fig1-data3.zip › Figure 1-source data 3/ACTIN_Figure 1-source data 3/Versteeg 2022-02-23 13h54m07s 119.944s(Chemiluminescence).tif]

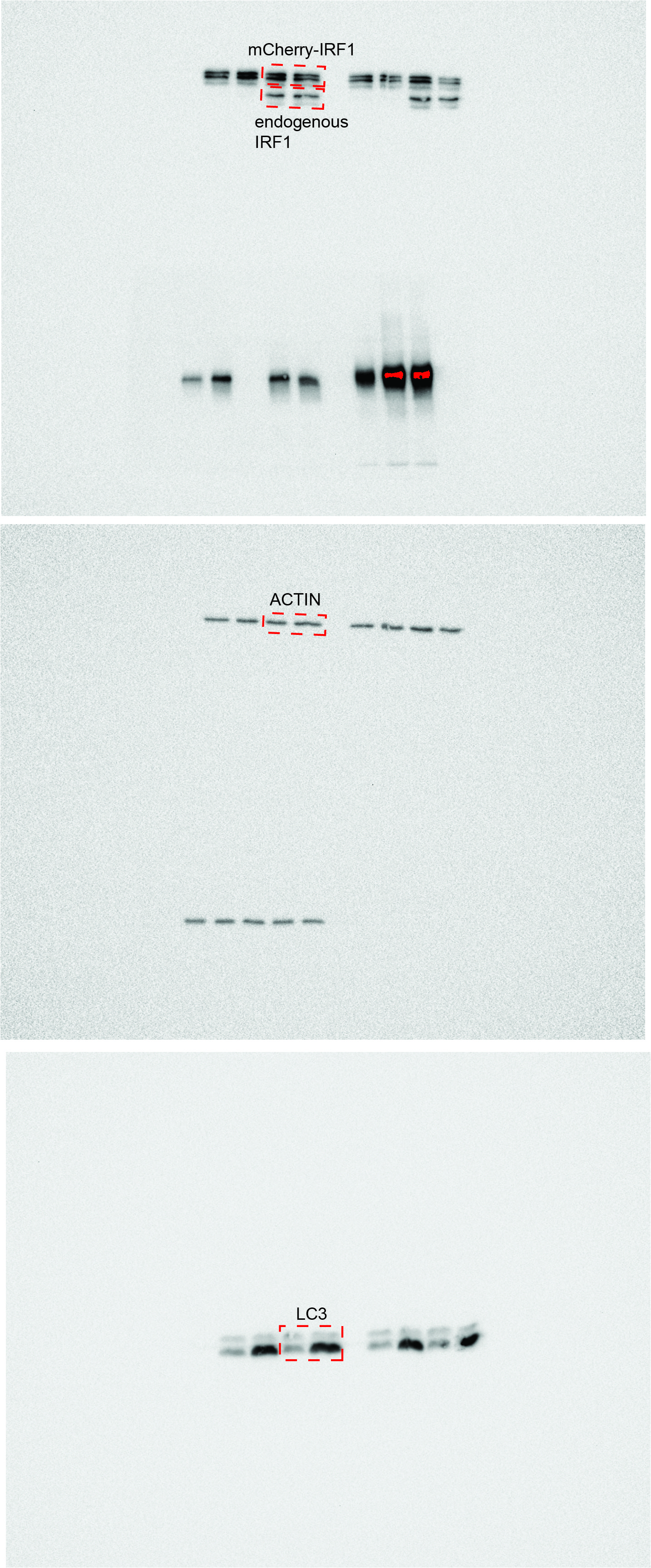

Supplement: Figure 1—source data 3. [file elife-89951-fig1-data3.zip › Figure 1-source data 3/Figure 1-source data 3.jpg]

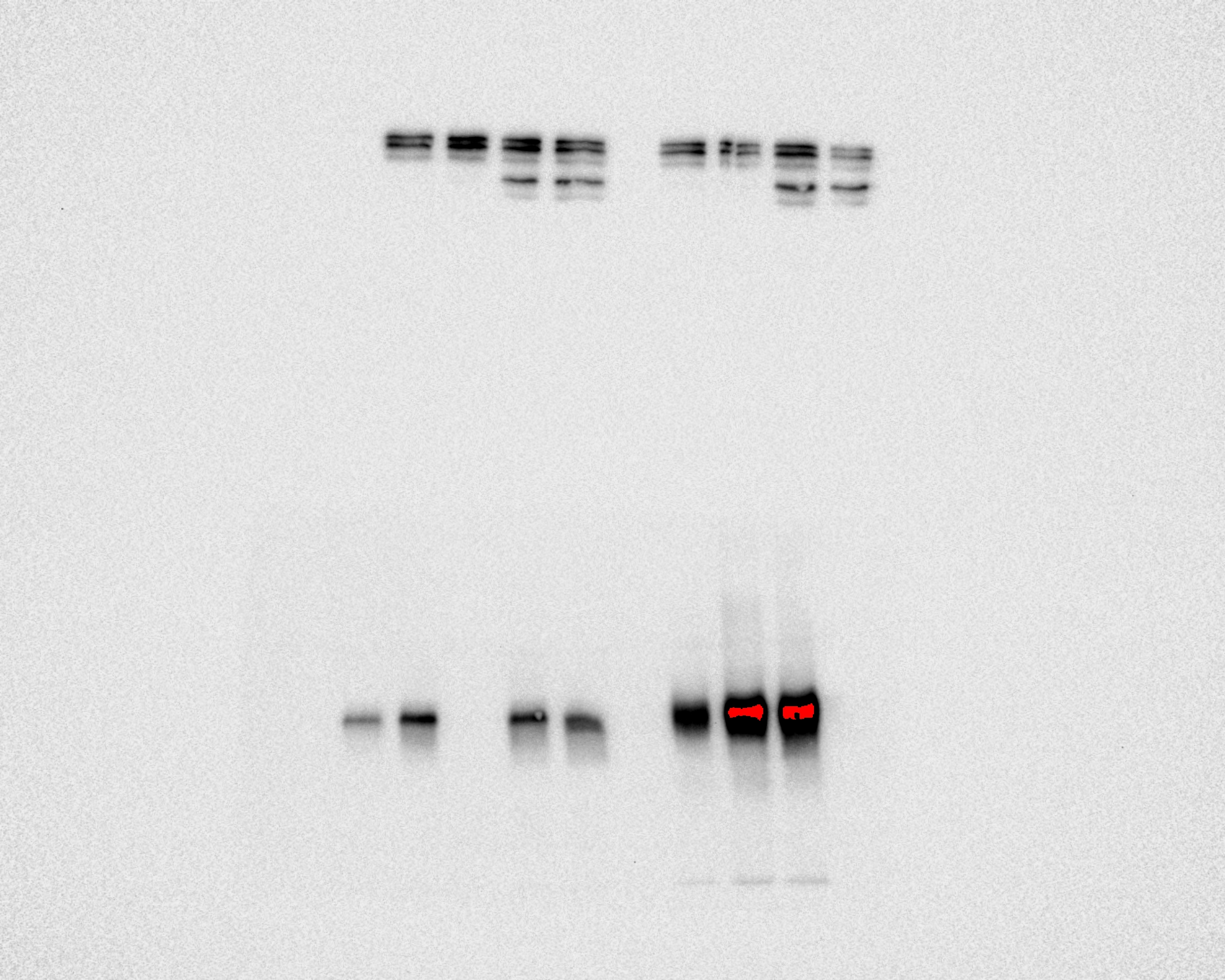

Supplement: Figure 1—source data 3. [file elife-89951-fig1-data3.zip › Figure 1-source data 3/IRF1_Figure 1-source data 3/Versteeg 2022-02-23 11h32m22s 299.990s(Chemiluminescence).jpg]

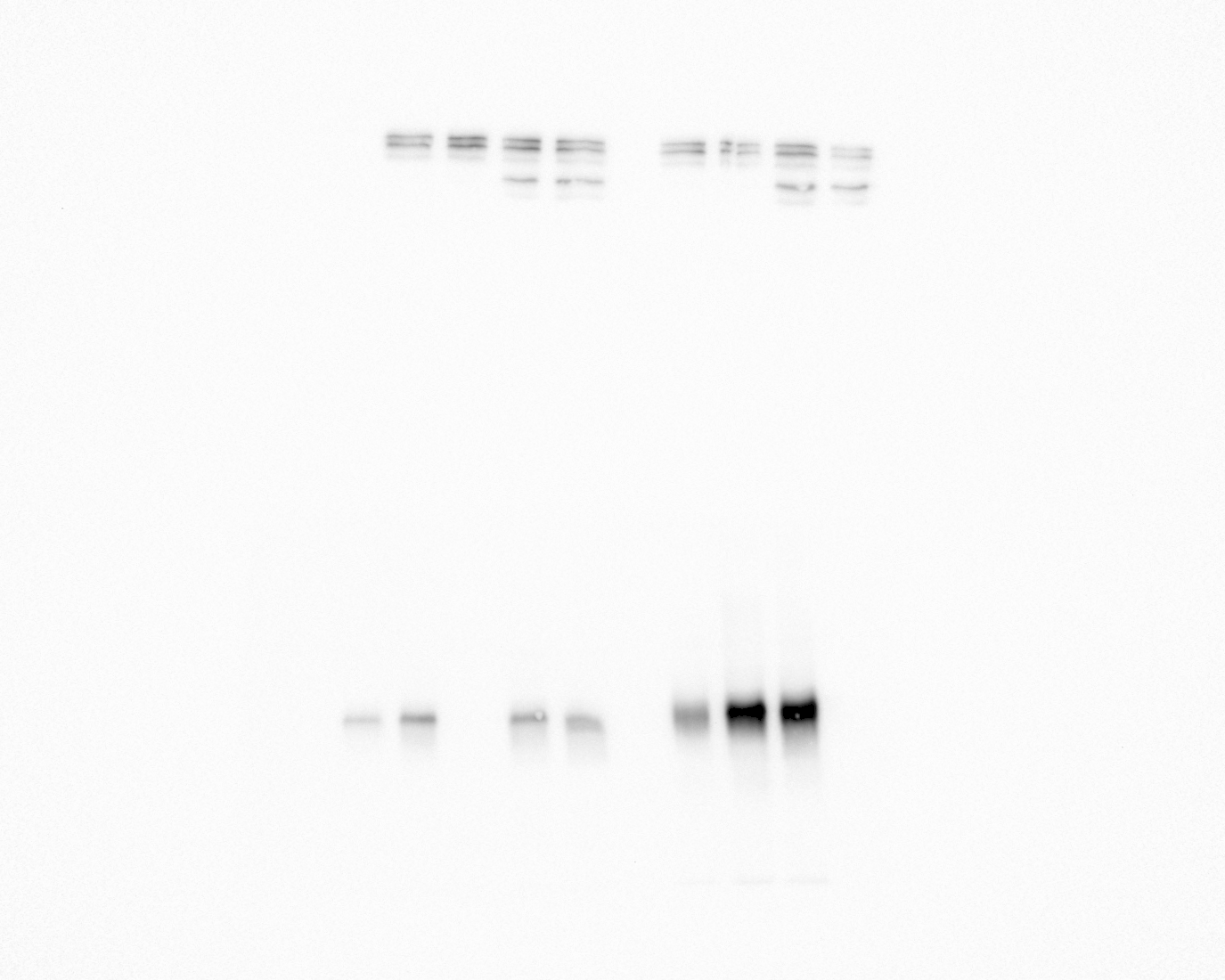

Supplement: Figure 1—source data 3. [file elife-89951-fig1-data3.zip › Figure 1-source data 3/IRF1_Figure 1-source data 3/Versteeg 2022-02-23 11h32m22s 299.990s(Chemiluminescence).raw16.tif]

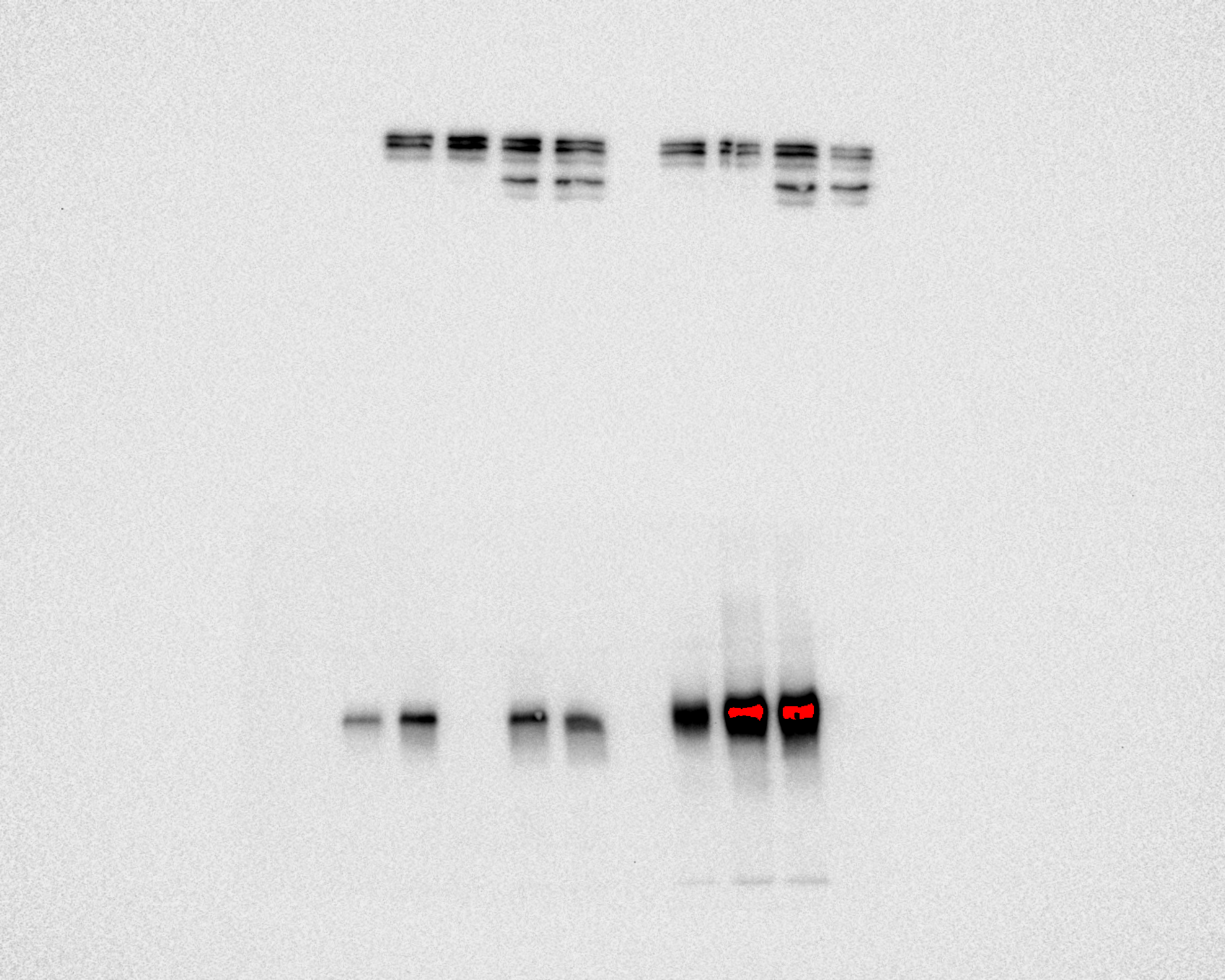

Supplement: Figure 1—source data 3. [file elife-89951-fig1-data3.zip › Figure 1-source data 3/IRF1_Figure 1-source data 3/Versteeg 2022-02-23 11h32m22s 299.990s(Chemiluminescence).tif]

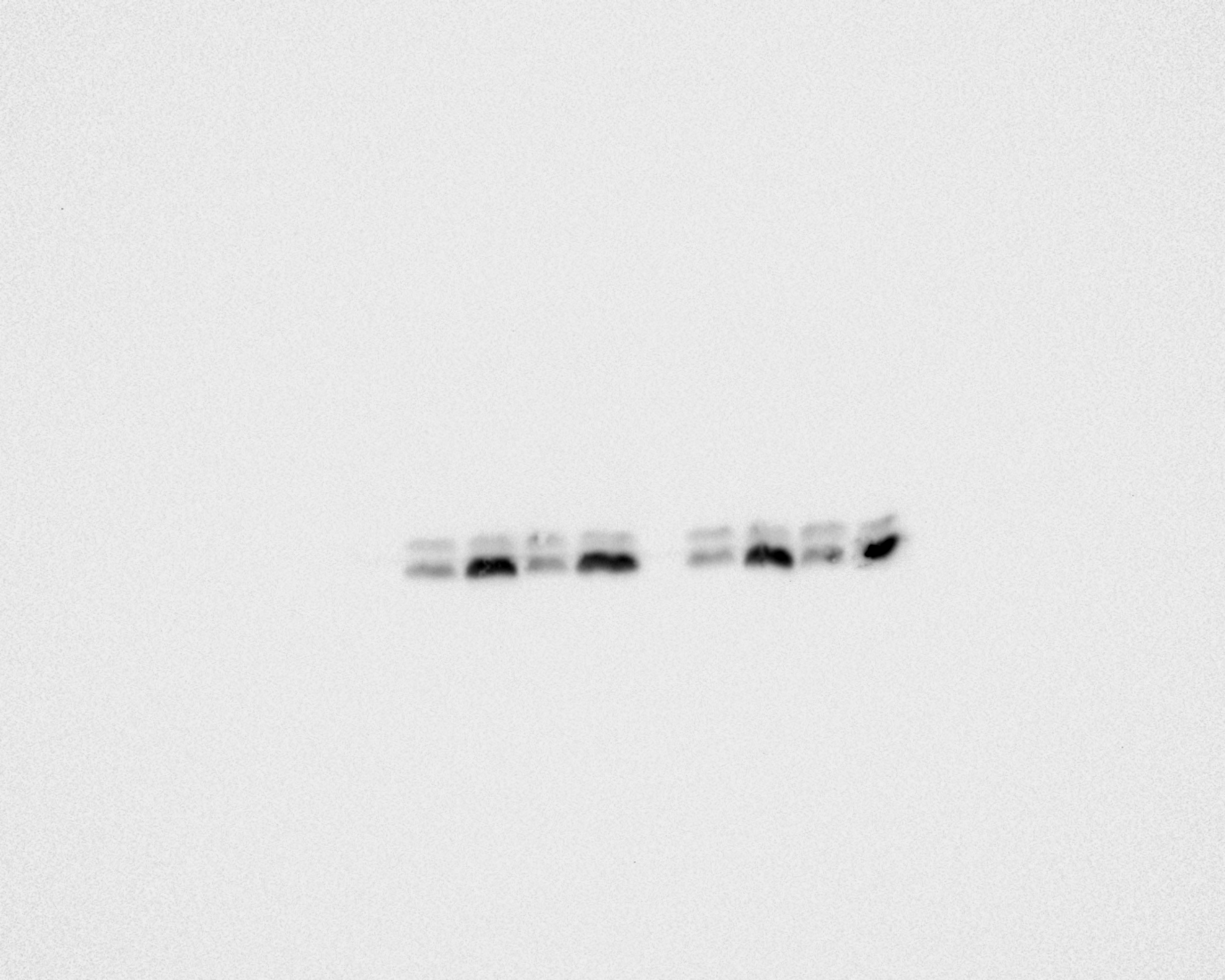

Supplement: Figure 1—source data 3. [file elife-89951-fig1-data3.zip › Figure 1-source data 3/LC3_Figure 1-source data 3/Versteeg 2022-03-04 13h45m05s 165.960s(Chemiluminescence).jpg]

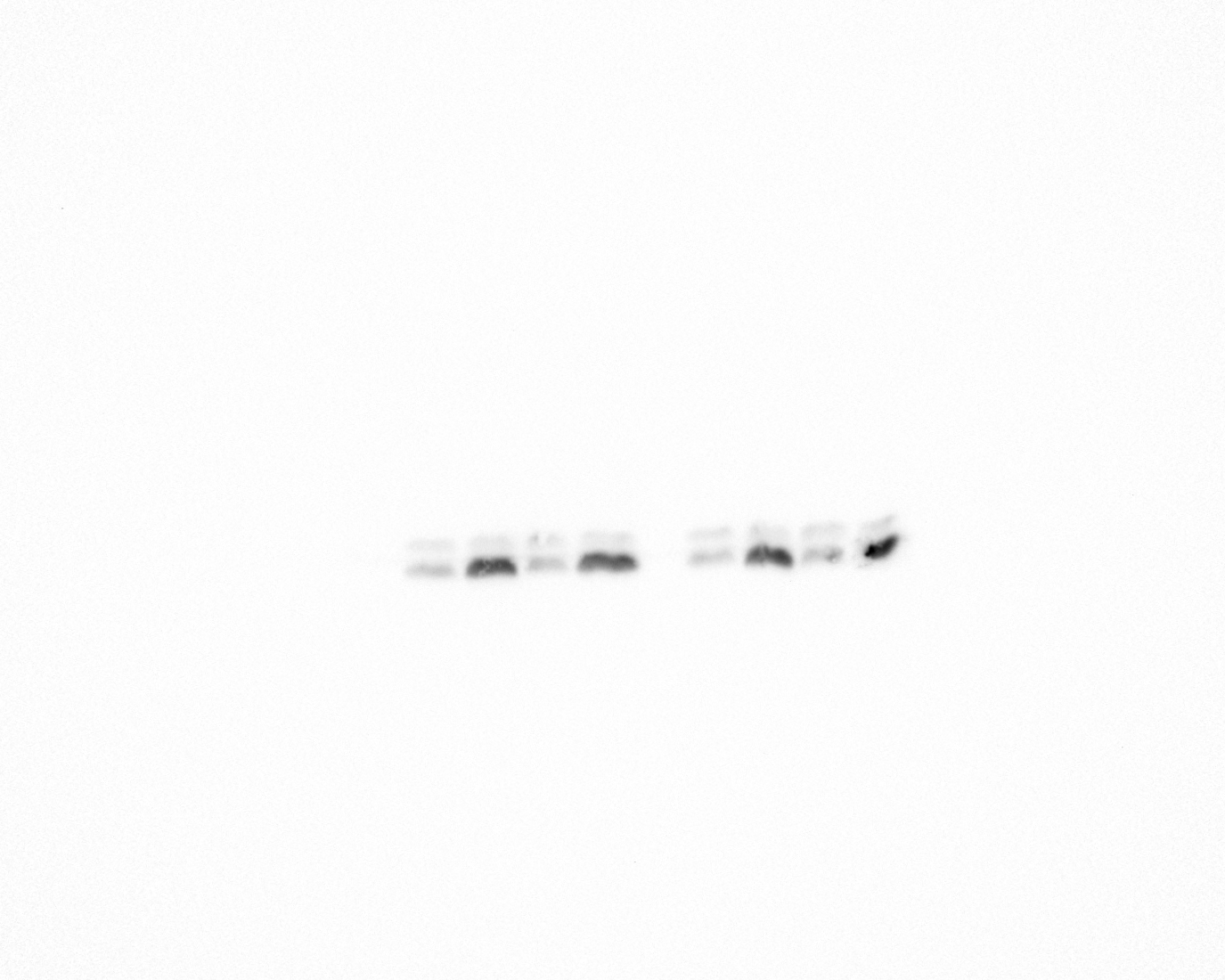

Supplement: Figure 1—source data 3. [file elife-89951-fig1-data3.zip › Figure 1-source data 3/LC3_Figure 1-source data 3/Versteeg 2022-03-04 13h45m05s 165.960s(Chemiluminescence).raw16.tif]

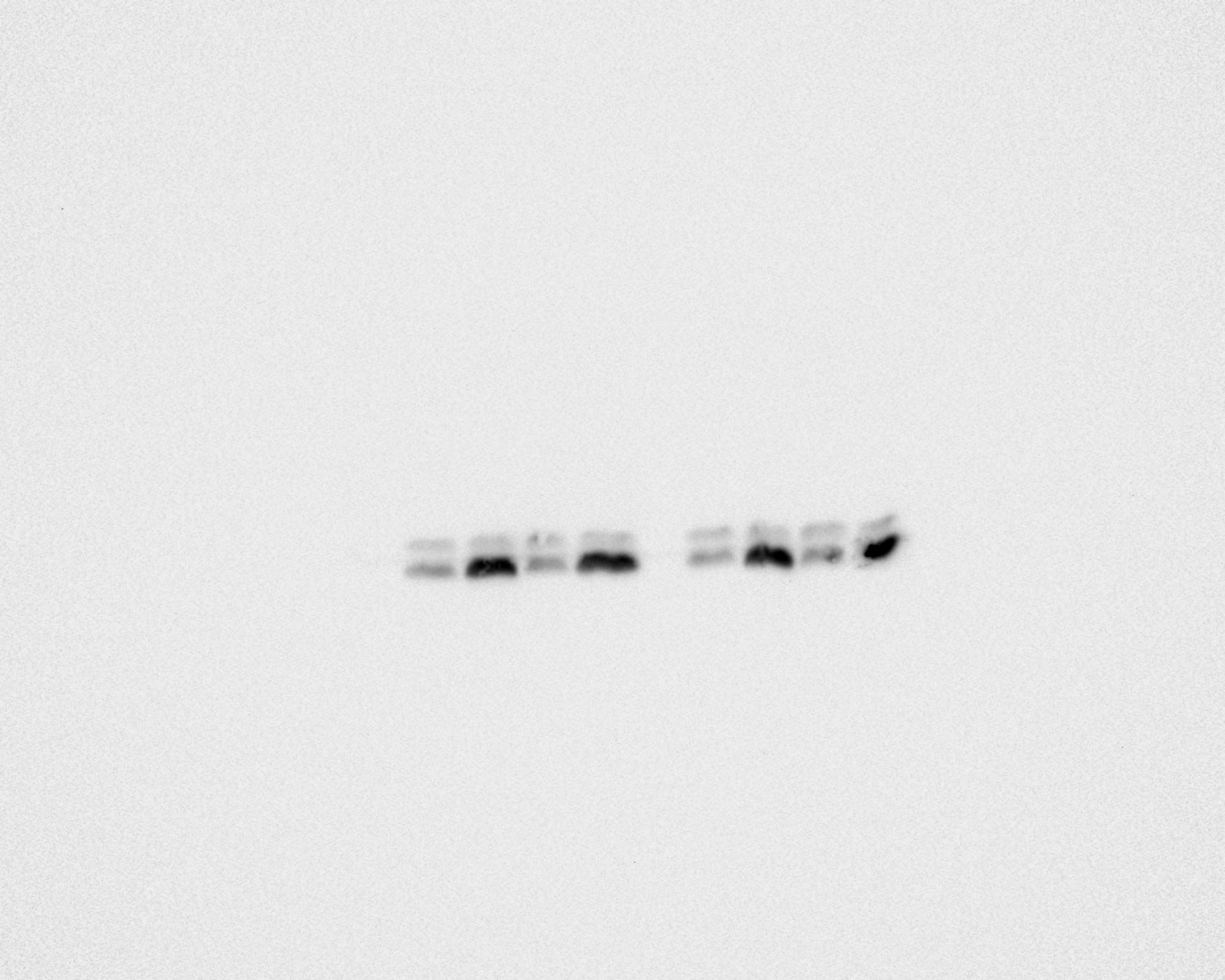

Supplement: Figure 1—source data 3. [file elife-89951-fig1-data3.zip › Figure 1-source data 3/LC3_Figure 1-source data 3/Versteeg 2022-03-04 13h45m05s 165.960s(Chemiluminescence).tif]

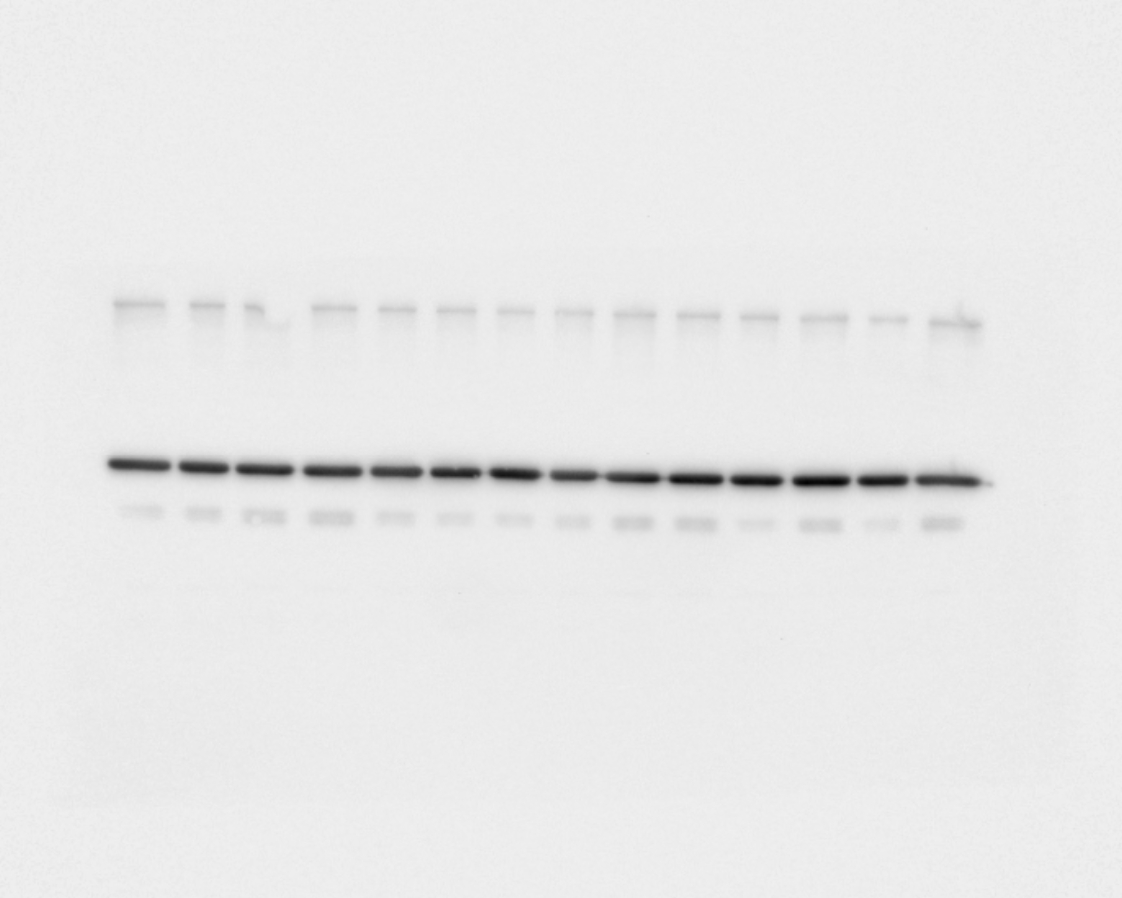

Supplement: Figure 1—figure supplement 1—source data 1. [file elife-89951-fig1-figsupp1-data1.zip › Figure 1-figure supplement 1-source data 1/ACTIN_Figure 1-figure supplement 1-source data 1/Versteeg 2023-03-13 14h24m29s 13.204s(Chemiluminescence).jpg]

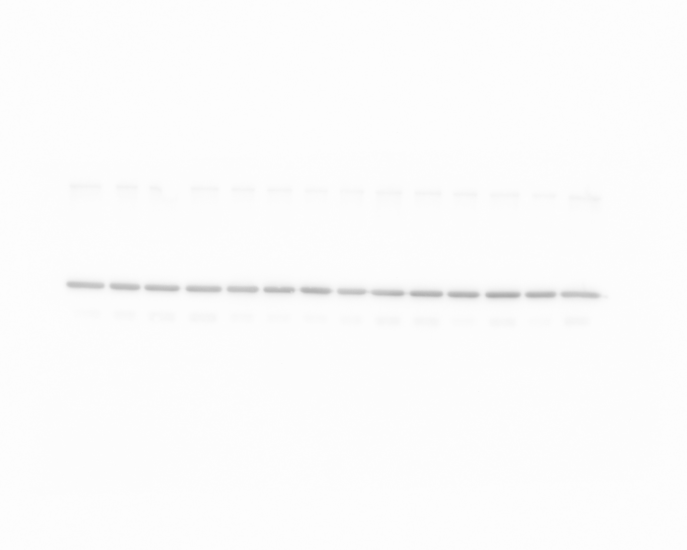

Supplement: Figure 1—figure supplement 1—source data 1. [file elife-89951-fig1-figsupp1-data1.zip › Figure 1-figure supplement 1-source data 1/ACTIN_Figure 1-figure supplement 1-source data 1/Versteeg 2023-03-13 14h24m29s 13.204s(Chemiluminescence).raw16.tif]

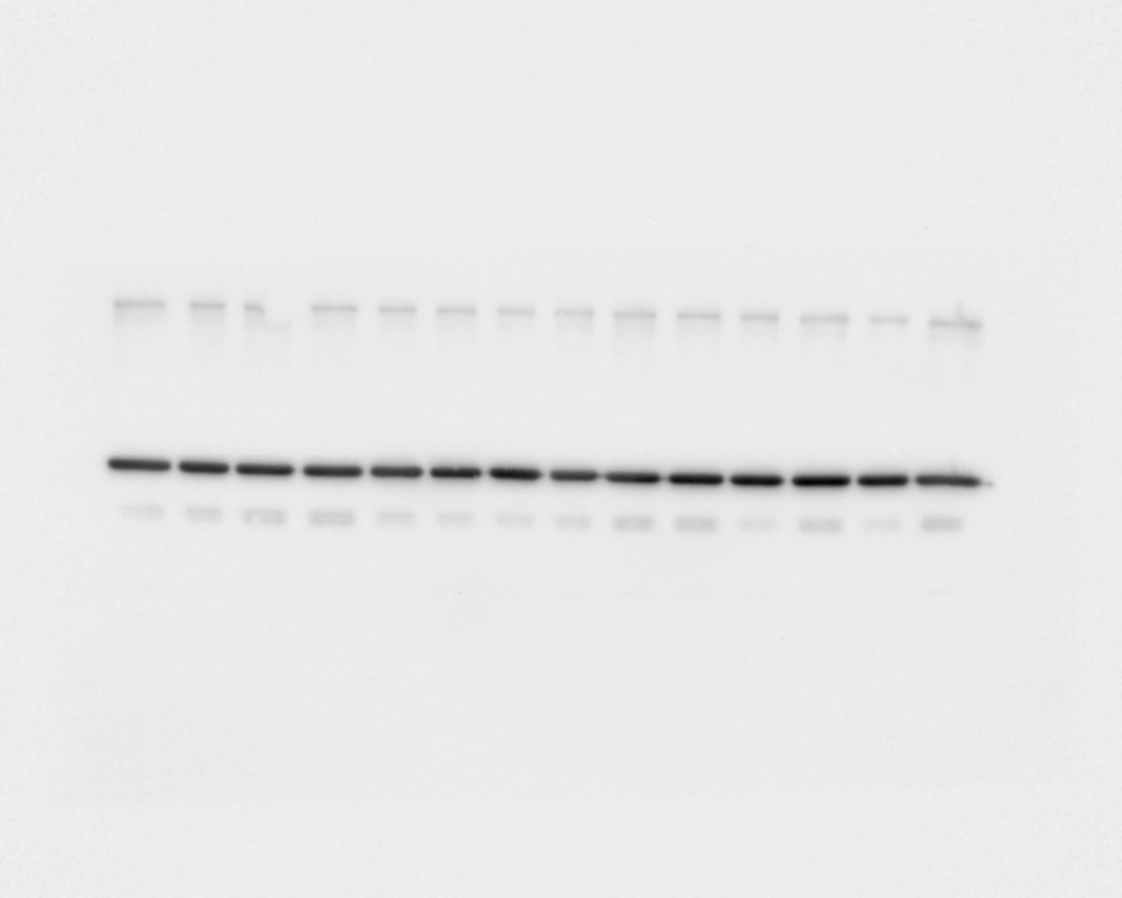

Supplement: Figure 1—figure supplement 1—source data 1. [file elife-89951-fig1-figsupp1-data1.zip › Figure 1-figure supplement 1-source data 1/ACTIN_Figure 1-figure supplement 1-source data 1/Versteeg 2023-03-13 14h24m29s 13.204s(Chemiluminescence).tif]

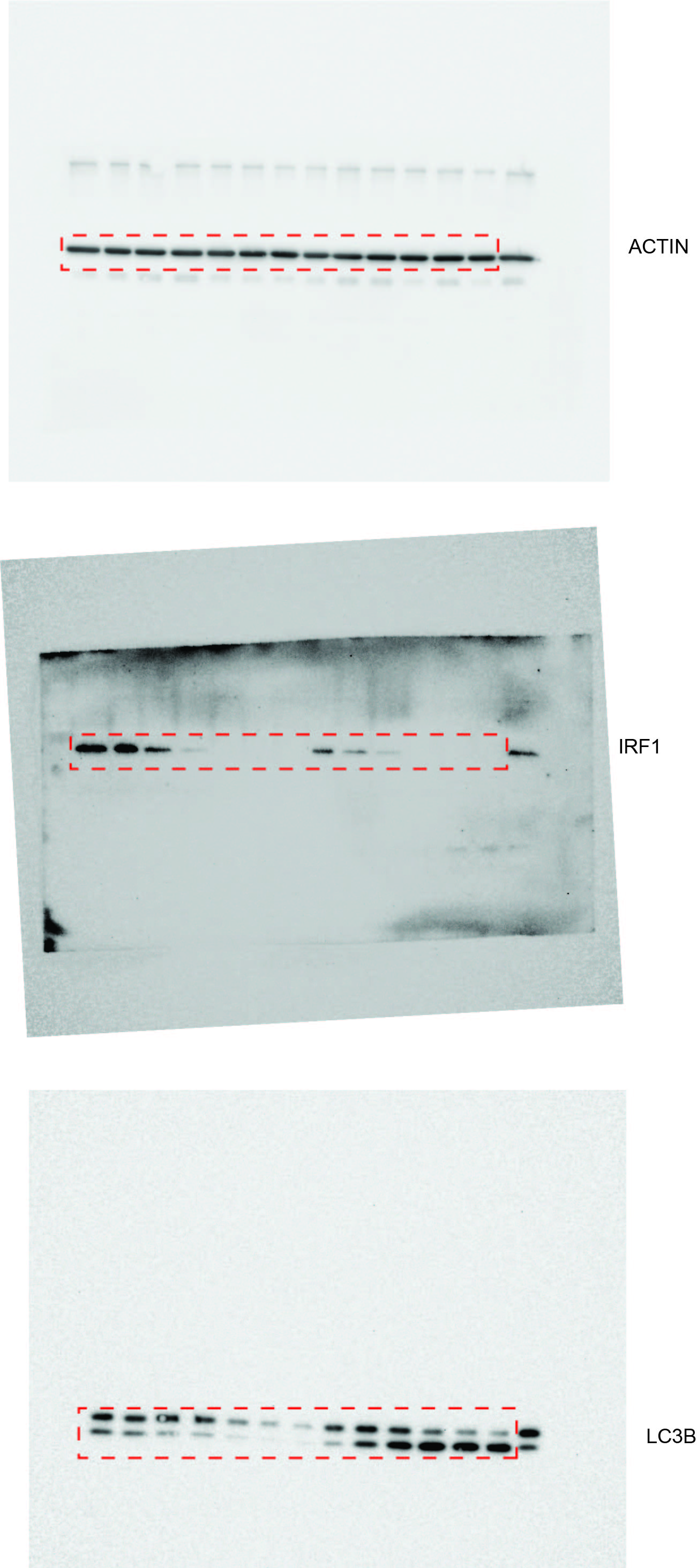

Supplement: Figure 1—figure supplement 1—source data 1. [file elife-89951-fig1-figsupp1-data1.zip › Figure 1-figure supplement 1-source data 1/Figure 1-figure supplement 1-source data 1.jpg]

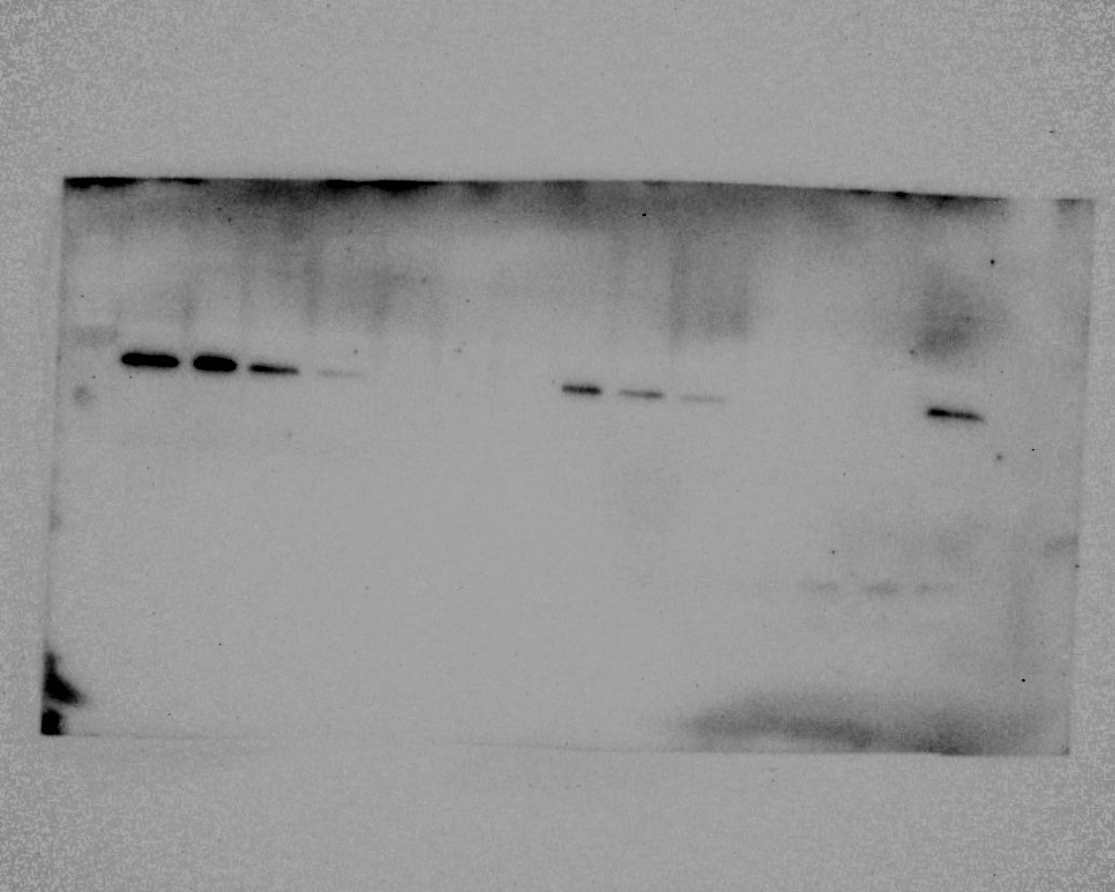

Supplement: Figure 1—figure supplement 1—source data 1. [file elife-89951-fig1-figsupp1-data1.zip › Figure 1-figure supplement 1-source data 1/IRF1_Figure 1-figure supplement 1-source data 1/Versteeg 2023-03-11 16h06m10s 30.000s(Chemiluminescence).jpg]

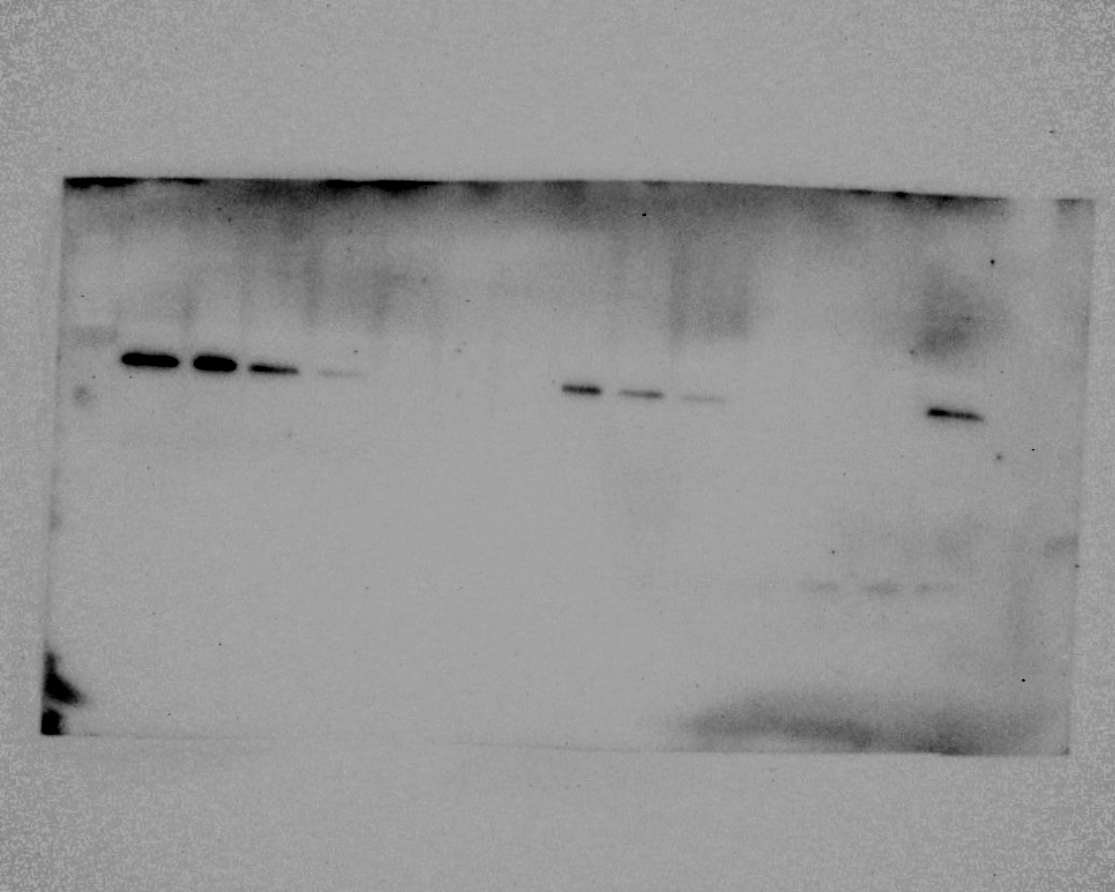

Supplement: Figure 1—figure supplement 1—source data 1. [file elife-89951-fig1-figsupp1-data1.zip › Figure 1-figure supplement 1-source data 1/IRF1_Figure 1-figure supplement 1-source data 1/Versteeg 2023-03-11 16h06m10s 30.000s(Chemiluminescence).tif]

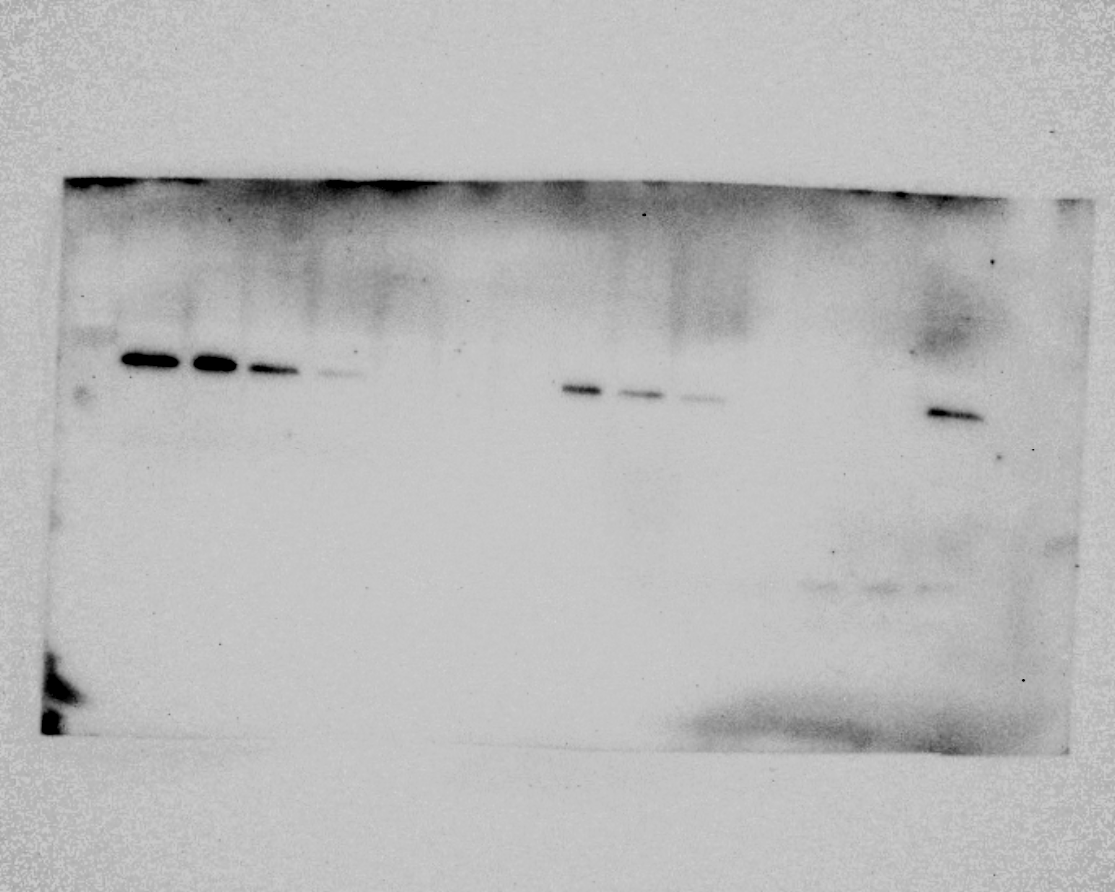

Supplement: Figure 1—figure supplement 1—source data 1. [file elife-89951-fig1-figsupp1-data1.zip › Figure 1-figure supplement 1-source data 1/IRF1_Figure 1-figure supplement 1-source data 1/Versteeg 2023-03-11 16h06m10s 30.000s(Chemiluminescence)_adj.tif]

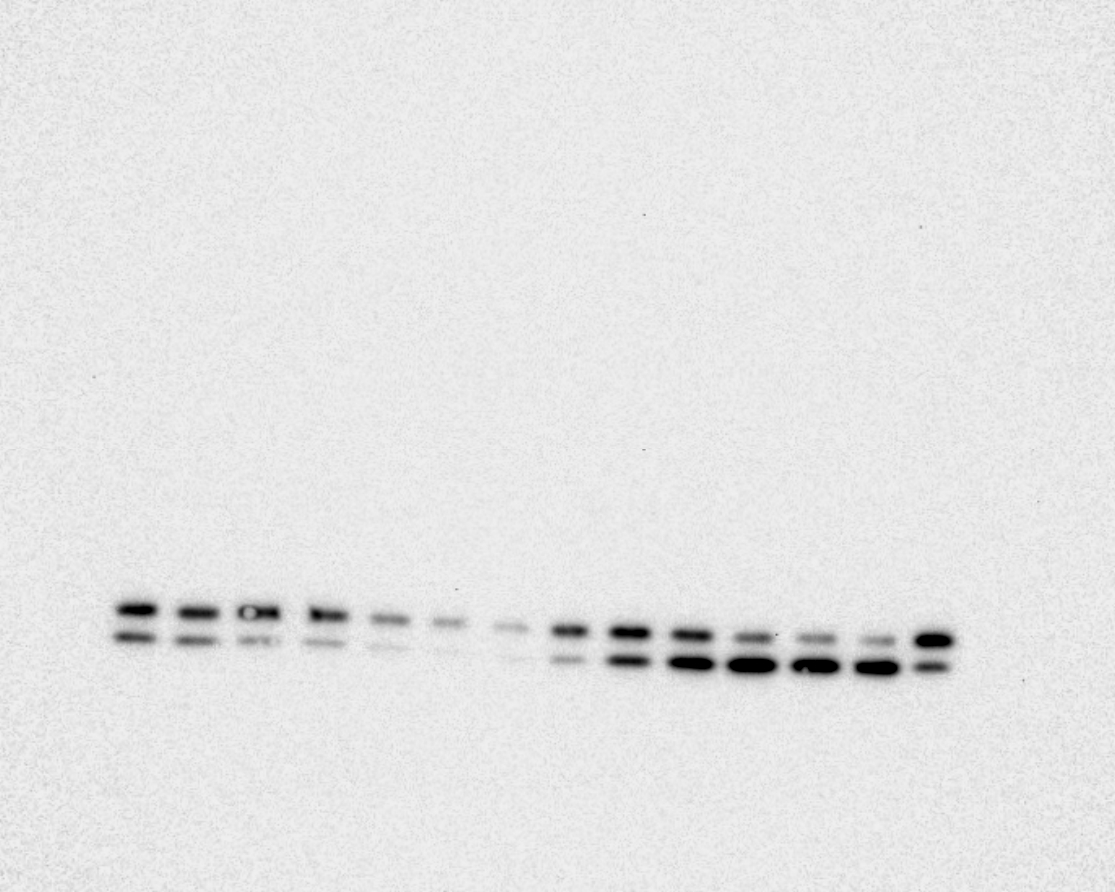

Supplement: Figure 1—figure supplement 1—source data 1. [file elife-89951-fig1-figsupp1-data1.zip › Figure 1-figure supplement 1-source data 1/LC3B_Figure 1-figure supplement 1-source data 1/Versteeg 2023-03-13 09h53m48s 123.040s(Chemiluminescence).jpg]

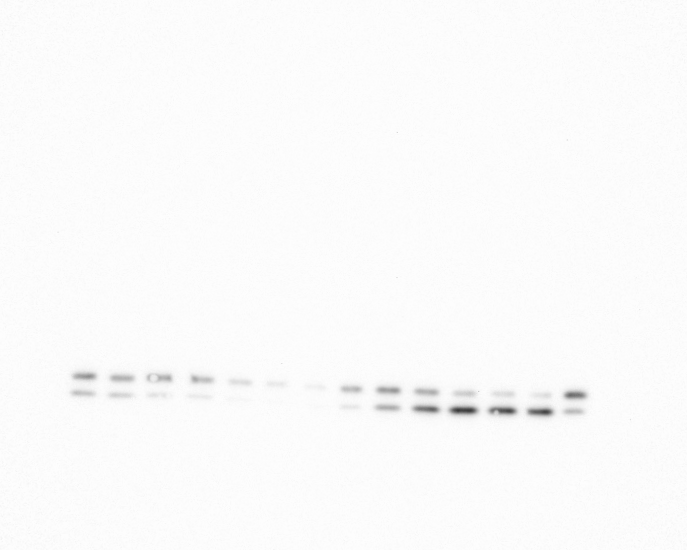

Supplement: Figure 1—figure supplement 1—source data 1. [file elife-89951-fig1-figsupp1-data1.zip › Figure 1-figure supplement 1-source data 1/LC3B_Figure 1-figure supplement 1-source data 1/Versteeg 2023-03-13 09h53m48s 123.040s(Chemiluminescence).raw16.tif]

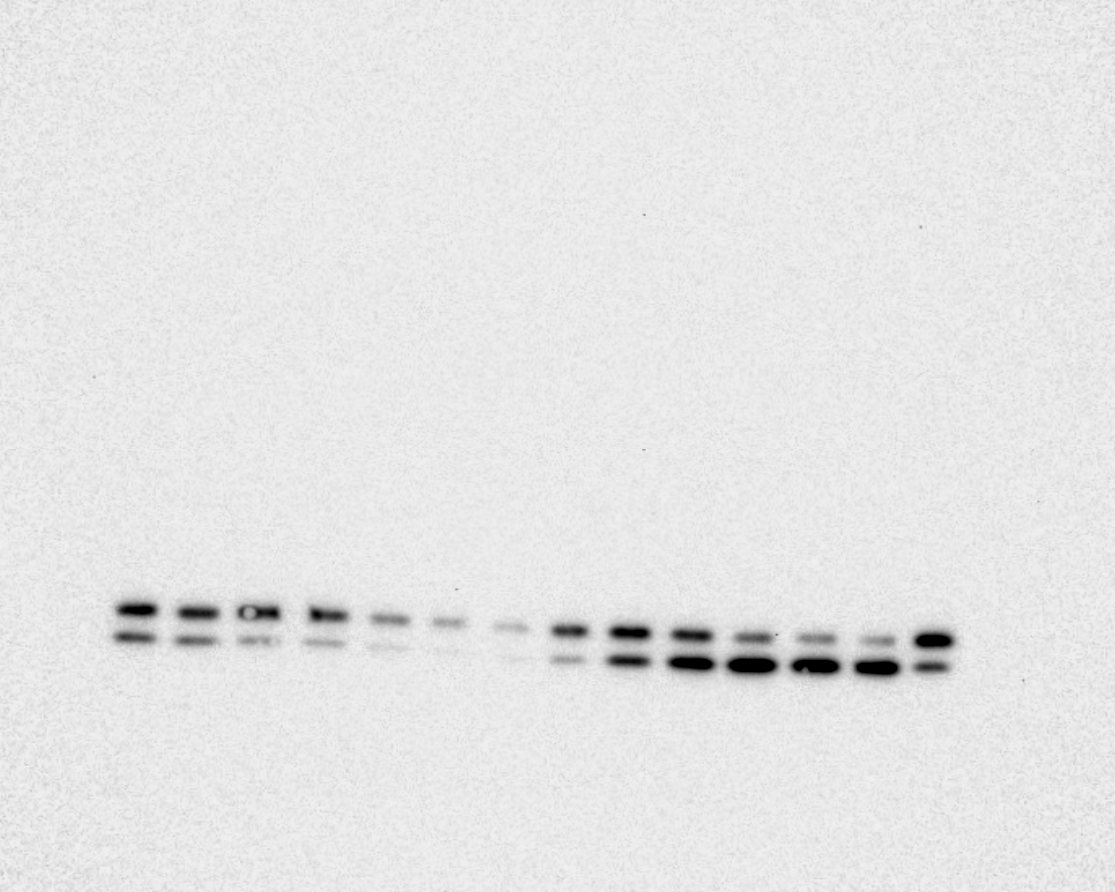

Supplement: Figure 1—figure supplement 1—source data 1. [file elife-89951-fig1-figsupp1-data1.zip › Figure 1-figure supplement 1-source data 1/LC3B_Figure 1-figure supplement 1-source data 1/Versteeg 2023-03-13 09h53m48s 123.040s(Chemiluminescence).tif]

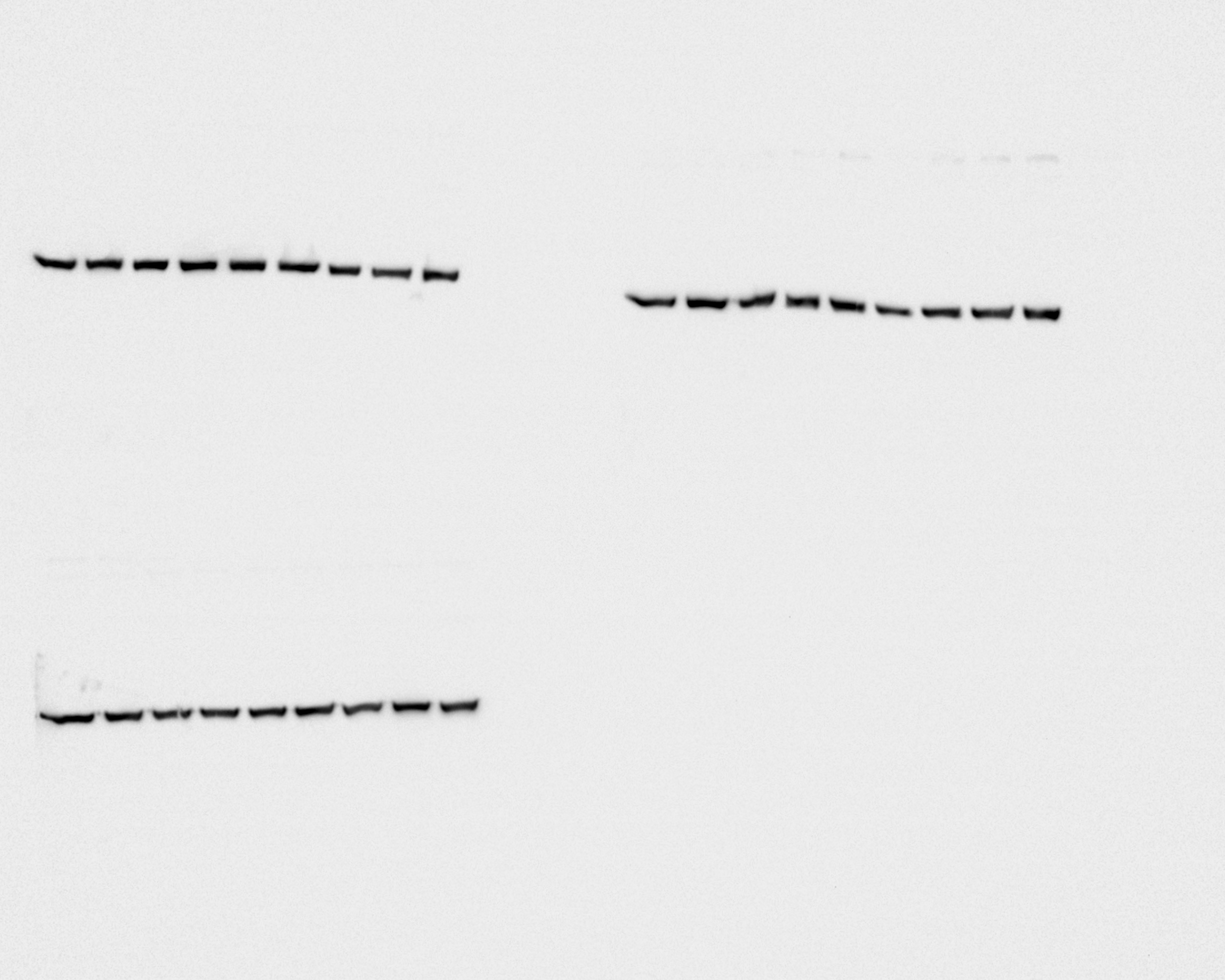

Supplement: Figure 1—figure supplement 1—source data 2. [file elife-89951-fig1-figsupp1-data2.zip › Figure 1-figure supplement 1-source data 2/ACTIN_Figure 1-figure supplement 1-source data 2/common 2019-01-30 14h11m04s 19.999s(Chemiluminescence).tif]

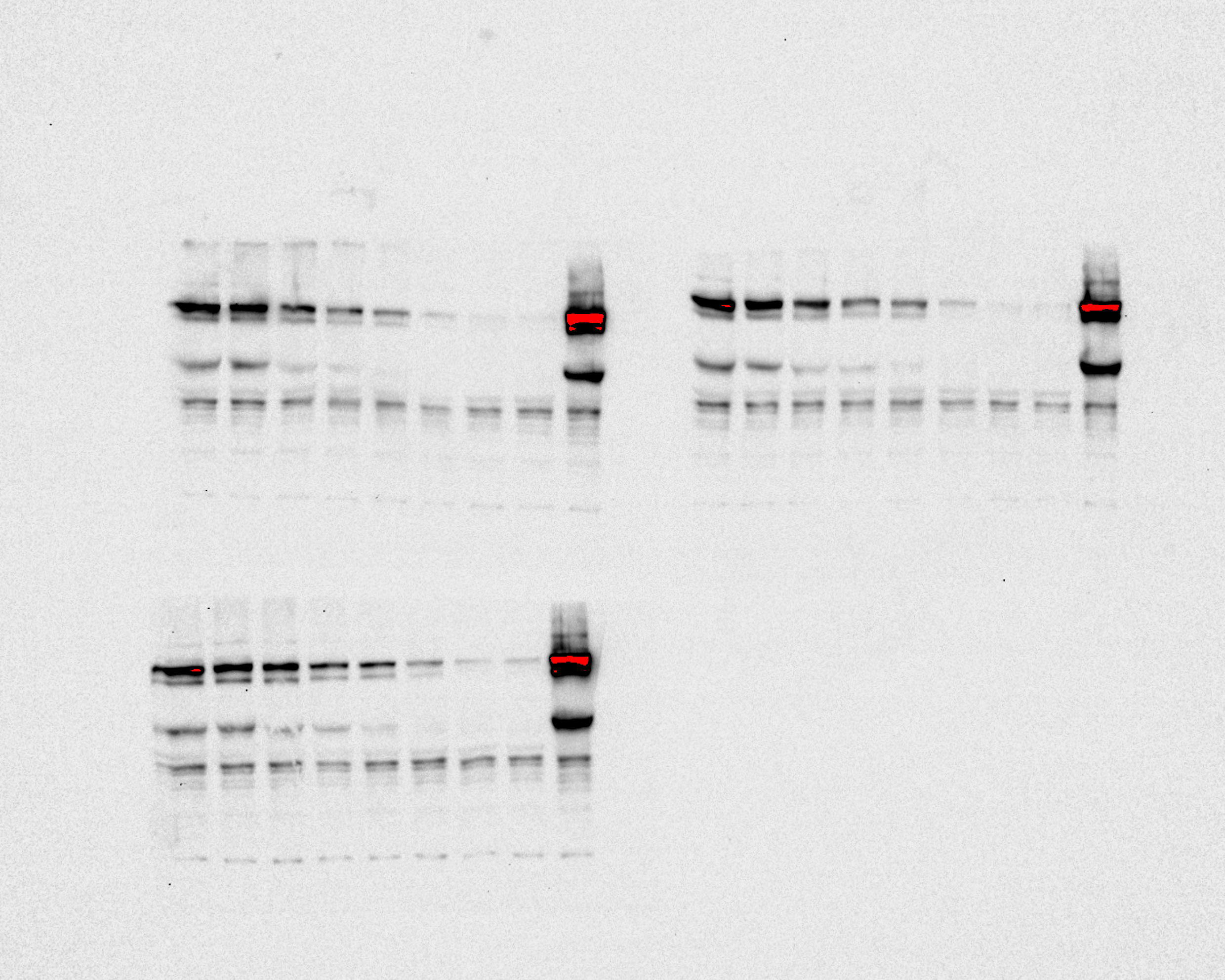

Supplement: Figure 1—figure supplement 1—source data 2. [file elife-89951-fig1-figsupp1-data2.zip › Figure 1-figure supplement 1-source data 2/cMYC_Figure 1-figure supplement 1-source data 2/Adriana 2019-02-01 11h55m25s 599.972s(Chemiluminescence).tif]

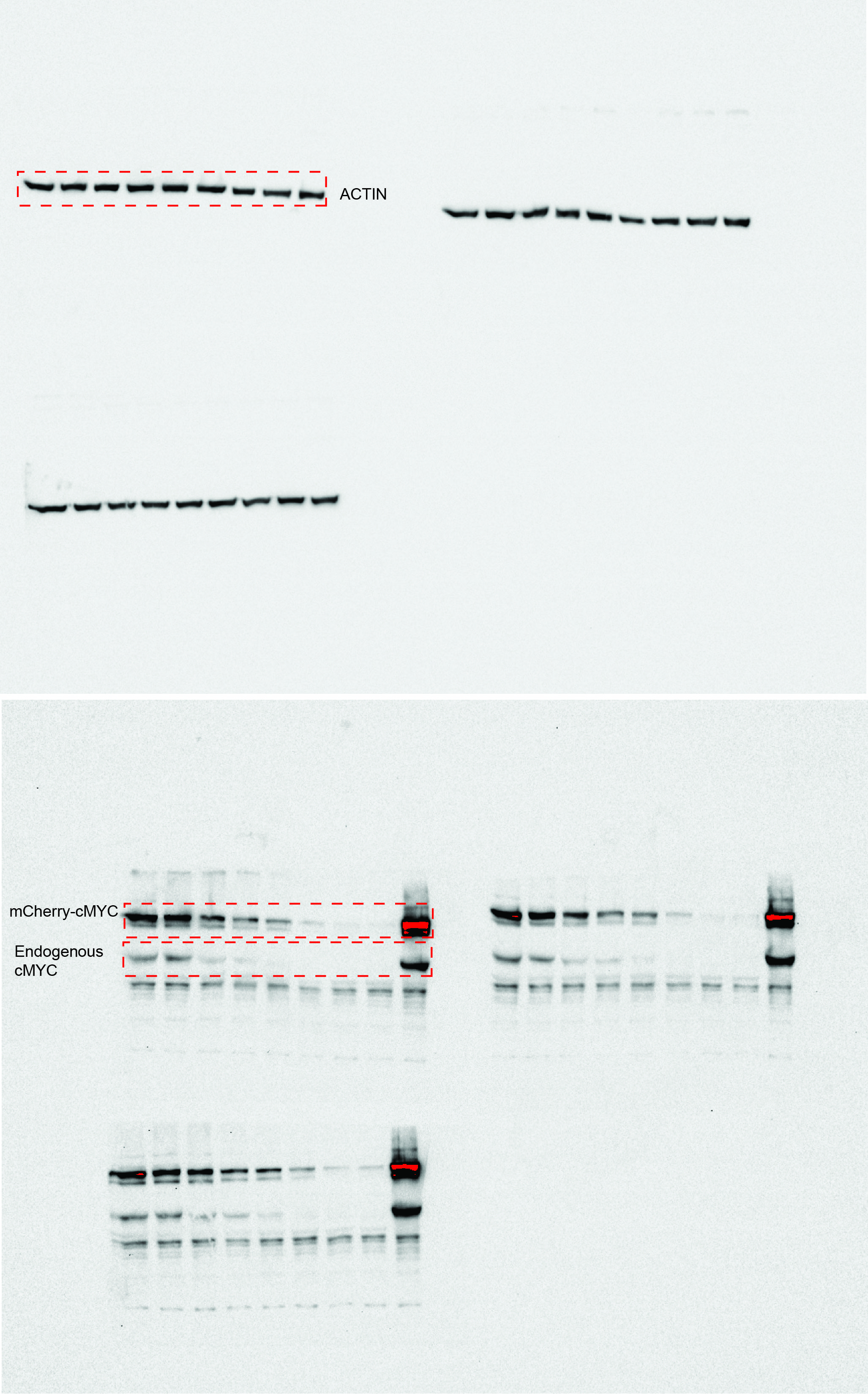

Supplement: Figure 1—figure supplement 1—source data 2. [file elife-89951-fig1-figsupp1-data2.zip › Figure 1-figure supplement 1-source data 2/Figure 1-figure supplement 1-source data 2.jpg]

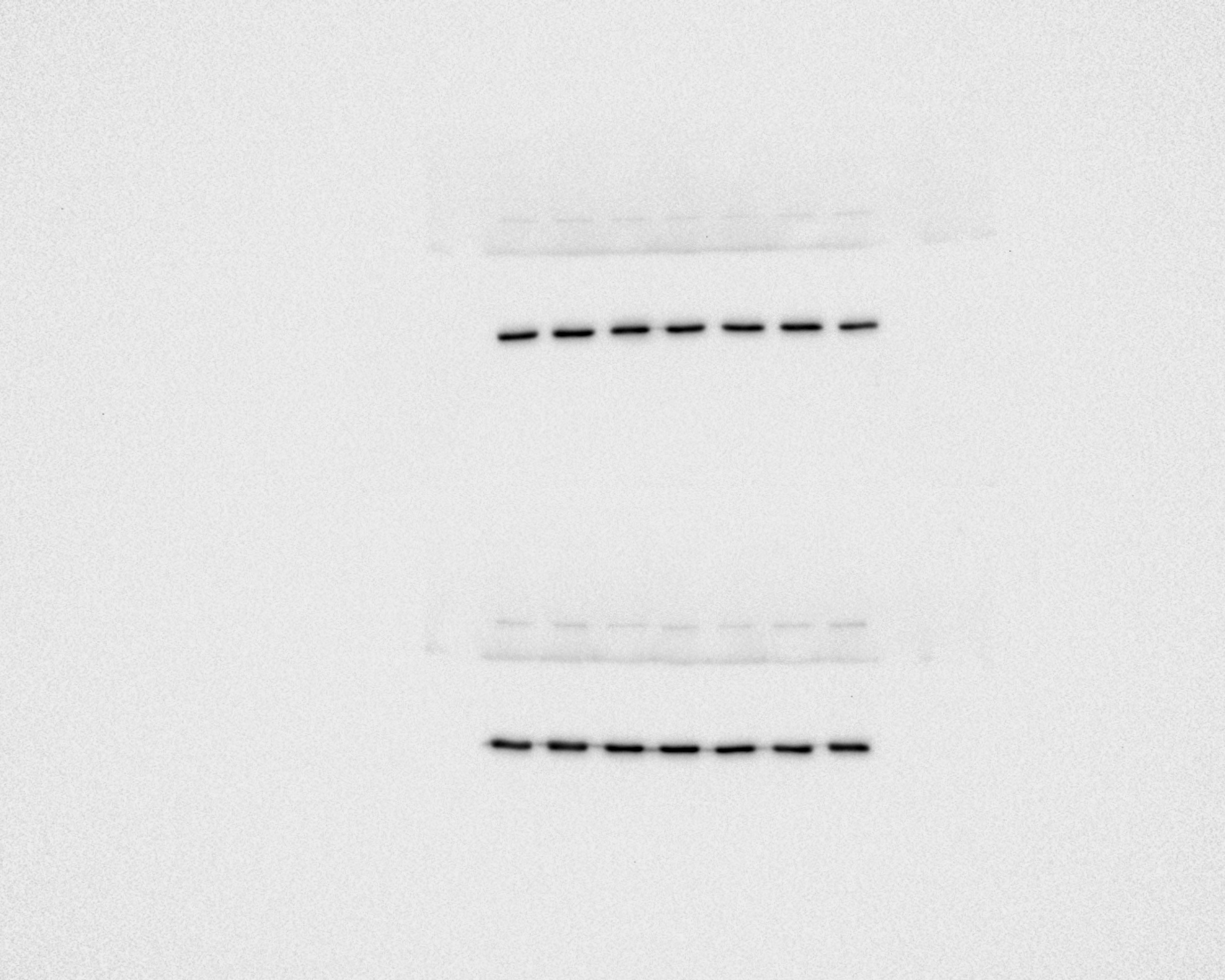

Supplement: Figure 3—source data 1. [file elife-89951-fig3-data1.zip › Figure 3-source data 1/ACTIN_Figure 3-source data 1/Versteeg 2022-03-03 12h37m09s 119.997s(Chemiluminescence).jpg]

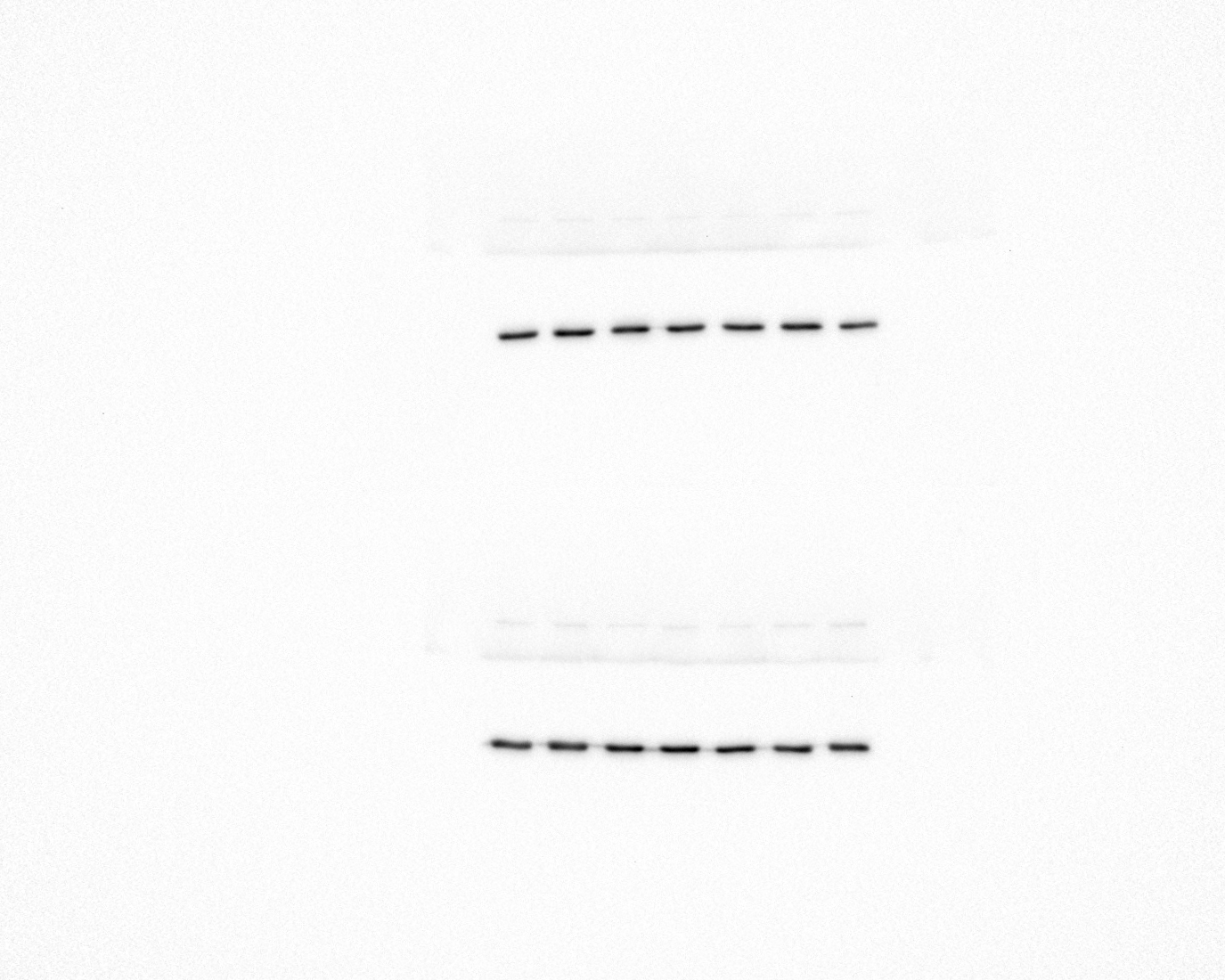

Supplement: Figure 3—source data 1. [file elife-89951-fig3-data1.zip › Figure 3-source data 1/ACTIN_Figure 3-source data 1/Versteeg 2022-03-03 12h37m09s 119.997s(Chemiluminescence).raw16.tif]

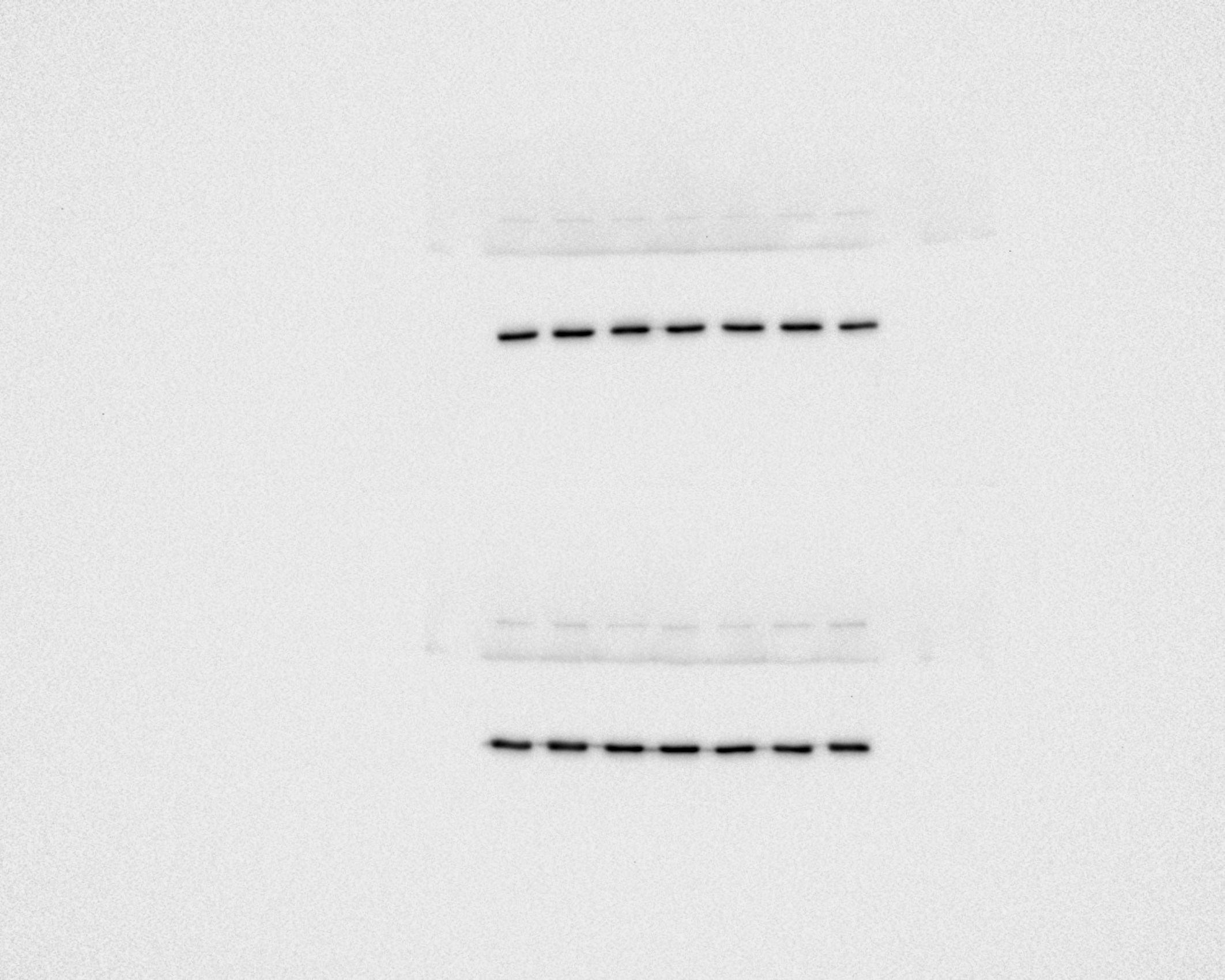

Supplement: Figure 3—source data 1. [file elife-89951-fig3-data1.zip › Figure 3-source data 1/ACTIN_Figure 3-source data 1/Versteeg 2022-03-03 12h37m09s 119.997s(Chemiluminescence).tif]

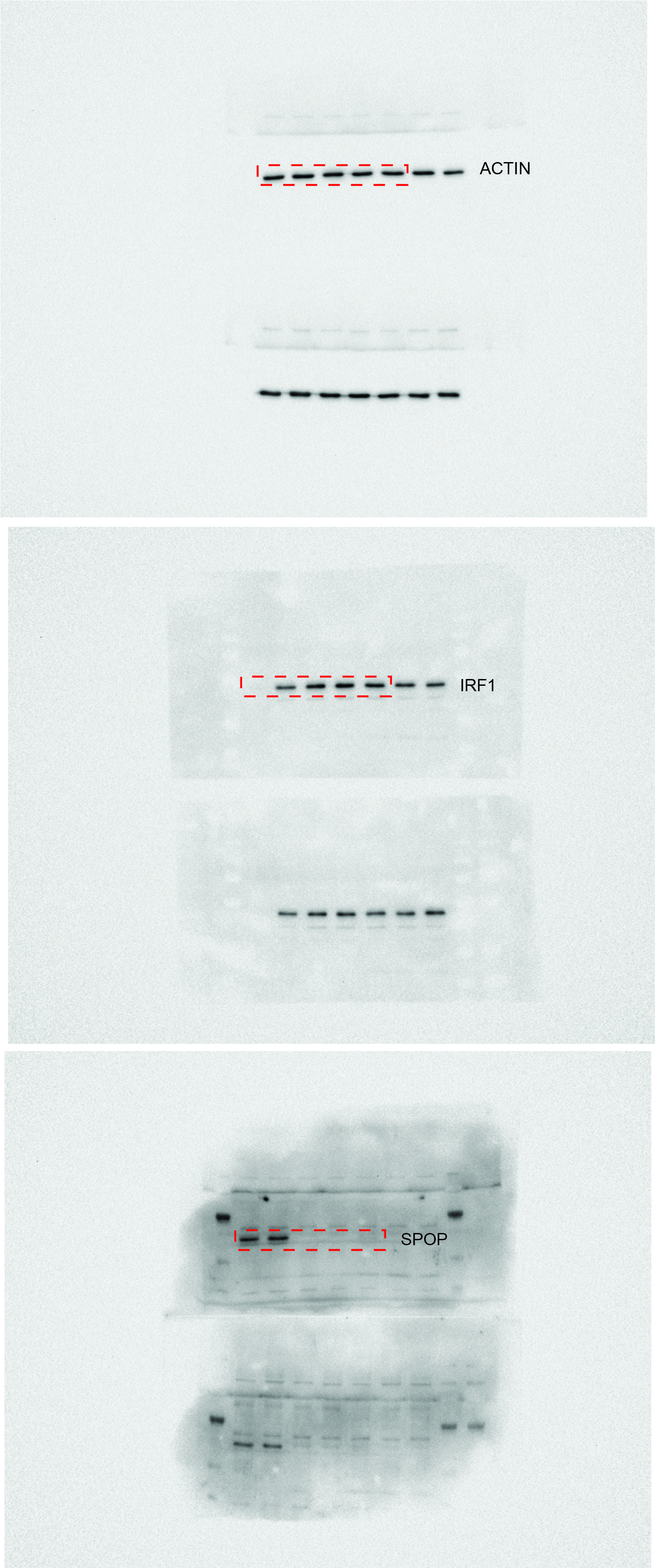

Supplement: Figure 3—source data 1. [file elife-89951-fig3-data1.zip › Figure 3-source data 1/Figure 3-source data 1.jpg]

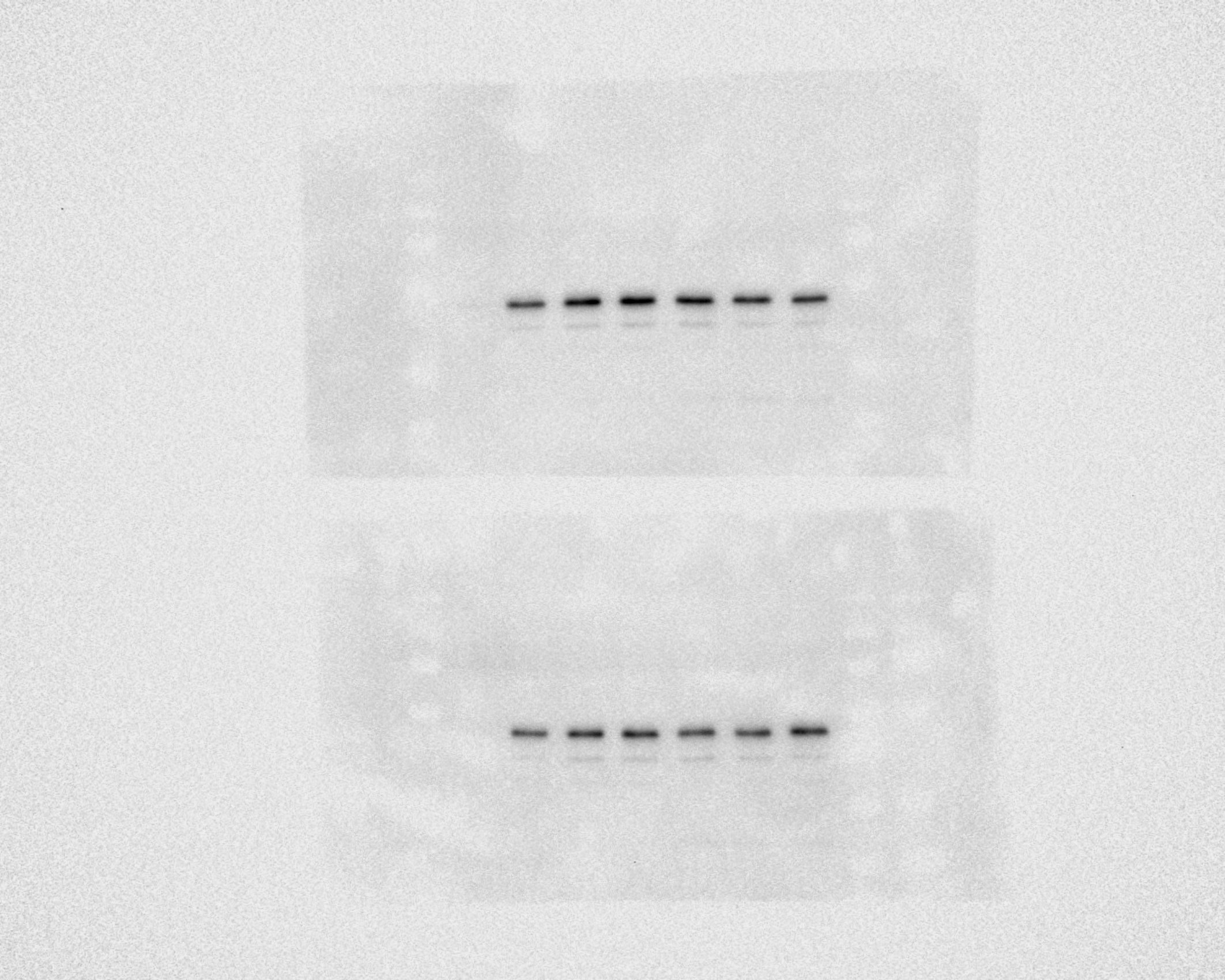

Supplement: Figure 3—source data 1. [file elife-89951-fig3-data1.zip › Figure 3-source data 1/IRF1_Figure 3-source data 1/Versteeg 2022-03-08 10h35m42s 299.990s(Chemiluminescence).jpg]

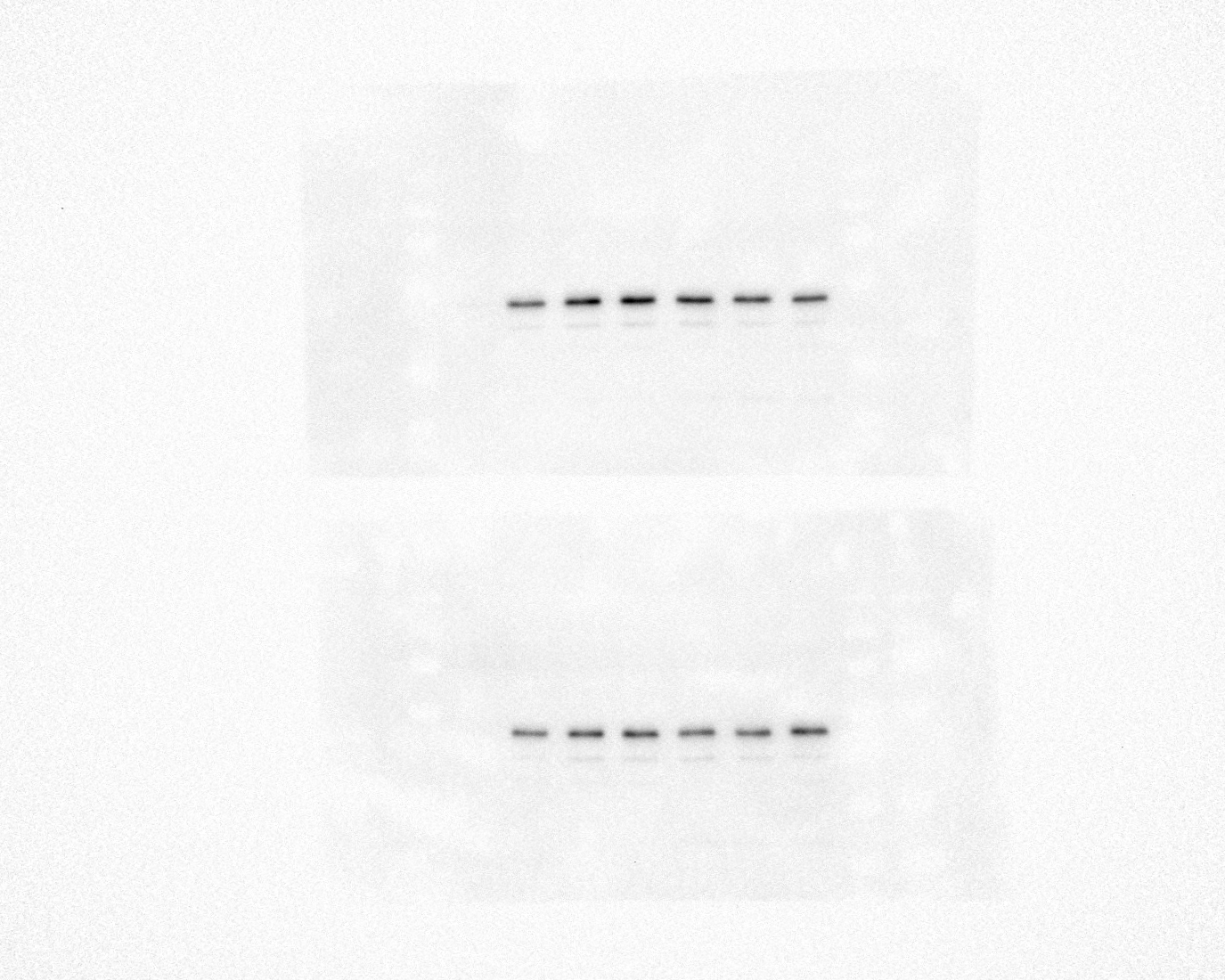

Supplement: Figure 3—source data 1. [file elife-89951-fig3-data1.zip › Figure 3-source data 1/IRF1_Figure 3-source data 1/Versteeg 2022-03-08 10h35m42s 299.990s(Chemiluminescence).raw16.tif]

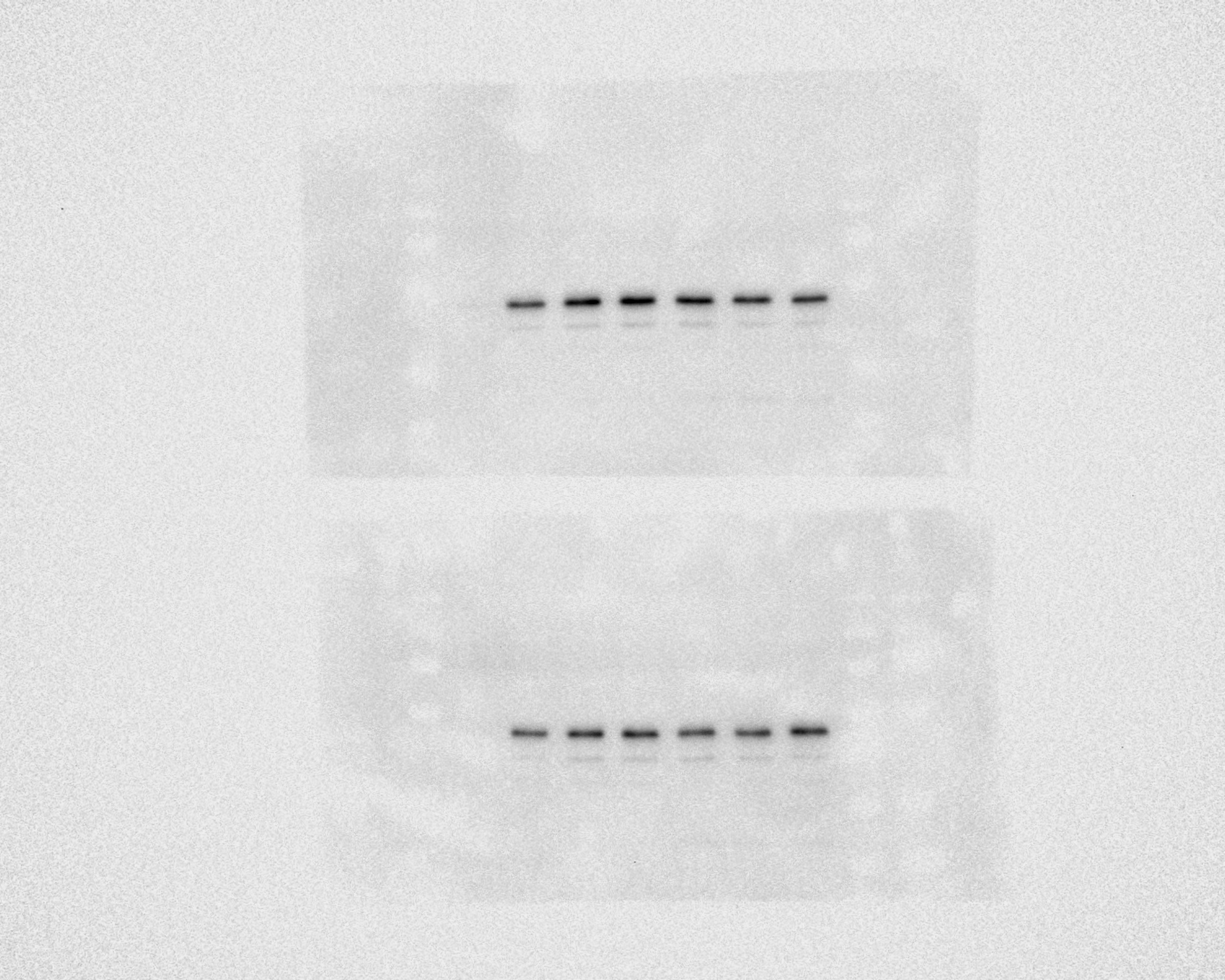

Supplement: Figure 3—source data 1. [file elife-89951-fig3-data1.zip › Figure 3-source data 1/IRF1_Figure 3-source data 1/Versteeg 2022-03-08 10h35m42s 299.990s(Chemiluminescence).tif]

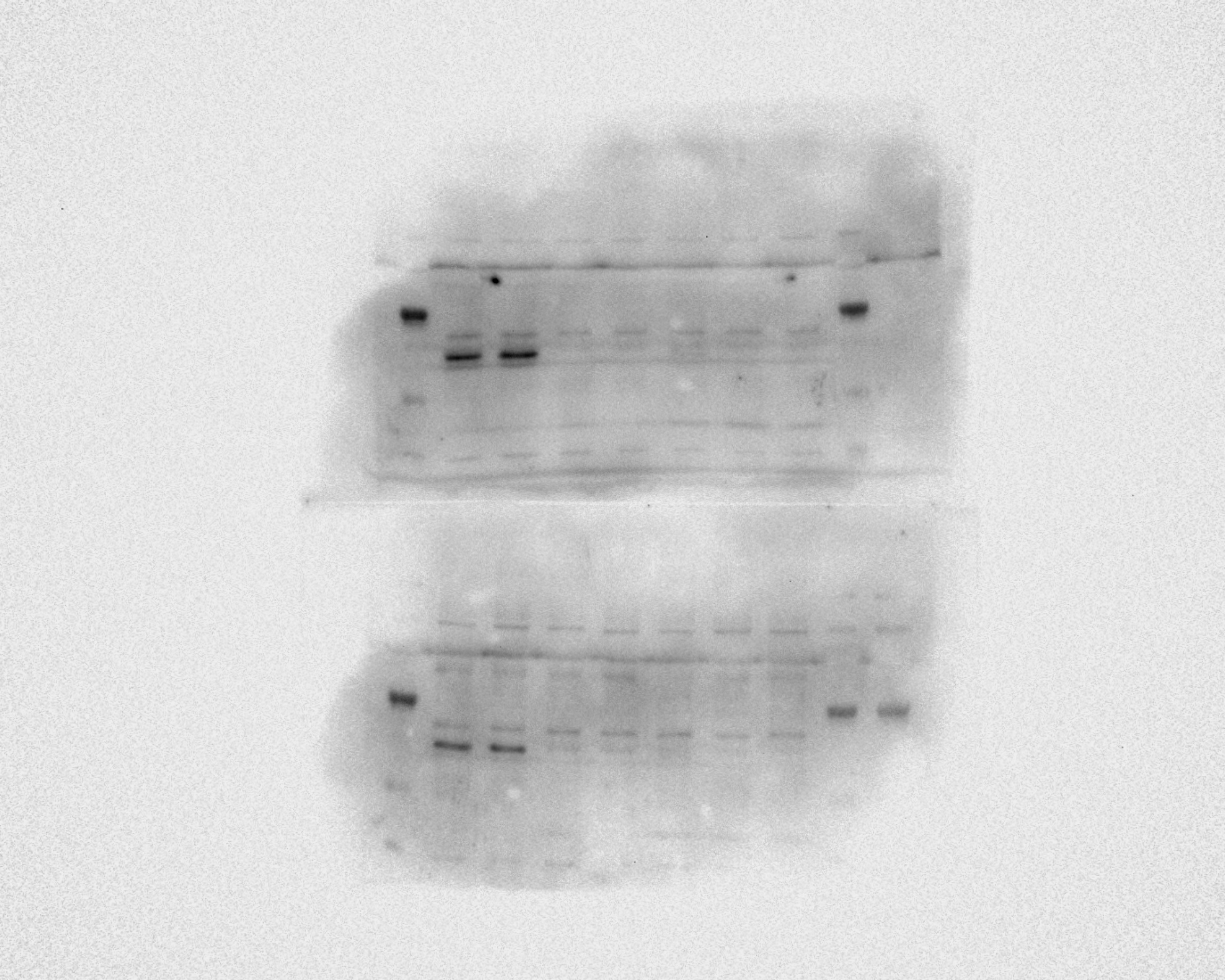

Supplement: Figure 3—source data 1. [file elife-89951-fig3-data1.zip › Figure 3-source data 1/SPOP_Figure 3-source data 1/Versteeg 2022-03-04 14h09m42s 299.990s(Chemiluminescence).jpg]

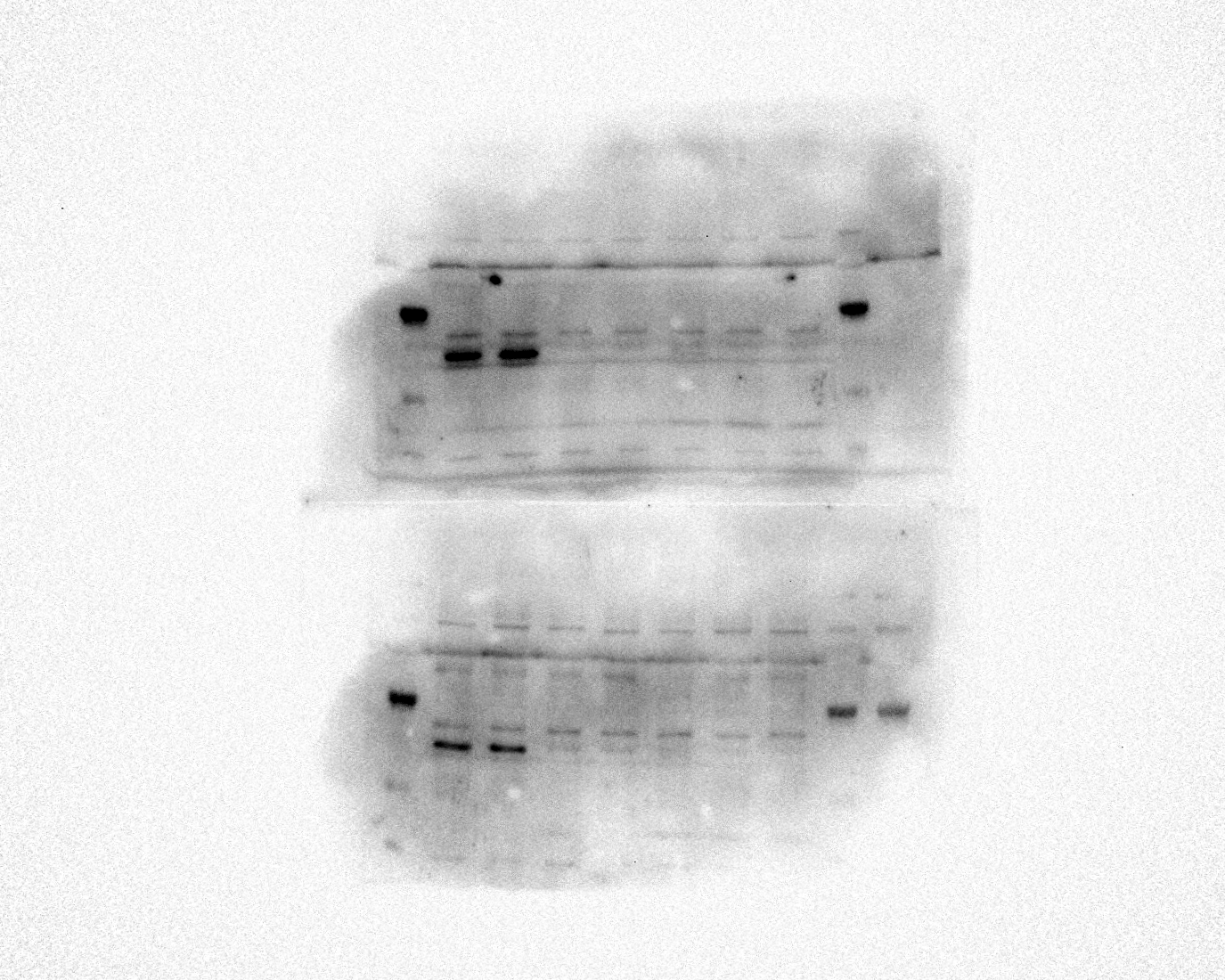

Supplement: Figure 3—source data 1. [file elife-89951-fig3-data1.zip › Figure 3-source data 1/SPOP_Figure 3-source data 1/Versteeg 2022-03-04 14h09m42s 299.990s(Chemiluminescence).raw16.tif]

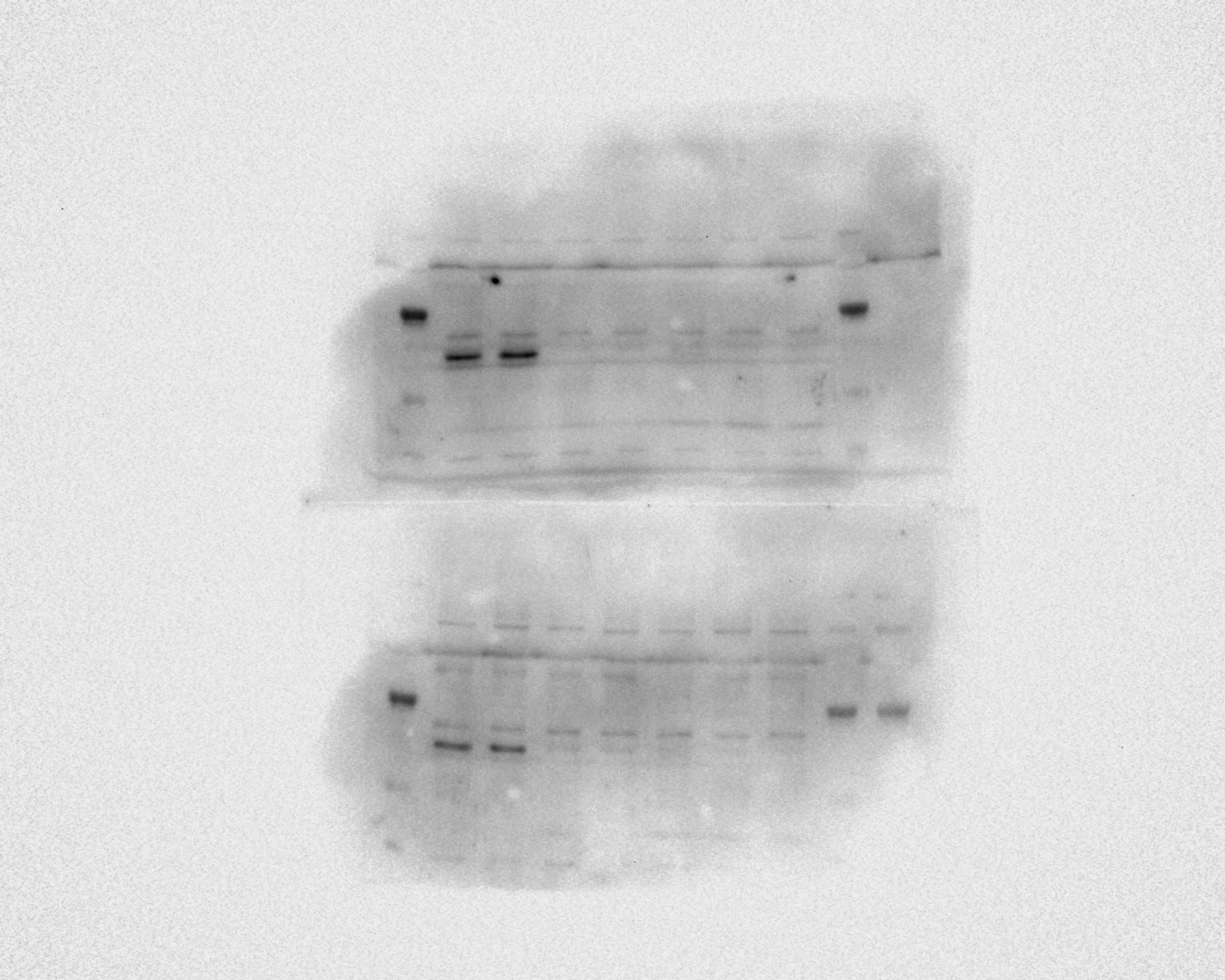

Supplement: Figure 3—source data 1. [file elife-89951-fig3-data1.zip › Figure 3-source data 1/SPOP_Figure 3-source data 1/Versteeg 2022-03-04 14h09m42s 299.990s(Chemiluminescence).tif]

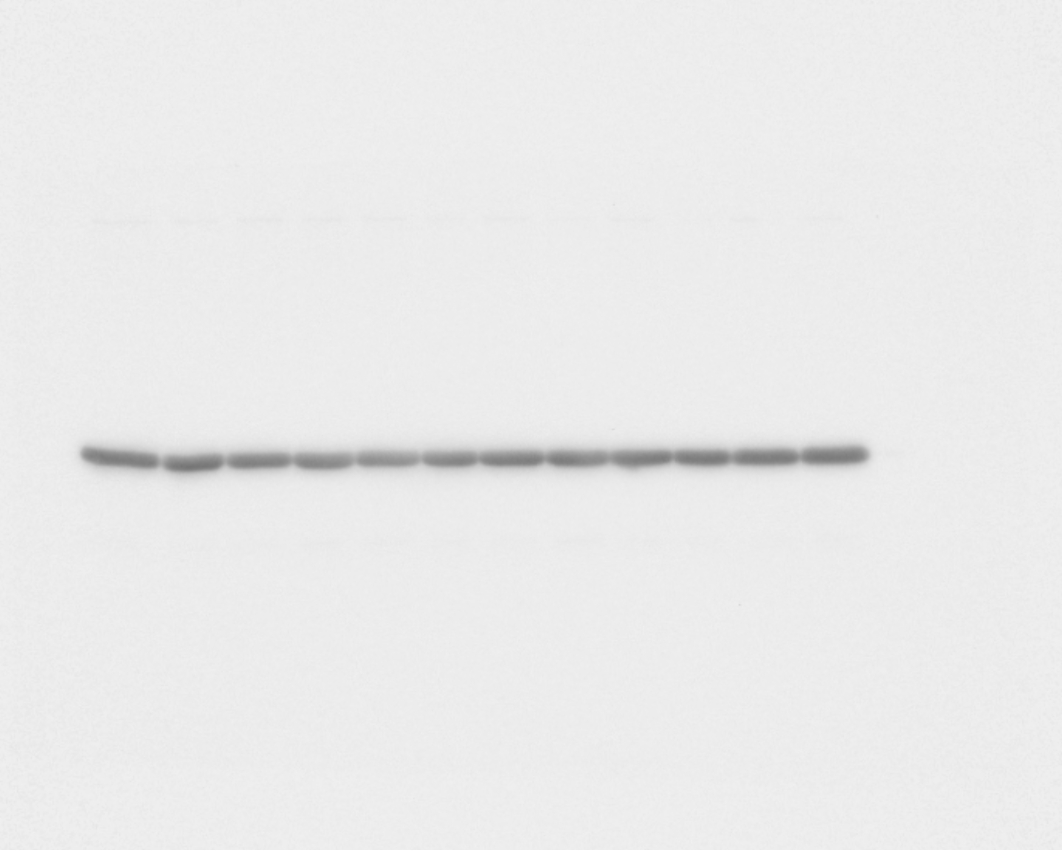

Supplement: Figure 3—source data 2. [file elife-89951-fig3-data2.zip › Figure 3-source data 2/ACTIN_Figure 3-source data 2/Versteeg 2023-04-06 10h56m31s 13.204s(Chemiluminescence).jpg]

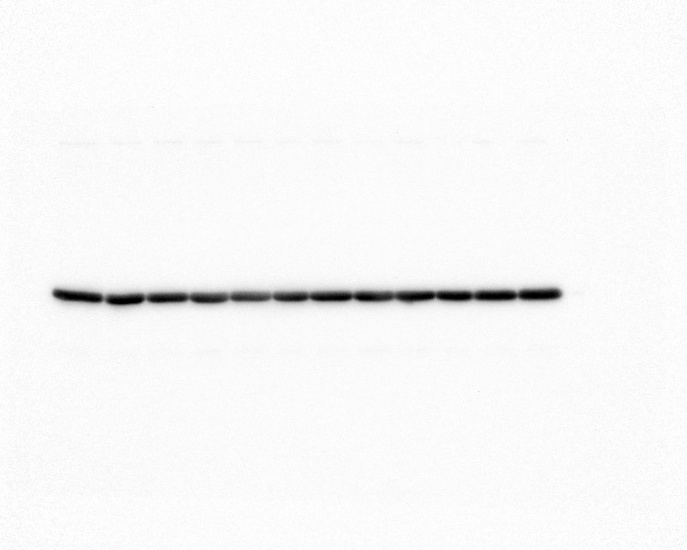

Supplement: Figure 3—source data 2. [file elife-89951-fig3-data2.zip › Figure 3-source data 2/ACTIN_Figure 3-source data 2/Versteeg 2023-04-06 10h56m31s 13.204s(Chemiluminescence).raw16.tif]

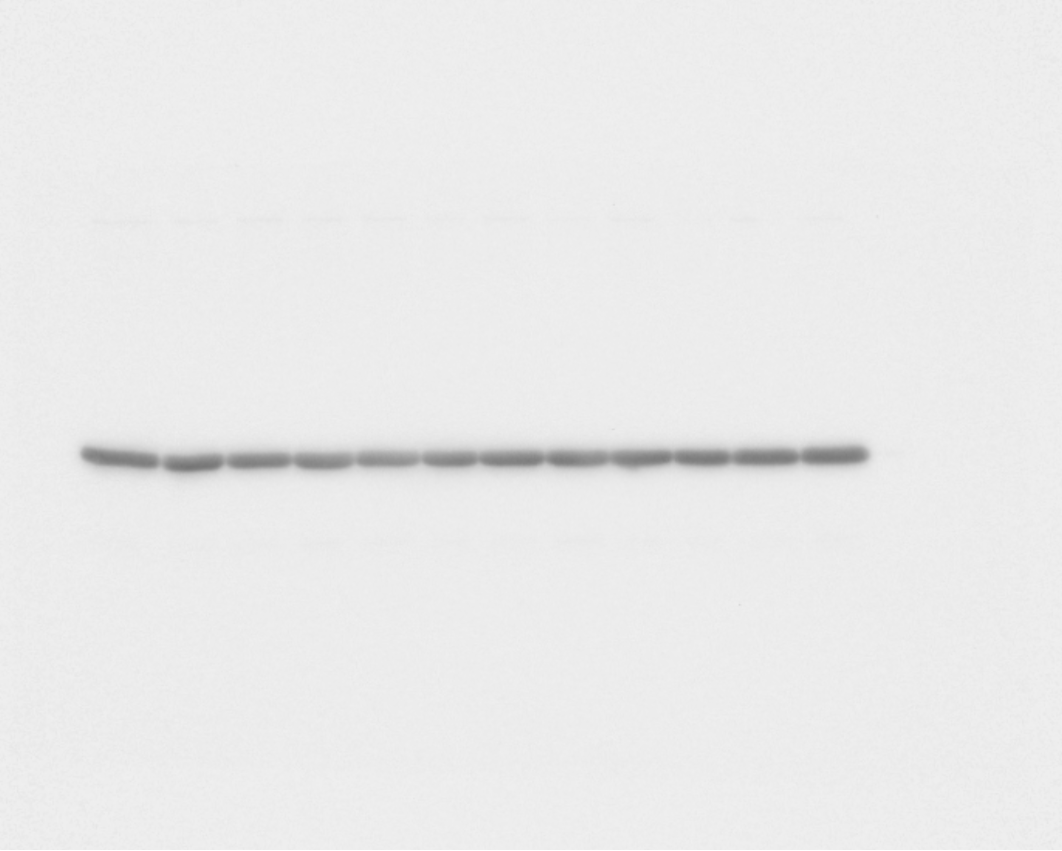

Supplement: Figure 3—source data 2. [file elife-89951-fig3-data2.zip › Figure 3-source data 2/ACTIN_Figure 3-source data 2/Versteeg 2023-04-06 10h56m31s 13.204s(Chemiluminescence).tif]

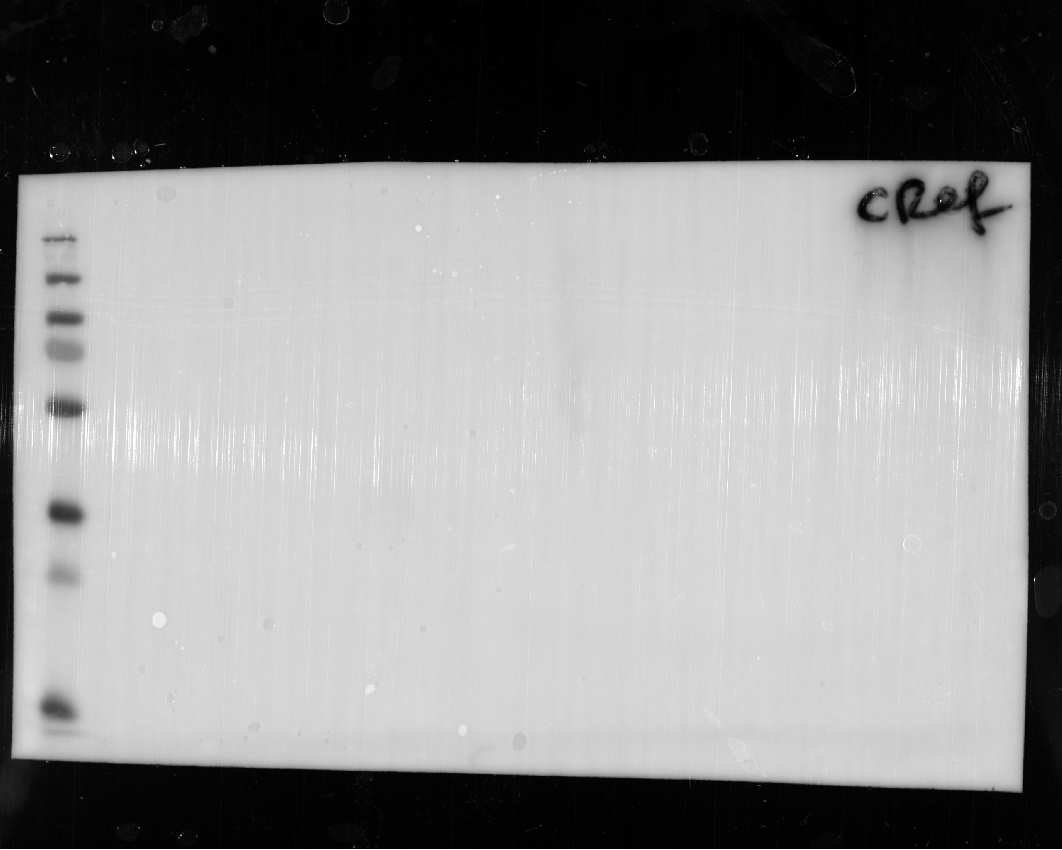

Supplement: Figure 3—source data 2. [file elife-89951-fig3-data2.zip › Figure 3-source data 2/ACTIN_Figure 3-source data 2/Versteeg 2023-04-06 10h58m19s 0.671s(Colorimetric).jpg]

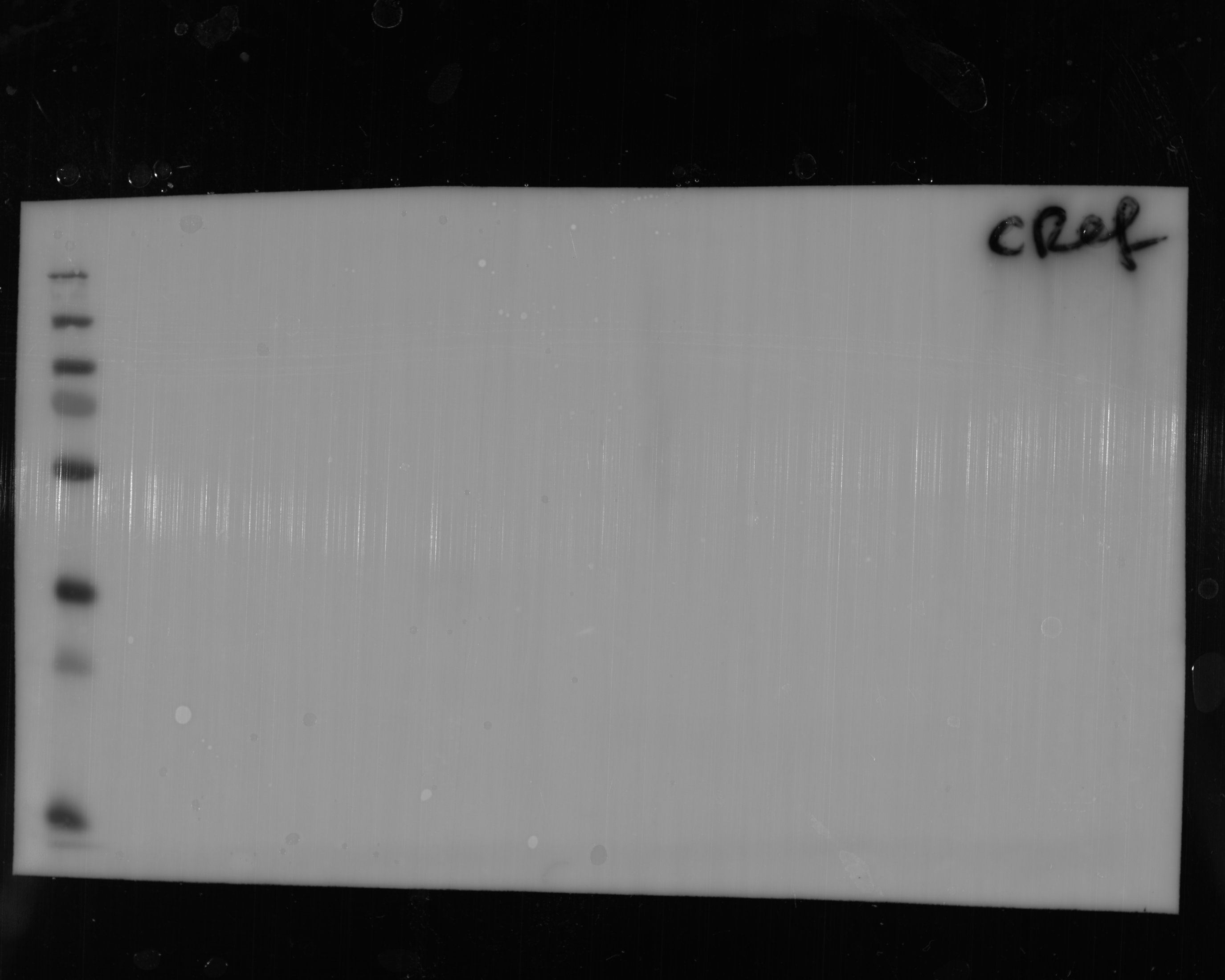

Supplement: Figure 3—source data 2. [file elife-89951-fig3-data2.zip › Figure 3-source data 2/ACTIN_Figure 3-source data 2/Versteeg 2023-04-06 10h58m19s 0.671s(Colorimetric).raw16.tif]

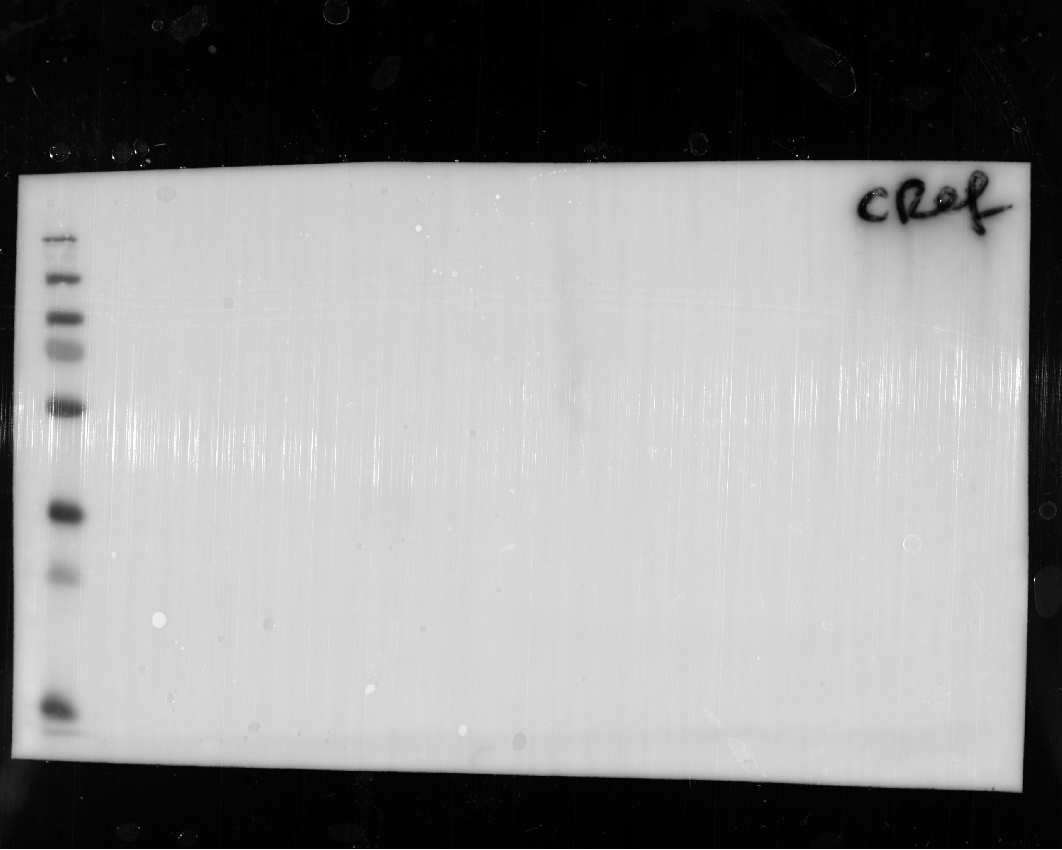

Supplement: Figure 3—source data 2. [file elife-89951-fig3-data2.zip › Figure 3-source data 2/ACTIN_Figure 3-source data 2/Versteeg 2023-04-06 10h58m19s 0.671s(Colorimetric).tif]

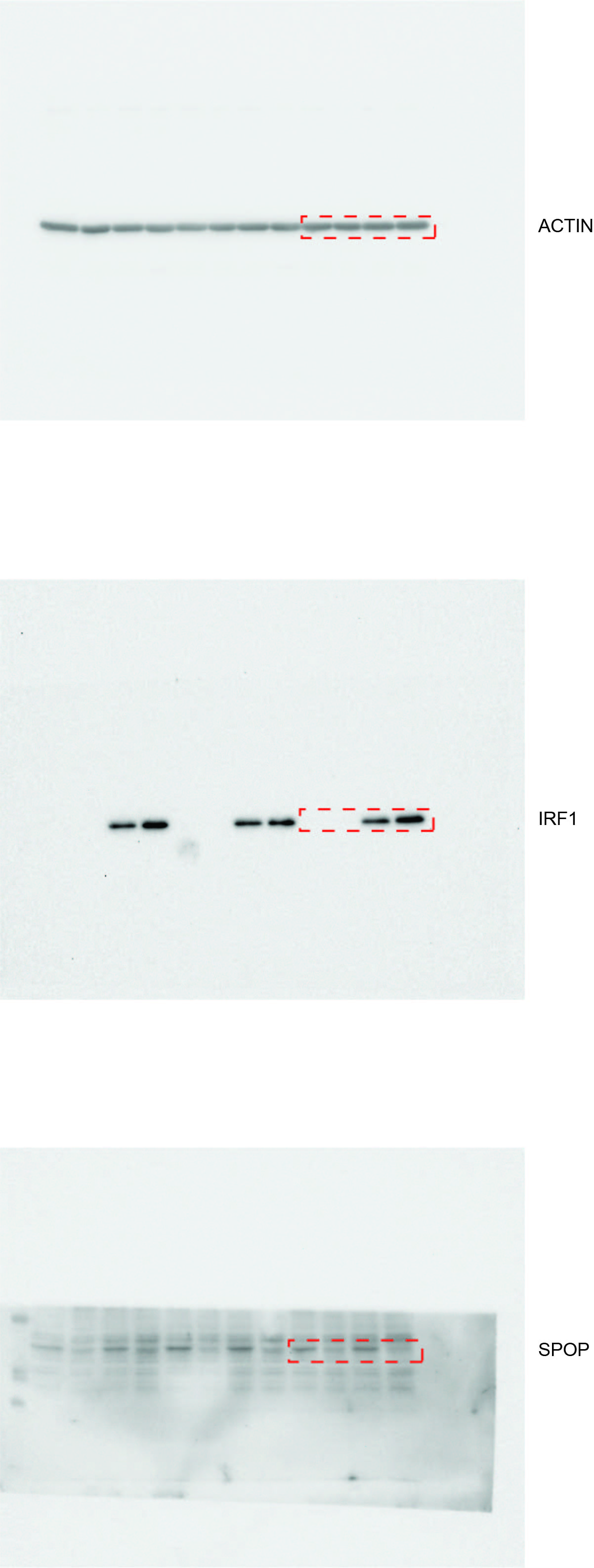

Supplement: Figure 3—source data 2. [file elife-89951-fig3-data2.zip › Figure 3-source data 2/Figure 3-source data 2.jpg]

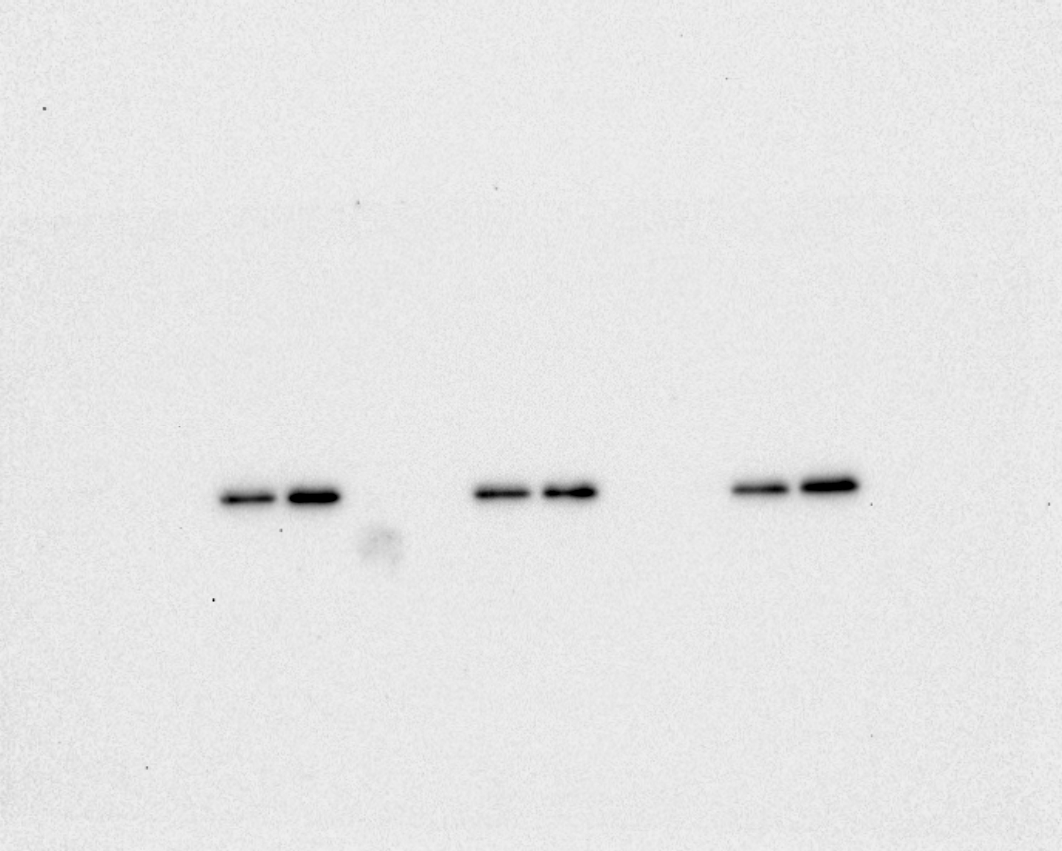

Supplement: Figure 3—source data 2. [file elife-89951-fig3-data2.zip › Figure 3-source data 2/IRF1_Figure 3-source data 2/valentina 2023-04-05 11h16m10s(Chemiluminescence).jpg]

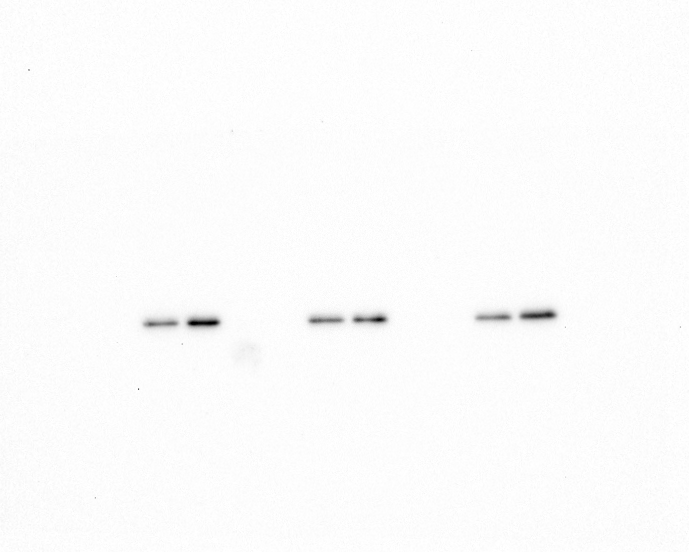

Supplement: Figure 3—source data 2. [file elife-89951-fig3-data2.zip › Figure 3-source data 2/IRF1_Figure 3-source data 2/valentina 2023-04-05 11h16m10s(Chemiluminescence).raw16.tif]

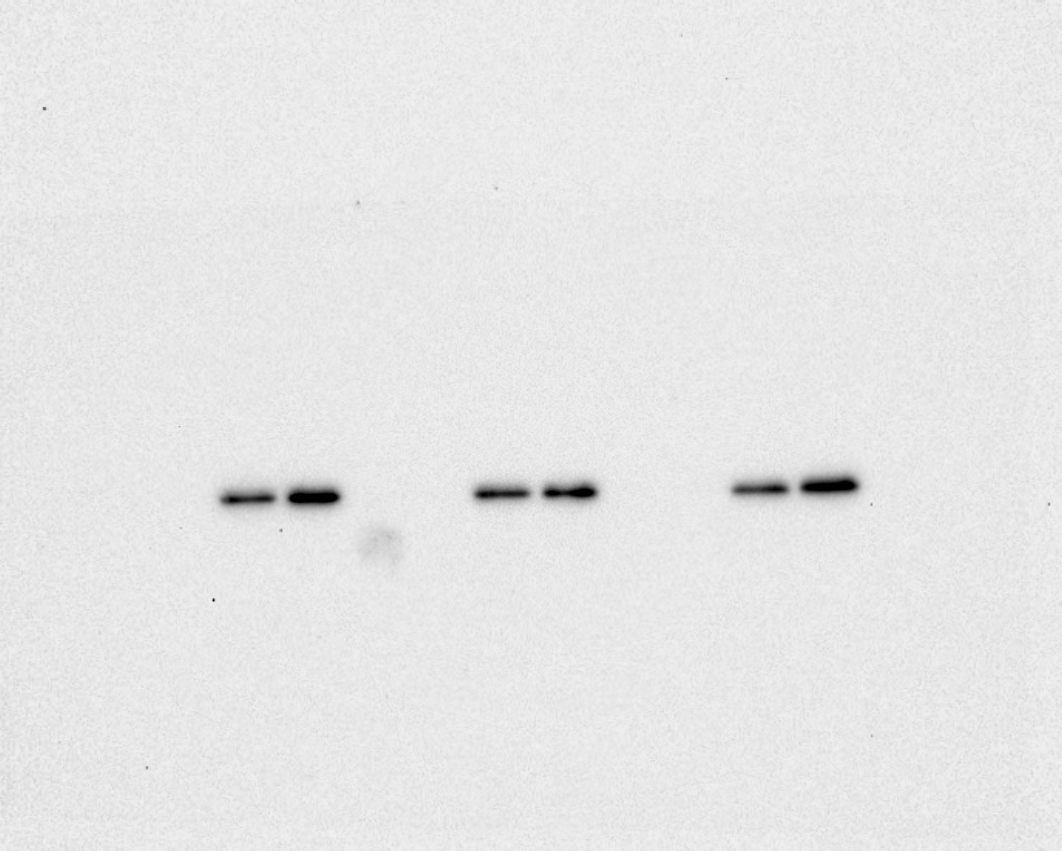

Supplement: Figure 3—source data 2. [file elife-89951-fig3-data2.zip › Figure 3-source data 2/IRF1_Figure 3-source data 2/valentina 2023-04-05 11h16m10s(Chemiluminescence).tif]

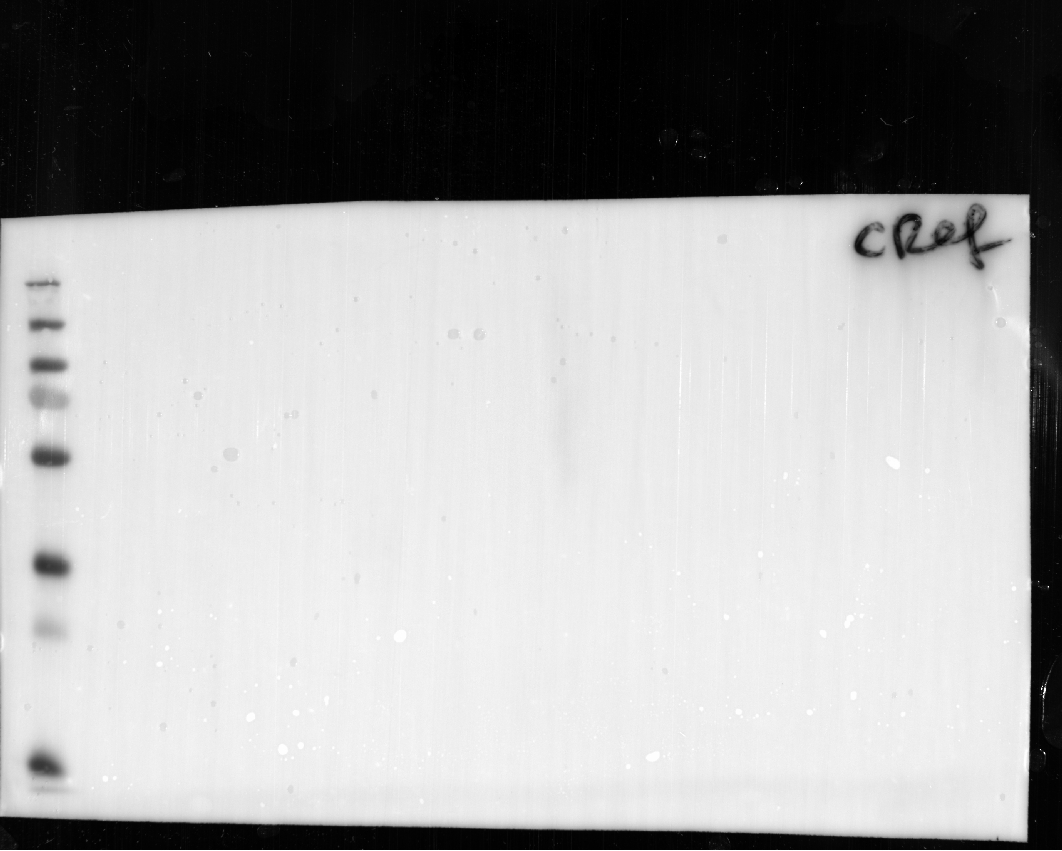

Supplement: Figure 3—source data 2. [file elife-89951-fig3-data2.zip › Figure 3-source data 2/IRF1_Figure 3-source data 2/valentina 2023-04-05 11h17m53s(Colorimetric).jpg]

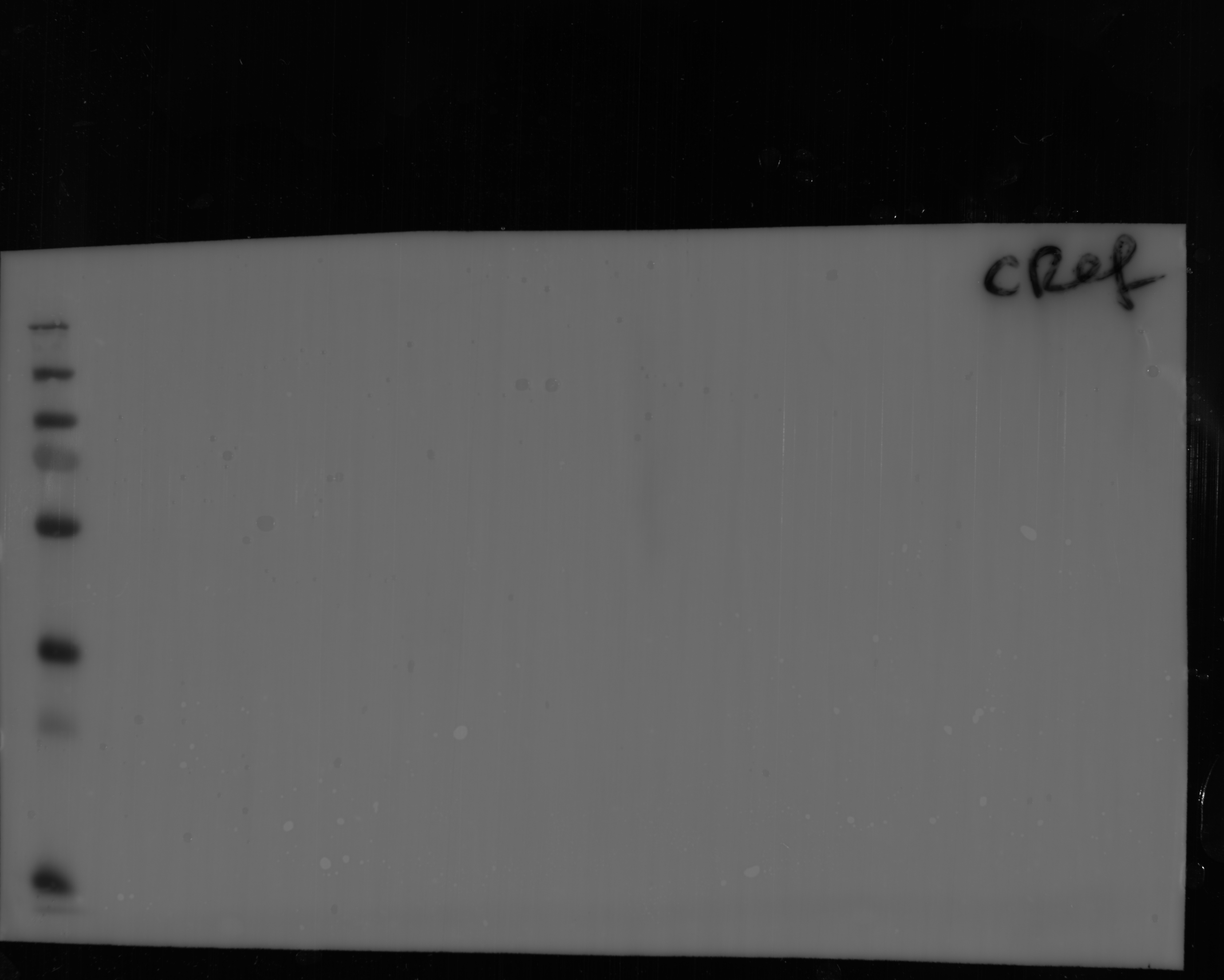

Supplement: Figure 3—source data 2. [file elife-89951-fig3-data2.zip › Figure 3-source data 2/IRF1_Figure 3-source data 2/valentina 2023-04-05 11h17m53s(Colorimetric).raw16.tif]

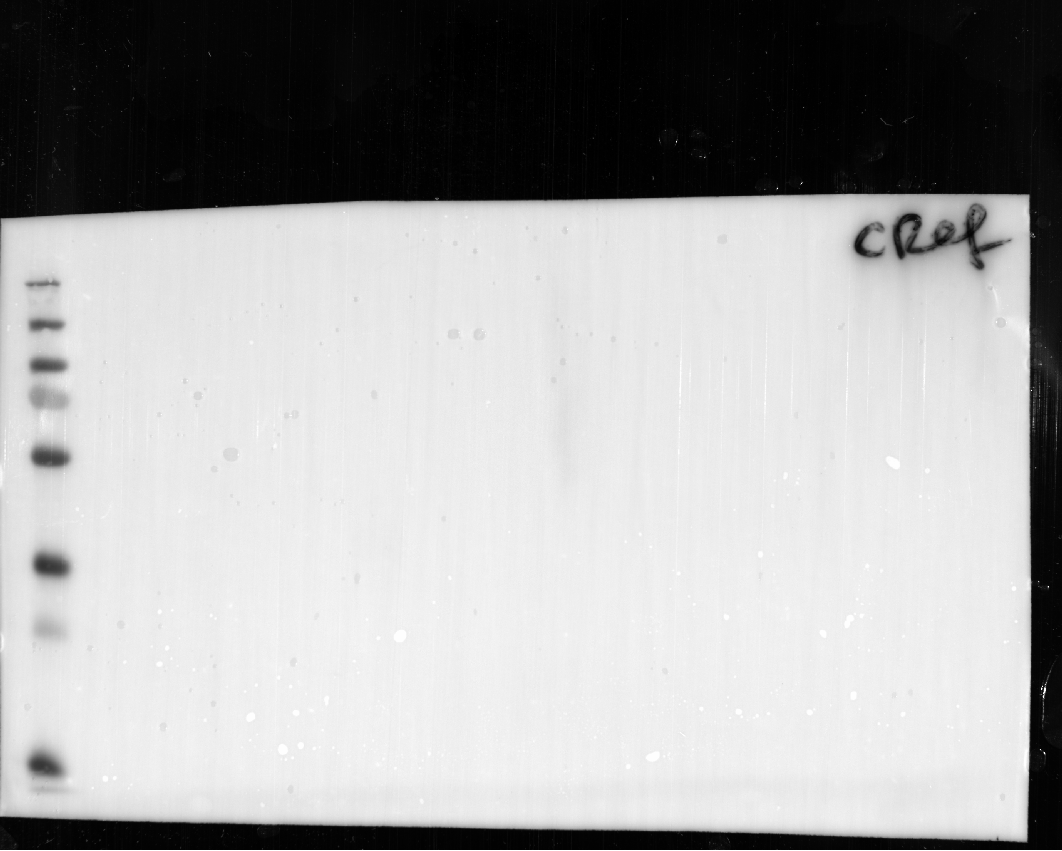

Supplement: Figure 3—source data 2. [file elife-89951-fig3-data2.zip › Figure 3-source data 2/IRF1_Figure 3-source data 2/valentina 2023-04-05 11h17m53s(Colorimetric).tif]

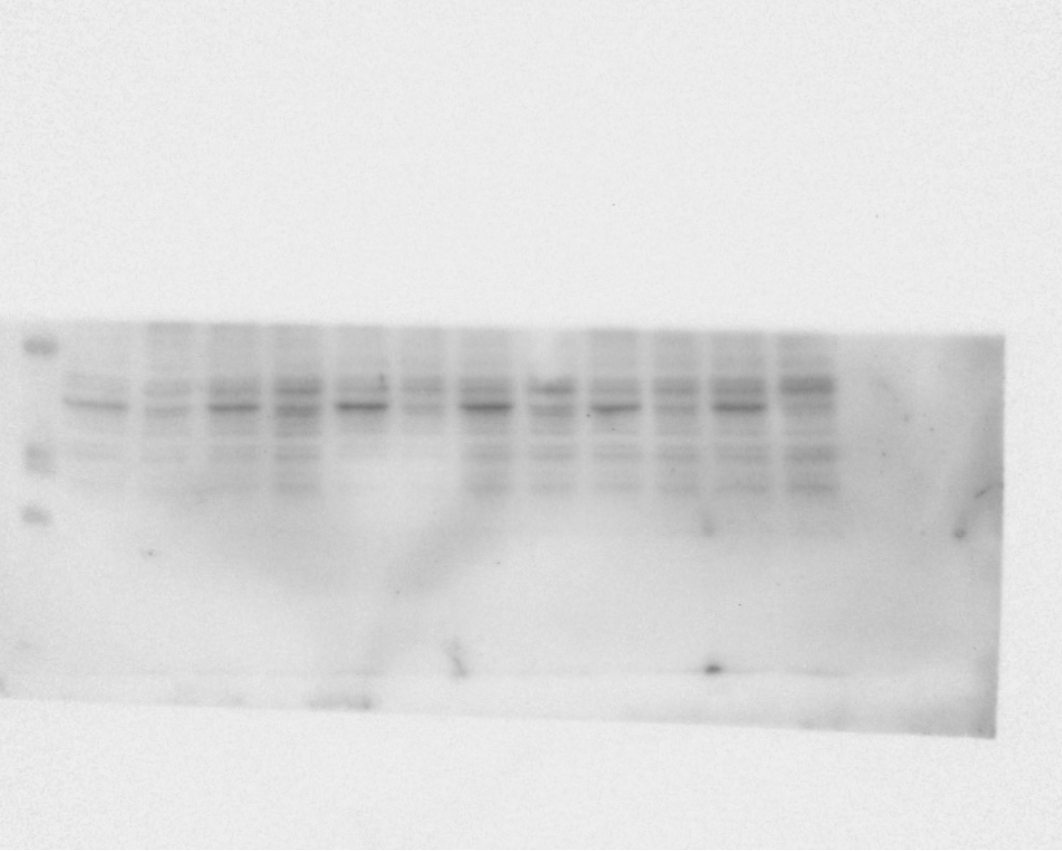

Supplement: Figure 3—source data 2. [file elife-89951-fig3-data2.zip › Figure 3-source data 2/SPOP_Figure 3-source data 2/Versteeg 2023-04-06 08h26m56s 37.612s(Chemiluminescence).jpg]

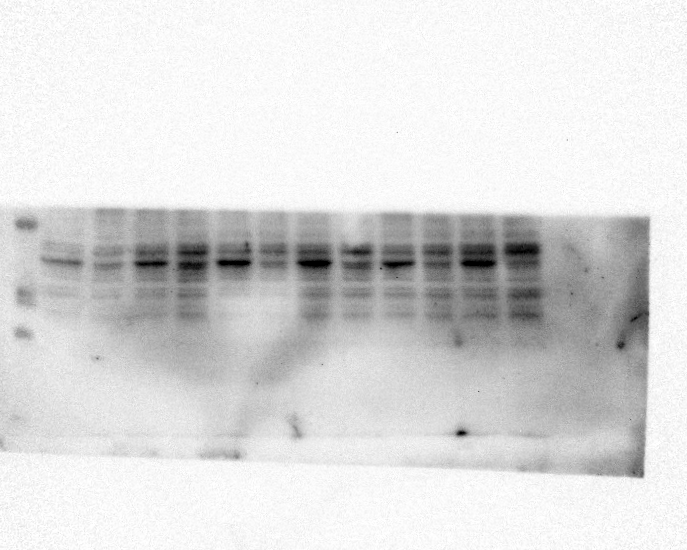

Supplement: Figure 3—source data 2. [file elife-89951-fig3-data2.zip › Figure 3-source data 2/SPOP_Figure 3-source data 2/Versteeg 2023-04-06 08h26m56s 37.612s(Chemiluminescence).raw16.tif]

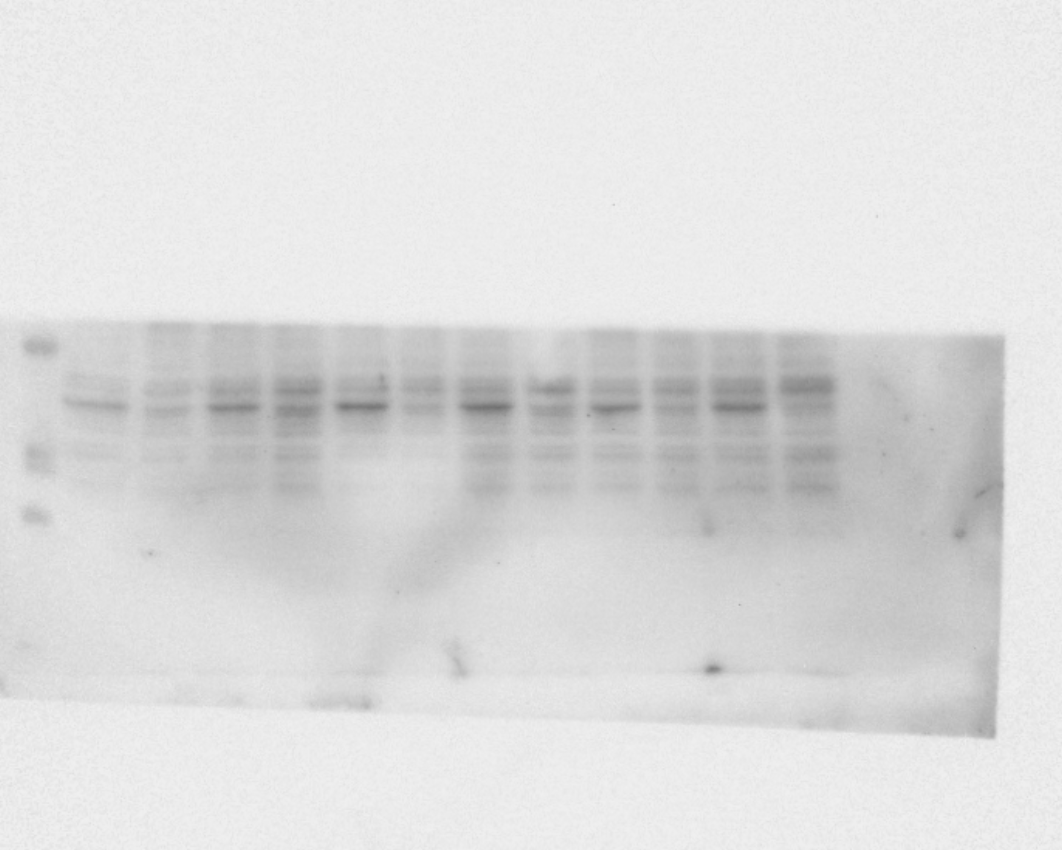

Supplement: Figure 3—source data 2. [file elife-89951-fig3-data2.zip › Figure 3-source data 2/SPOP_Figure 3-source data 2/Versteeg 2023-04-06 08h26m56s 37.612s(Chemiluminescence).tif]

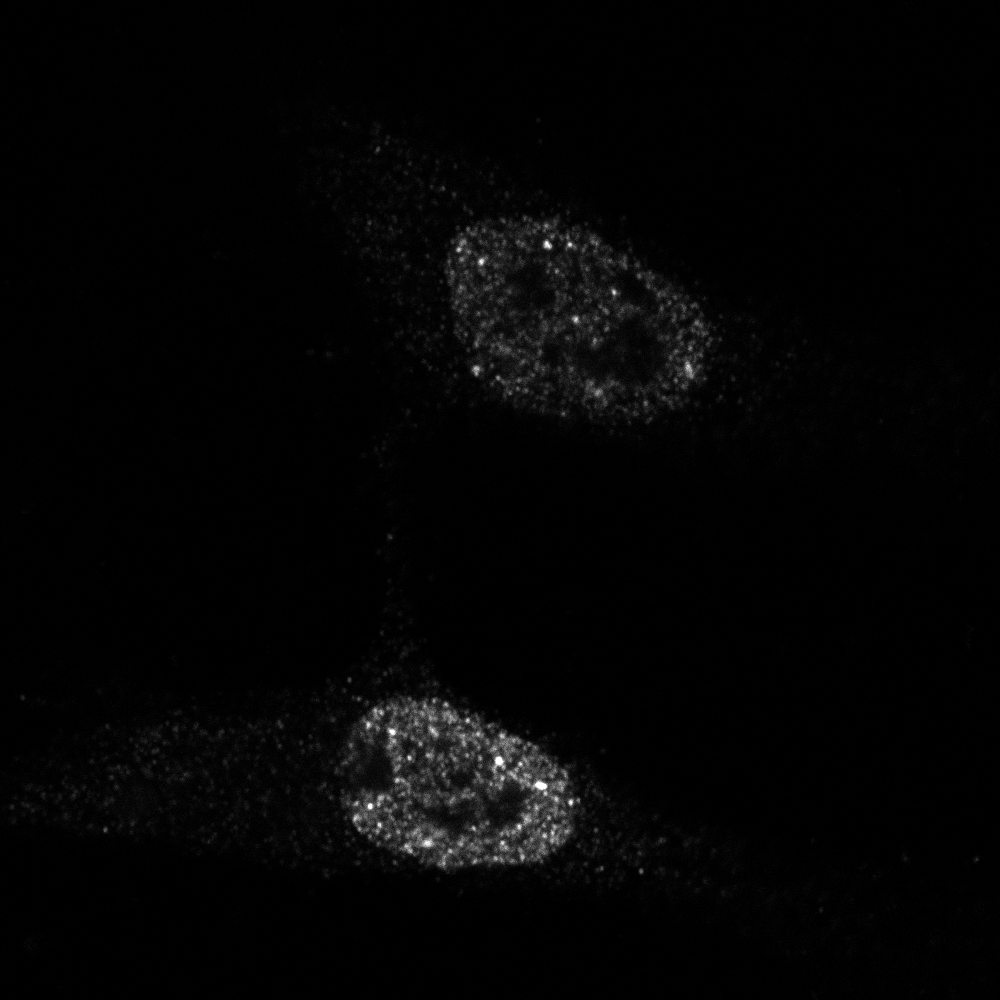

Supplement: Figure 4—source data 1. [file elife-89951-fig4-data1.zip › Figure 4-source data 1/HeLa_IRF1+SPOP_UNTR_Figure 4-source data 1.tif]

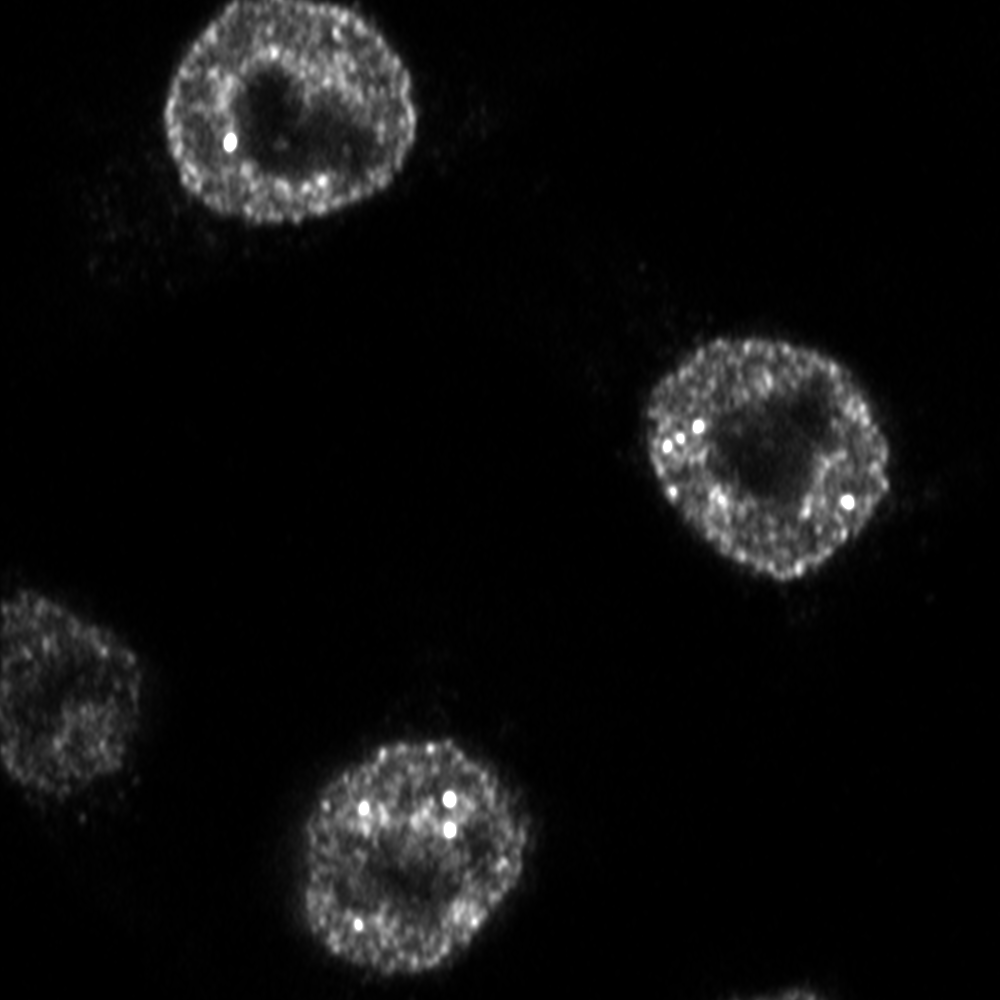

Supplement: Figure 4—source data 1. [file elife-89951-fig4-data1.zip › Figure 4-source data 1/RKO_IRF1+SPOP_UNTR_Figure 4-source data 1.tif]

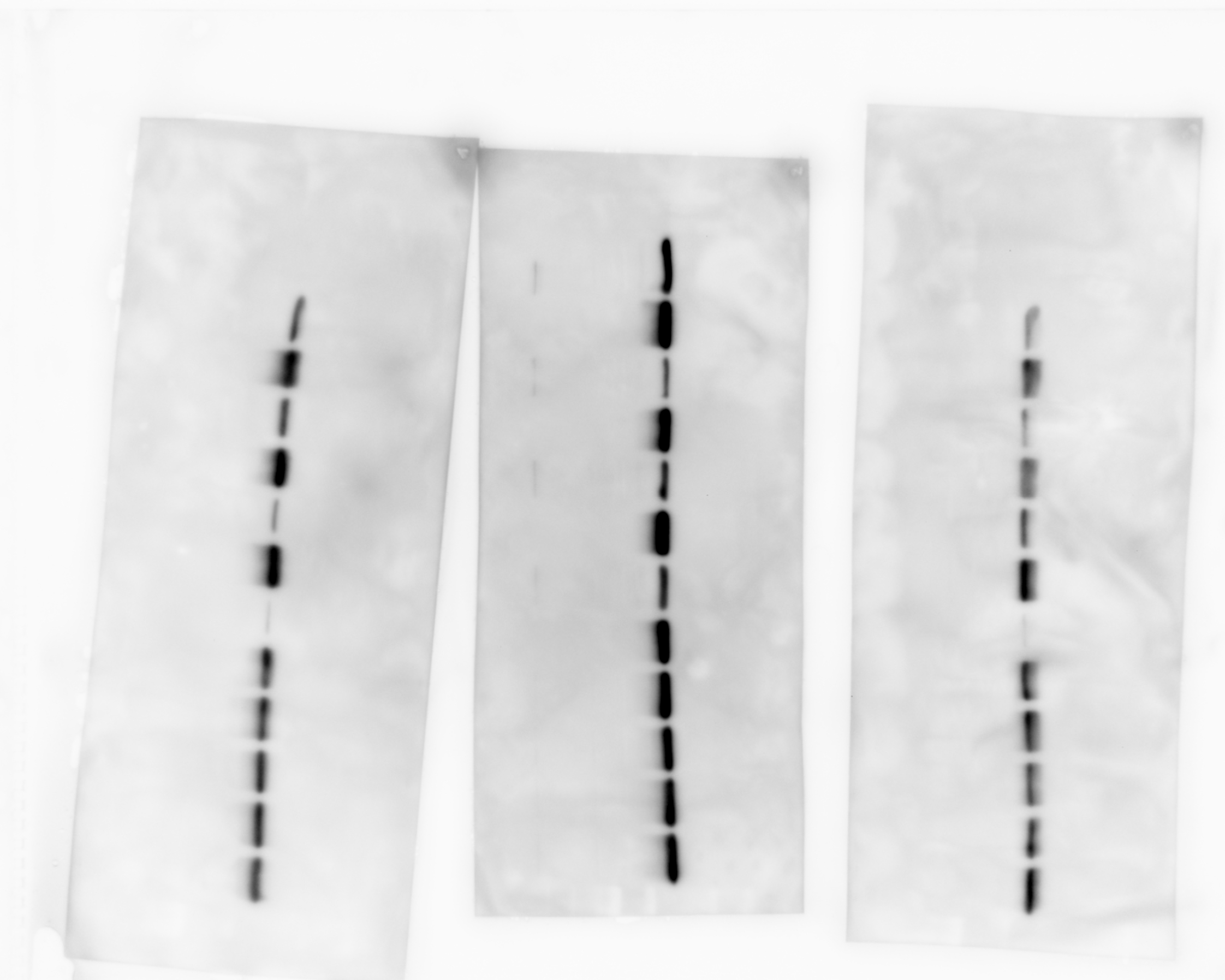

Supplement: Figure 4—source data 2. [file elife-89951-fig4-data2.zip › Figure 4-source data 2/ACTIN_Figure 4-source data 2/Versteeg 2023-03-27 13h57m54s 60.000s(Chemiluminescence).jpg]

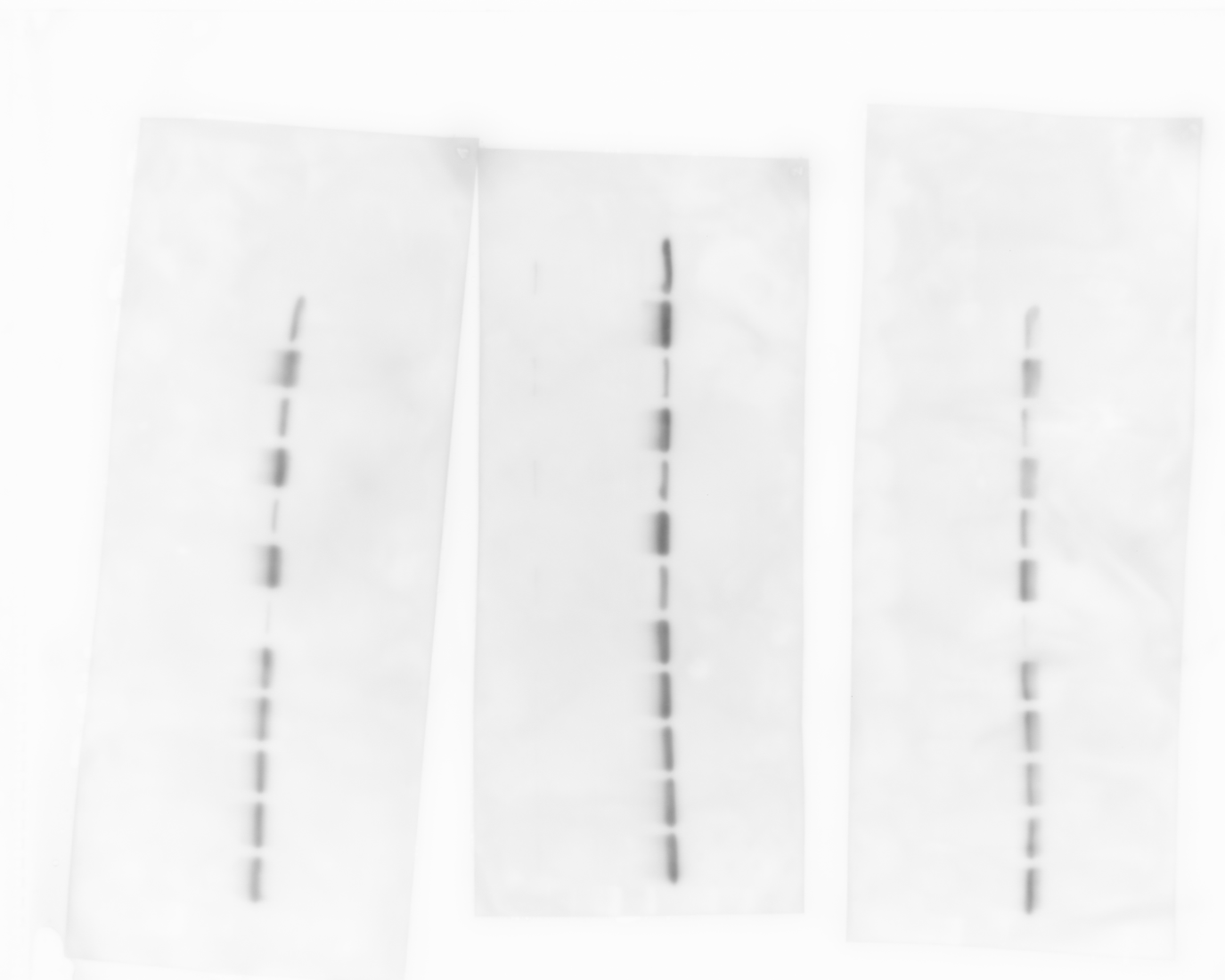

Supplement: Figure 4—source data 2. [file elife-89951-fig4-data2.zip › Figure 4-source data 2/ACTIN_Figure 4-source data 2/Versteeg 2023-03-27 13h57m54s 60.000s(Chemiluminescence).raw16.tif]

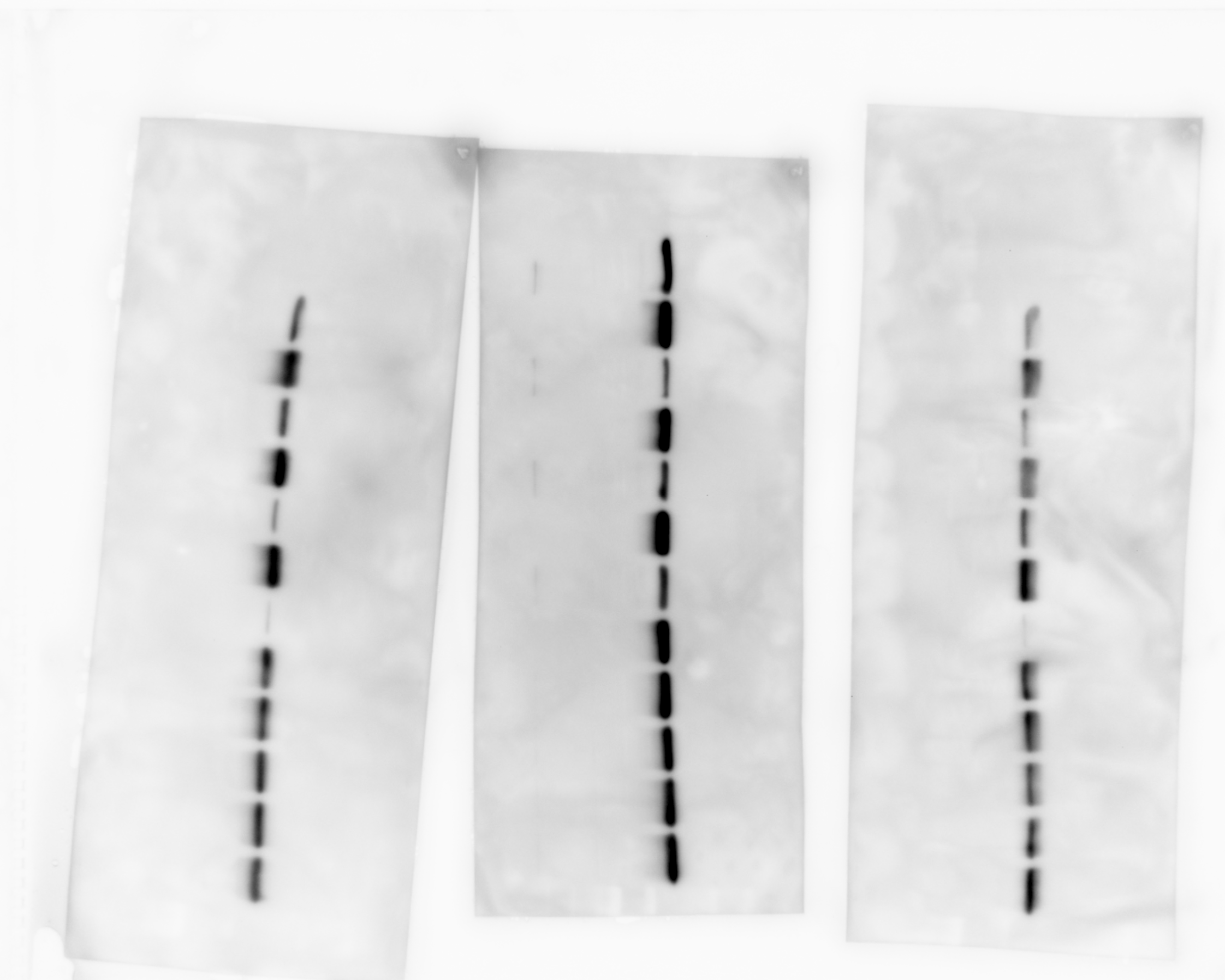

Supplement: Figure 4—source data 2. [file elife-89951-fig4-data2.zip › Figure 4-source data 2/ACTIN_Figure 4-source data 2/Versteeg 2023-03-27 13h57m54s 60.000s(Chemiluminescence).tif]

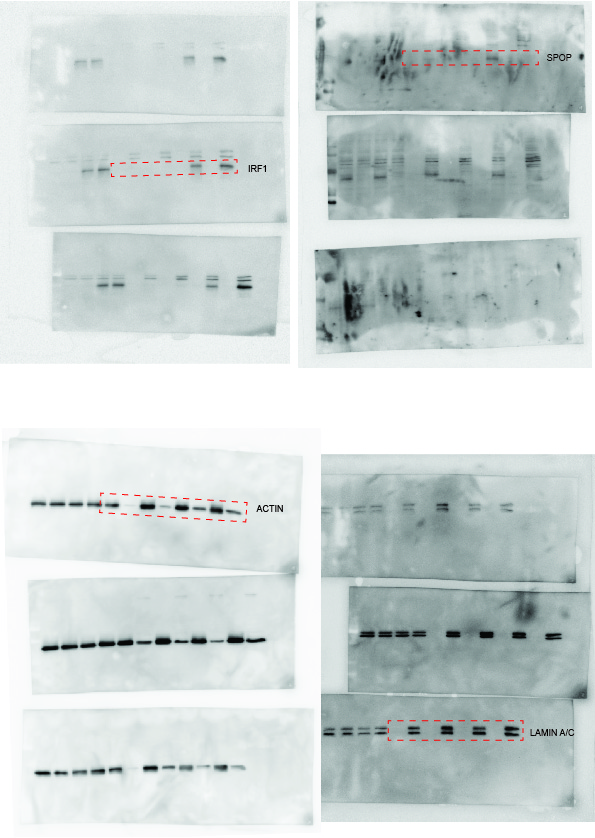

Supplement: Figure 4—source data 2. [file elife-89951-fig4-data2.zip › Figure 4-source data 2/Figure 4-source data 2.jpg]

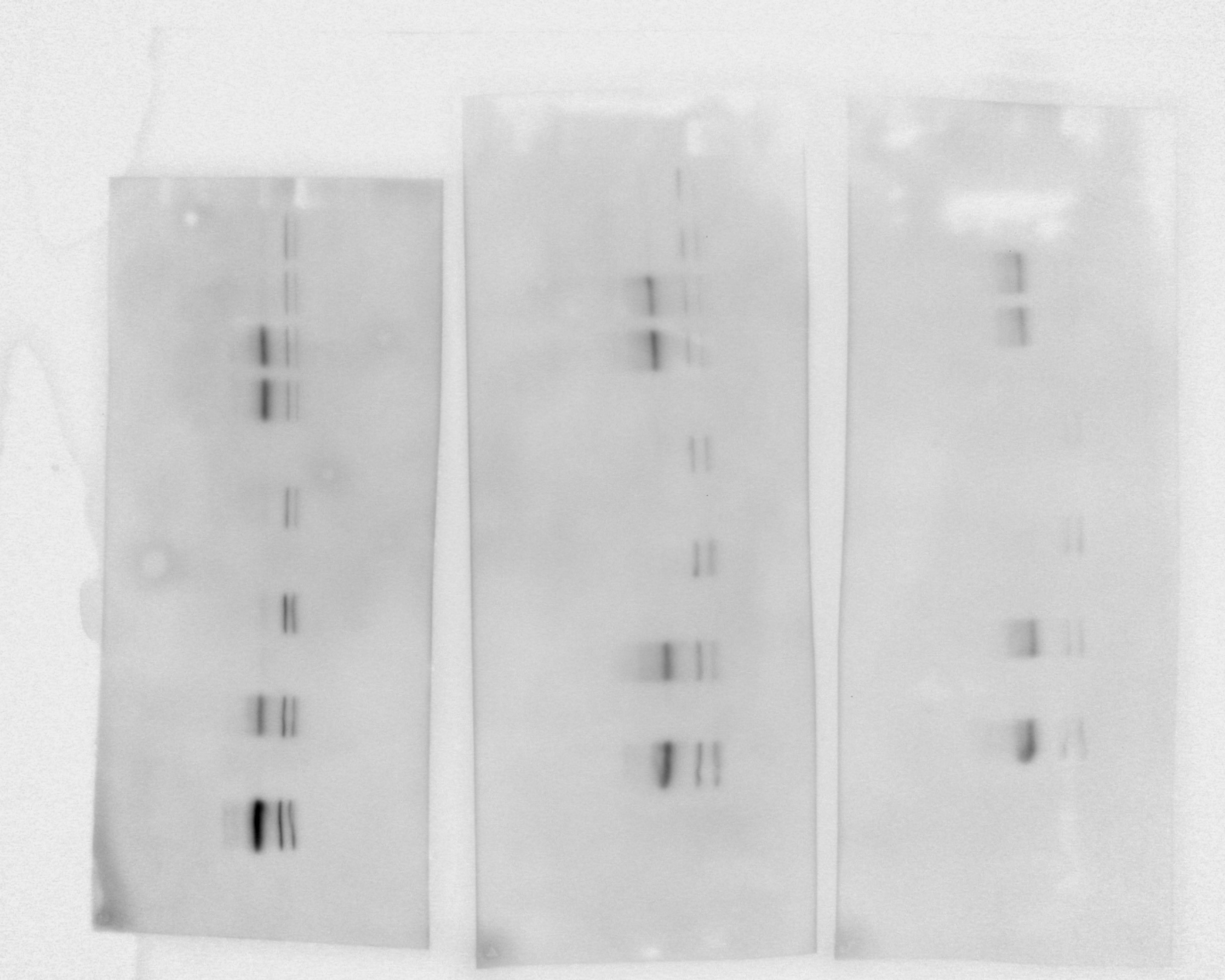

Supplement: Figure 4—source data 2. [file elife-89951-fig4-data2.zip › Figure 4-source data 2/IRF1_Figure 4-source data 2/Versteeg 2023-03-23 11h07m13s 59.995s(Chemiluminescence).jpg]

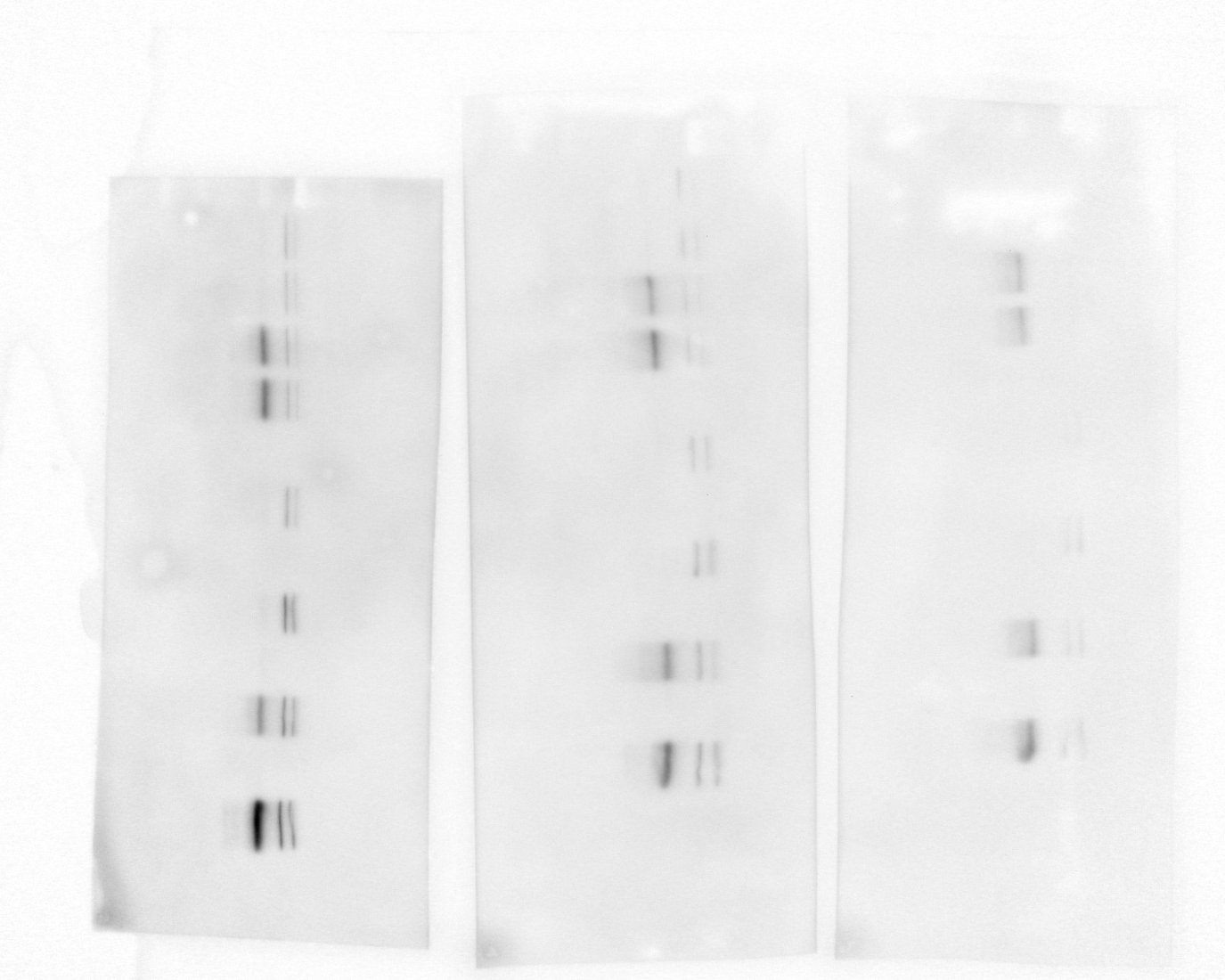

Supplement: Figure 4—source data 2. [file elife-89951-fig4-data2.zip › Figure 4-source data 2/IRF1_Figure 4-source data 2/Versteeg 2023-03-23 11h07m13s 59.995s(Chemiluminescence).raw16.tif]

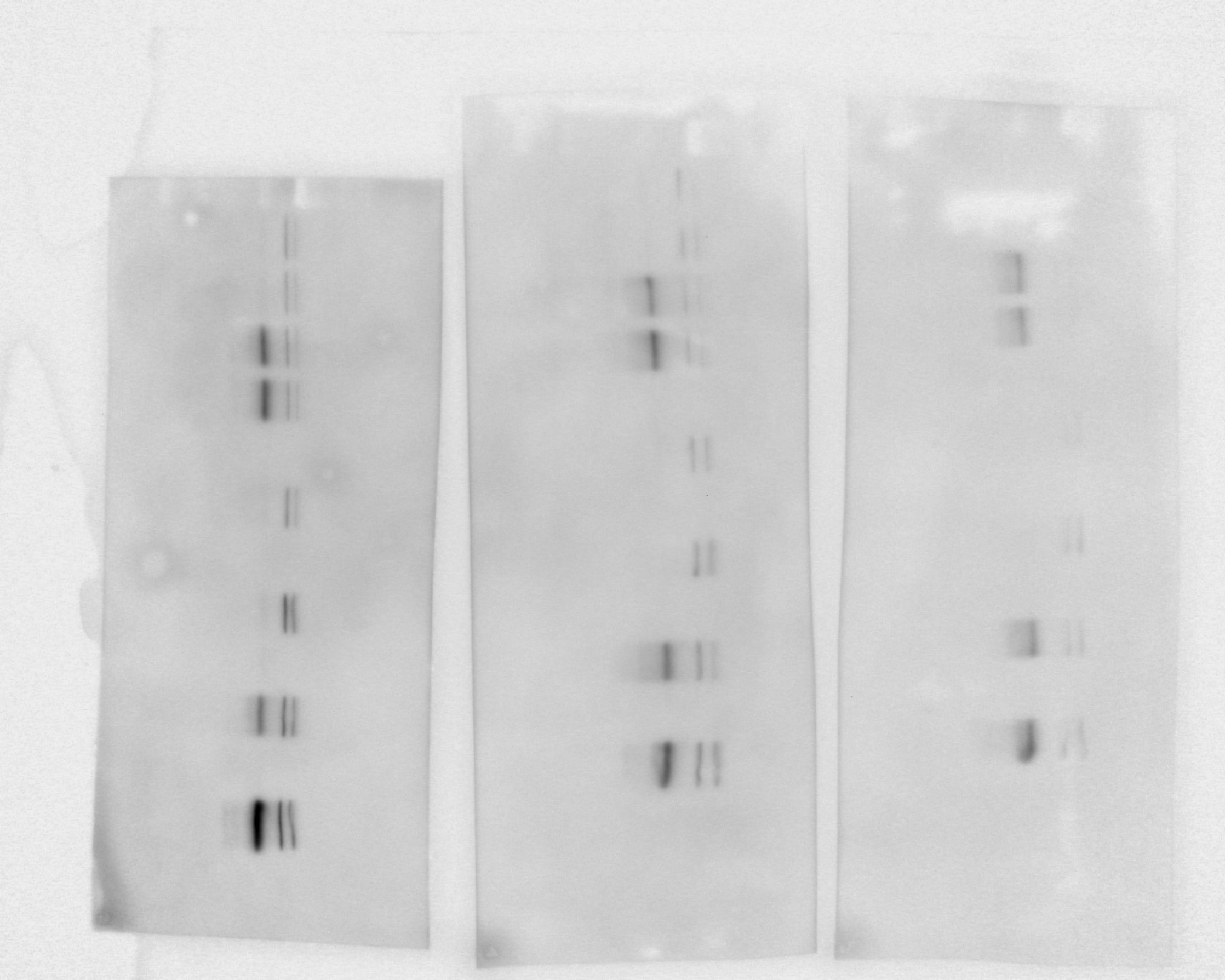

Supplement: Figure 4—source data 2. [file elife-89951-fig4-data2.zip › Figure 4-source data 2/IRF1_Figure 4-source data 2/Versteeg 2023-03-23 11h07m13s 59.995s(Chemiluminescence).tif]

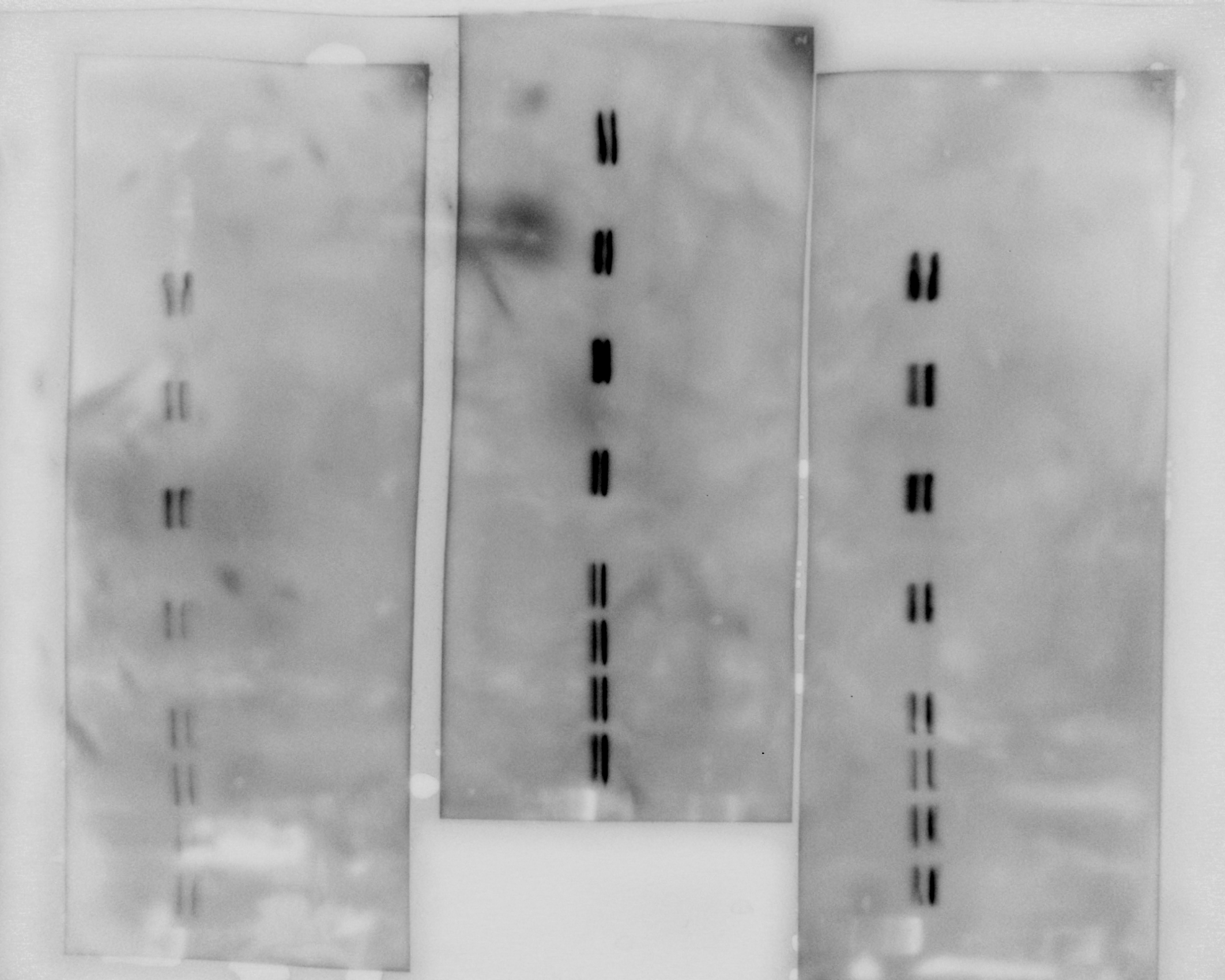

Supplement: Figure 4—source data 2. [file elife-89951-fig4-data2.zip › Figure 4-source data 2/LAMIN AC_Figure 4-source data 2/Versteeg 2023-03-23 15h46m32s 10.000s(Chemiluminescence).jpg]

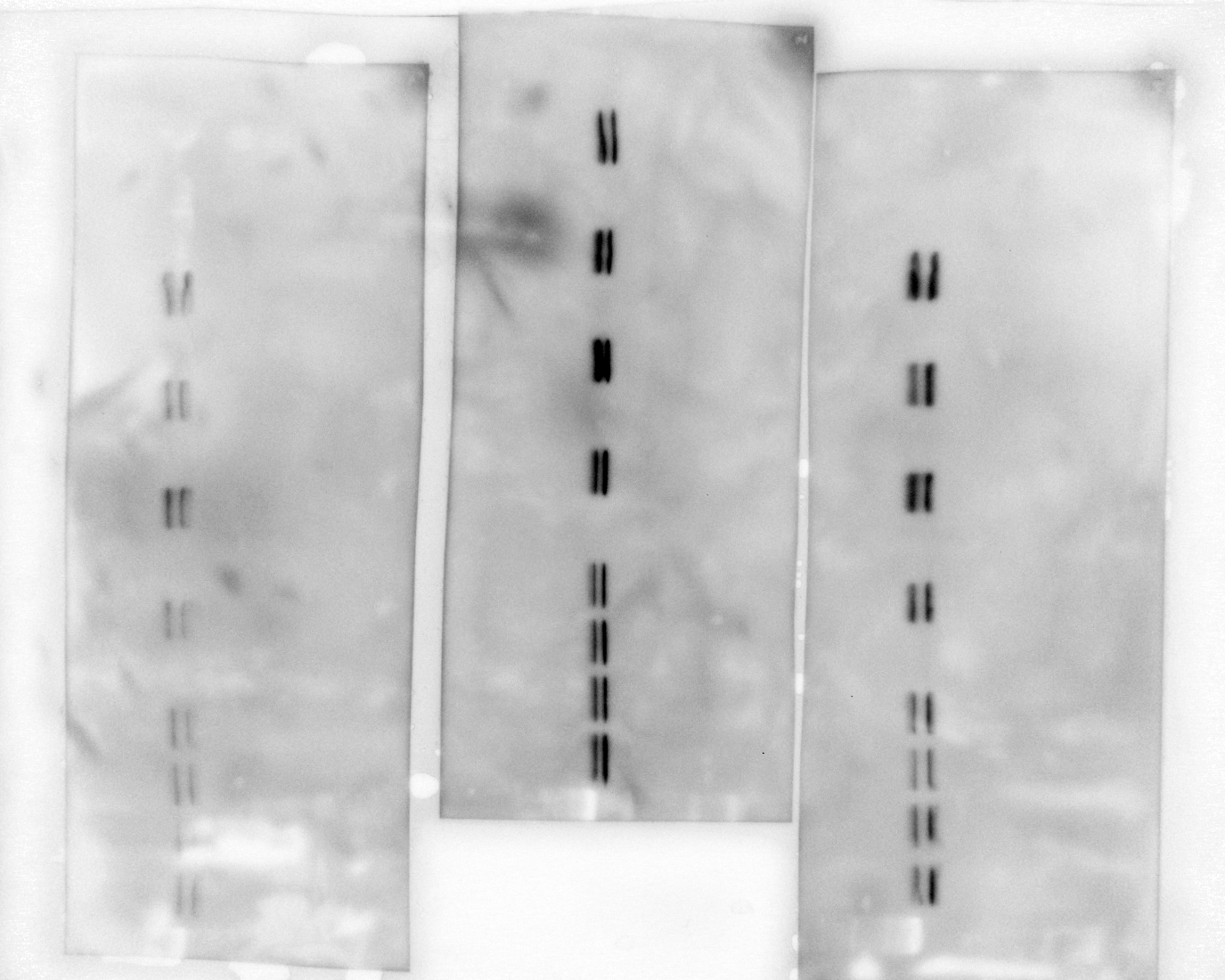

Supplement: Figure 4—source data 2. [file elife-89951-fig4-data2.zip › Figure 4-source data 2/LAMIN AC_Figure 4-source data 2/Versteeg 2023-03-23 15h46m32s 10.000s(Chemiluminescence).raw16.tif]

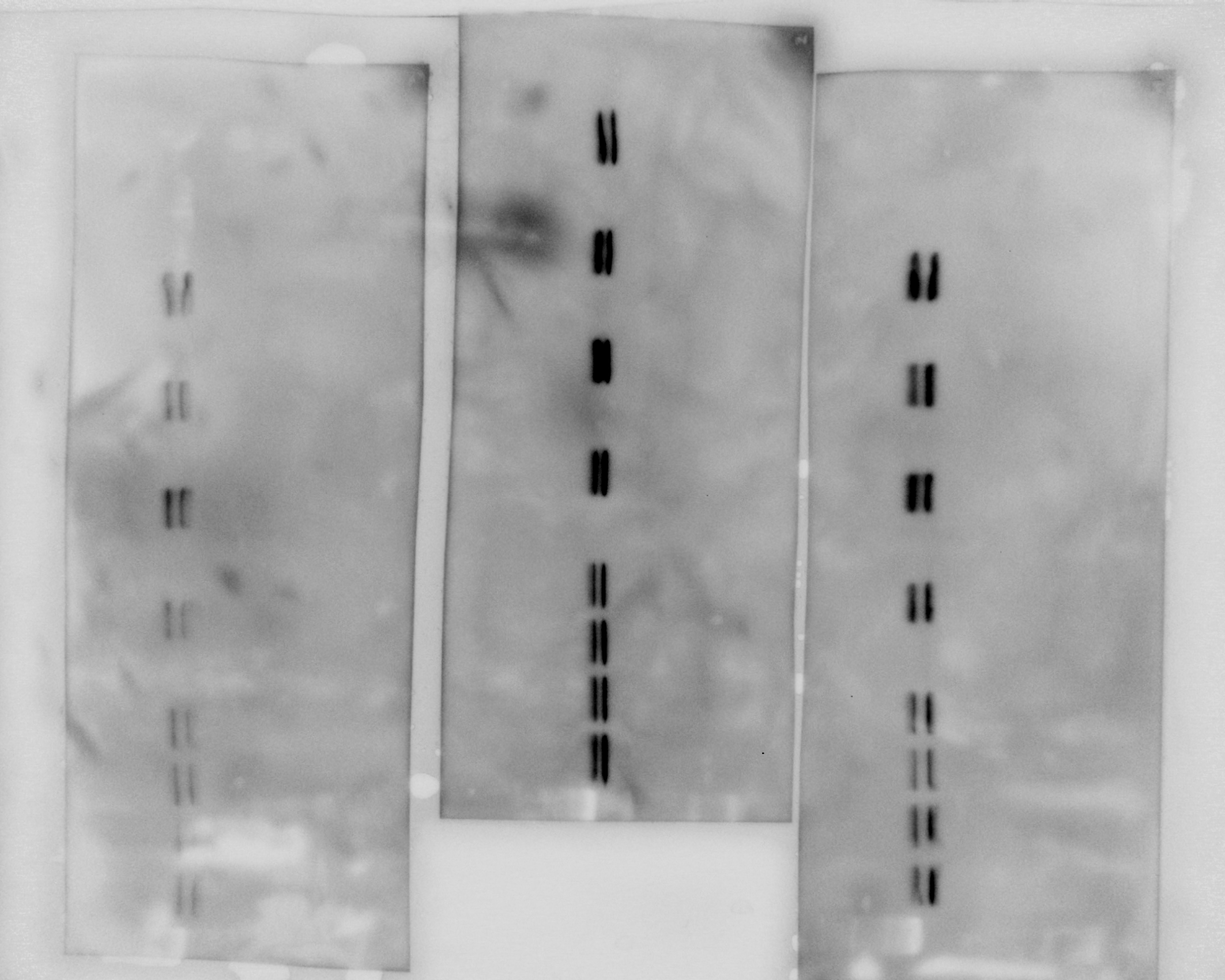

Supplement: Figure 4—source data 2. [file elife-89951-fig4-data2.zip › Figure 4-source data 2/LAMIN AC_Figure 4-source data 2/Versteeg 2023-03-23 15h46m32s 10.000s(Chemiluminescence).tif]

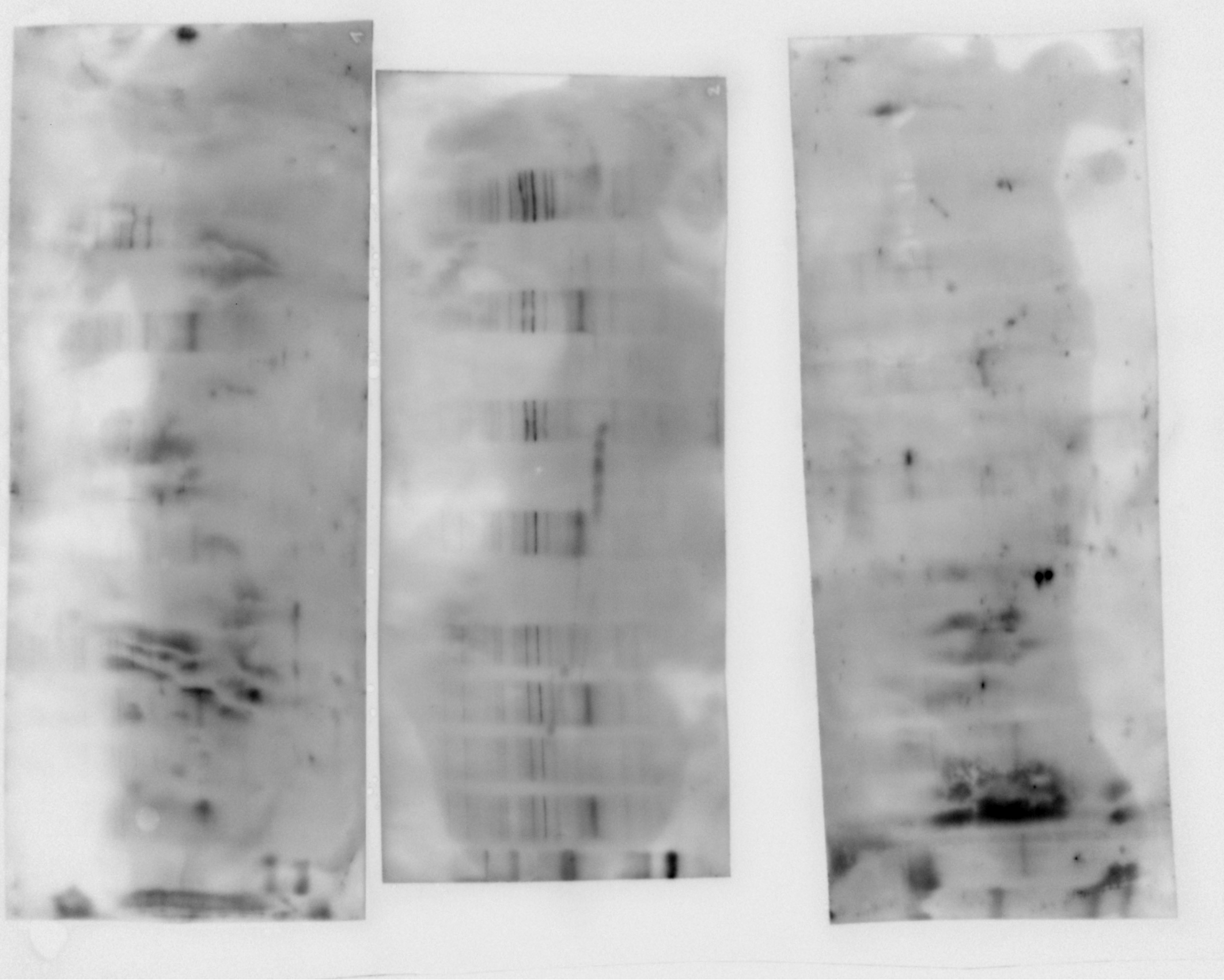

Supplement: Figure 4—source data 2. [file elife-89951-fig4-data2.zip › Figure 4-source data 2/SPOP_Figure 4-source data 2/Run 2023-03-24 10h45m37s 13.204s(Chemiluminescence).jpg]

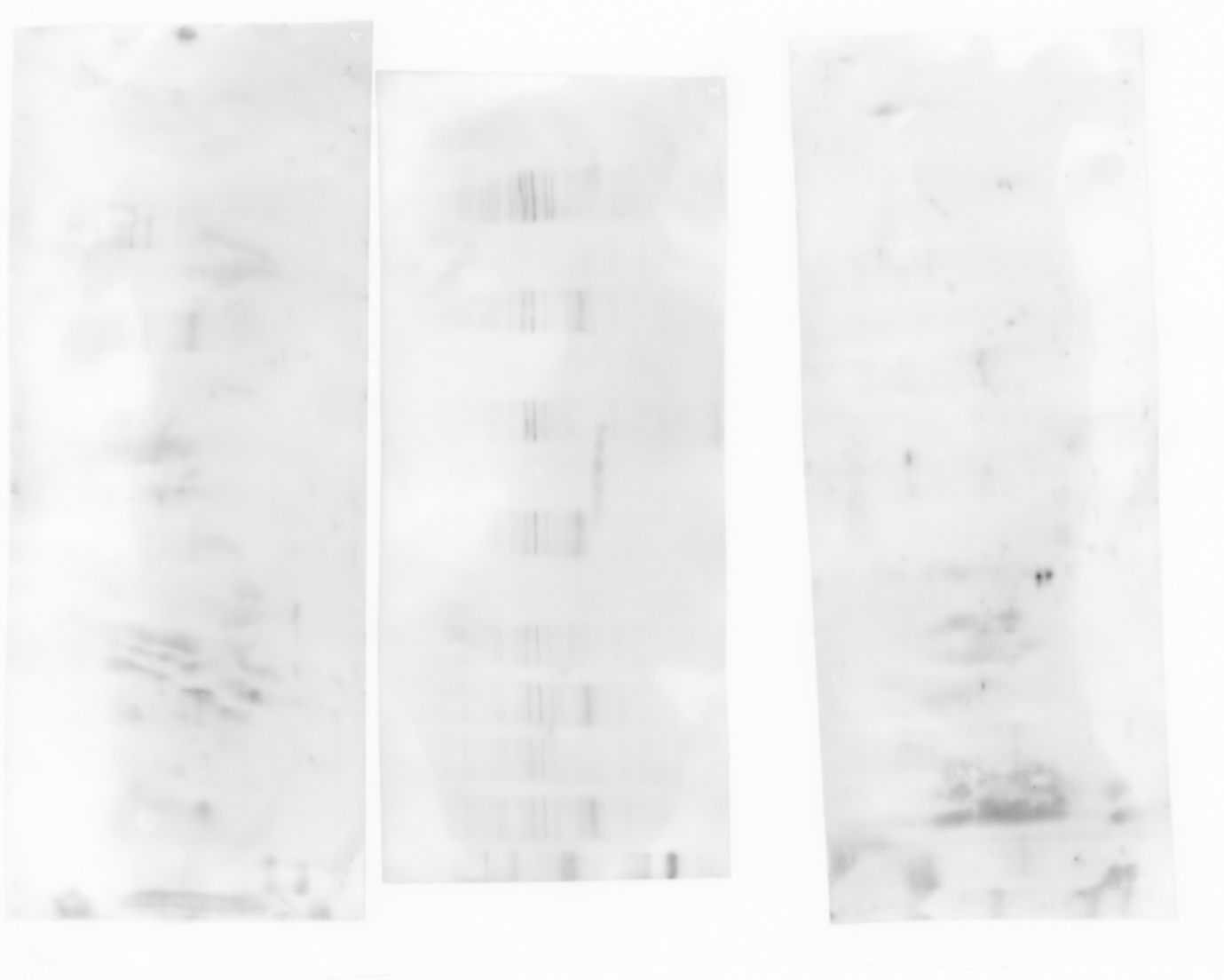

Supplement: Figure 4—source data 2. [file elife-89951-fig4-data2.zip › Figure 4-source data 2/SPOP_Figure 4-source data 2/Run 2023-03-24 10h45m37s 13.204s(Chemiluminescence).raw16.tif]

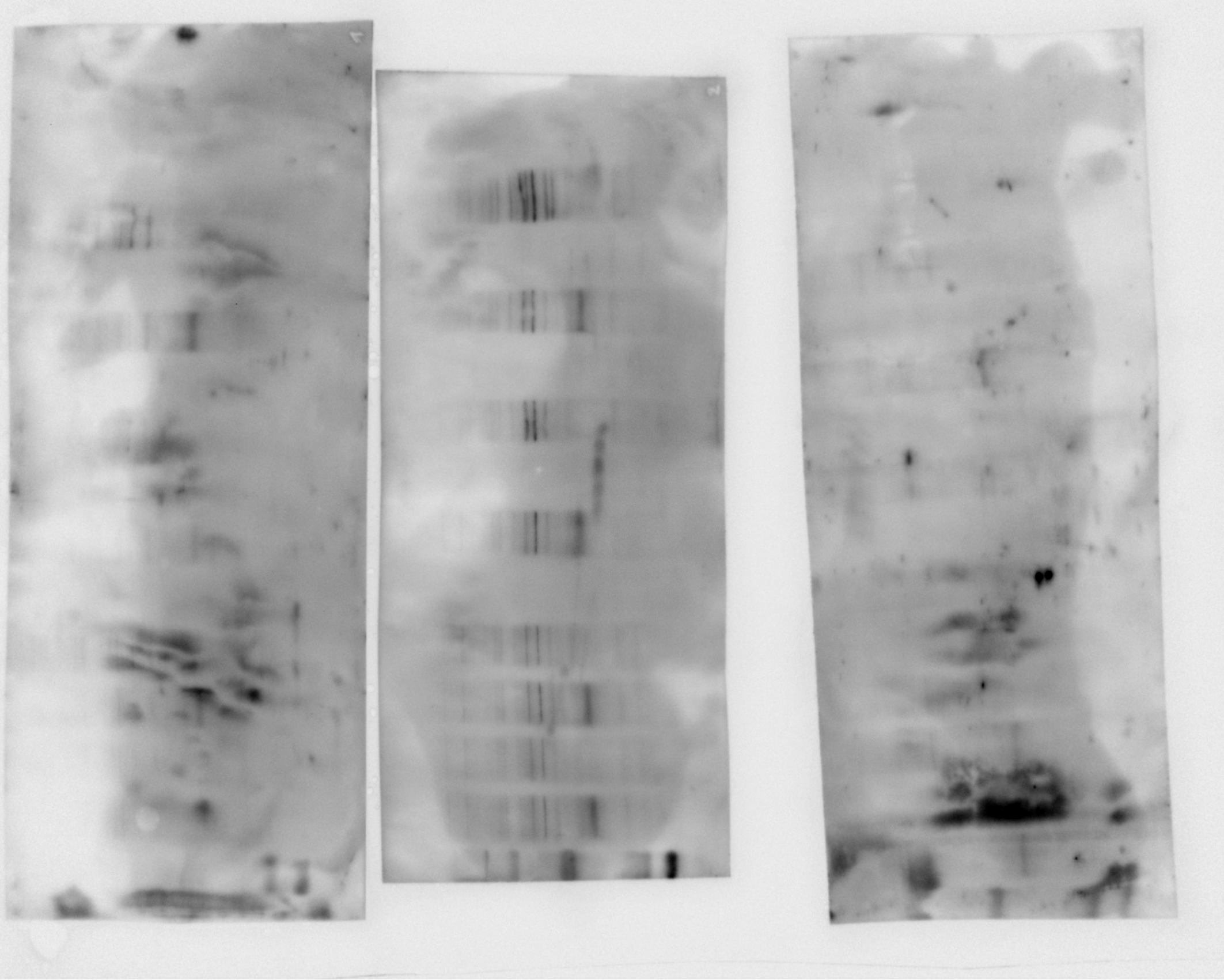

Supplement: Figure 4—source data 2. [file elife-89951-fig4-data2.zip › Figure 4-source data 2/SPOP_Figure 4-source data 2/Run 2023-03-24 10h45m37s 13.204s(Chemiluminescence).tif]

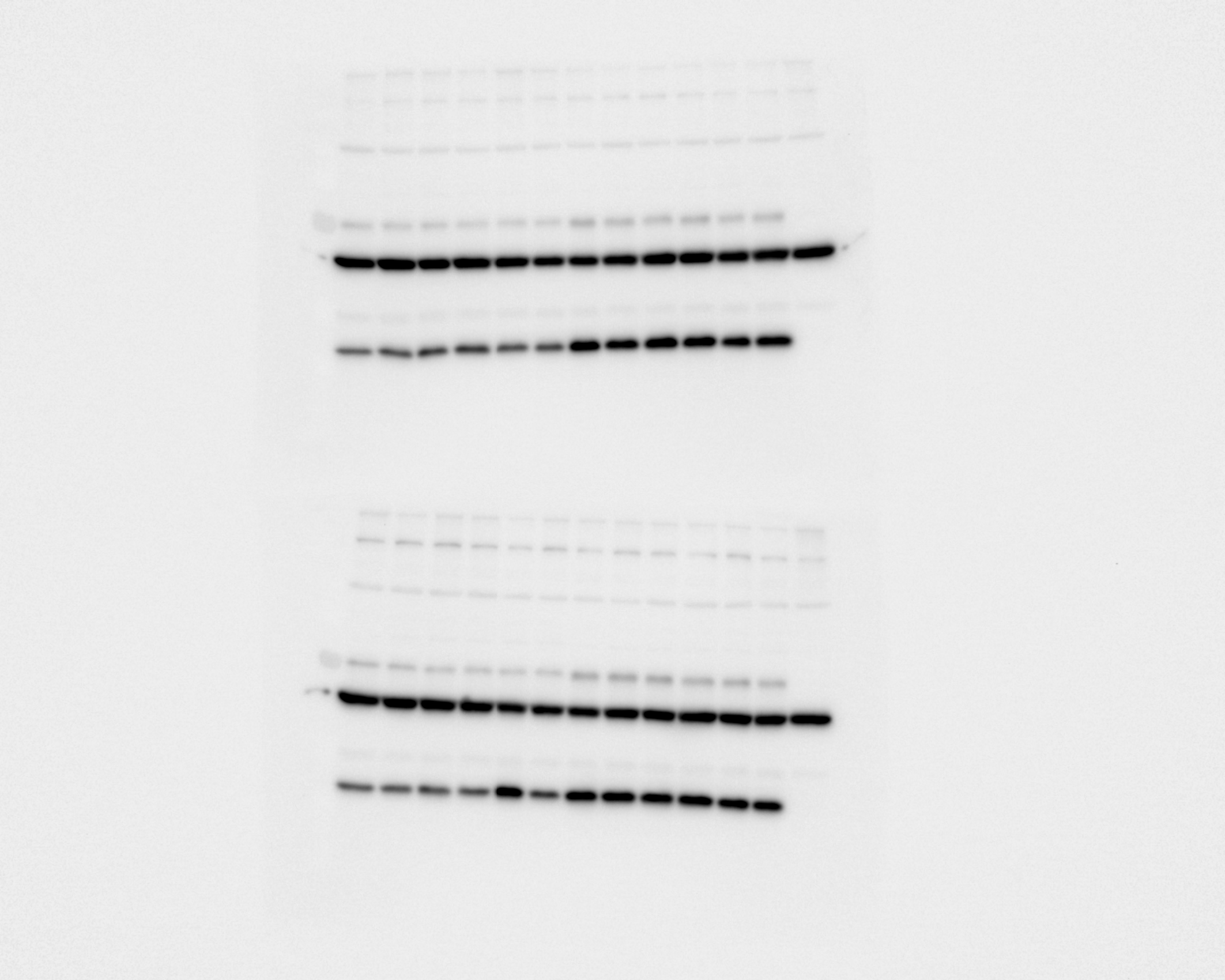

Supplement: Figure 4—source data 3. [file elife-89951-fig4-data3.zip › Figure 4-source data 3/ACTIN_Figure 4-source data 3/versteeg 2020-03-12 16h41m27s(Chemiluminescence).tif]

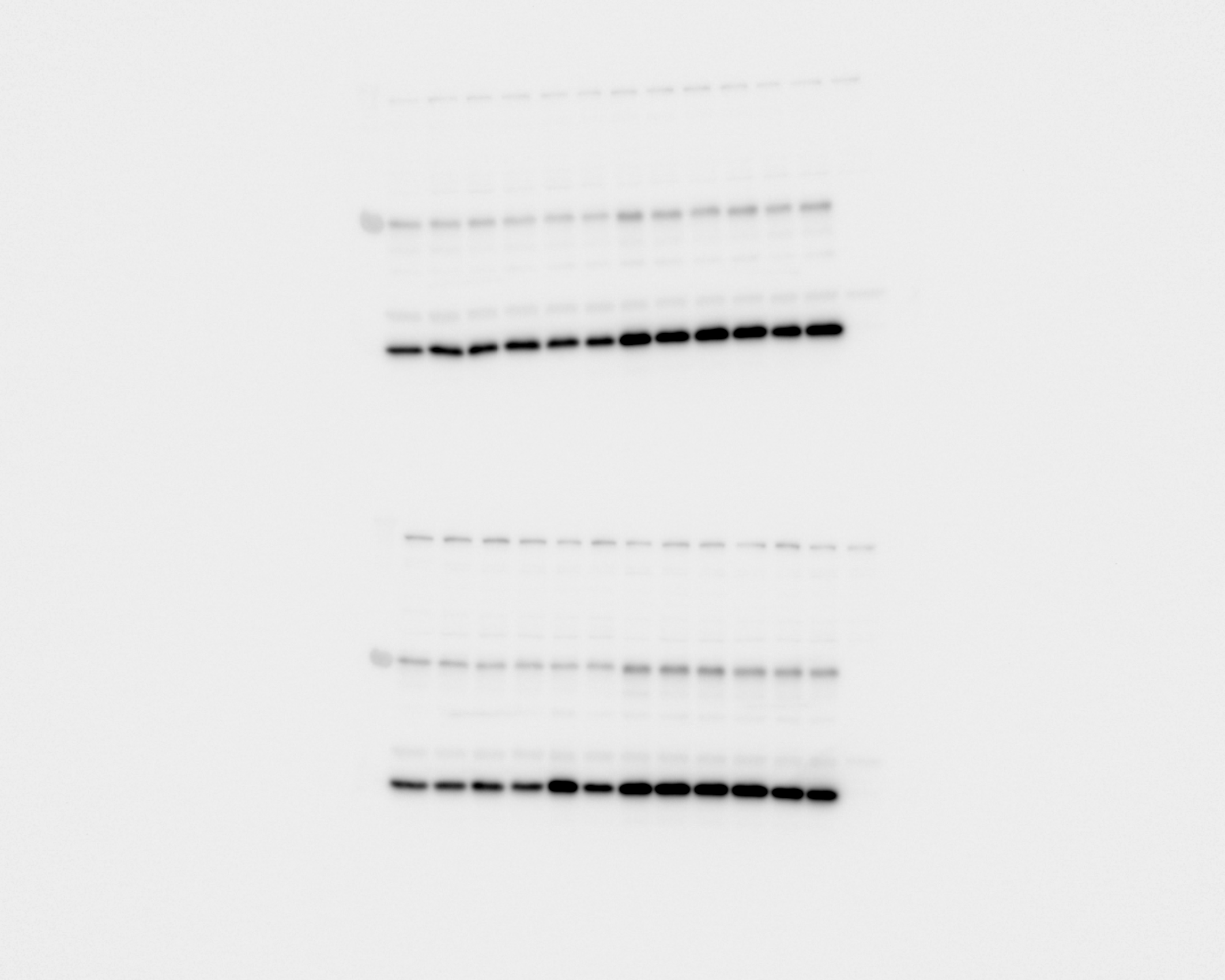

Supplement: Figure 4—source data 3. [file elife-89951-fig4-data3.zip › Figure 4-source data 3/EGFP_Figure 4-source data 3/versteeg 2020-03-12 13h37m14s(Chemiluminescence).tif]

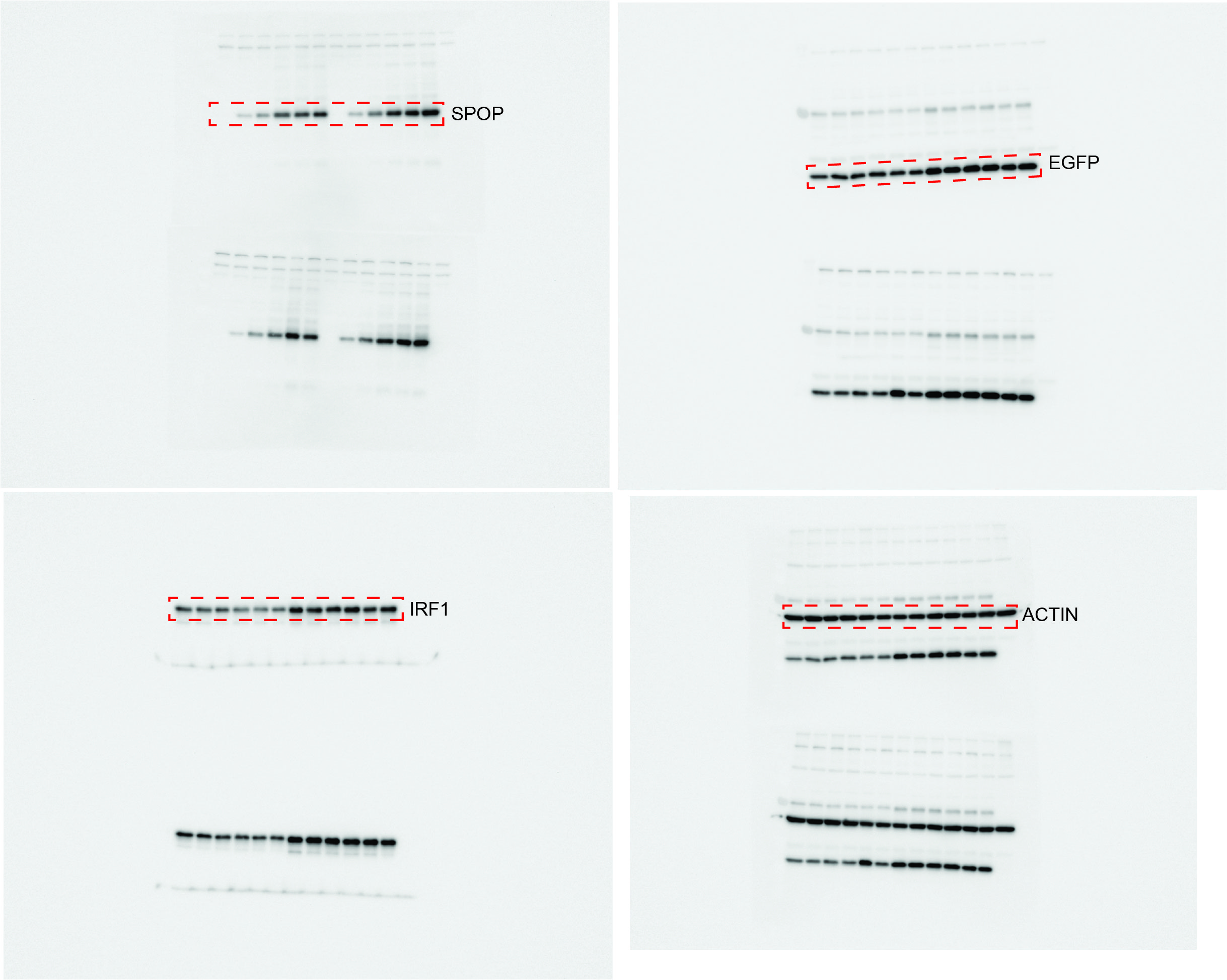

Supplement: Figure 4—source data 3. [file elife-89951-fig4-data3.zip › Figure 4-source data 3/Figure 4-source data 3.jpg]

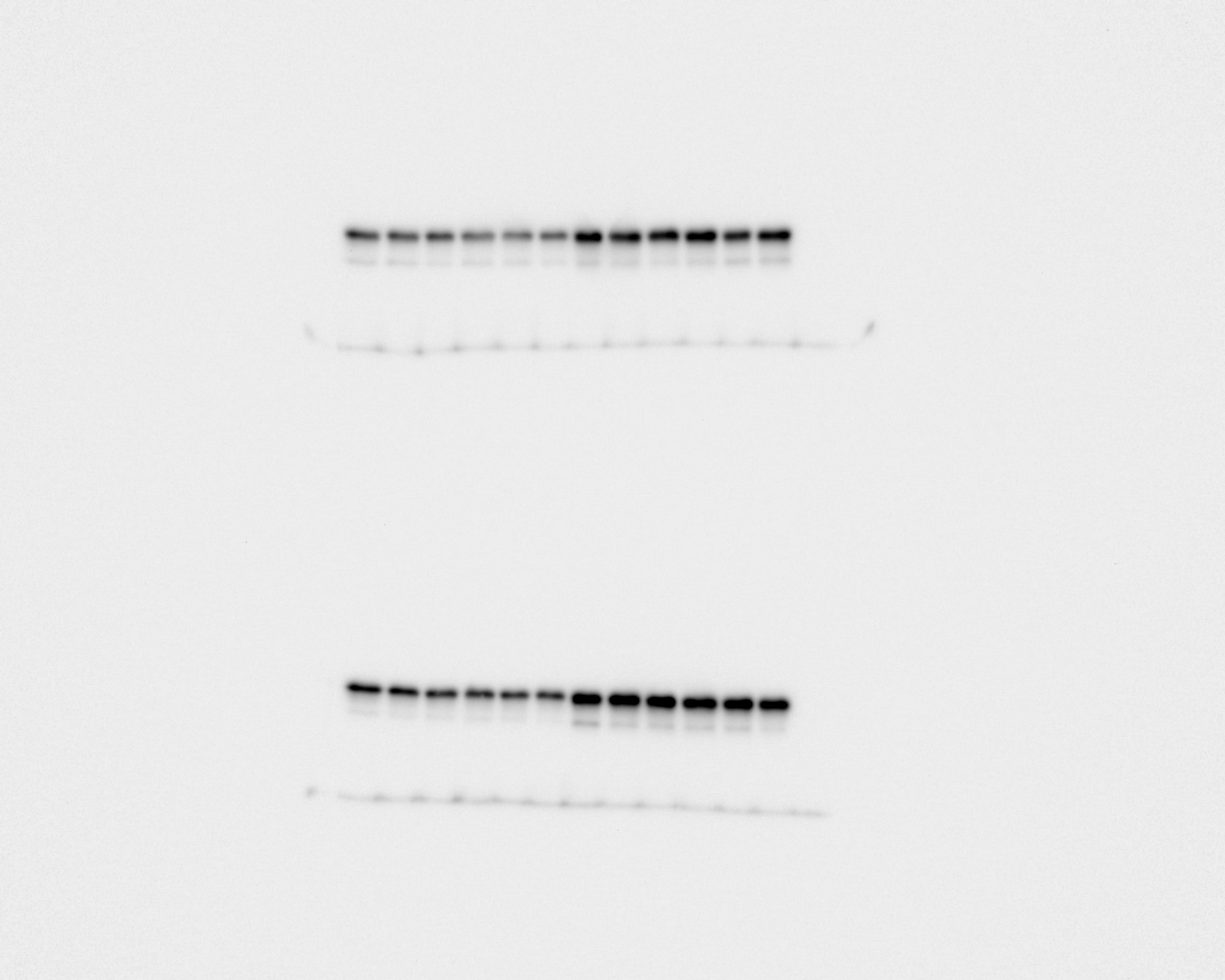

Supplement: Figure 4—source data 3. [file elife-89951-fig4-data3.zip › Figure 4-source data 3/IRF1_Figure 4-source data 3/versteeg 2020-03-11 16h15m12s(Chemiluminescence).tif]

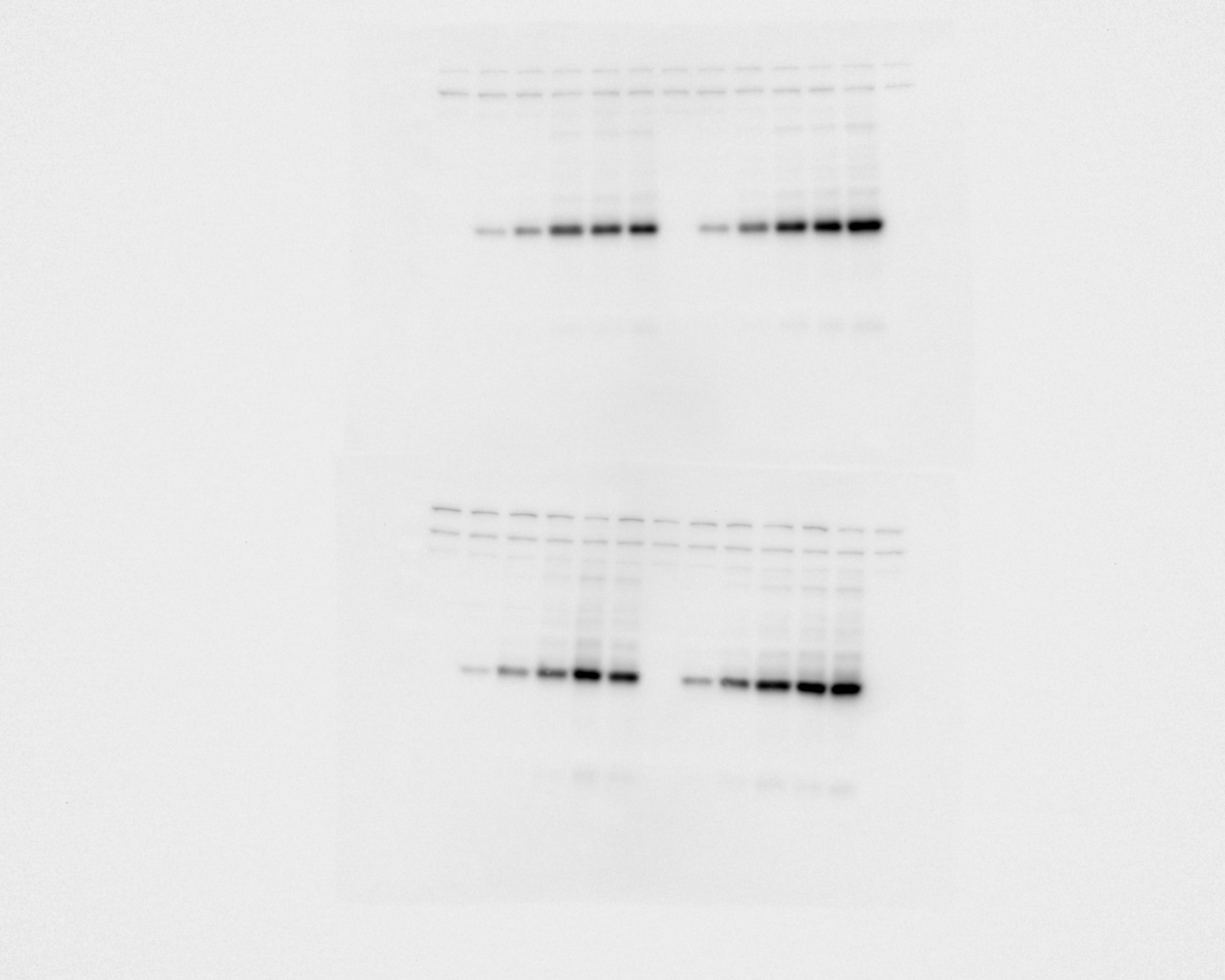

Supplement: Figure 4—source data 3. [file elife-89951-fig4-data3.zip › Figure 4-source data 3/SPOP_Figure 4-source data 3/versteeg 2020-03-13 12h57m28s(Chemiluminescence).tif]

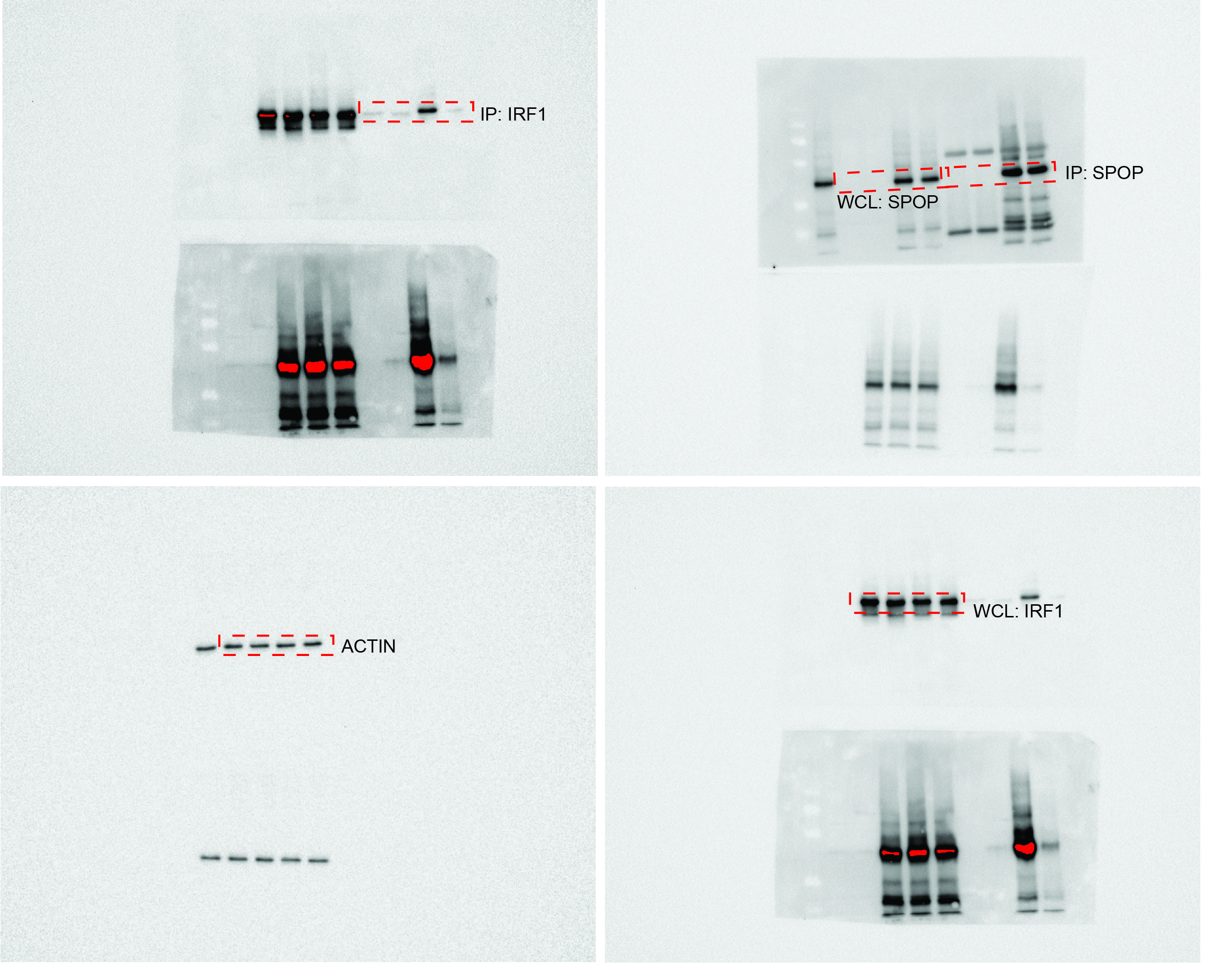

Supplement: Figure 4—source data 4. [file elife-89951-fig4-data4.zip › Figure 4-source data 4/Figure 4-source data 4.jpg]

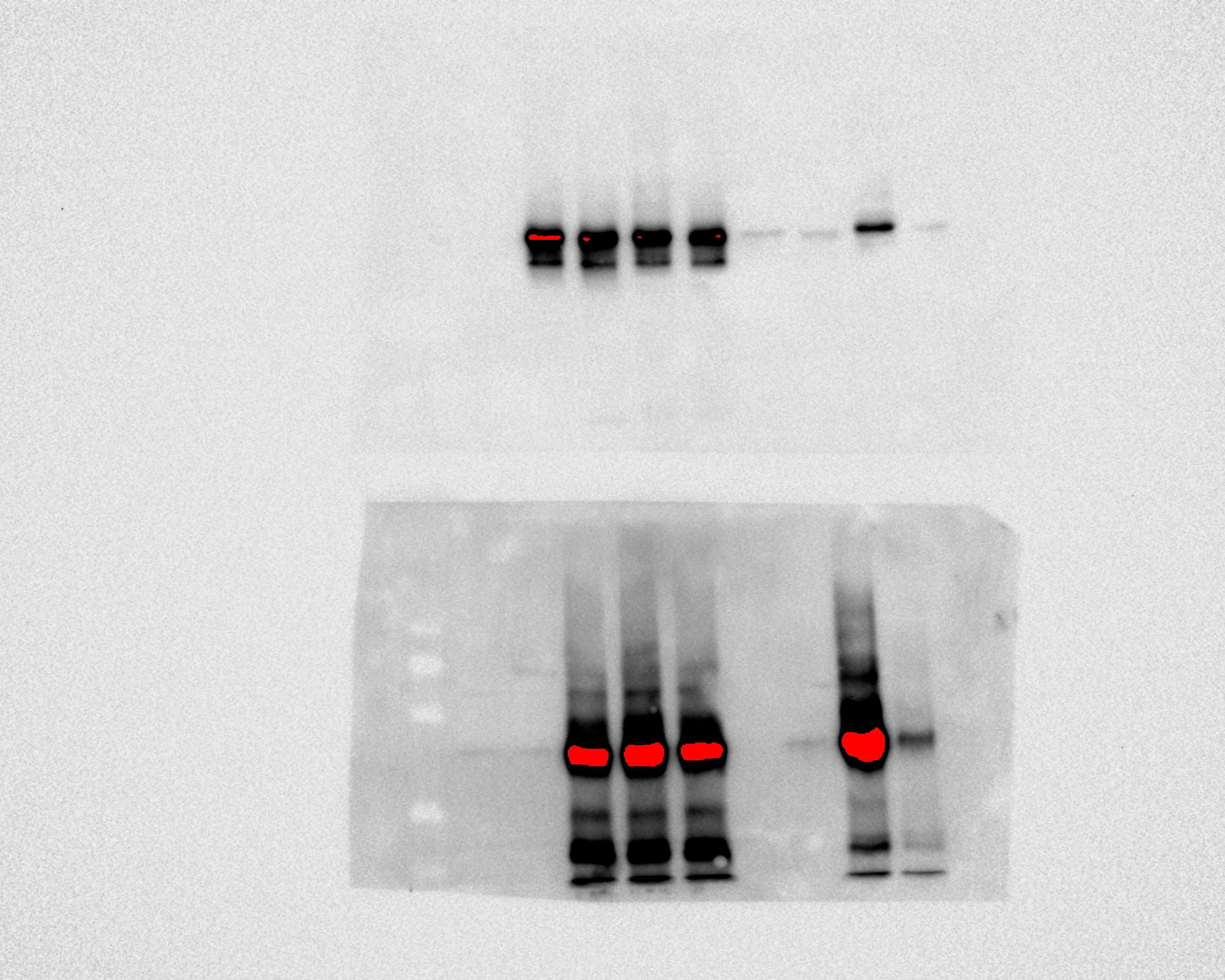

Supplement: Figure 4—source data 4. [file elife-89951-fig4-data4.zip › Figure 4-source data 4/IP_MYC-IRF1_Figure 4-source data 4/Versteeg 2022-02-24 14h38m35s 299.990s(Chemiluminescence).jpg]

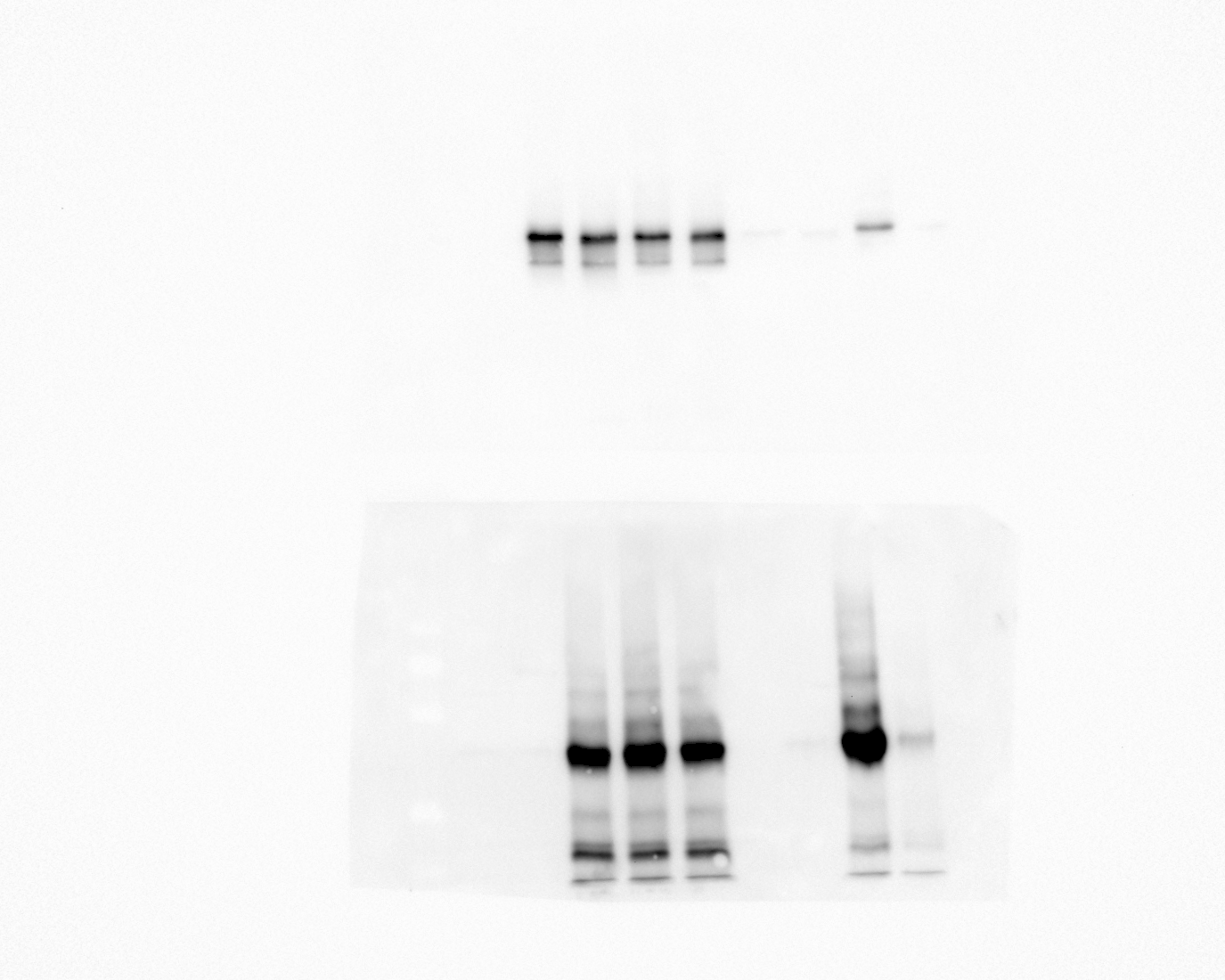

Supplement: Figure 4—source data 4. [file elife-89951-fig4-data4.zip › Figure 4-source data 4/IP_MYC-IRF1_Figure 4-source data 4/Versteeg 2022-02-24 14h38m35s 299.990s(Chemiluminescence).raw16.tif]

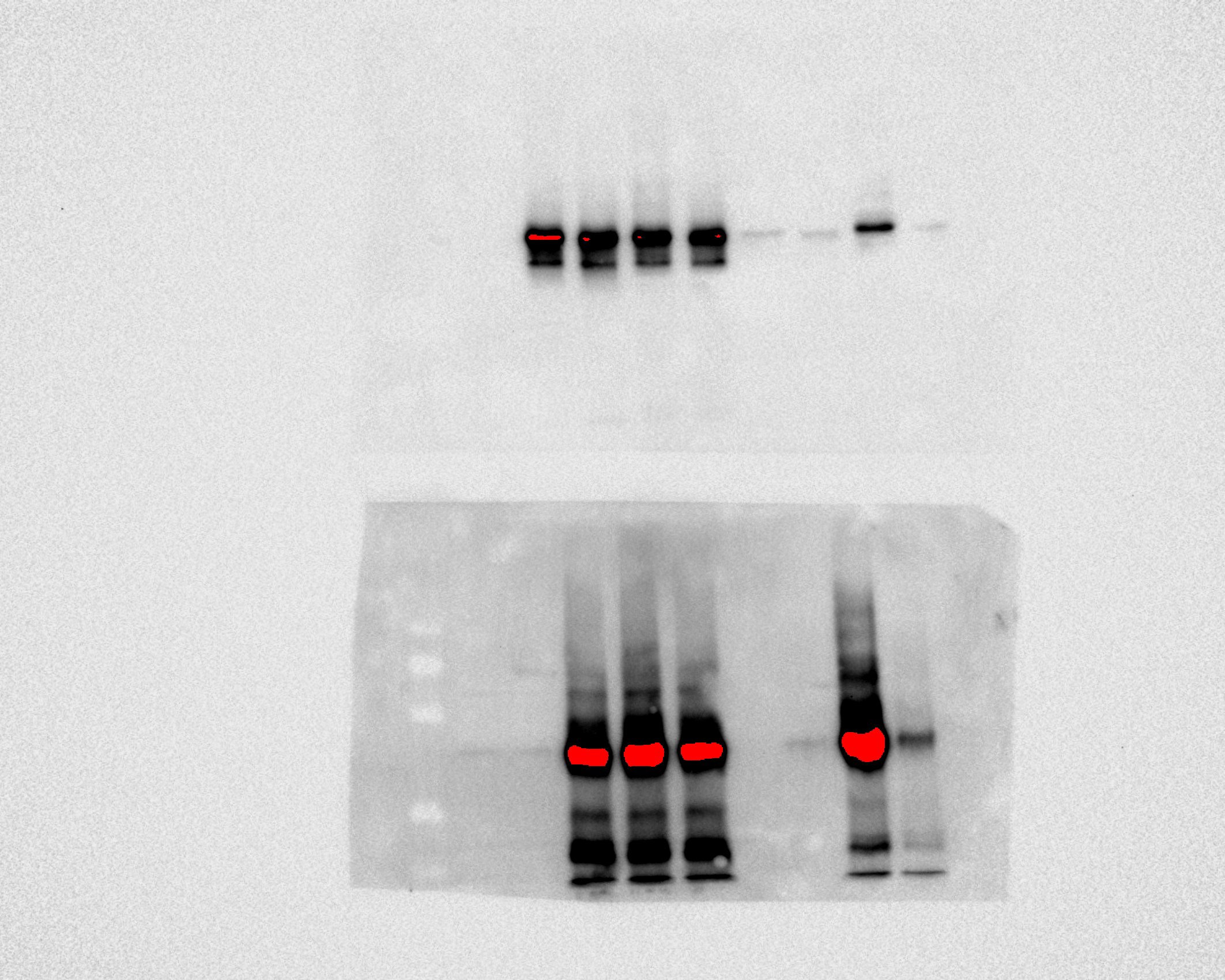

Supplement: Figure 4—source data 4. [file elife-89951-fig4-data4.zip › Figure 4-source data 4/IP_MYC-IRF1_Figure 4-source data 4/Versteeg 2022-02-24 14h38m35s 299.990s(Chemiluminescence).tif]

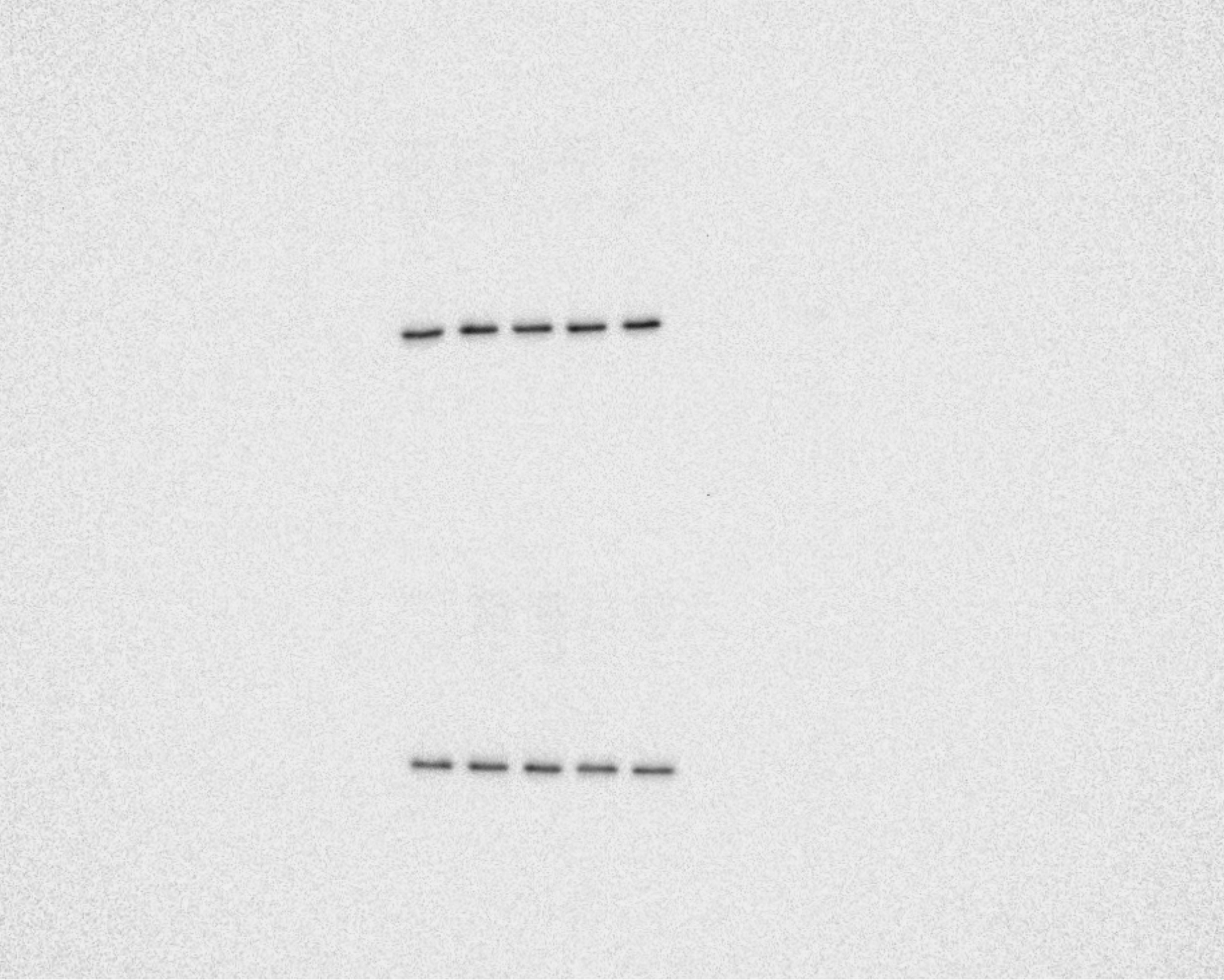

Supplement: Figure 4—source data 4. [file elife-89951-fig4-data4.zip › Figure 4-source data 4/WB_ACTIN_Figure 4-source data 4/Versteeg 2022-02-23 13h45m33s 59.464s(Chemiluminescence).jpg]

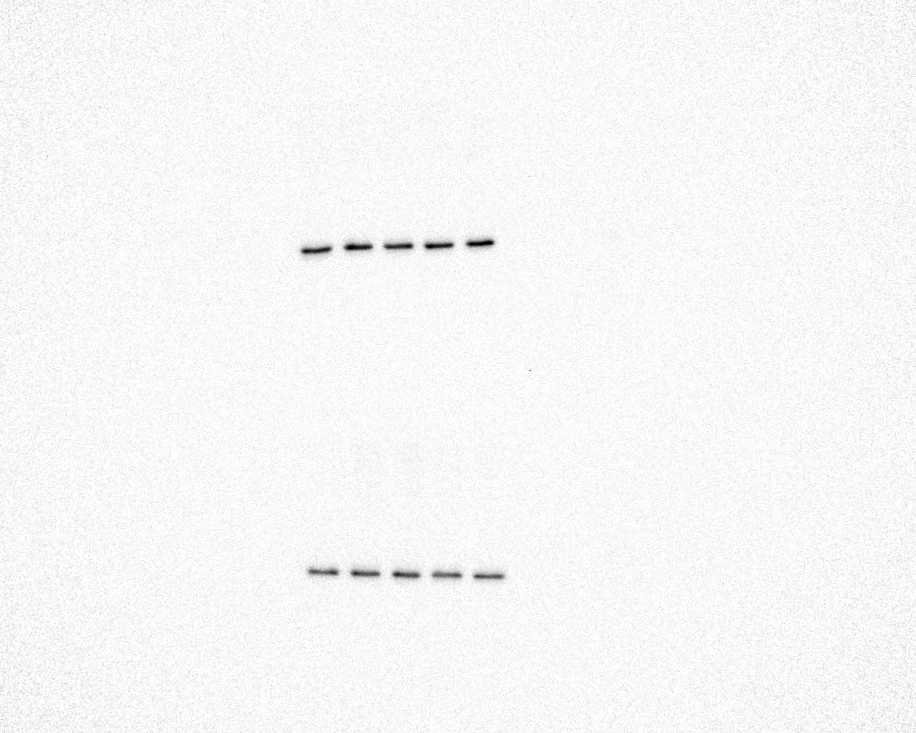

Supplement: Figure 4—source data 4. [file elife-89951-fig4-data4.zip › Figure 4-source data 4/WB_ACTIN_Figure 4-source data 4/Versteeg 2022-02-23 13h45m33s 59.464s(Chemiluminescence).raw16.tif]

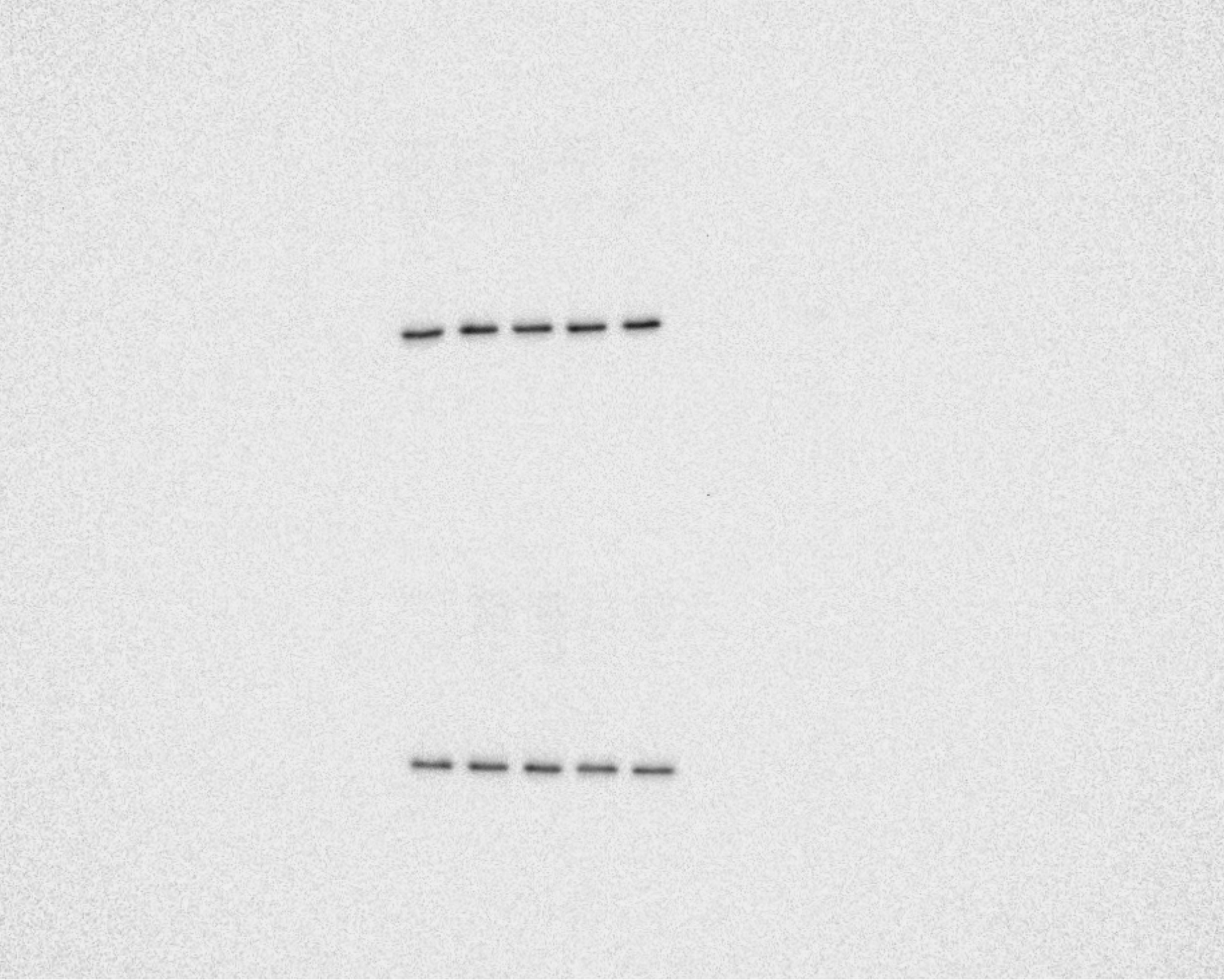

Supplement: Figure 4—source data 4. [file elife-89951-fig4-data4.zip › Figure 4-source data 4/WB_ACTIN_Figure 4-source data 4/Versteeg 2022-02-23 13h45m33s 59.464s(Chemiluminescence).tif]

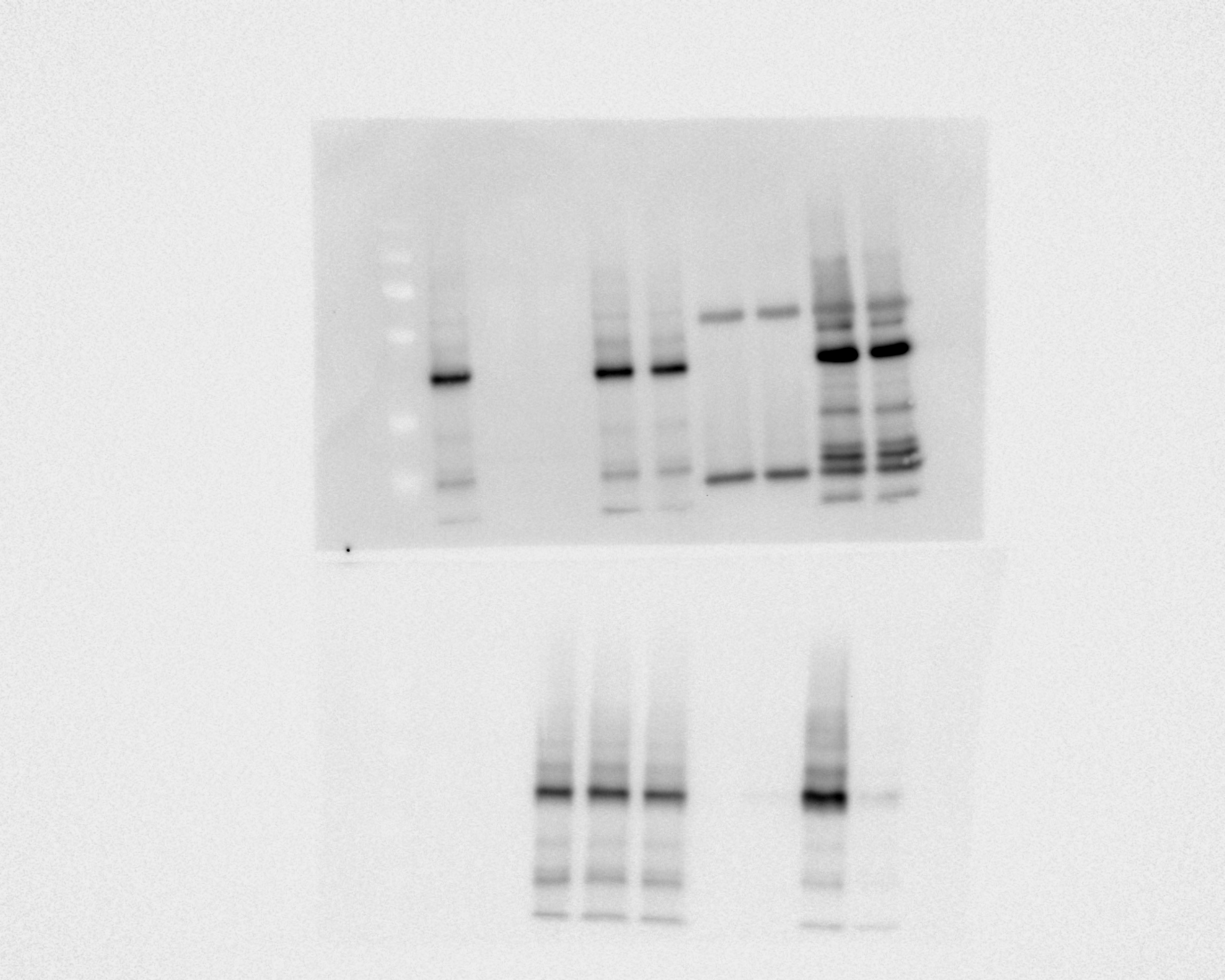

Supplement: Figure 4—source data 4. [file elife-89951-fig4-data4.zip › Figure 4-source data 4/WB_and_IP_Ollas-SPOP_Figure 4-source data 4/Versteeg 2022-02-23 11h18m44s 27.442s(Chemiluminescence).jpg]

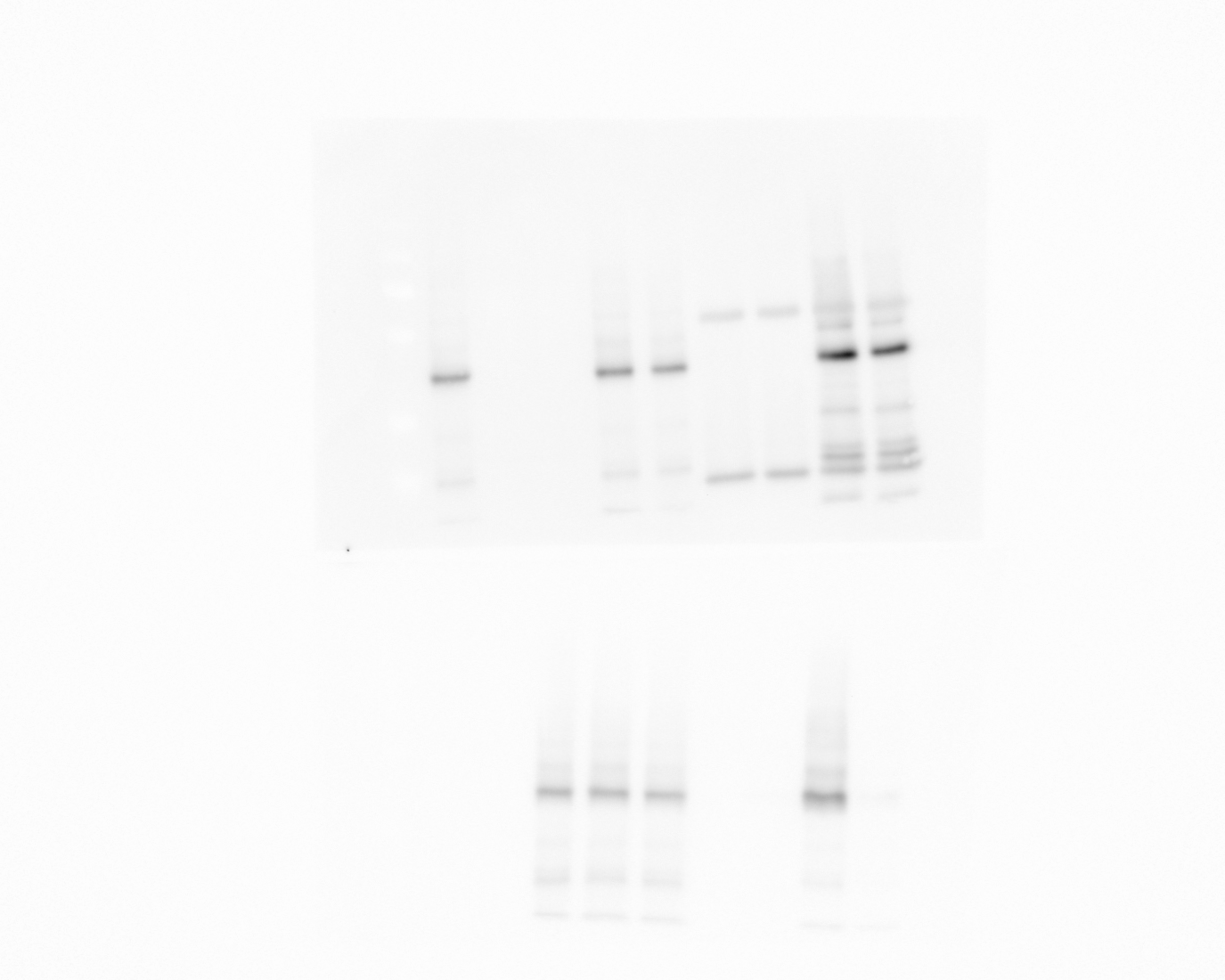

Supplement: Figure 4—source data 4. [file elife-89951-fig4-data4.zip › Figure 4-source data 4/WB_and_IP_Ollas-SPOP_Figure 4-source data 4/Versteeg 2022-02-23 11h18m44s 27.442s(Chemiluminescence).raw16.tif]

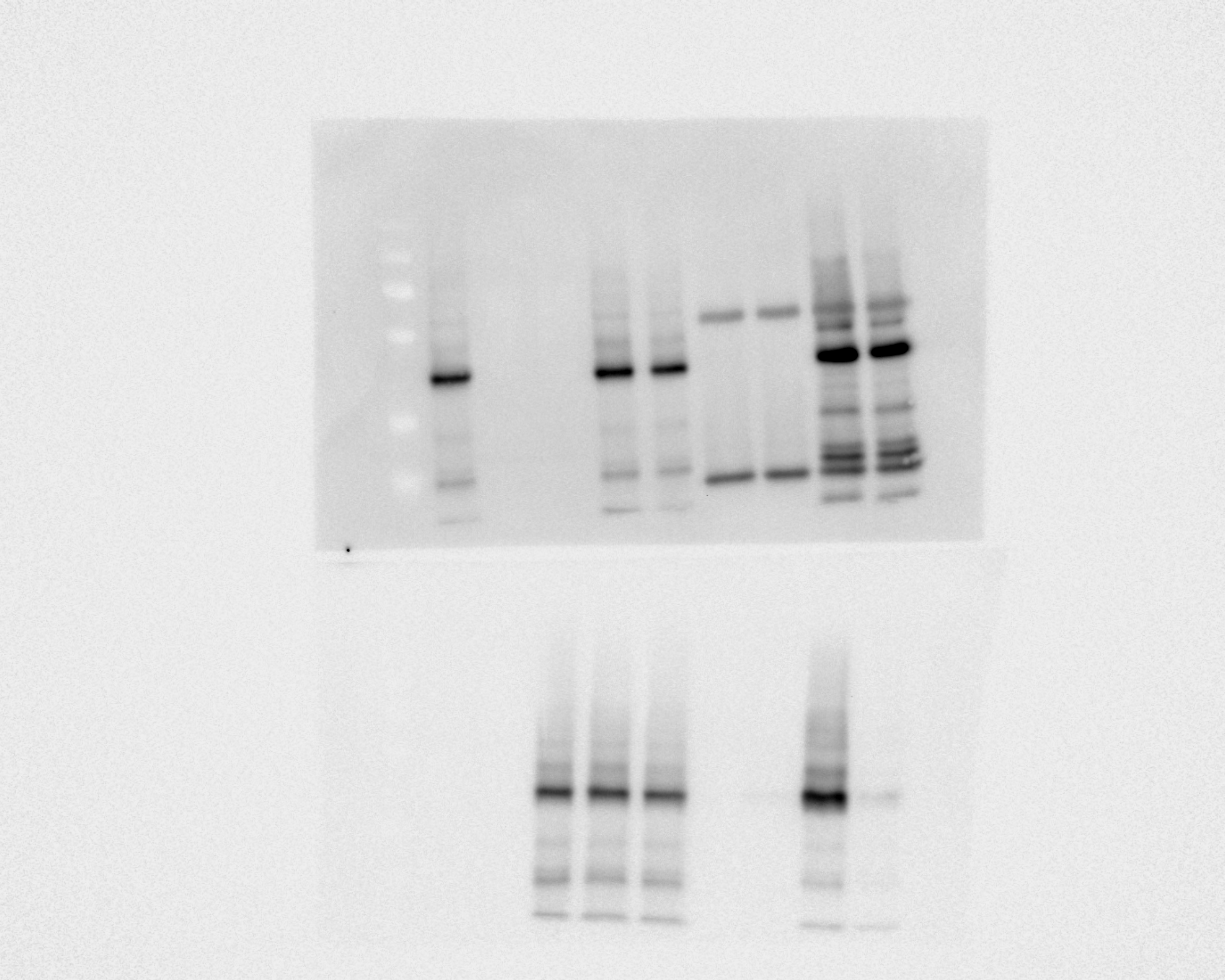

Supplement: Figure 4—source data 4. [file elife-89951-fig4-data4.zip › Figure 4-source data 4/WB_and_IP_Ollas-SPOP_Figure 4-source data 4/Versteeg 2022-02-23 11h18m44s 27.442s(Chemiluminescence).tif]

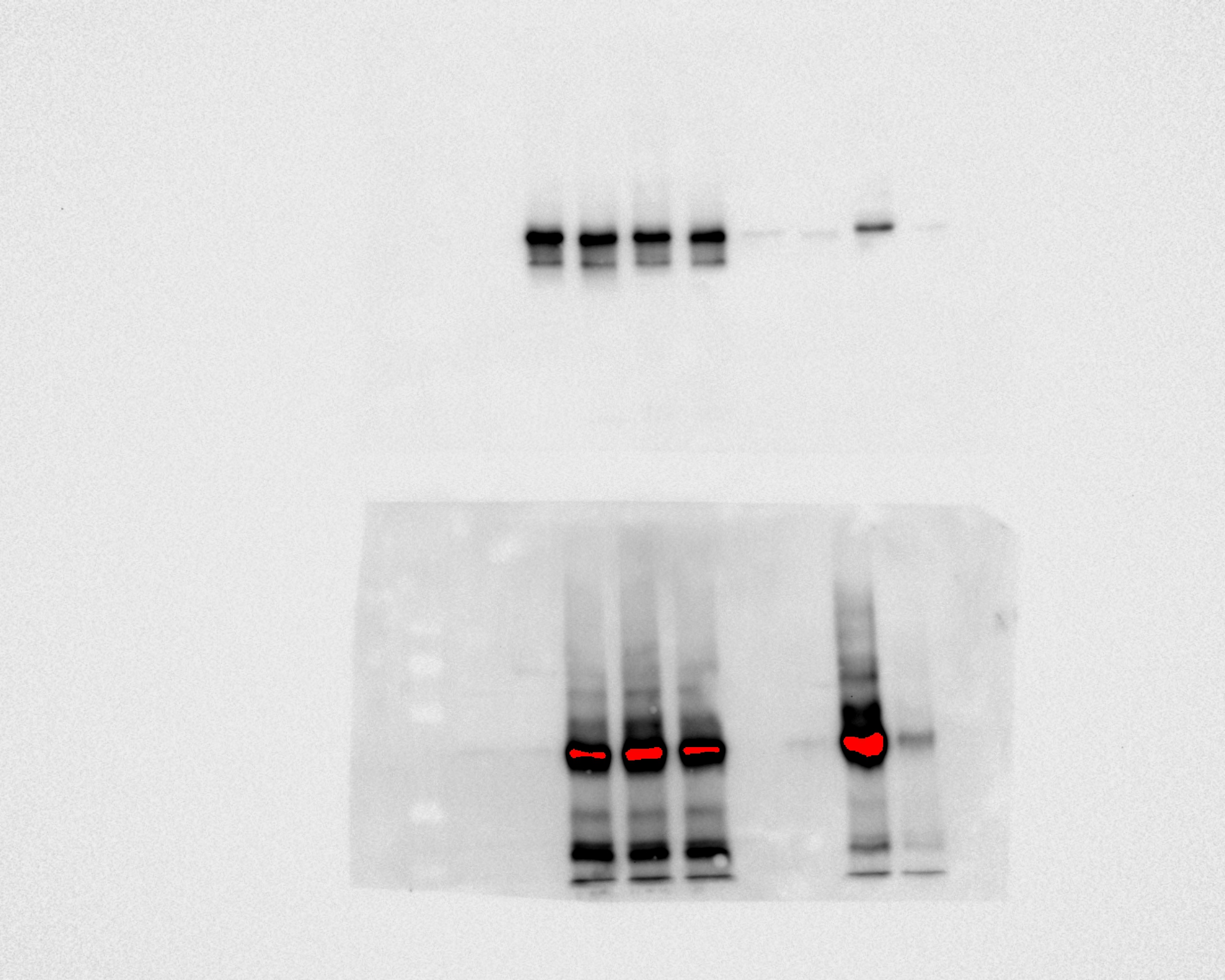

Supplement: Figure 4—source data 4. [file elife-89951-fig4-data4.zip › Figure 4-source data 4/WB_MYC-IRF1_Figure 4-source data 4/Versteeg 2022-02-24 14h35m30s 155.650s(Chemiluminescence).jpg]

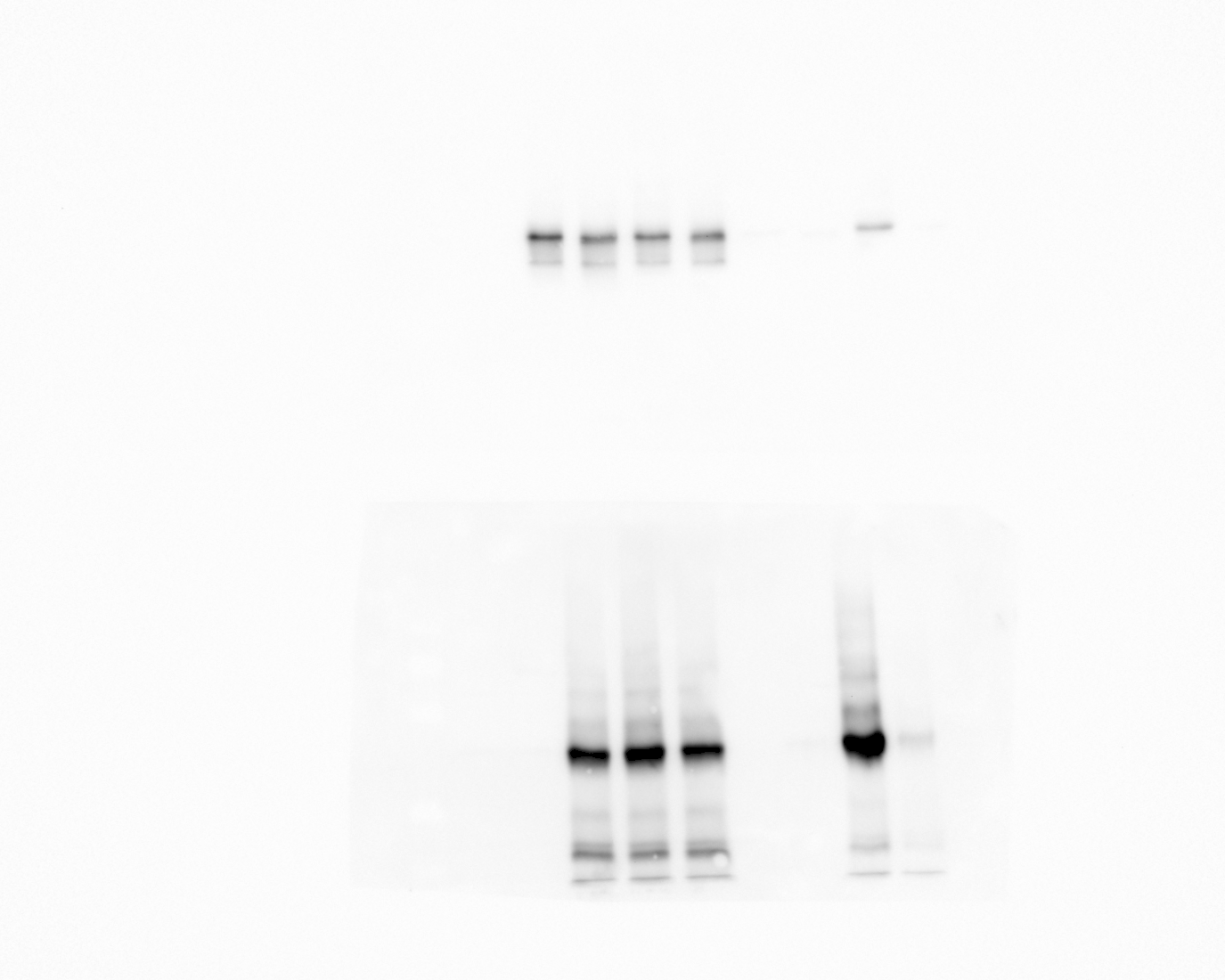

Supplement: Figure 4—source data 4. [file elife-89951-fig4-data4.zip › Figure 4-source data 4/WB_MYC-IRF1_Figure 4-source data 4/Versteeg 2022-02-24 14h35m30s 155.650s(Chemiluminescence).raw16.tif]

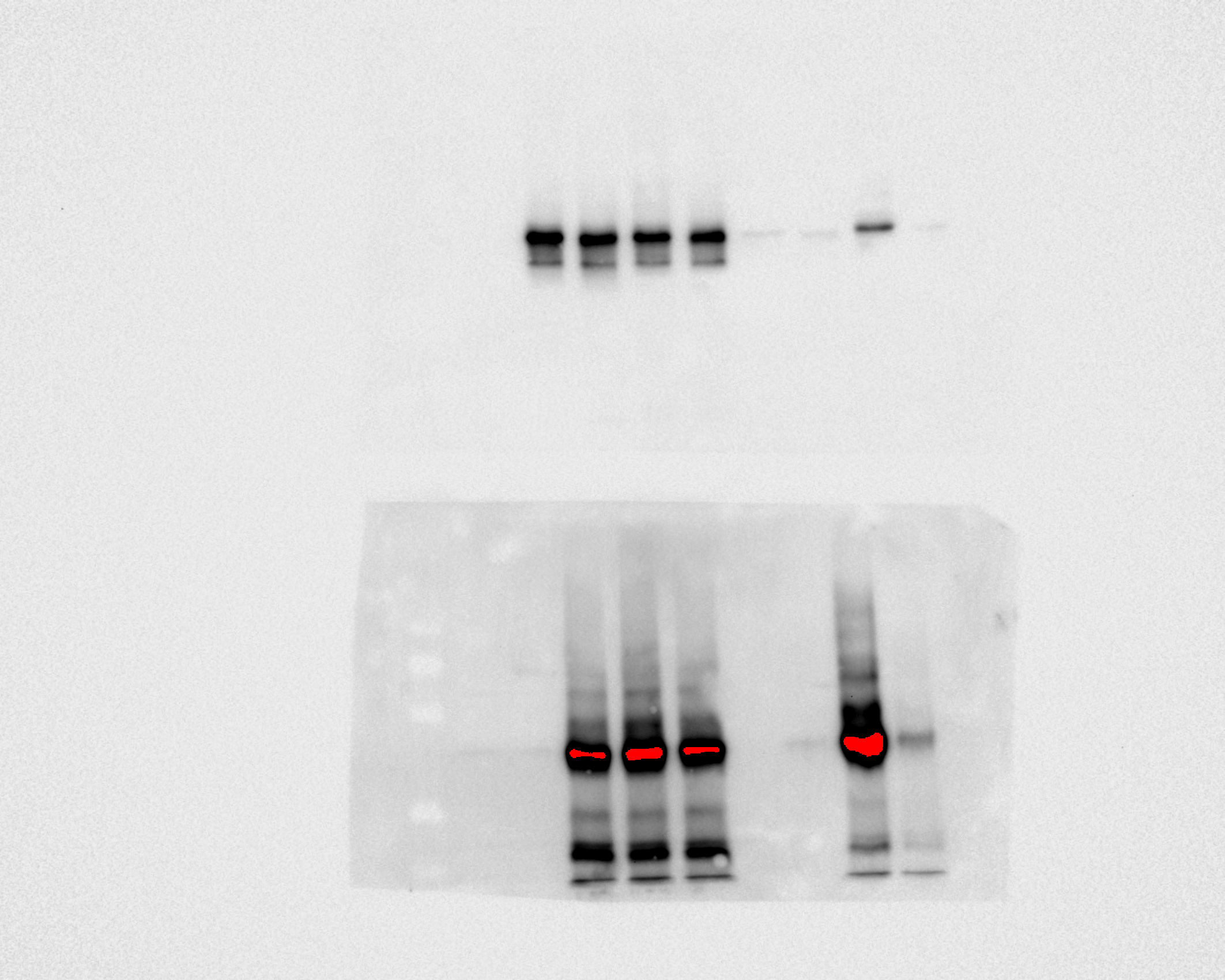

Supplement: Figure 4—source data 4. [file elife-89951-fig4-data4.zip › Figure 4-source data 4/WB_MYC-IRF1_Figure 4-source data 4/Versteeg 2022-02-24 14h35m30s 155.650s(Chemiluminescence).tif]

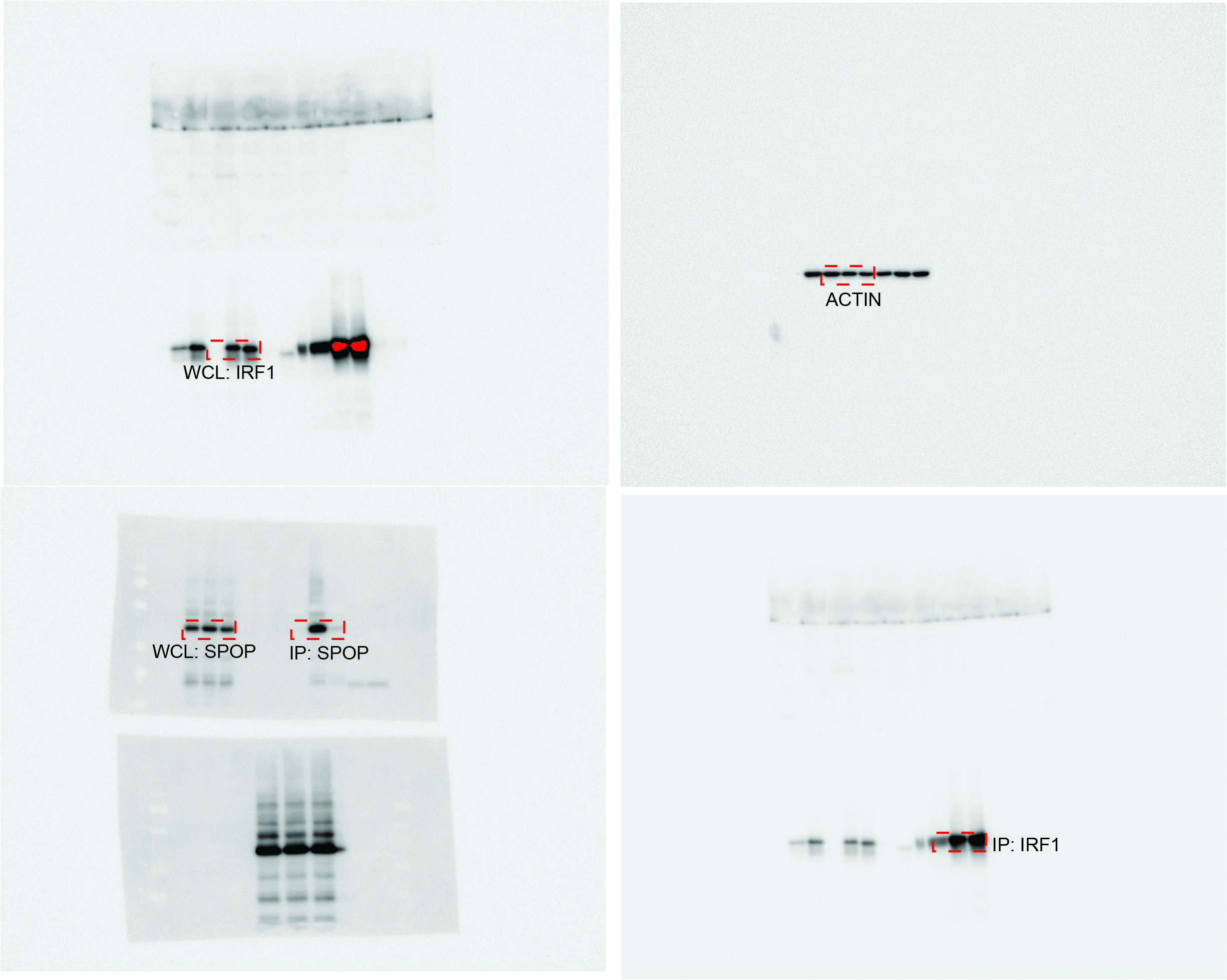

Supplement: Figure 4—source data 5. [file elife-89951-fig4-data5.zip › Figure 4-source data 5/Figure 4-source data 5.jpg]

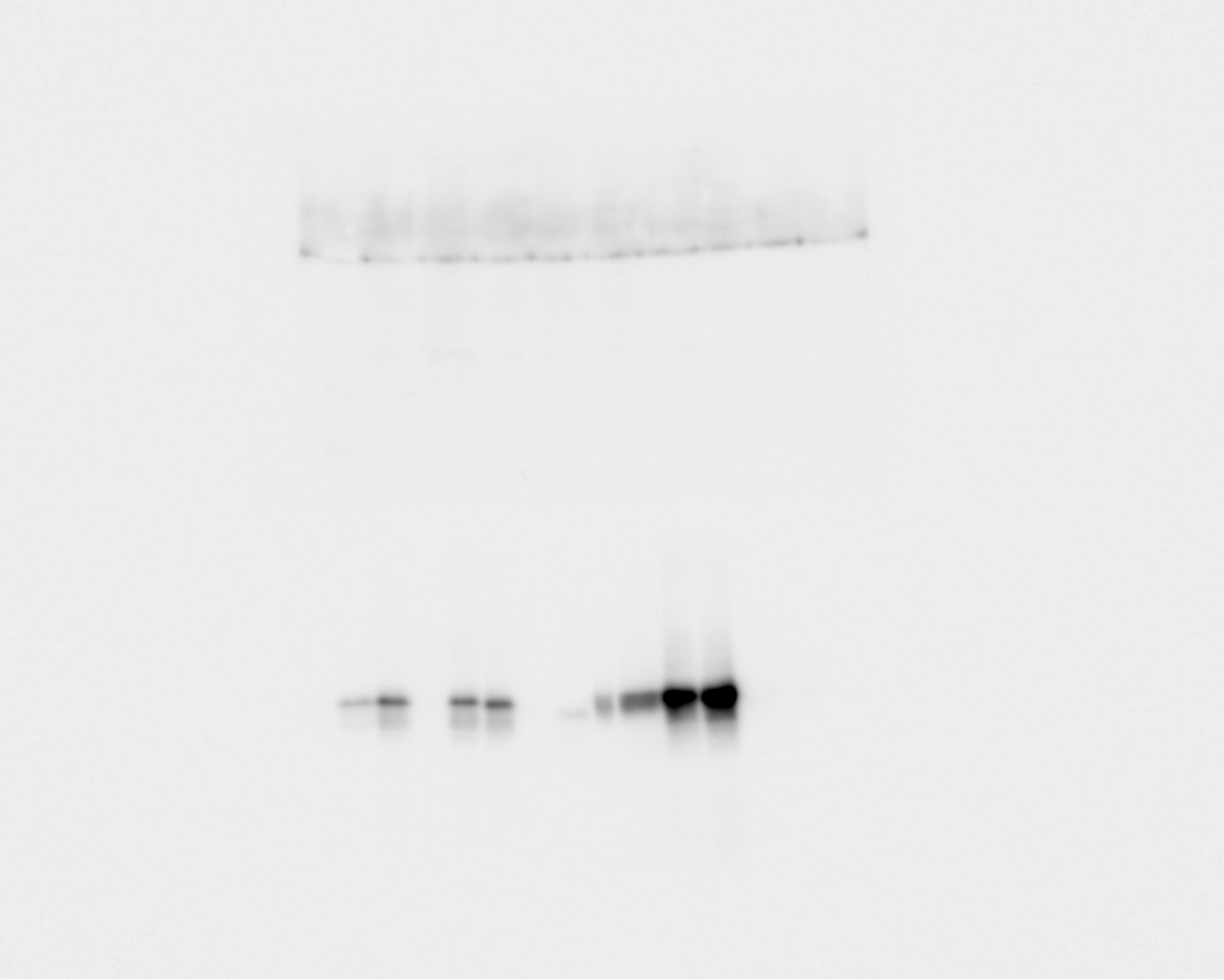

Supplement: Figure 4—source data 5. [file elife-89951-fig4-data5.zip › Figure 4-source data 5/IP_MYC-IRF1_Figure 4-source data 5/Versteeg 2022-02-08 09h51m50s 4.105s(Chemiluminescence).jpg]

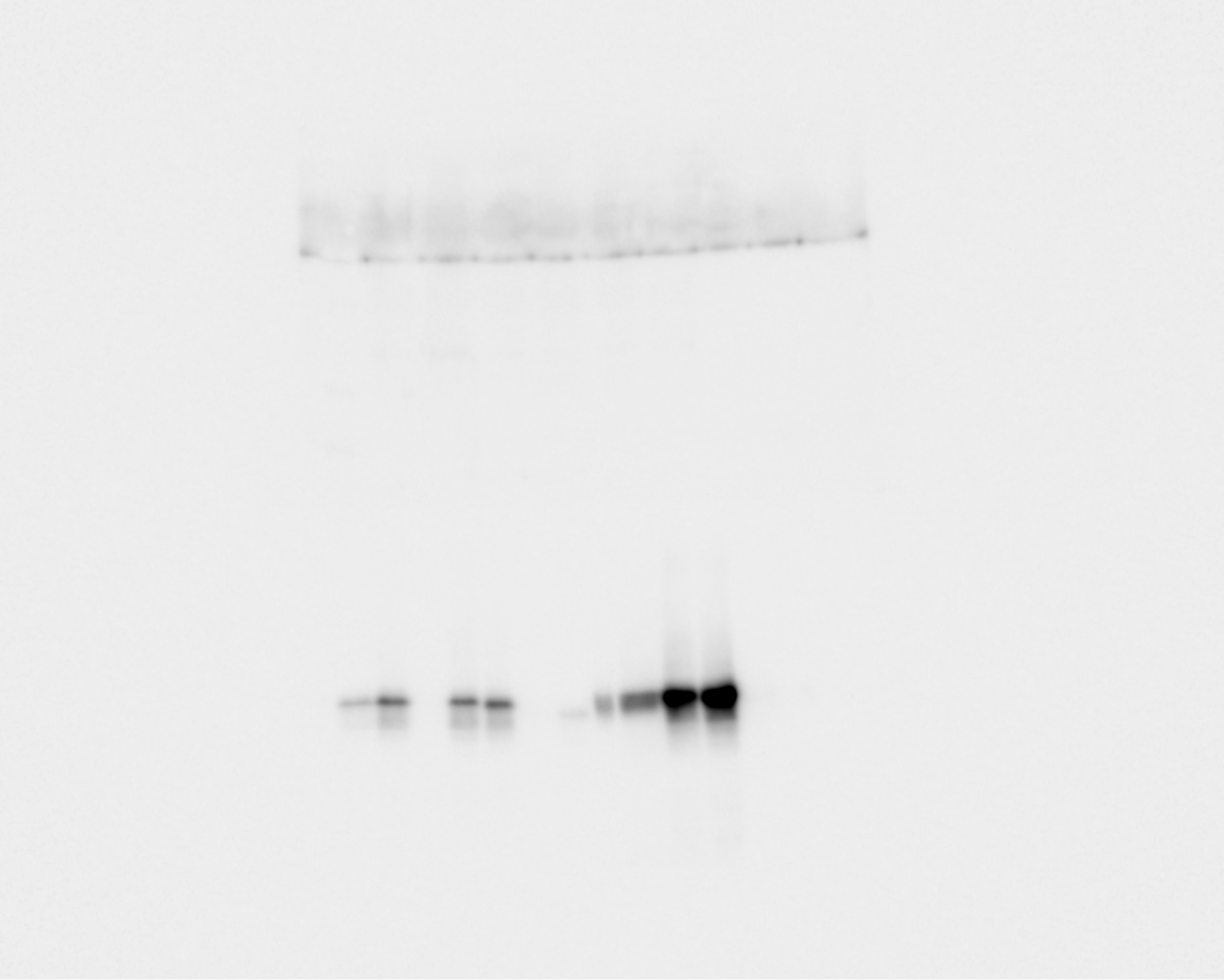

Supplement: Figure 4—source data 5. [file elife-89951-fig4-data5.zip › Figure 4-source data 5/IP_MYC-IRF1_Figure 4-source data 5/Versteeg 2022-02-08 09h51m50s 4.105s(Chemiluminescence).tif]

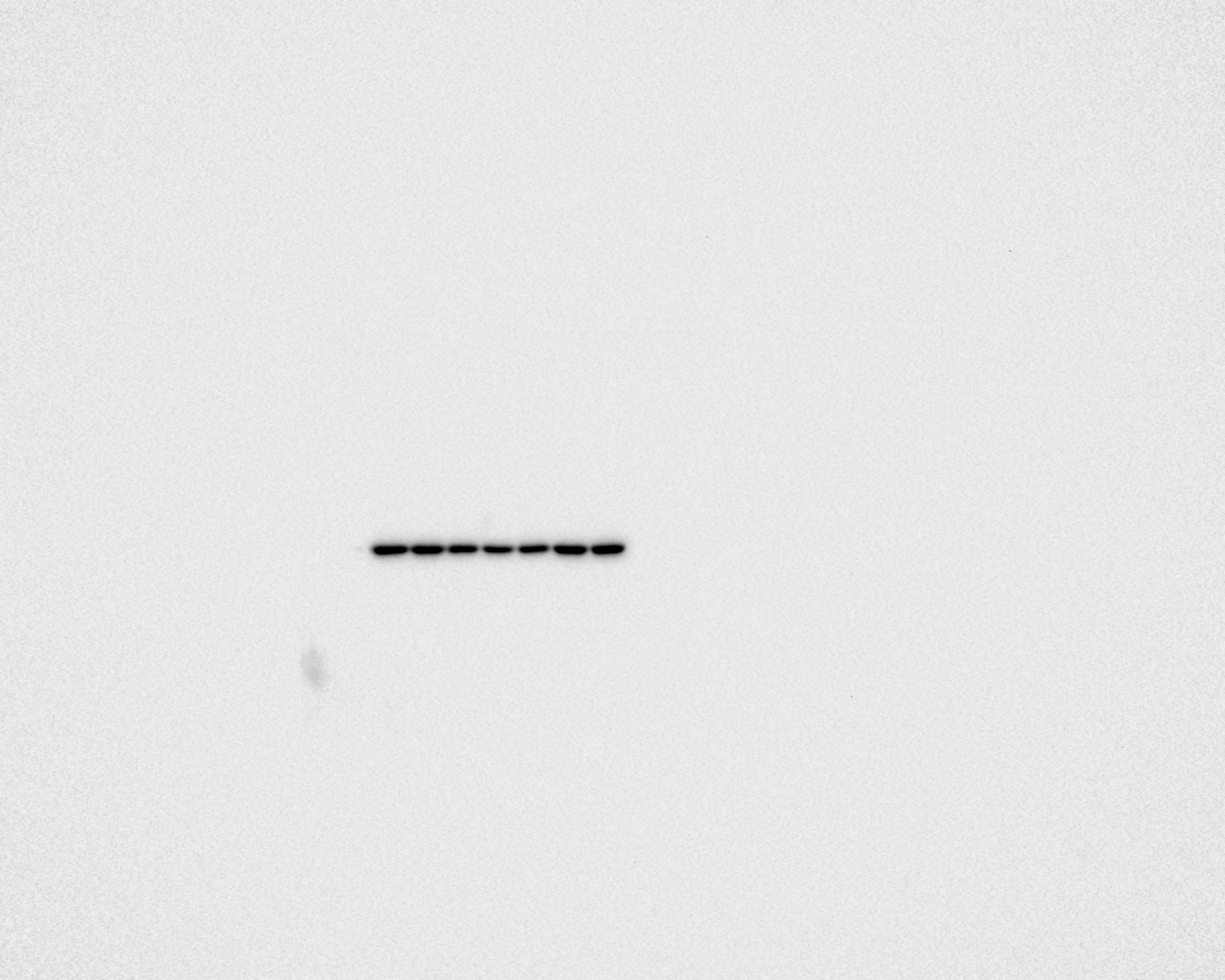

Supplement: Figure 4—source data 5. [file elife-89951-fig4-data5.zip › Figure 4-source data 5/WB_ACTIN_Figure 4-source data 5/Versteeg 2022-02-16 09h28m22s 113.734s(Chemiluminescence).jpg]

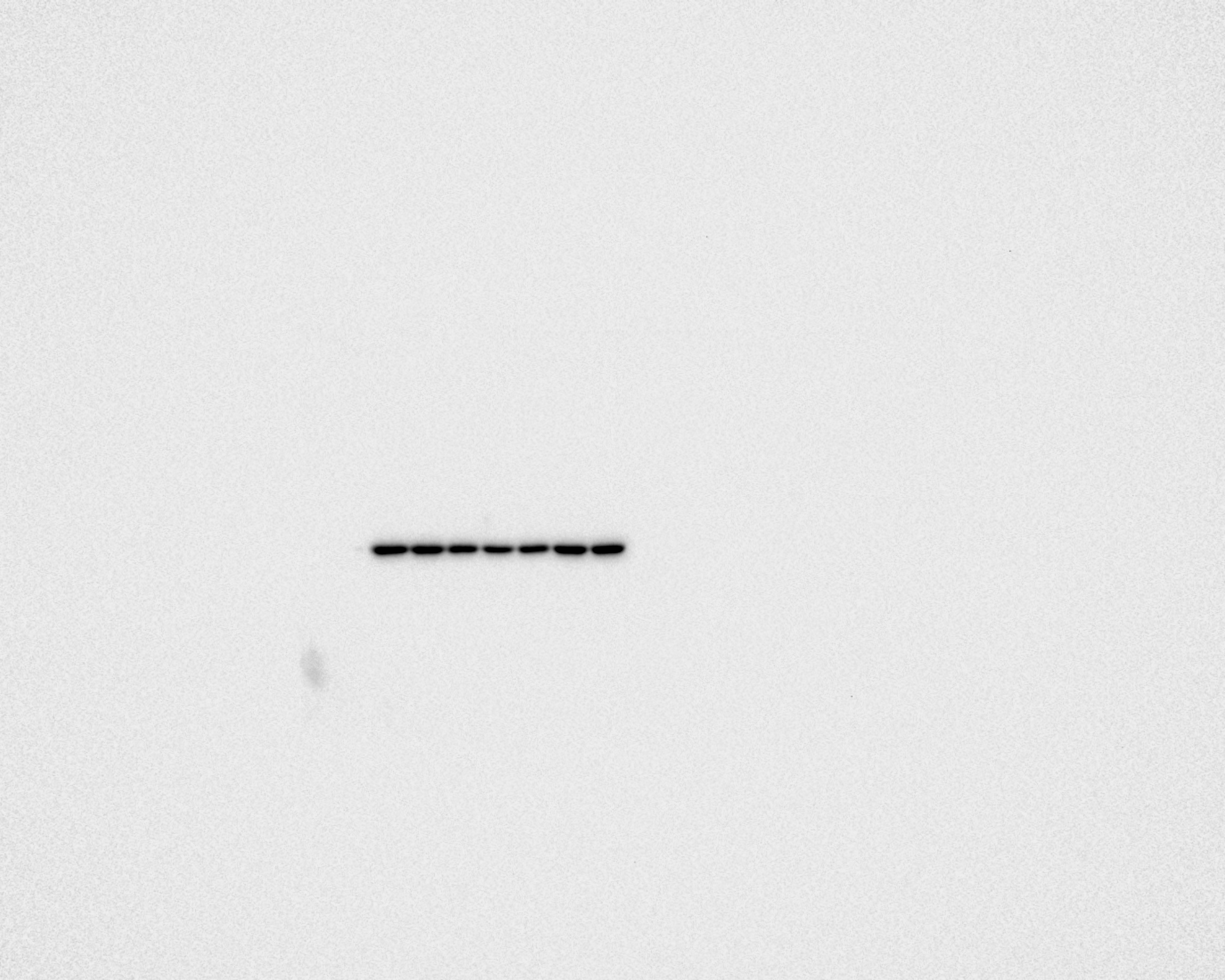

Supplement: Figure 4—source data 5. [file elife-89951-fig4-data5.zip › Figure 4-source data 5/WB_ACTIN_Figure 4-source data 5/Versteeg 2022-02-16 09h28m22s 113.734s(Chemiluminescence).tif]

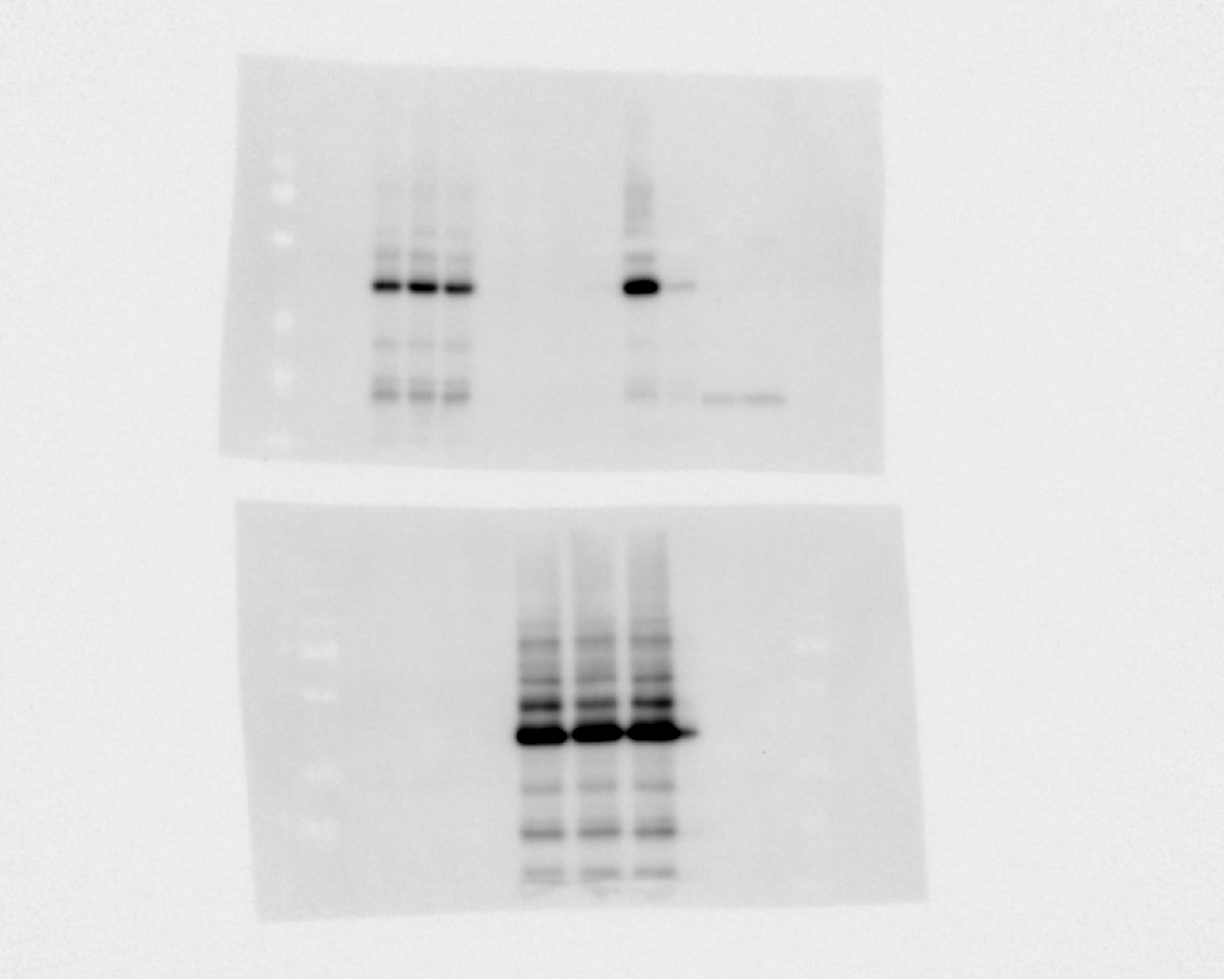

Supplement: Figure 4—source data 5. [file elife-89951-fig4-data5.zip › Figure 4-source data 5/WB_and_IP_Ollas-SPOP_Figure 4-source data 5/Versteeg 2022-02-09 11h54m23s 5.000s(Chemiluminescence).jpg]

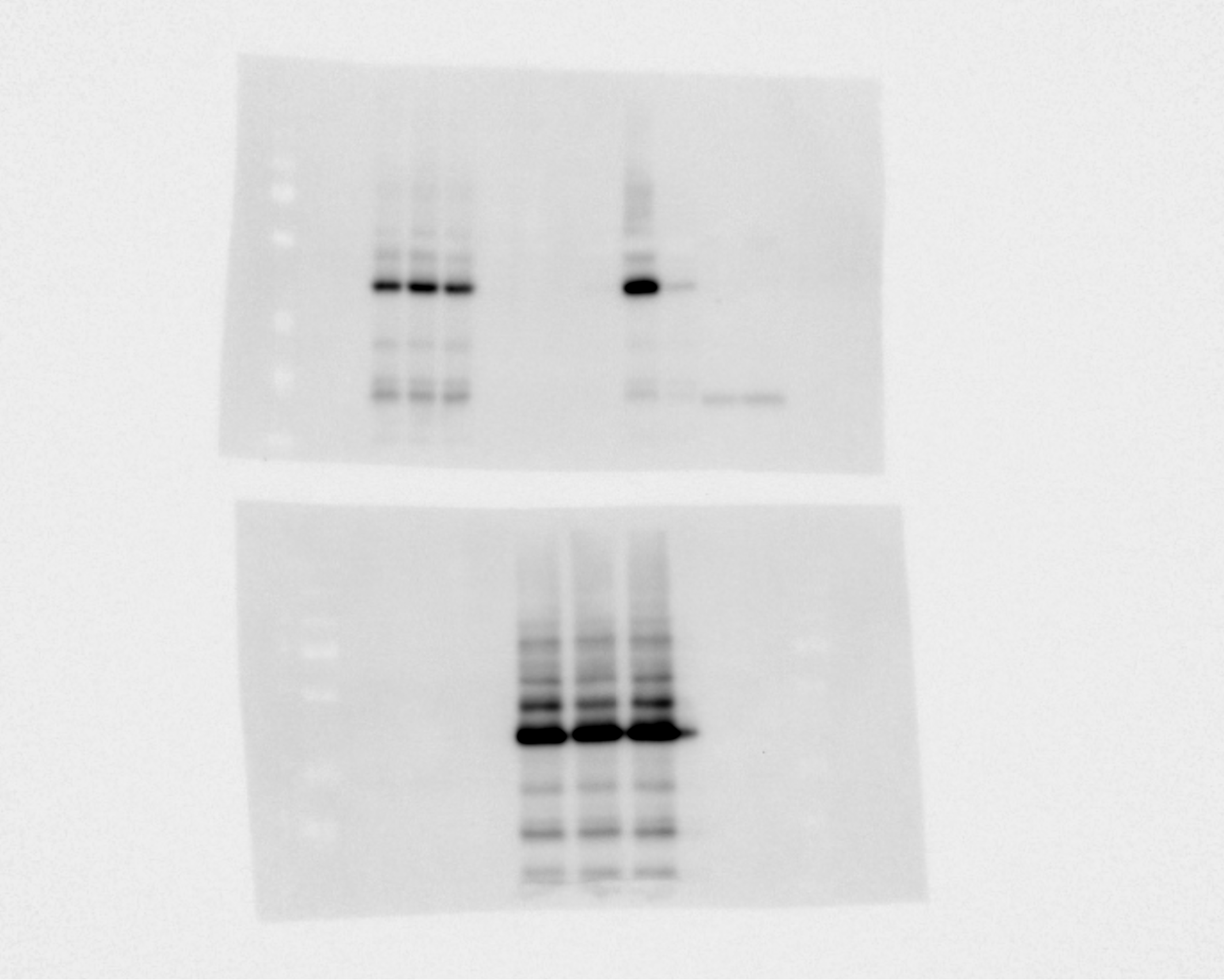

Supplement: Figure 4—source data 5. [file elife-89951-fig4-data5.zip › Figure 4-source data 5/WB_and_IP_Ollas-SPOP_Figure 4-source data 5/Versteeg 2022-02-09 11h54m23s 5.000s(Chemiluminescence).tif]

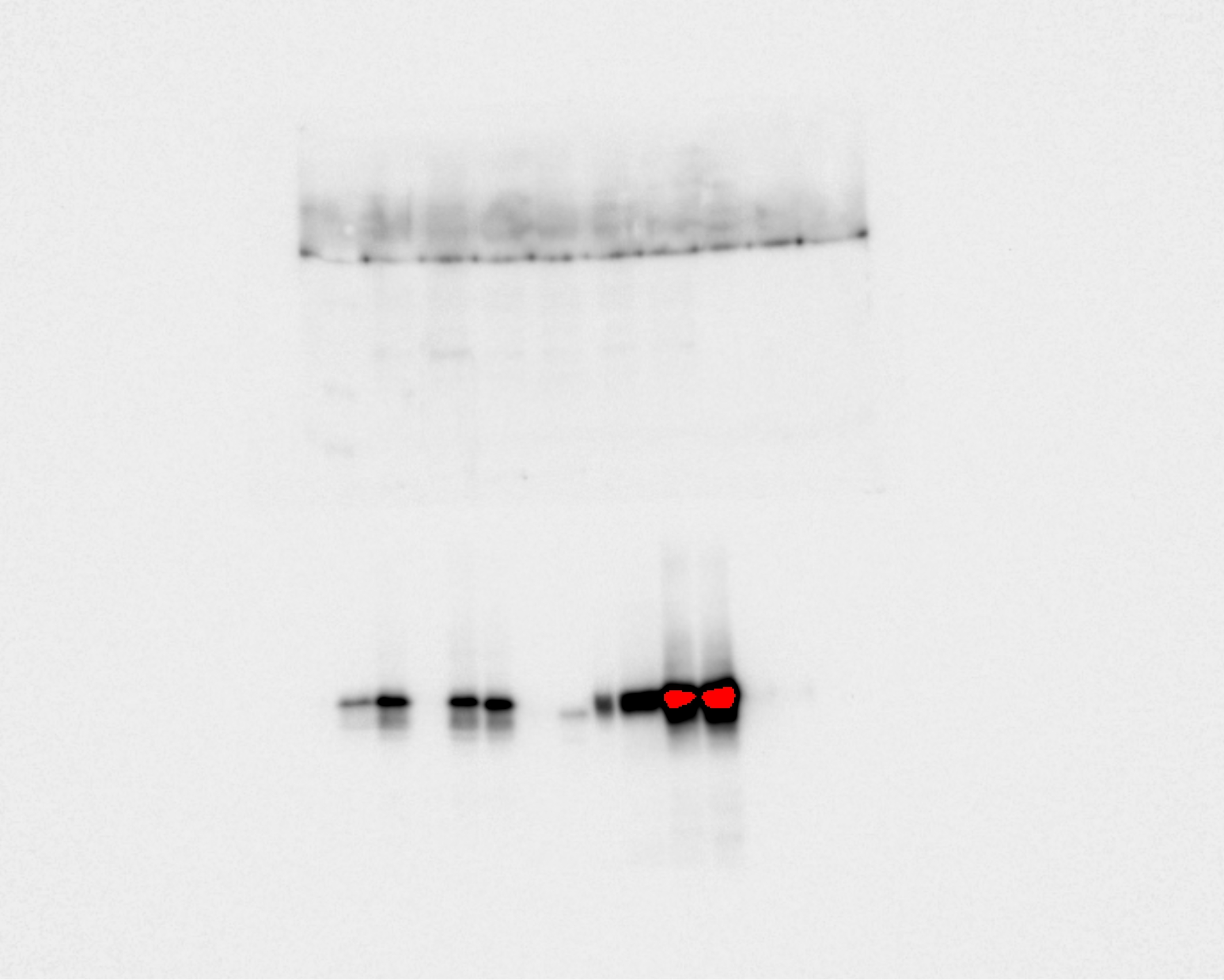

Supplement: Figure 4—source data 5. [file elife-89951-fig4-data5.zip › Figure 4-source data 5/WB_MYC-IRF1_Figure 4-source data 5/Versteeg 2022-02-08 09h52m02s 13.420s(Chemiluminescence).jpg]
