# Supplementary figures and images for: SPOP targets the immune transcription factor IRF1 for proteasomal degradation (part 2 of 2)
Source: eLife. 2023 Aug 25;12:e89951. doi: 10.7554/eLife.89951 (PMC10491434; doi:10.7554/eLife.89951)

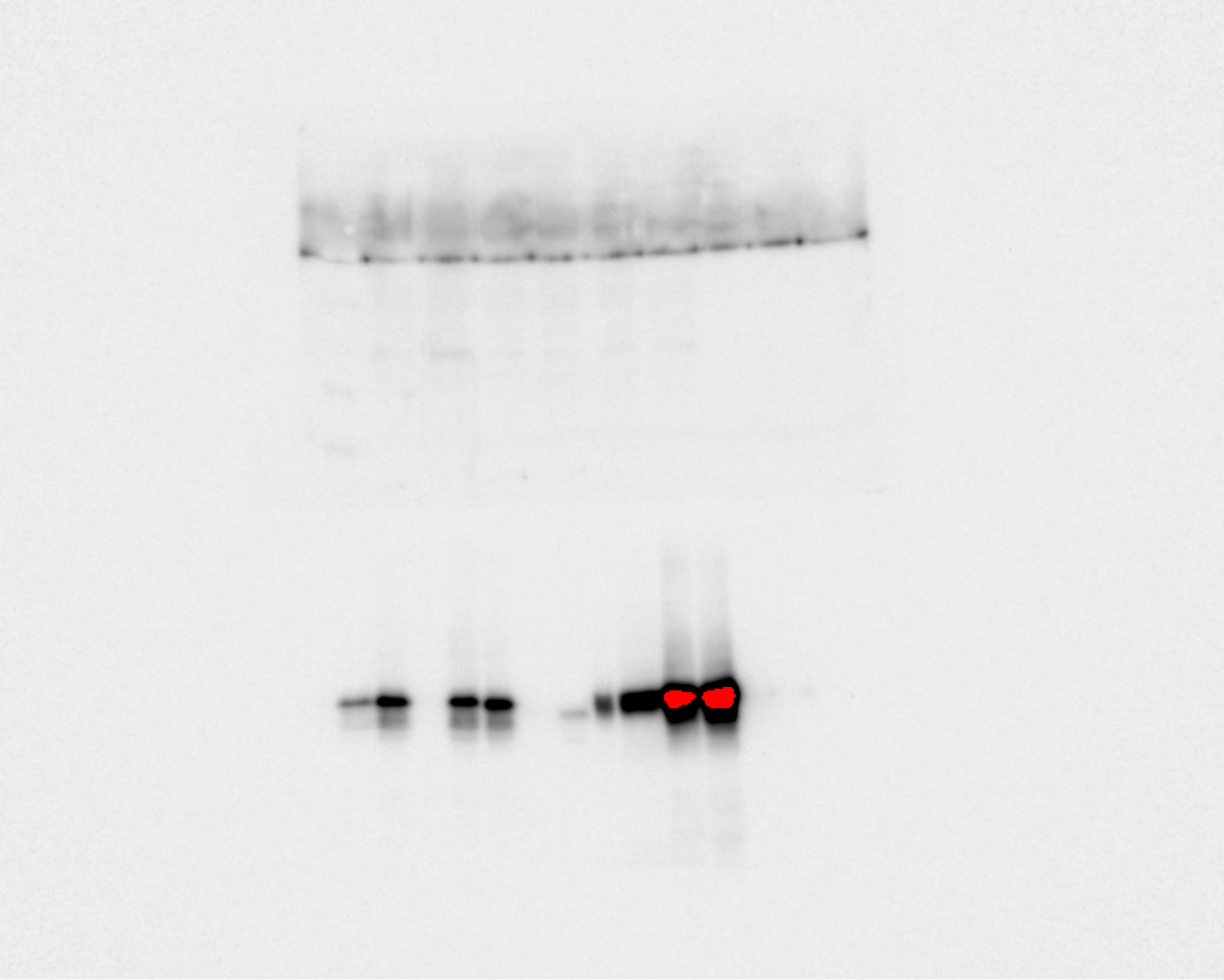

Supplement: Figure 4—source data 5. [file elife-89951-fig4-data5.zip › Figure 4-source data 5/WB_MYC-IRF1_Figure 4-source data 5/Versteeg 2022-02-08 09h52m02s 13.420s(Chemiluminescence).tif]

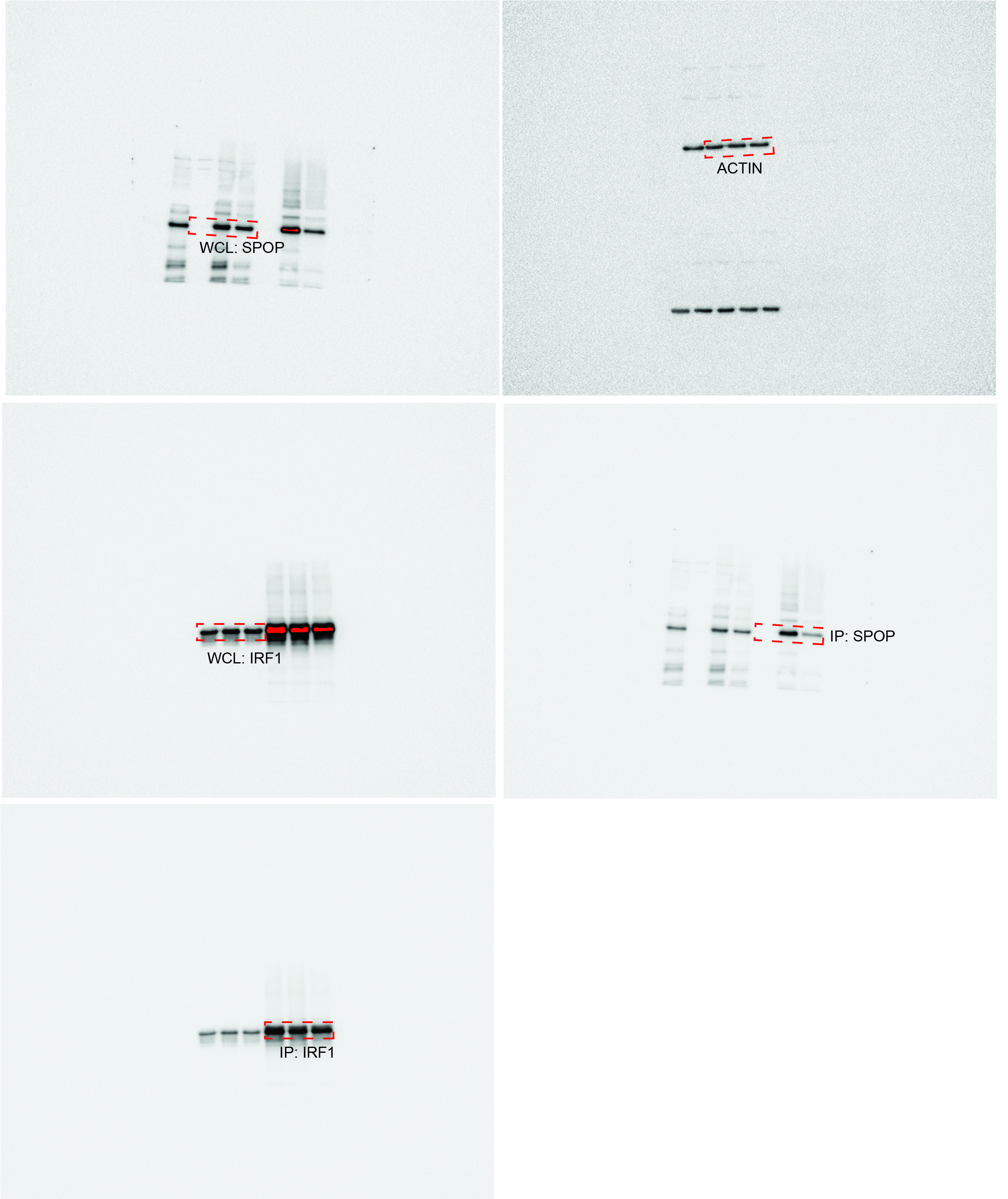

Supplement: Figure 4—source data 6. [file elife-89951-fig4-data6.zip › Figure 4-source data 6/Figure 4-source data 6.jpg]

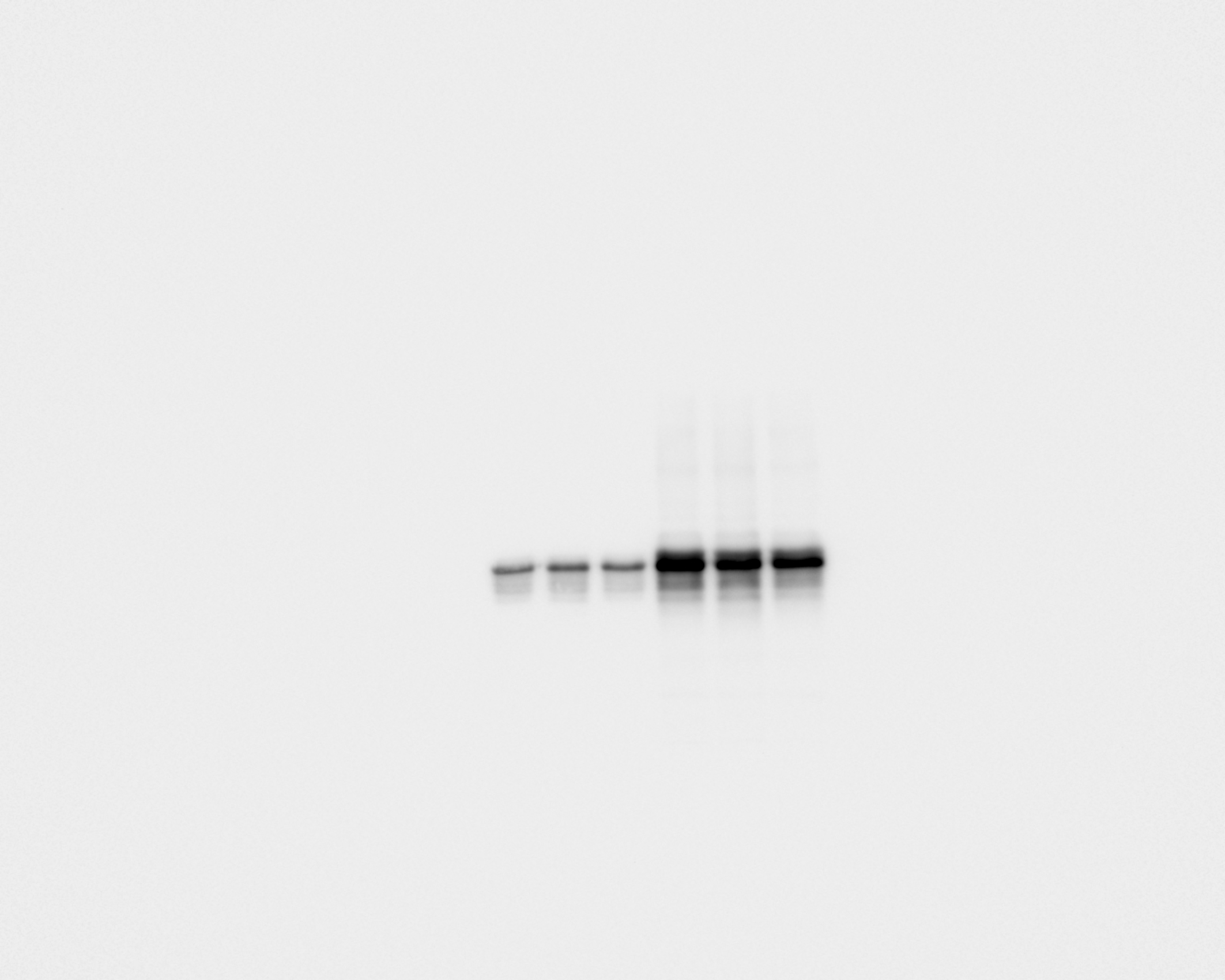

Supplement: Figure 4—source data 6. [file elife-89951-fig4-data6.zip › Figure 4-source data 6/IP_MYC-IRF1_Figure 4-source data 6/Versteeg 2022-04-22 11h25m54s 21.620s(Chemiluminescence).jpg]

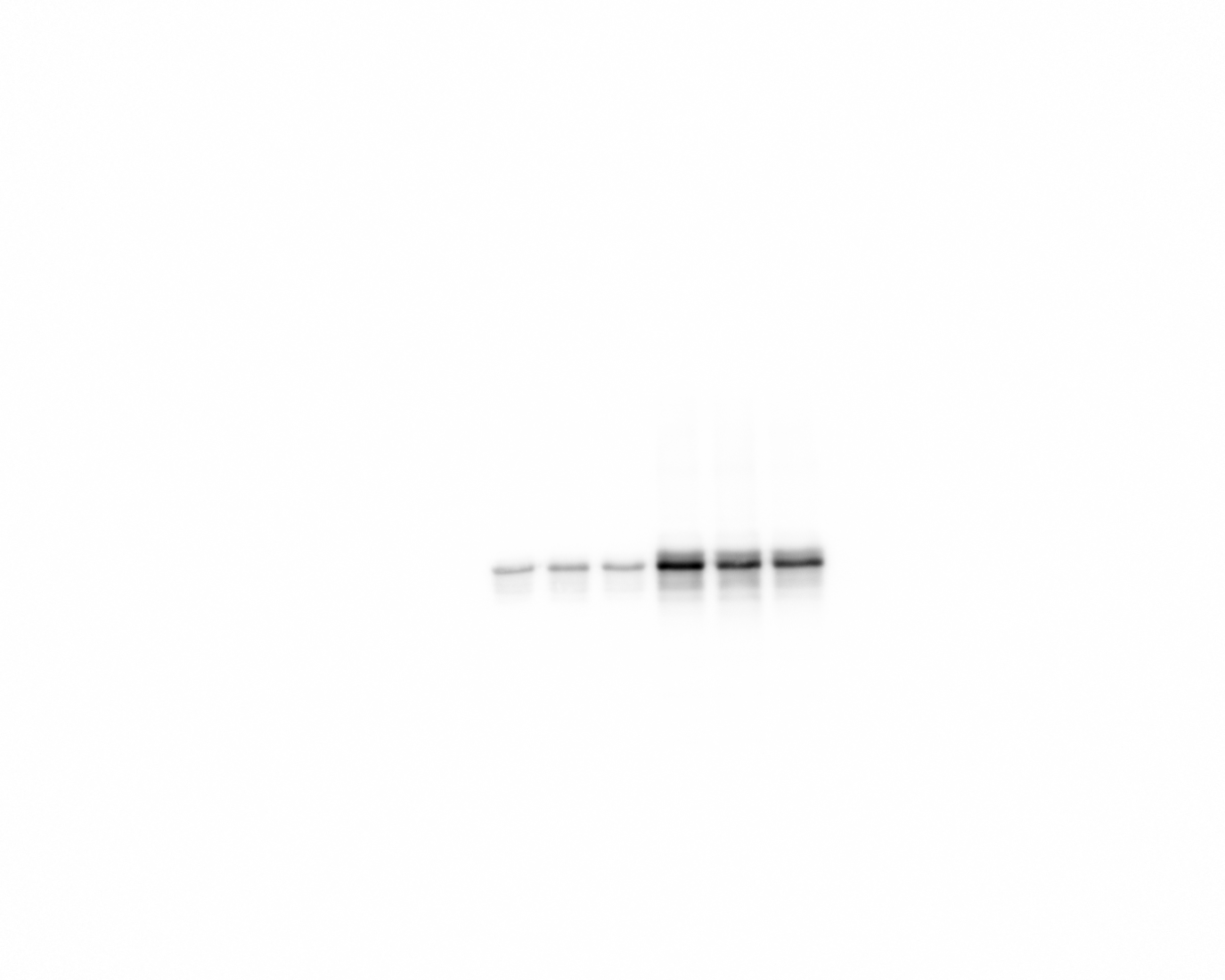

Supplement: Figure 4—source data 6. [file elife-89951-fig4-data6.zip › Figure 4-source data 6/IP_MYC-IRF1_Figure 4-source data 6/Versteeg 2022-04-22 11h25m54s 21.620s(Chemiluminescence).raw16.tif]

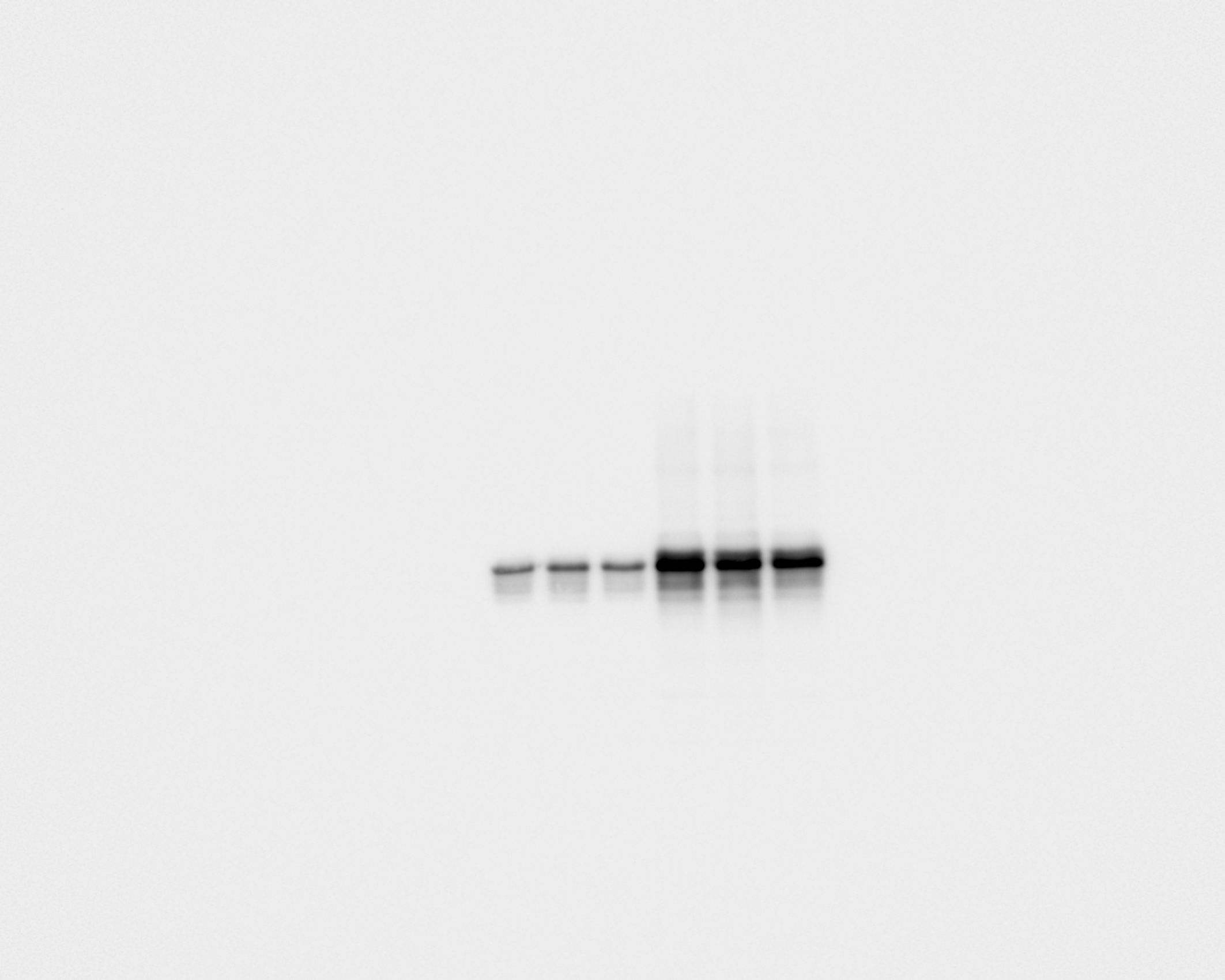

Supplement: Figure 4—source data 6. [file elife-89951-fig4-data6.zip › Figure 4-source data 6/IP_MYC-IRF1_Figure 4-source data 6/Versteeg 2022-04-22 11h25m54s 21.620s(Chemiluminescence).tif]

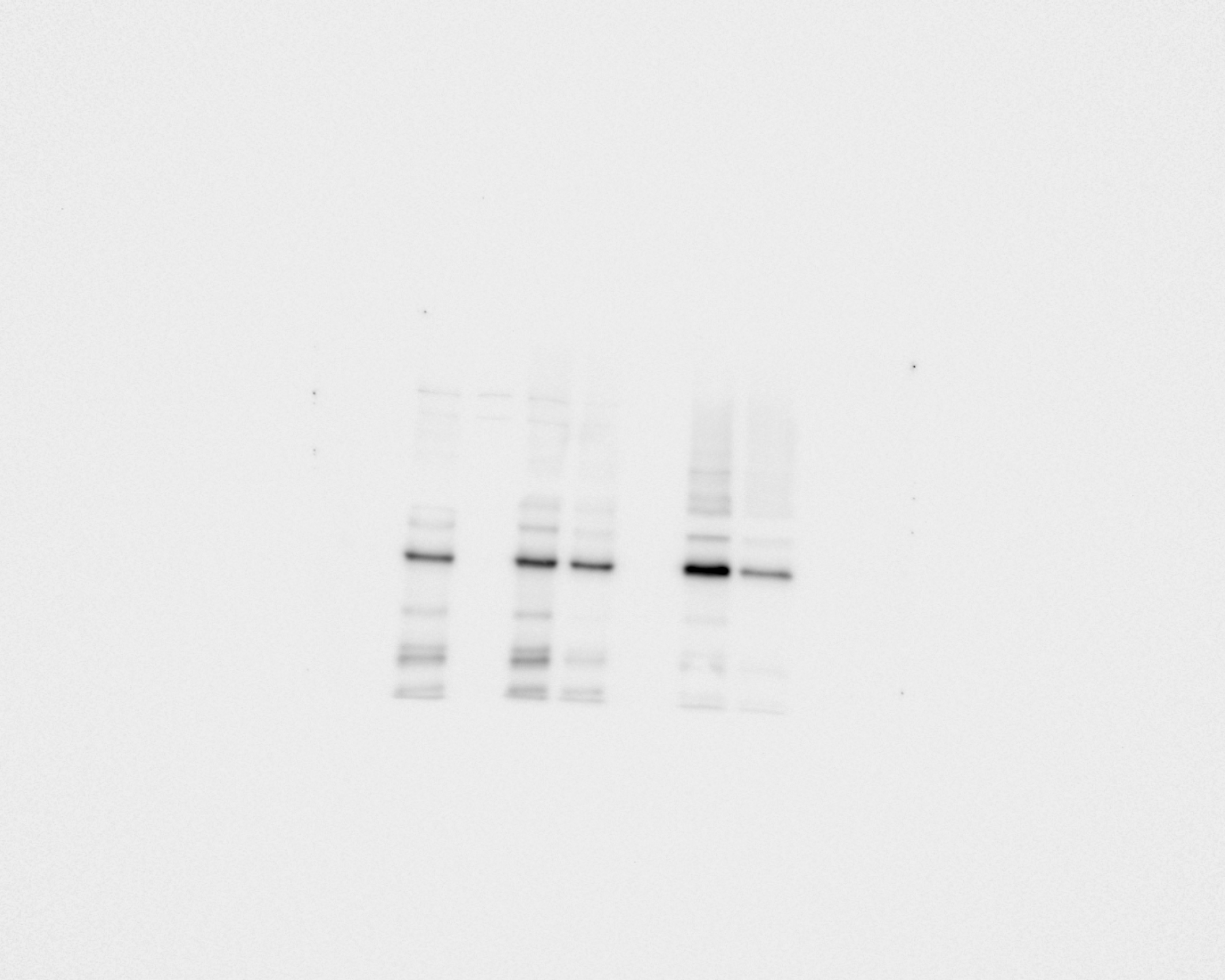

Supplement: Figure 4—source data 6. [file elife-89951-fig4-data6.zip › Figure 4-source data 6/IP_Ollas-SPOP_Figure 4-source data 6/Versteeg 2022-04-26 09h19m57s 52.550s(Chemiluminescence).jpg]

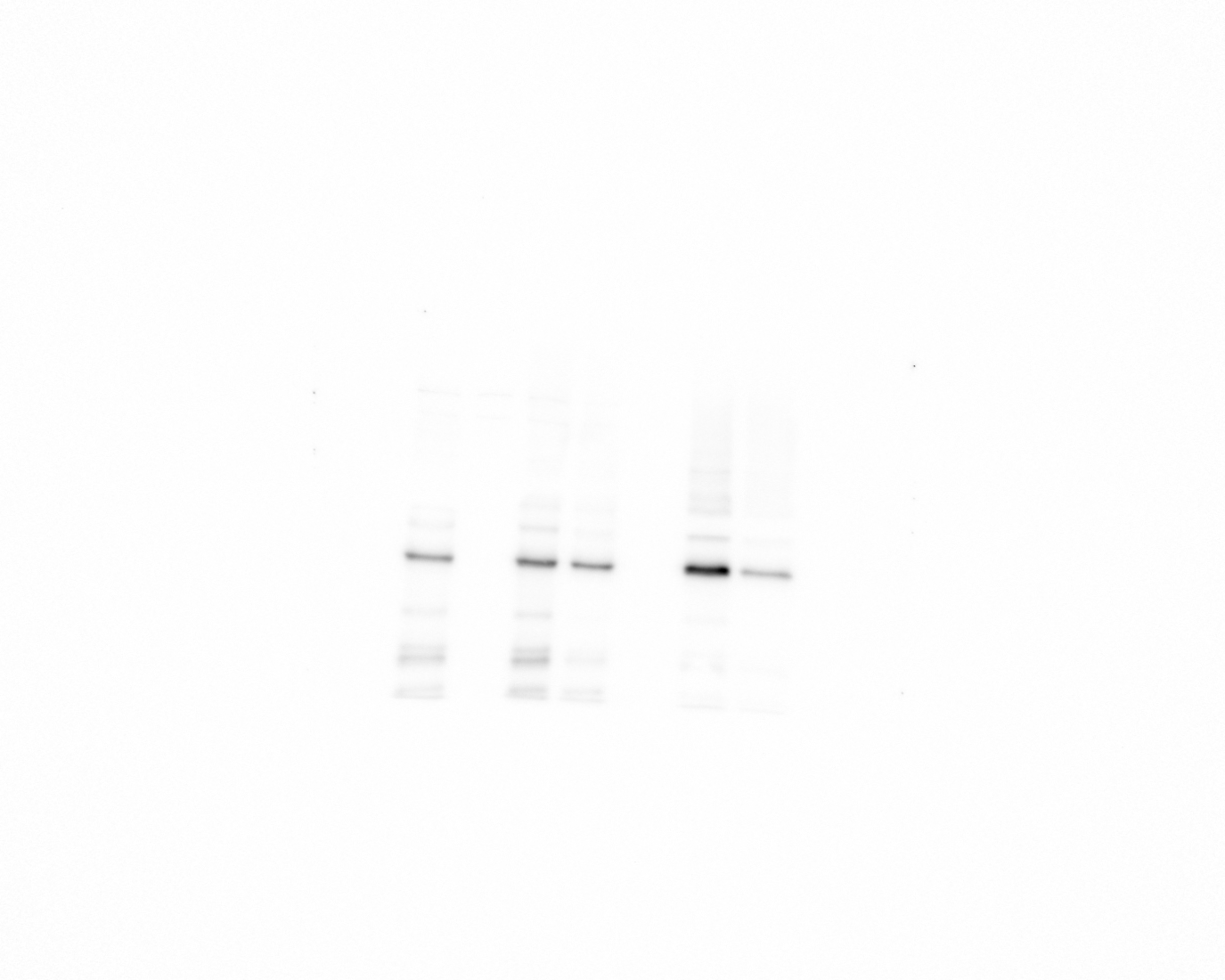

Supplement: Figure 4—source data 6. [file elife-89951-fig4-data6.zip › Figure 4-source data 6/IP_Ollas-SPOP_Figure 4-source data 6/Versteeg 2022-04-26 09h19m57s 52.550s(Chemiluminescence).raw16.tif]

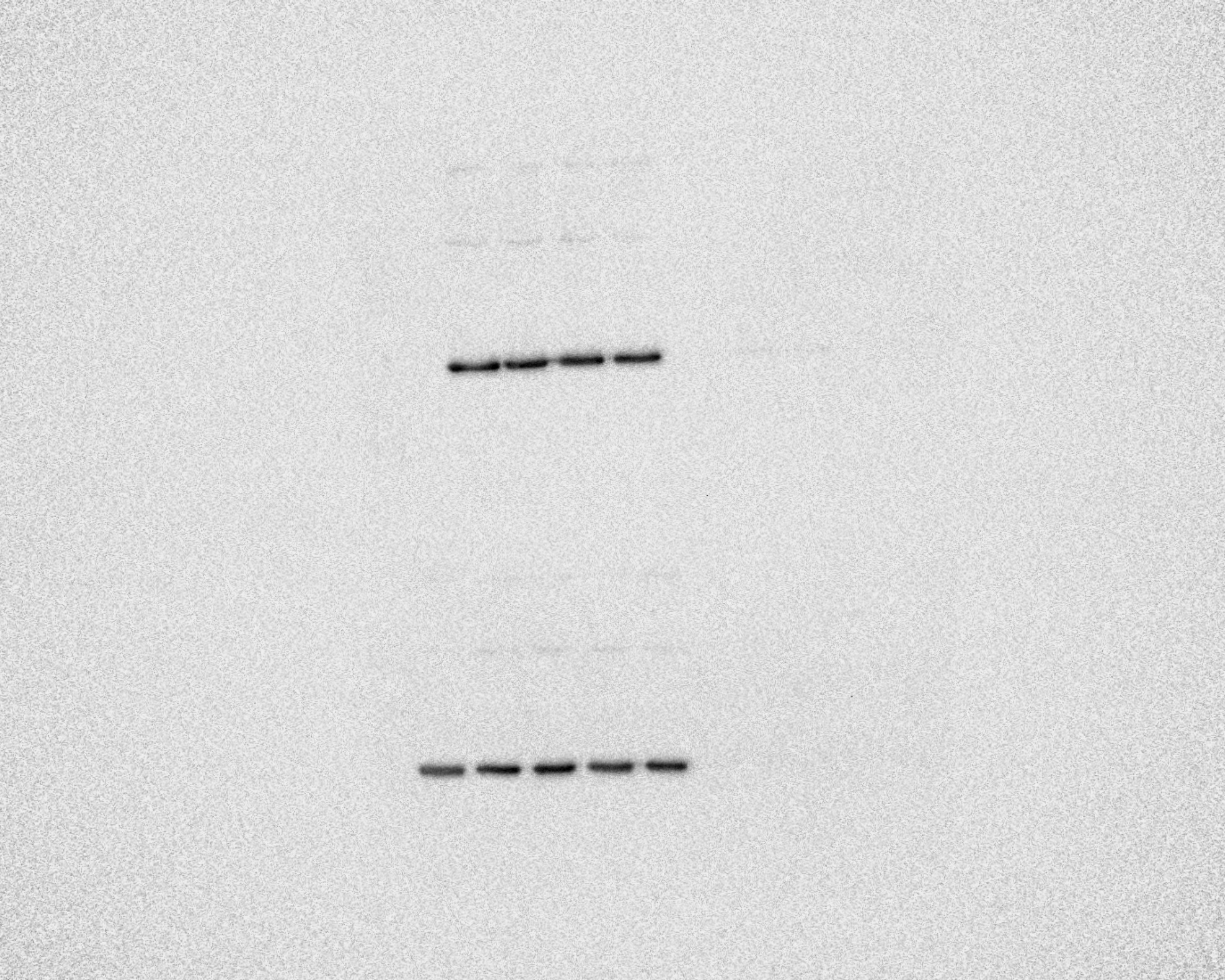

Supplement: Figure 4—source data 6. [file elife-89951-fig4-data6.zip › Figure 4-source data 6/WB_ACTIN_Figure 4-source data 6/Versteeg 2022-05-02 13h17m25s 119.944s(Chemiluminescence) ACTIN.jpg]

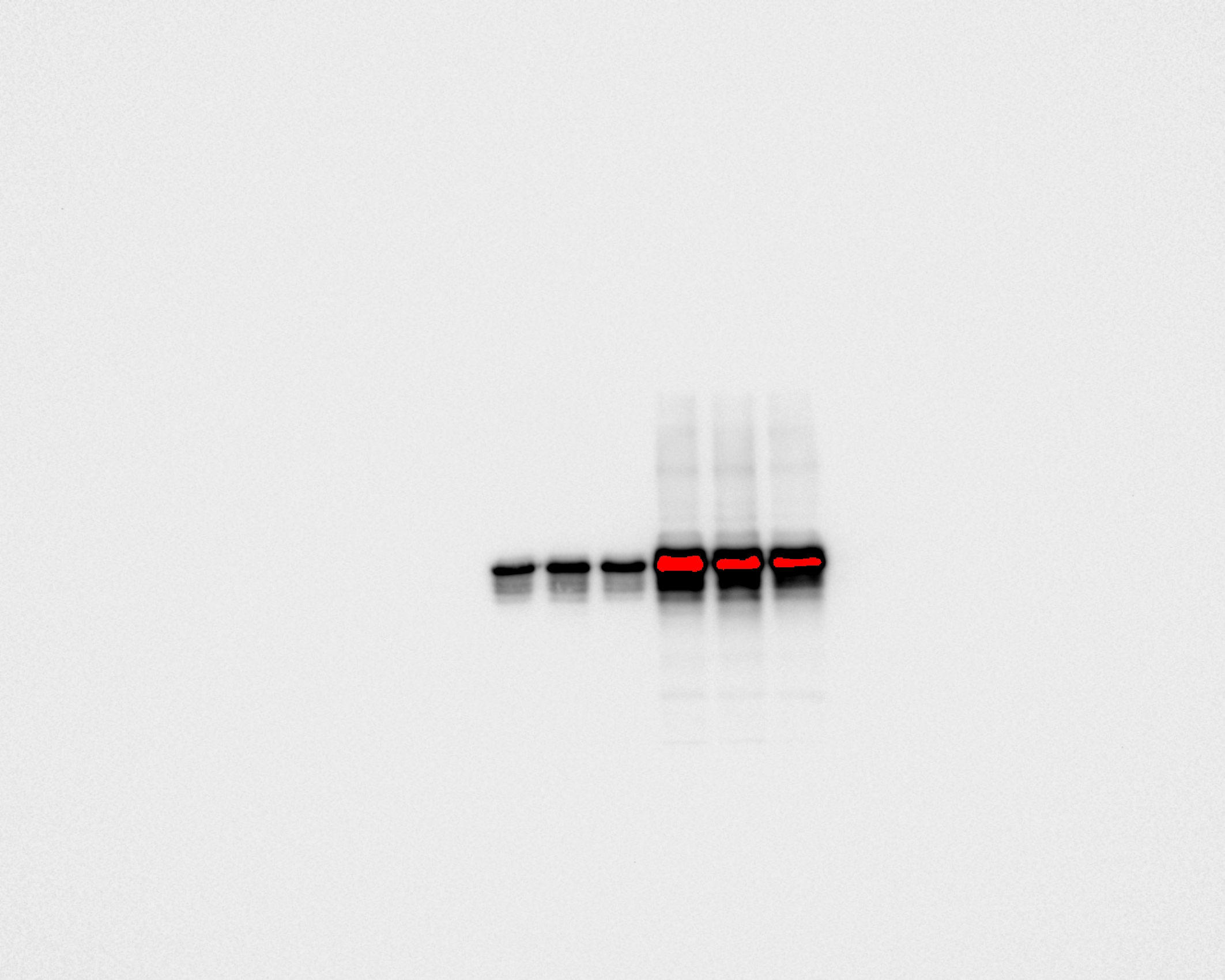

Supplement: Figure 4—source data 6. [file elife-89951-fig4-data6.zip › Figure 4-source data 6/WB_MYC-IRF1_Figure 4-source data 6/Versteeg 2022-04-22 11h27m00s 73.170s(Chemiluminescence).jpg]

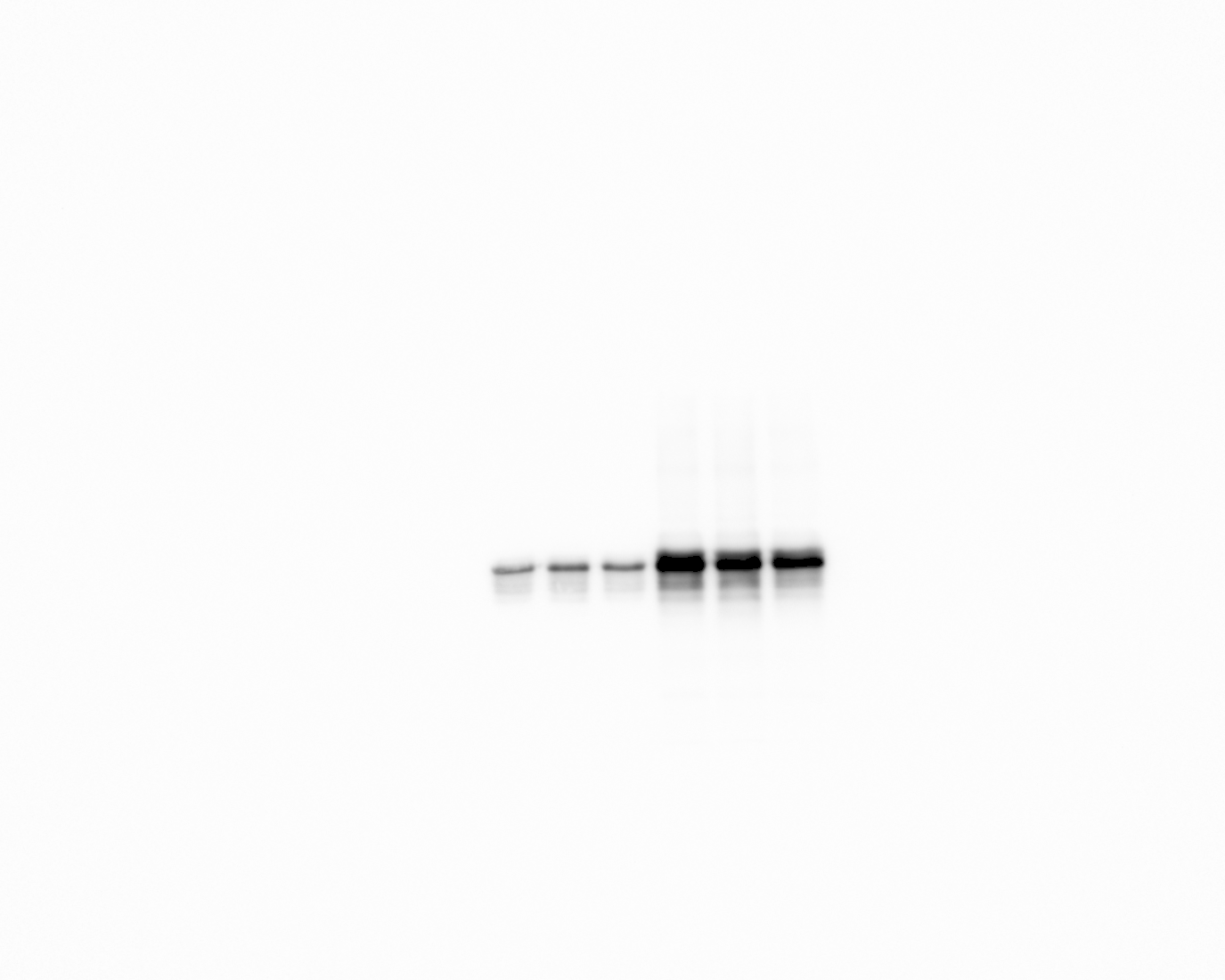

Supplement: Figure 4—source data 6. [file elife-89951-fig4-data6.zip › Figure 4-source data 6/WB_MYC-IRF1_Figure 4-source data 6/Versteeg 2022-04-22 11h27m00s 73.170s(Chemiluminescence).raw16.tif]

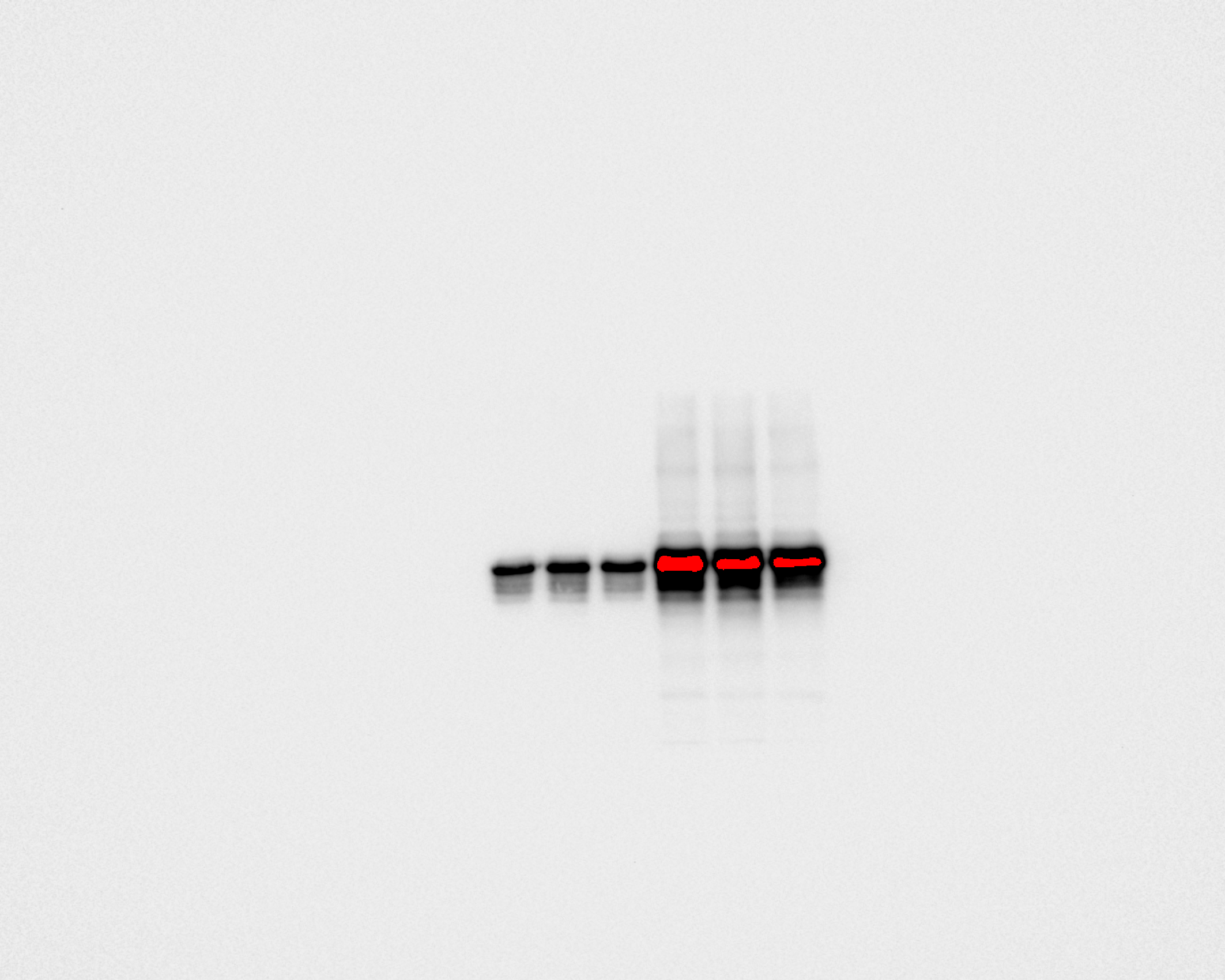

Supplement: Figure 4—source data 6. [file elife-89951-fig4-data6.zip › Figure 4-source data 6/WB_MYC-IRF1_Figure 4-source data 6/Versteeg 2022-04-22 11h27m00s 73.170s(Chemiluminescence).tif]

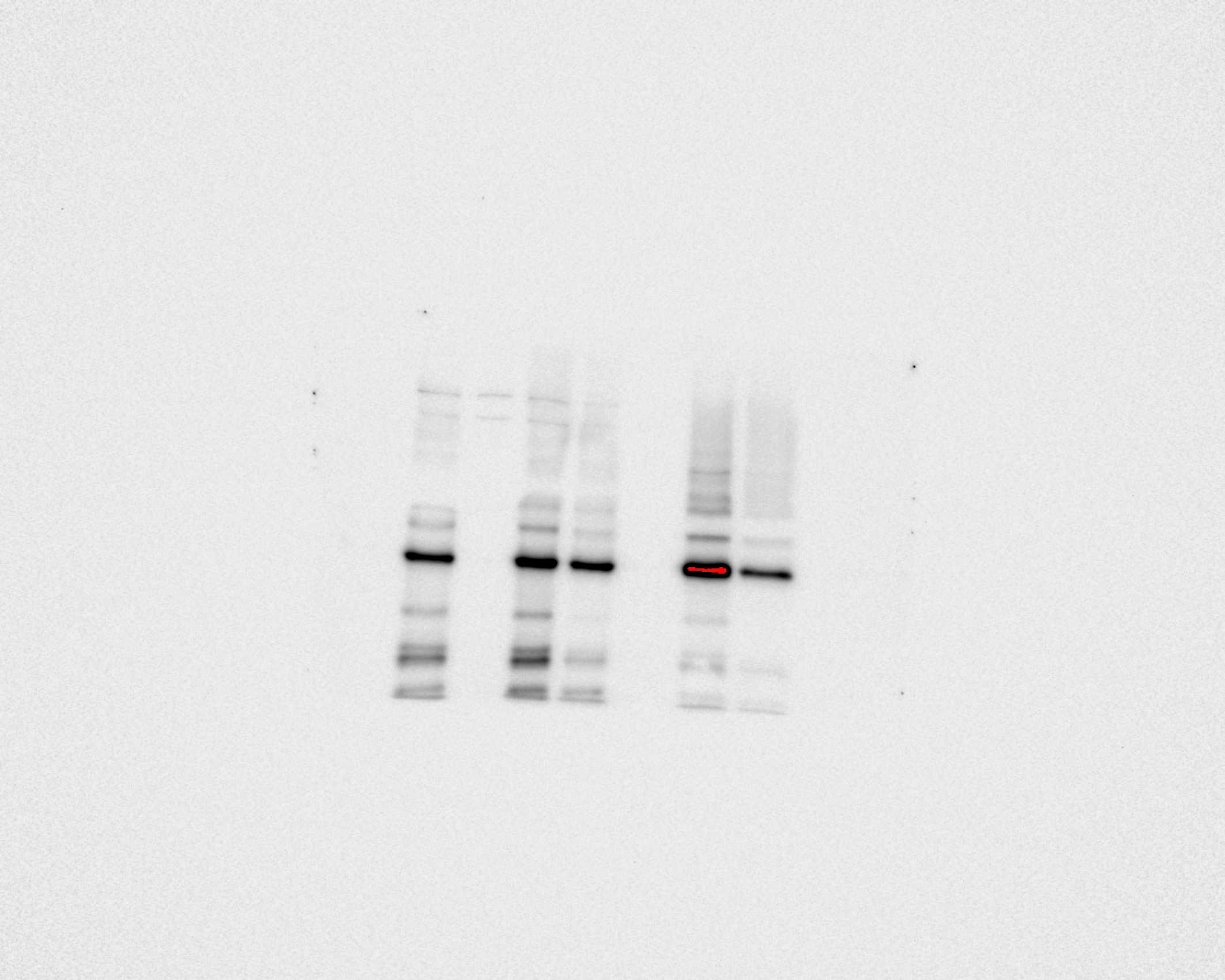

Supplement: Figure 4—source data 6. [file elife-89951-fig4-data6.zip › Figure 4-source data 6/WB_Ollas-SPOP_Figure 4-source data 6/Versteeg 2022-04-26 09h21m56s 145.340s(Chemiluminescence).jpg]

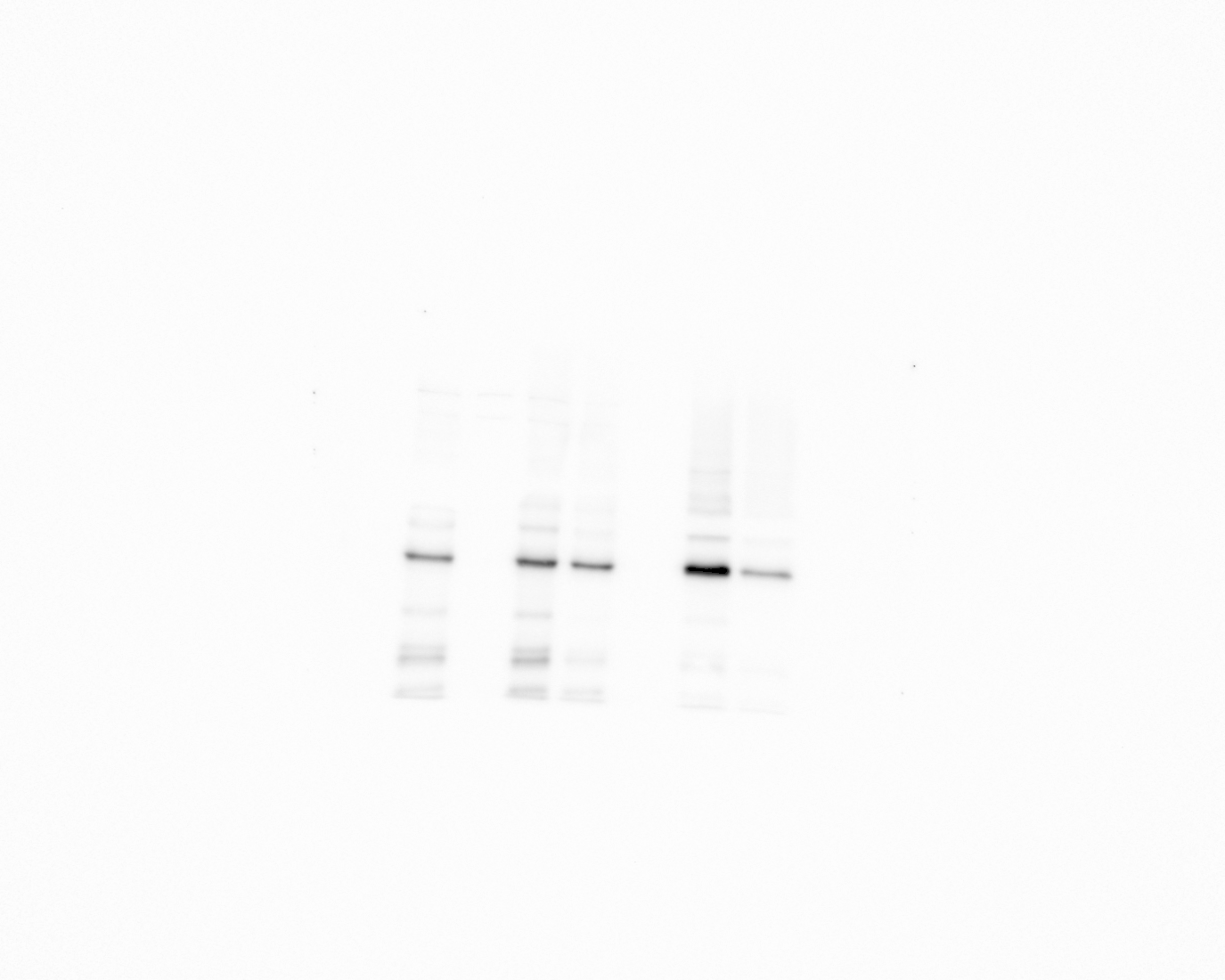

Supplement: Figure 4—source data 6. [file elife-89951-fig4-data6.zip › Figure 4-source data 6/WB_Ollas-SPOP_Figure 4-source data 6/Versteeg 2022-04-26 09h21m56s 145.340s(Chemiluminescence).raw16.tif]

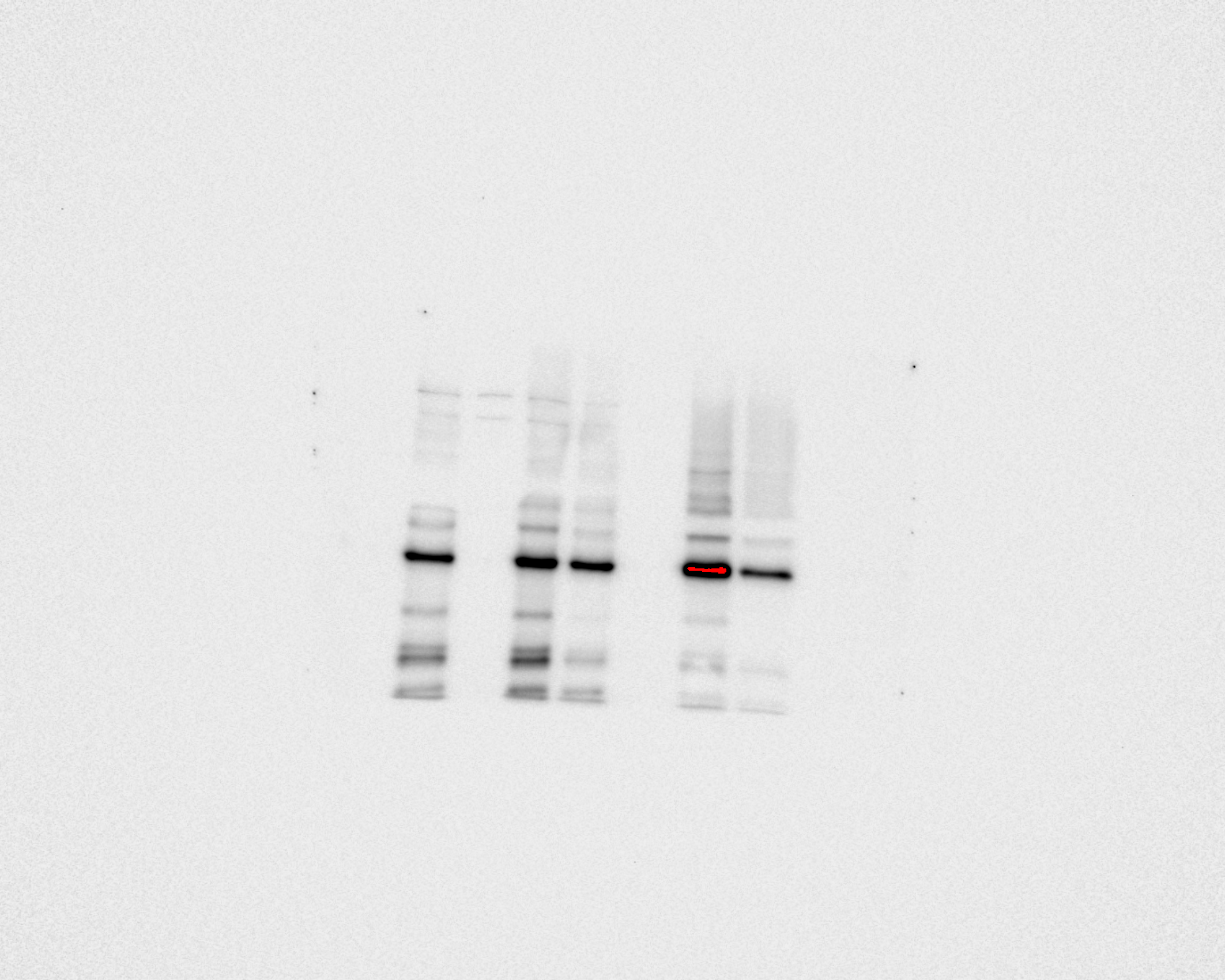

Supplement: Figure 4—source data 6. [file elife-89951-fig4-data6.zip › Figure 4-source data 6/WB_Ollas-SPOP_Figure 4-source data 6/Versteeg 2022-04-26 09h21m56s 145.340s(Chemiluminescence).tif]

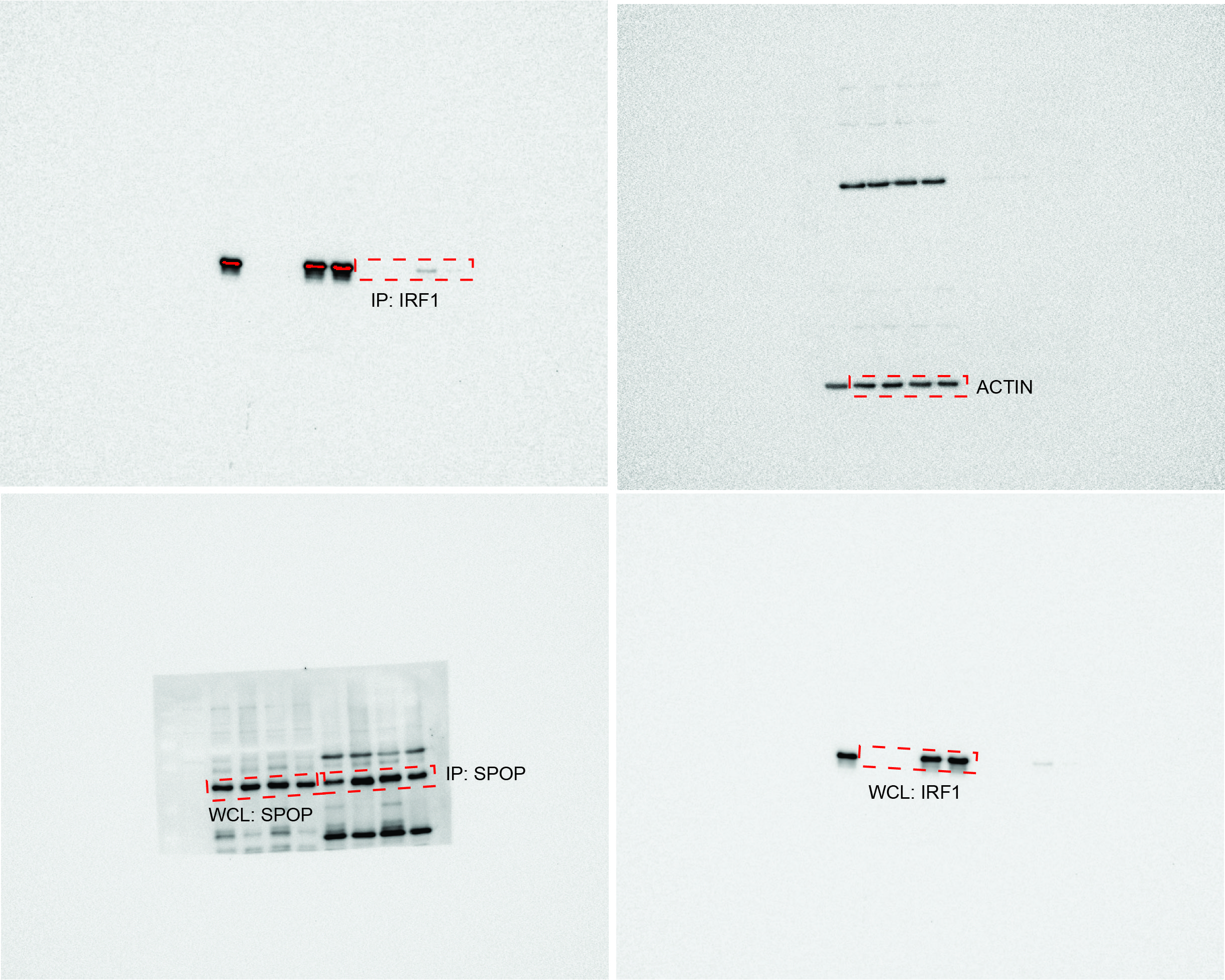

Supplement: Figure 4—source data 7. [file elife-89951-fig4-data7.zip › Figure 4-source data 7/Figure 4-source data 7.jpg]

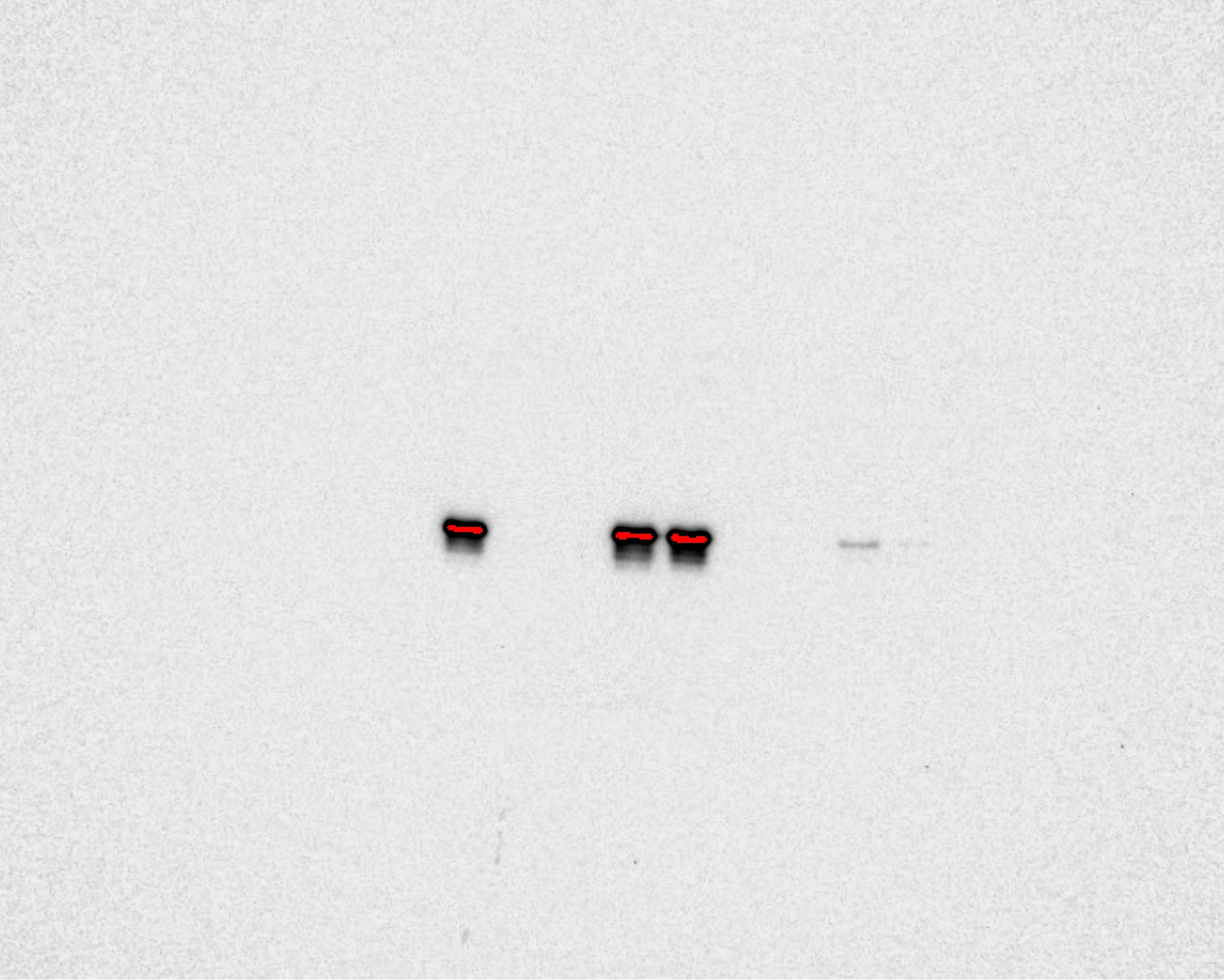

Supplement: Figure 4—source data 7. [file elife-89951-fig4-data7.zip › Figure 4-source data 7/IP_MYC-IRF1_Figure 4-source data 7/Versteeg 2022-04-26 09h16m37s 227.820s(Chemiluminescence).jpg]

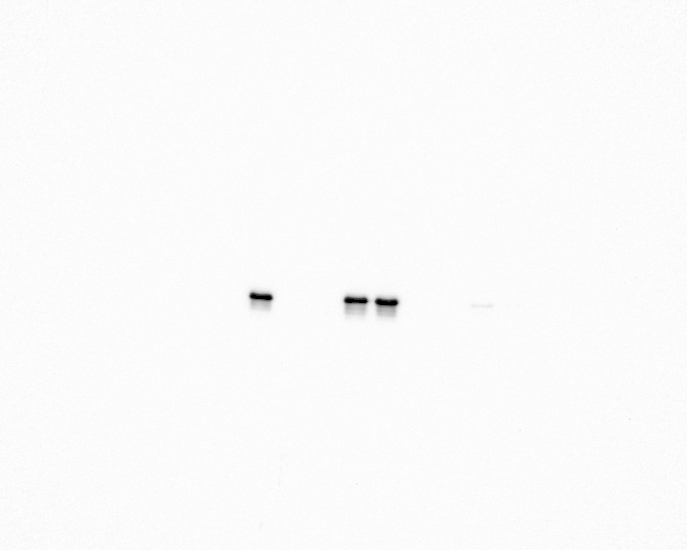

Supplement: Figure 4—source data 7. [file elife-89951-fig4-data7.zip › Figure 4-source data 7/IP_MYC-IRF1_Figure 4-source data 7/Versteeg 2022-04-26 09h16m37s 227.820s(Chemiluminescence).raw16.tif]

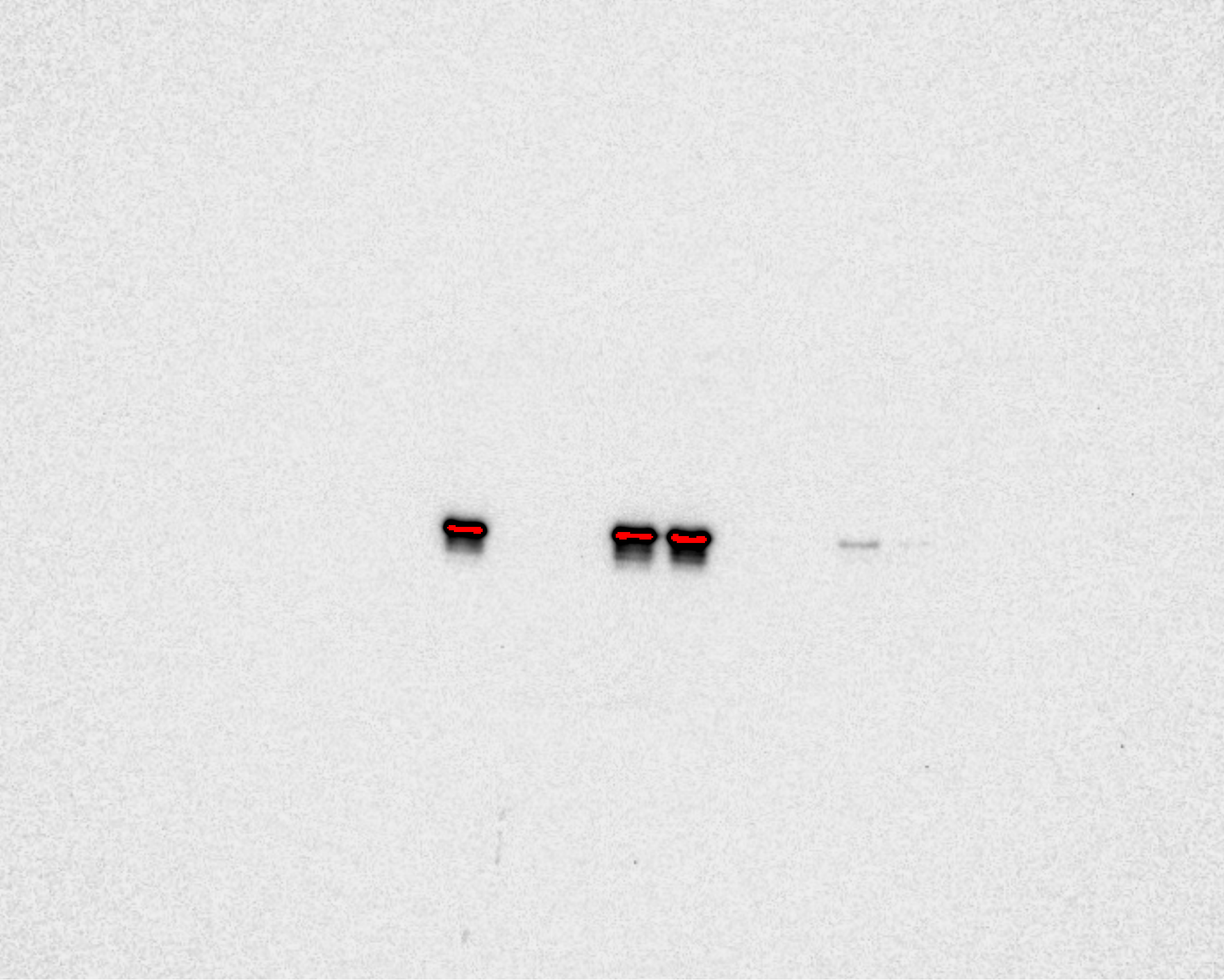

Supplement: Figure 4—source data 7. [file elife-89951-fig4-data7.zip › Figure 4-source data 7/IP_MYC-IRF1_Figure 4-source data 7/Versteeg 2022-04-26 09h16m37s 227.820s(Chemiluminescence).tif]

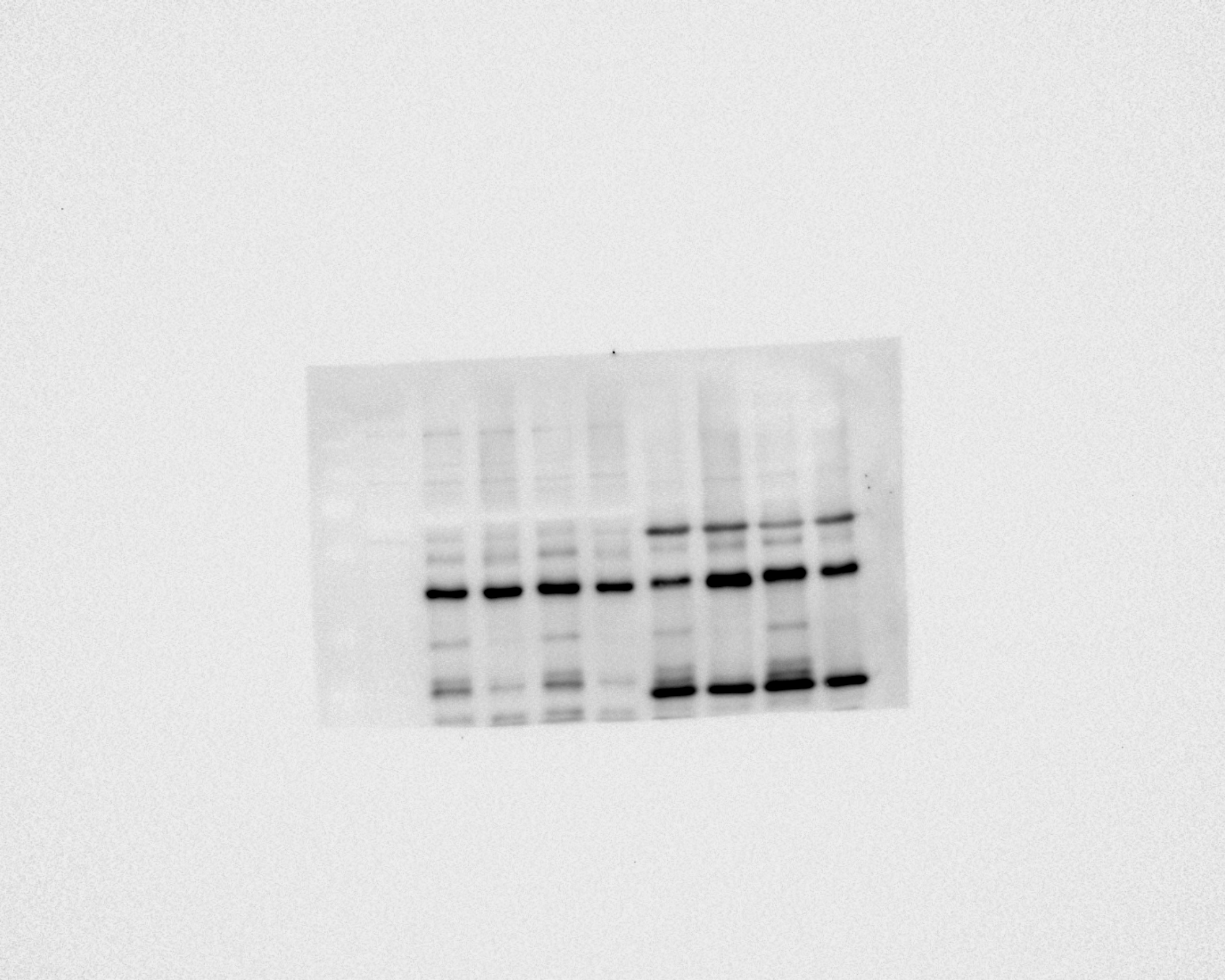

Supplement: Figure 4—source data 7. [file elife-89951-fig4-data7.zip › Figure 4-source data 7/WB_and_IP_Ollas-SPOP_Figure 4-source data 7/Versteeg 2022-04-22 12h20m14s 176.270s(Chemiluminescence).jpg]

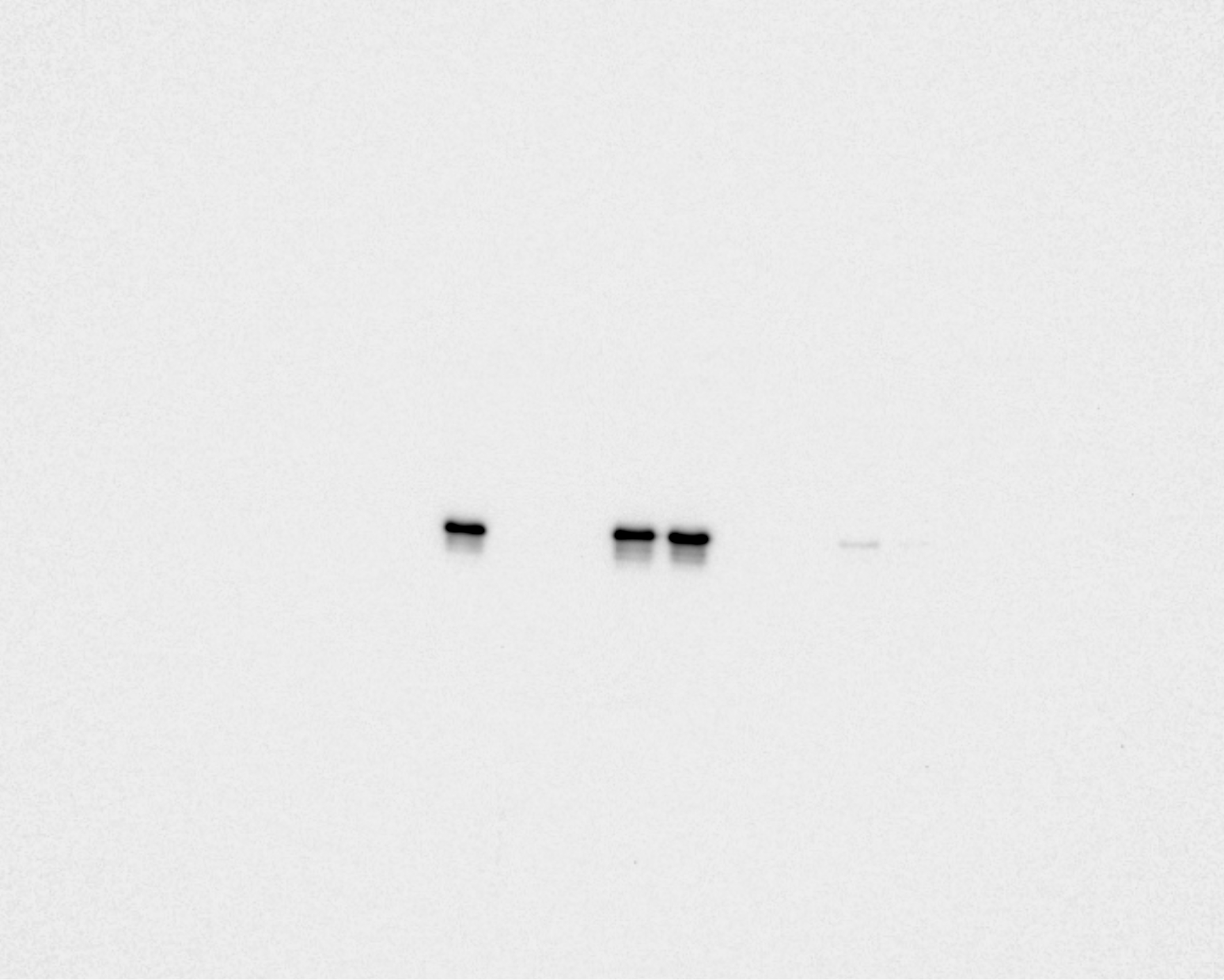

Supplement: Figure 4—source data 7. [file elife-89951-fig4-data7.zip › Figure 4-source data 7/WB_MYC-IRF1_Figure 4-source data 7/Versteeg 2022-04-26 09h13m49s 73.170s(Chemiluminescence).jpg]

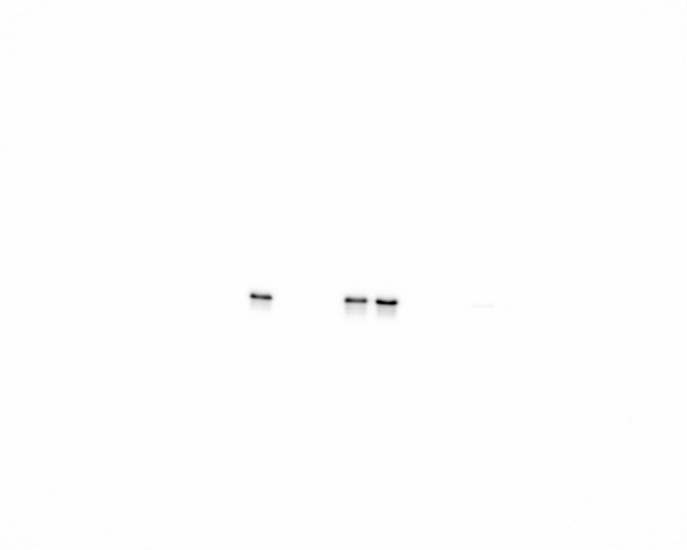

Supplement: Figure 4—source data 7. [file elife-89951-fig4-data7.zip › Figure 4-source data 7/WB_MYC-IRF1_Figure 4-source data 7/Versteeg 2022-04-26 09h13m49s 73.170s(Chemiluminescence).raw16.tif]

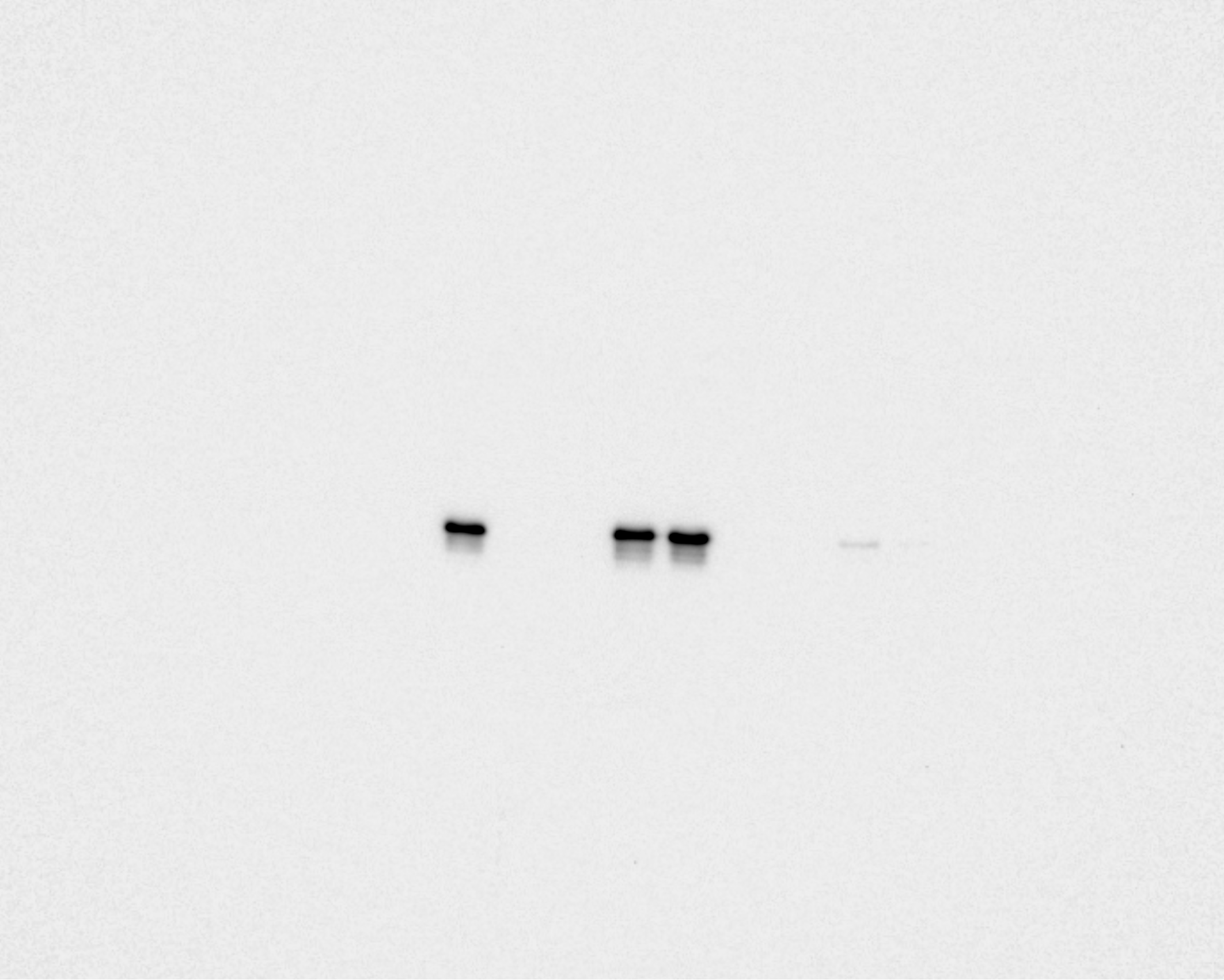

Supplement: Figure 4—source data 7. [file elife-89951-fig4-data7.zip › Figure 4-source data 7/WB_MYC-IRF1_Figure 4-source data 7/Versteeg 2022-04-26 09h13m49s 73.170s(Chemiluminescence).tif]

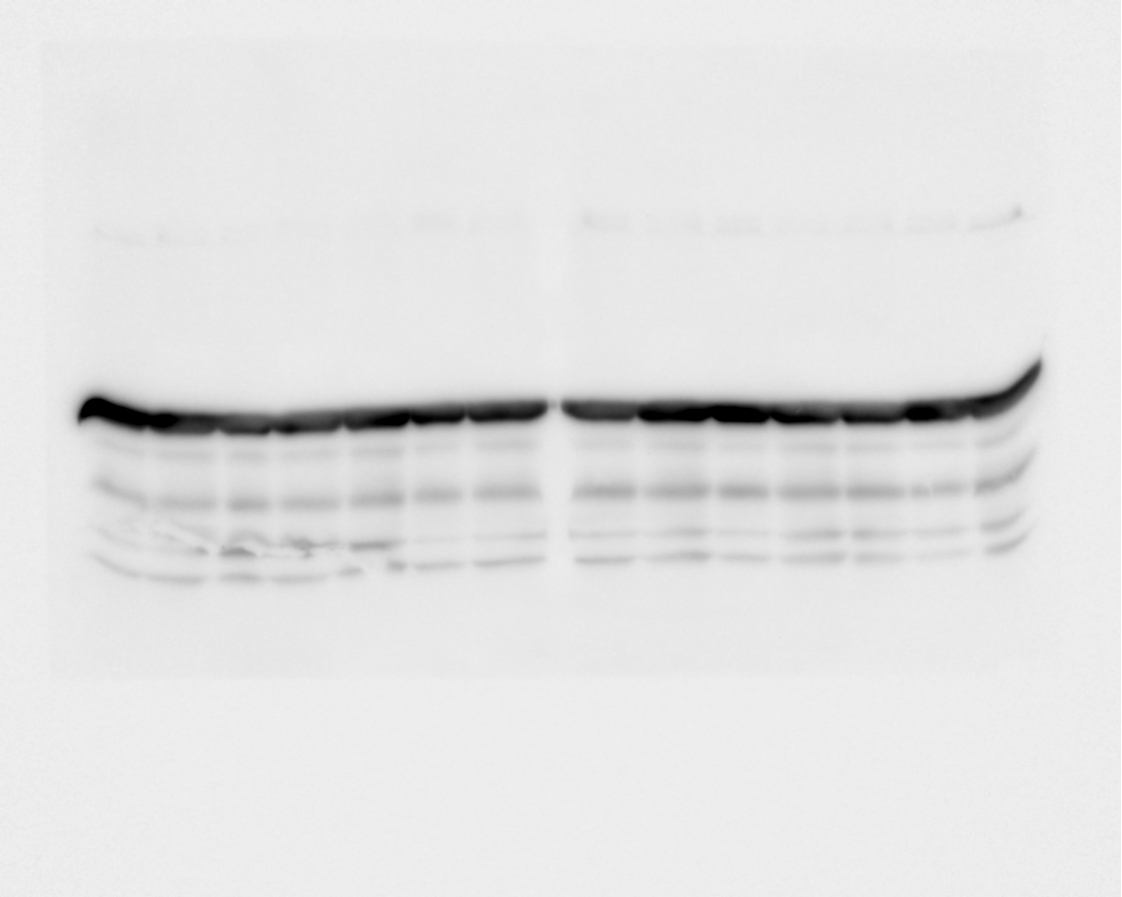

Supplement: Figure 4—source data 8. [file elife-89951-fig4-data8.zip › Figure 4-source data 8/ACTIN_Figure 4-source data 8/Versteeg 2023-05-09 12h44m26s 13.204s(Chemiluminescence).jpg]

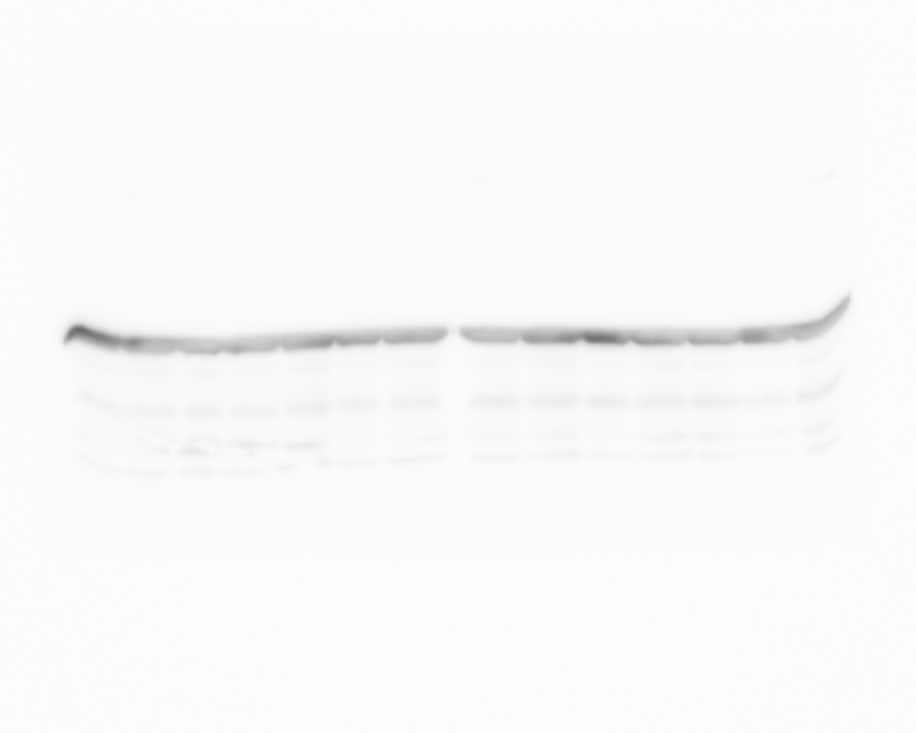

Supplement: Figure 4—source data 8. [file elife-89951-fig4-data8.zip › Figure 4-source data 8/ACTIN_Figure 4-source data 8/Versteeg 2023-05-09 12h44m26s 13.204s(Chemiluminescence).raw16.tif]

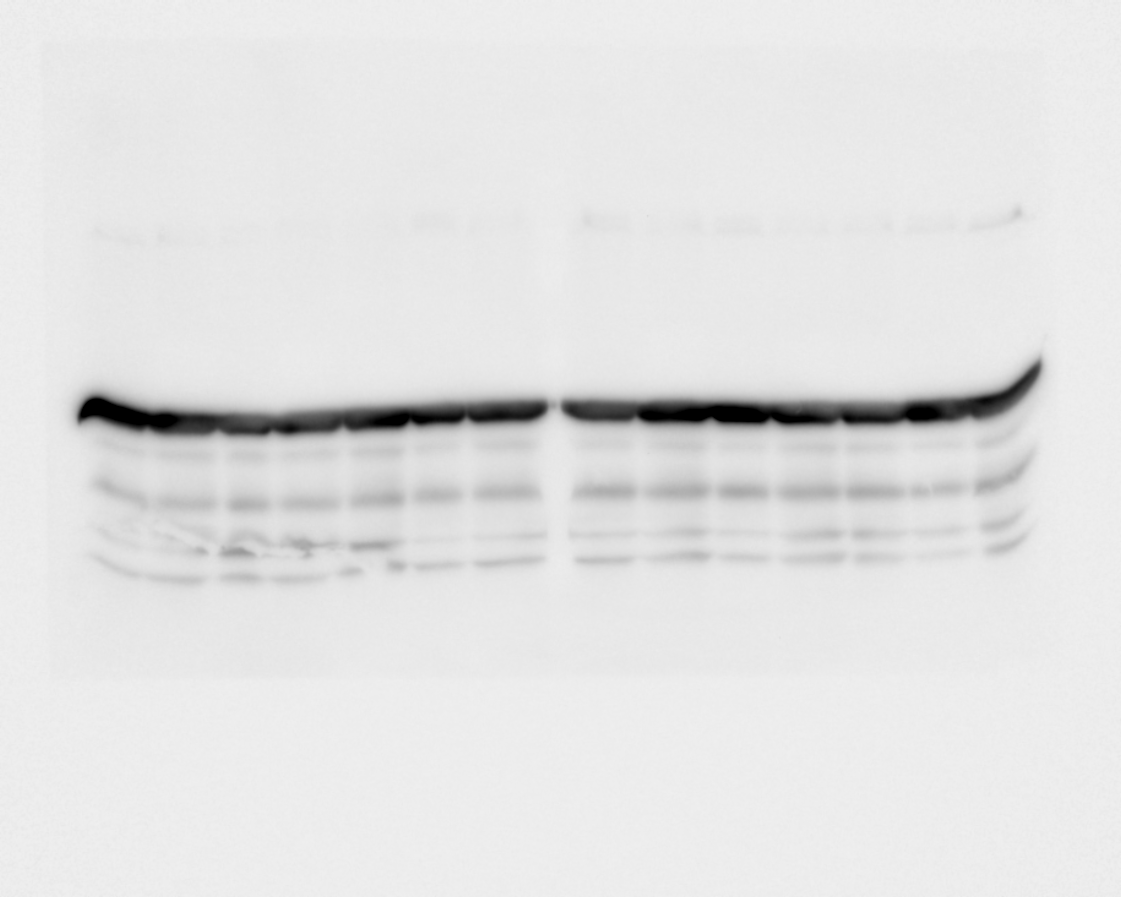

Supplement: Figure 4—source data 8. [file elife-89951-fig4-data8.zip › Figure 4-source data 8/ACTIN_Figure 4-source data 8/Versteeg 2023-05-09 12h44m26s 13.204s(Chemiluminescence).tif]

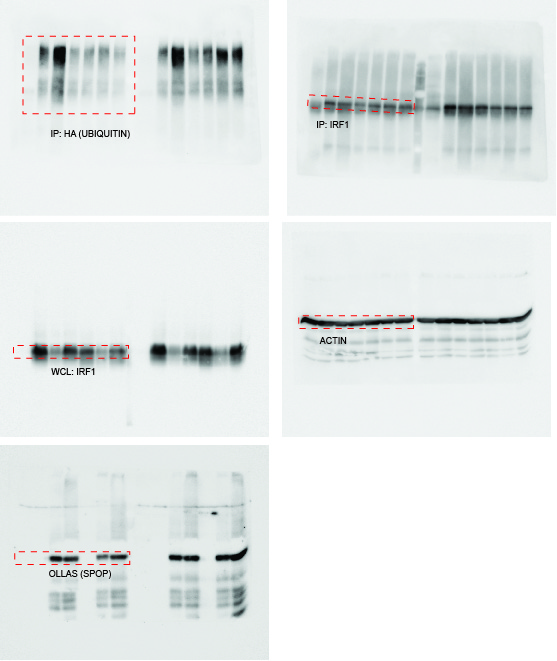

Supplement: Figure 4—source data 8. [file elife-89951-fig4-data8.zip › Figure 4-source data 8/Figure 4-source data 8.jpg]

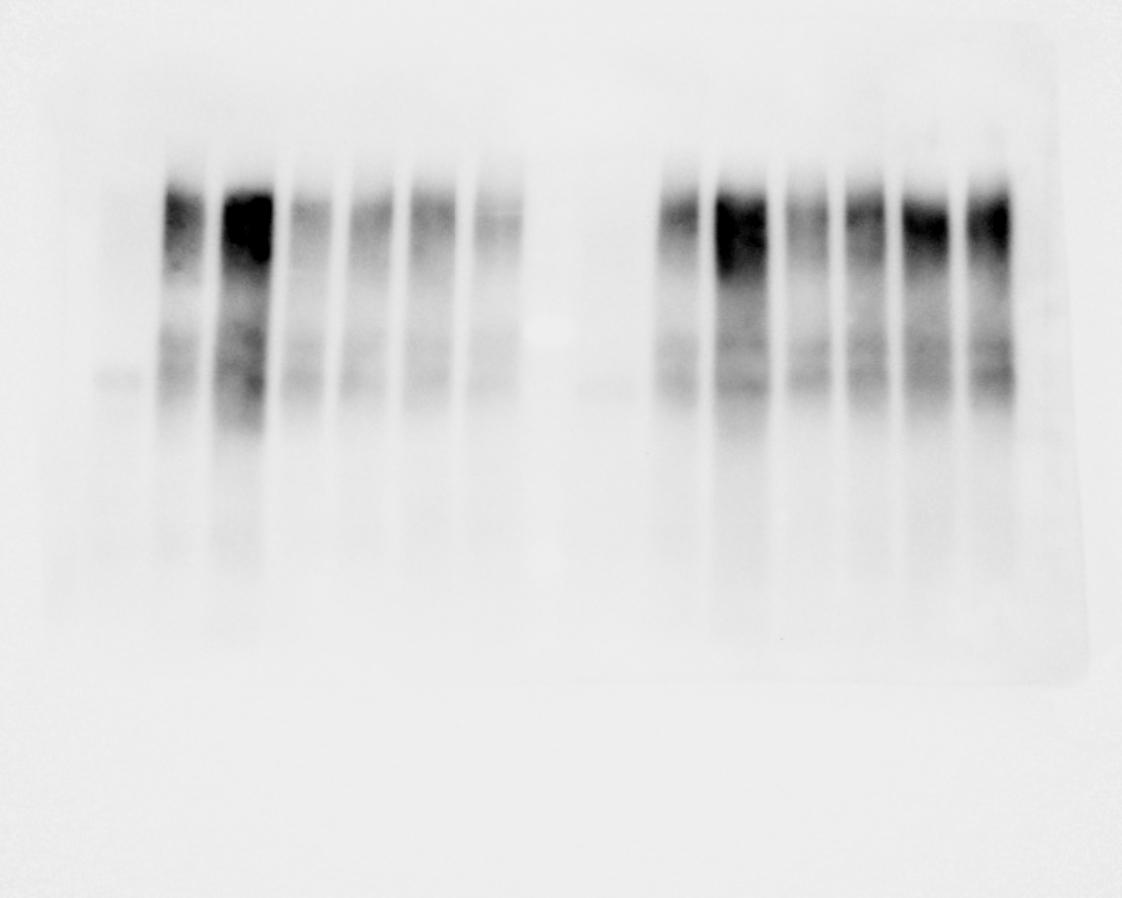

Supplement: Figure 4—source data 8. [file elife-89951-fig4-data8.zip › Figure 4-source data 8/HA (UBIQUITIN)_Figure 4-source data 8/Versteeg 2023-05-10 10h09m52s 5.000s(Chemiluminescence).jpg]

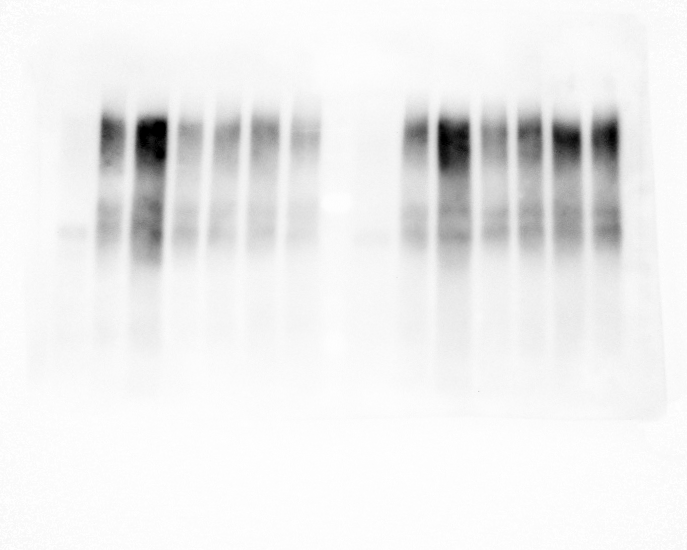

Supplement: Figure 4—source data 8. [file elife-89951-fig4-data8.zip › Figure 4-source data 8/HA (UBIQUITIN)_Figure 4-source data 8/Versteeg 2023-05-10 10h09m52s 5.000s(Chemiluminescence).raw16.tif]

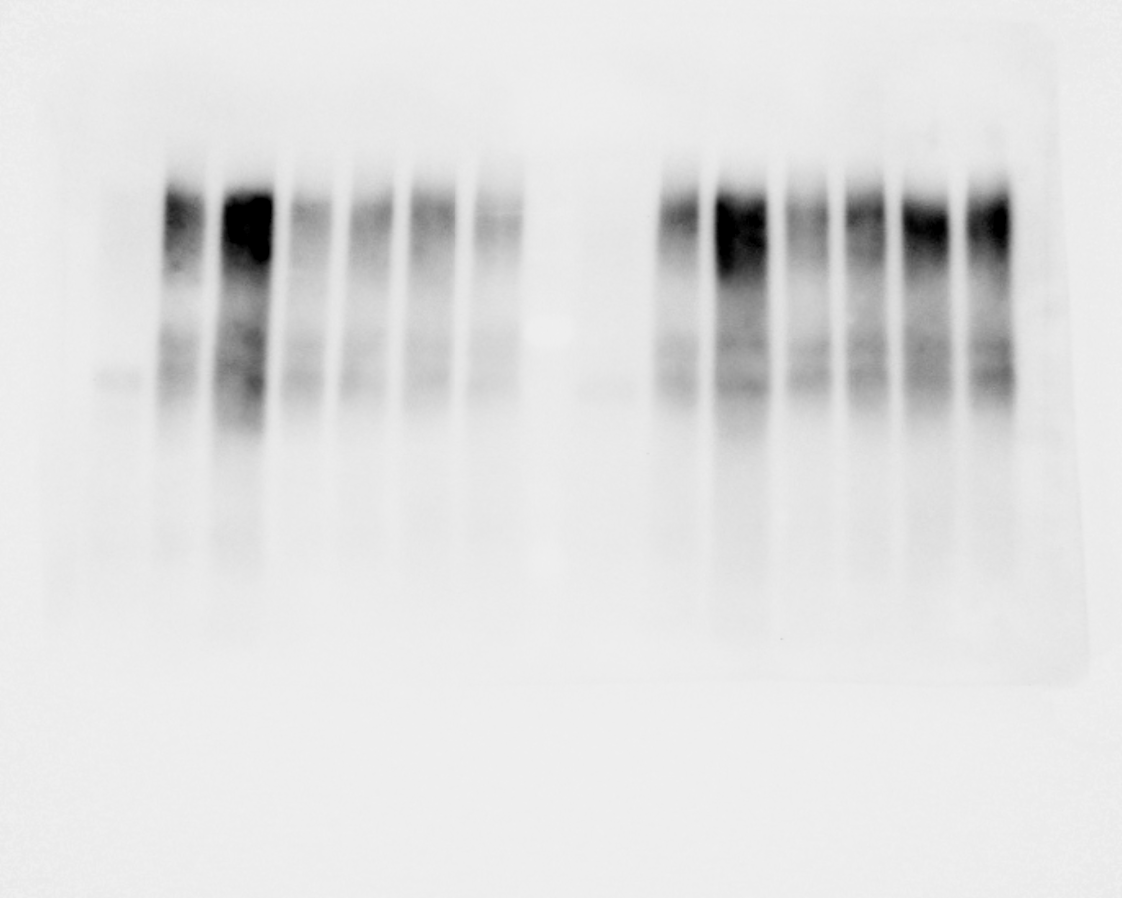

Supplement: Figure 4—source data 8. [file elife-89951-fig4-data8.zip › Figure 4-source data 8/HA (UBIQUITIN)_Figure 4-source data 8/Versteeg 2023-05-10 10h09m52s 5.000s(Chemiluminescence).tif]

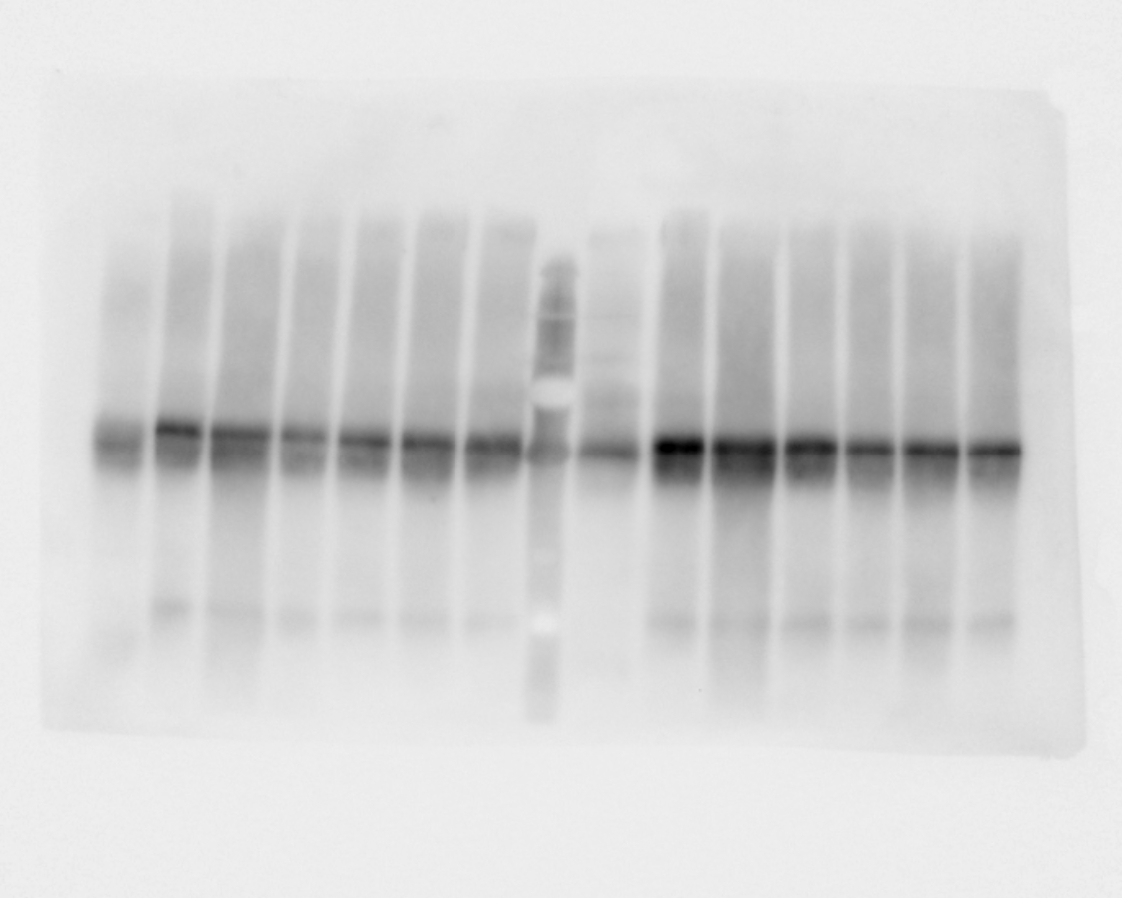

Supplement: Figure 4—source data 8. [file elife-89951-fig4-data8.zip › Figure 4-source data 8/IRF1_IP_Figure 4-source data 8/Versteeg 2023-05-11 10h18m21s 5.061s(Chemiluminescence).jpg]

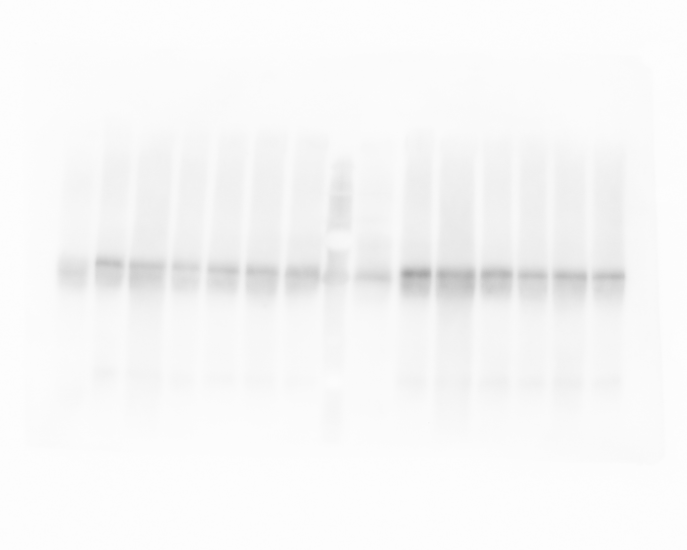

Supplement: Figure 4—source data 8. [file elife-89951-fig4-data8.zip › Figure 4-source data 8/IRF1_IP_Figure 4-source data 8/Versteeg 2023-05-11 10h18m21s 5.061s(Chemiluminescence).raw16.tif]

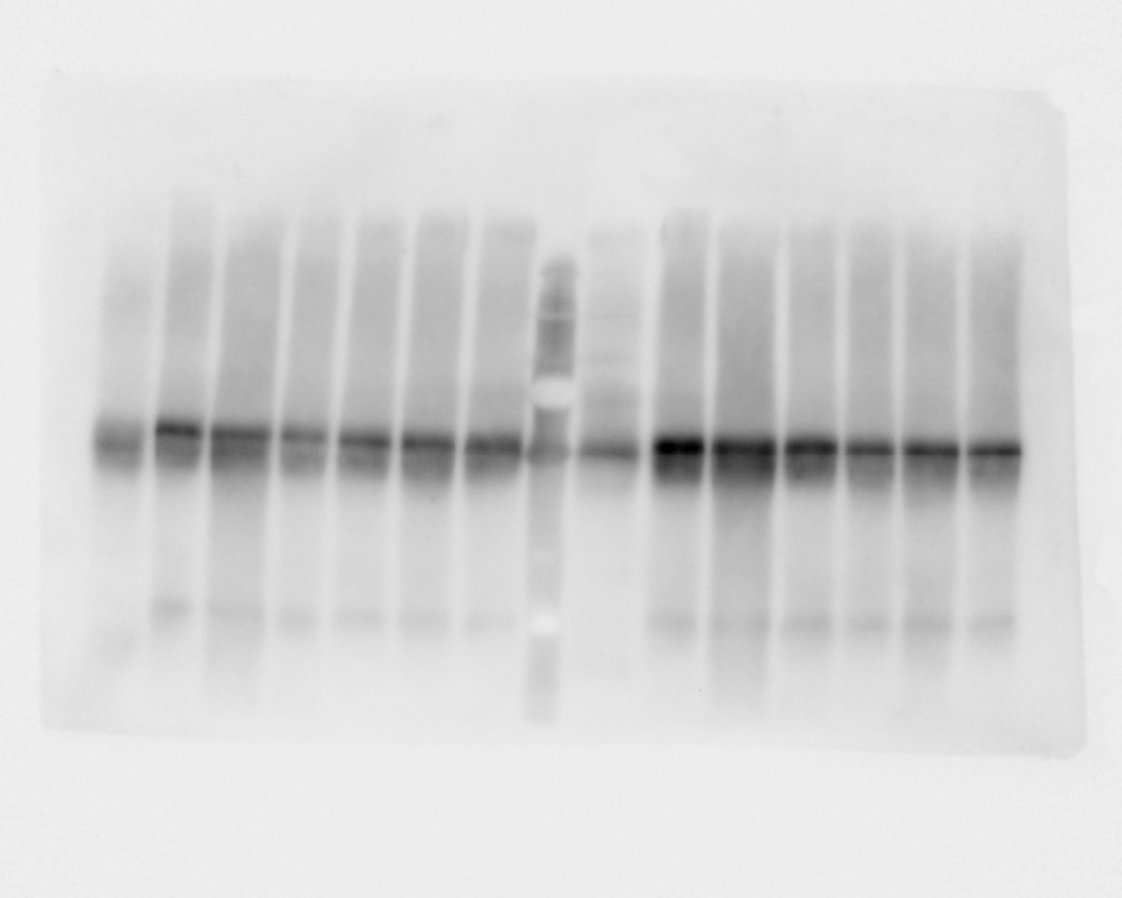

Supplement: Figure 4—source data 8. [file elife-89951-fig4-data8.zip › Figure 4-source data 8/IRF1_IP_Figure 4-source data 8/Versteeg 2023-05-11 10h18m21s 5.061s(Chemiluminescence).tif]

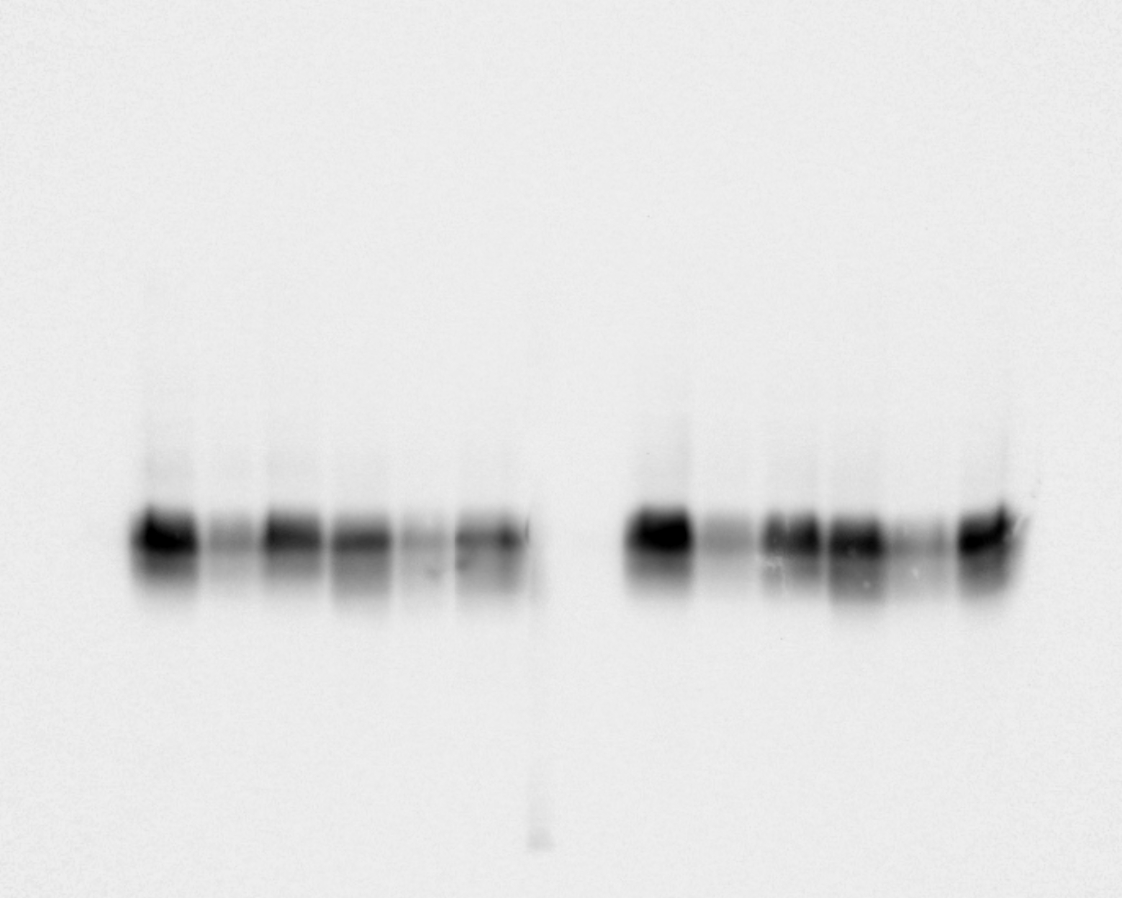

Supplement: Figure 4—source data 8. [file elife-89951-fig4-data8.zip › Figure 4-source data 8/IRF1_WCL_Figure 4-source data 8/Versteeg 2023-05-03 12h09m24s 17.244s(Chemiluminescence).jpg]

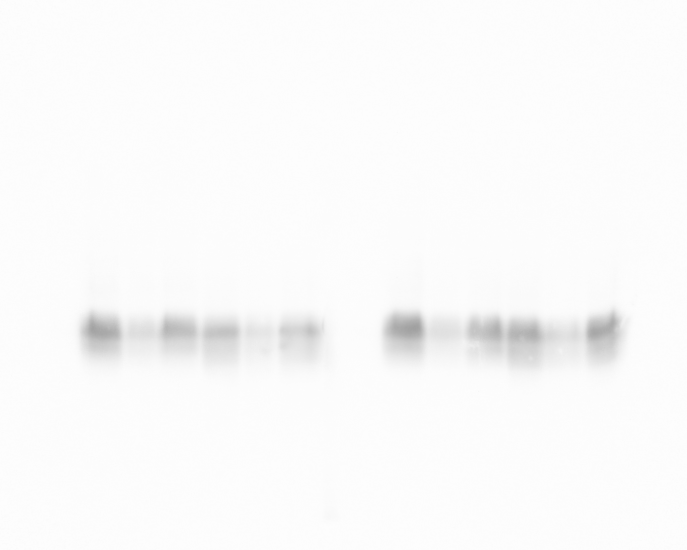

Supplement: Figure 4—source data 8. [file elife-89951-fig4-data8.zip › Figure 4-source data 8/IRF1_WCL_Figure 4-source data 8/Versteeg 2023-05-03 12h09m24s 17.244s(Chemiluminescence).raw16.tif]

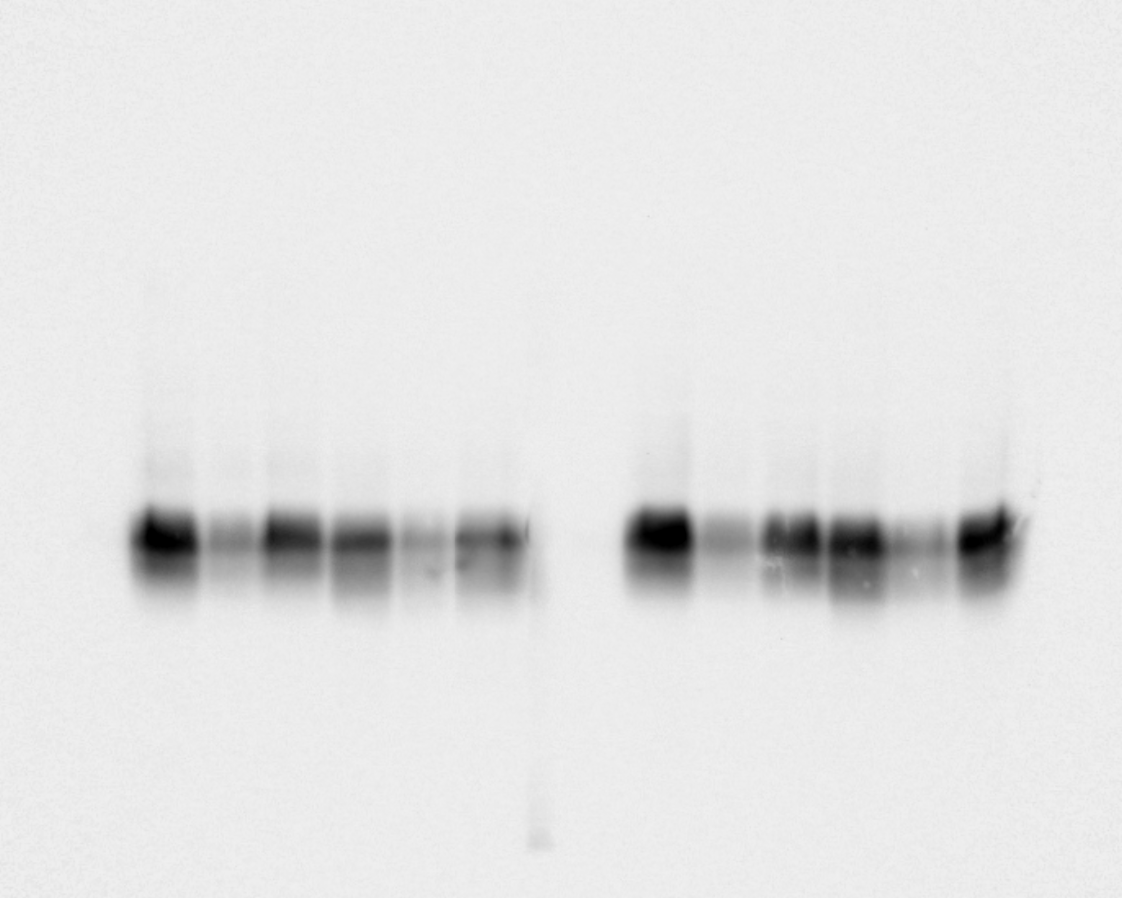

Supplement: Figure 4—source data 8. [file elife-89951-fig4-data8.zip › Figure 4-source data 8/IRF1_WCL_Figure 4-source data 8/Versteeg 2023-05-03 12h09m24s 17.244s(Chemiluminescence).tif]

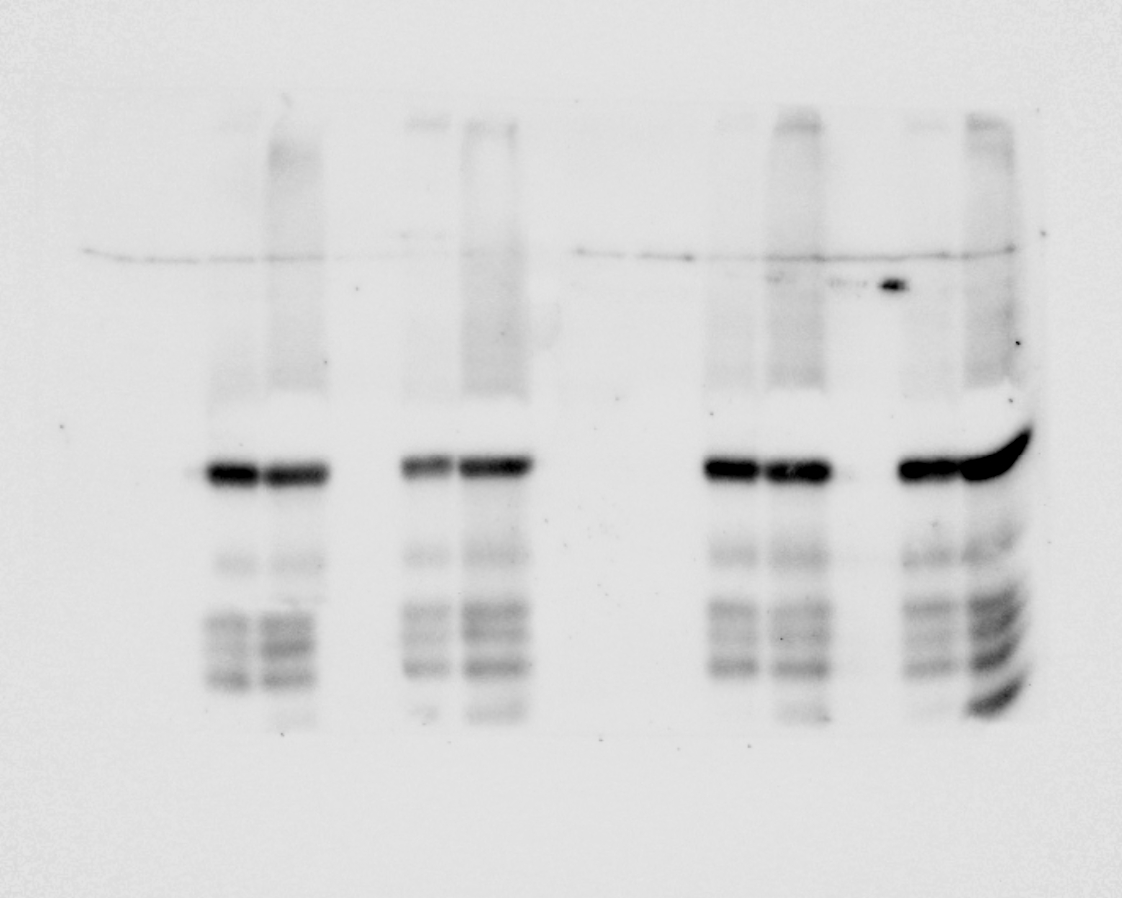

Supplement: Figure 4—source data 8. [file elife-89951-fig4-data8.zip › Figure 4-source data 8/OLLAS (SPOP)_Figure 4-source data 8/Versteeg 2023-05-05 11h01m56s 15.000s(Chemiluminescence).jpg]

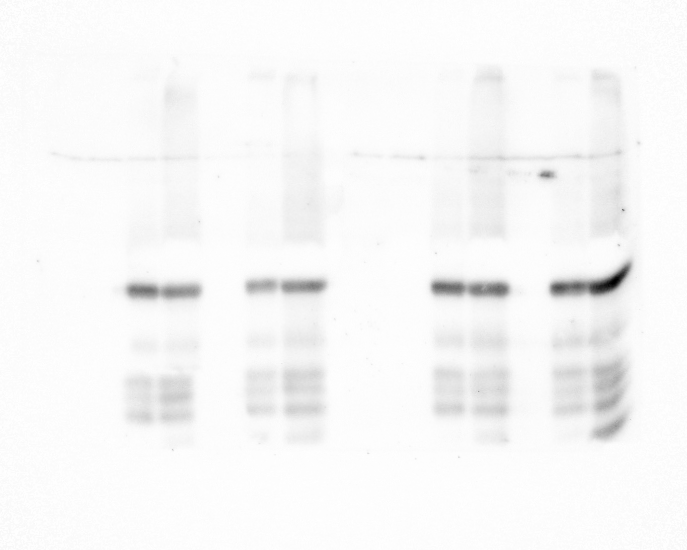

Supplement: Figure 4—source data 8. [file elife-89951-fig4-data8.zip › Figure 4-source data 8/OLLAS (SPOP)_Figure 4-source data 8/Versteeg 2023-05-05 11h01m56s 15.000s(Chemiluminescence).raw16.tif]

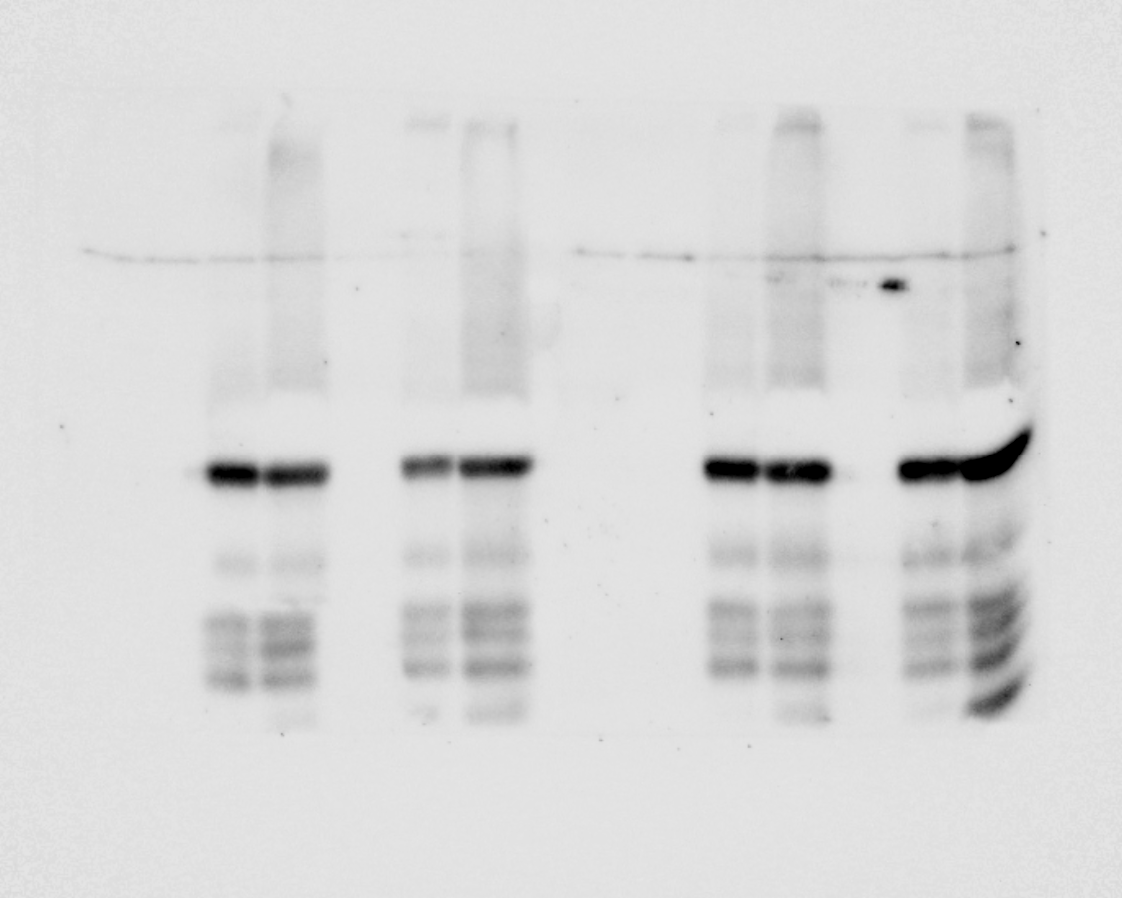

Supplement: Figure 4—source data 8. [file elife-89951-fig4-data8.zip › Figure 4-source data 8/OLLAS (SPOP)_Figure 4-source data 8/Versteeg 2023-05-05 11h01m56s 15.000s(Chemiluminescence).tif]

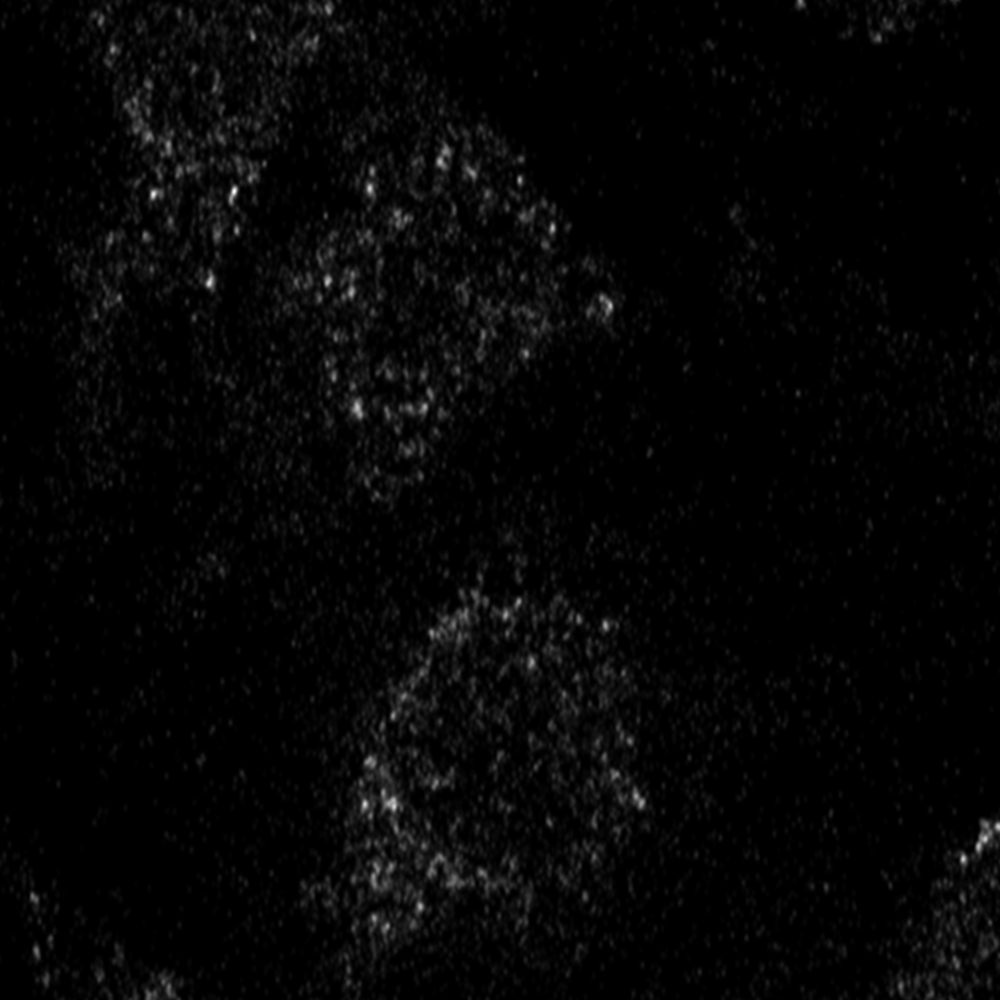

Supplement: Figure 4—figure supplement 1—source data 1. [file elife-89951-fig4-figsupp1-data1.zip › Figure 4-figure supplement 1-source data 1/IRF1_EPOX_Figure 4-figure supplement 1-source data 1.tif]

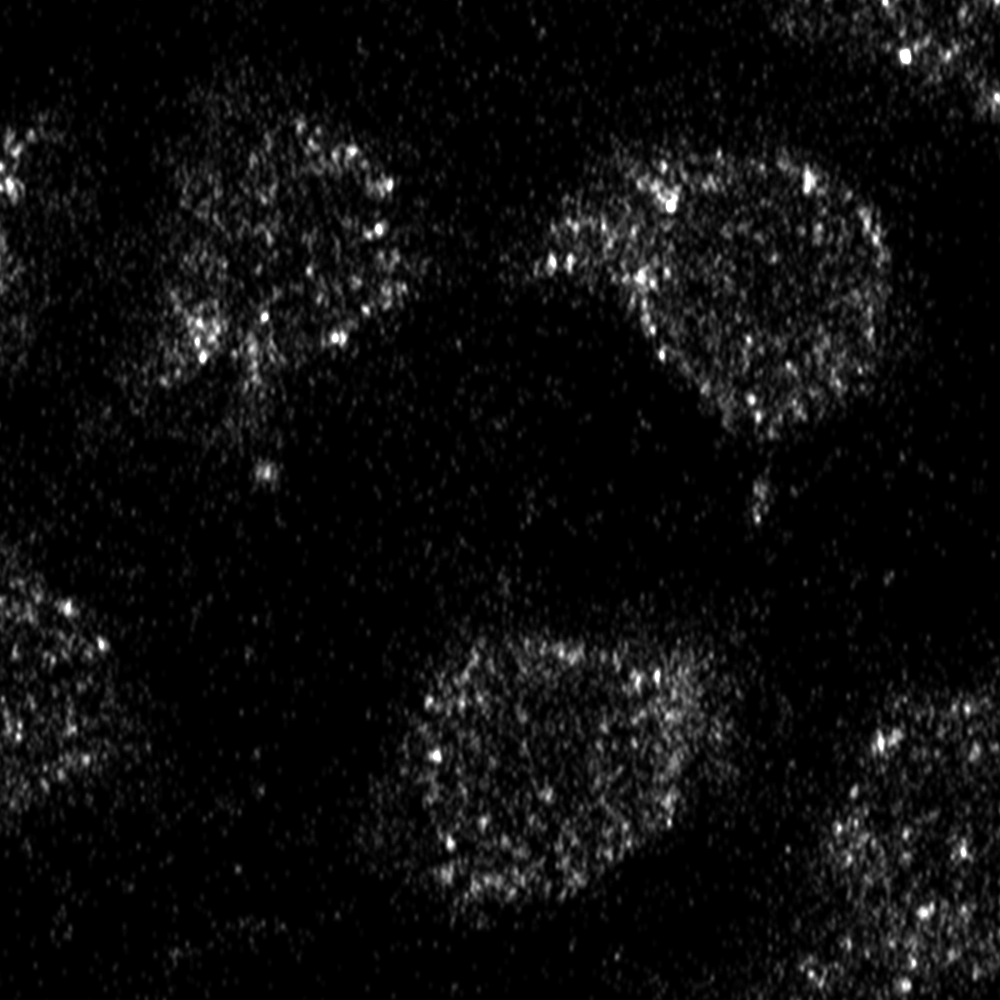

Supplement: Figure 4—figure supplement 1—source data 1. [file elife-89951-fig4-figsupp1-data1.zip › Figure 4-figure supplement 1-source data 1/IRF1_UNTR_Figure 4-figure supplement 1-source data 1.tif]

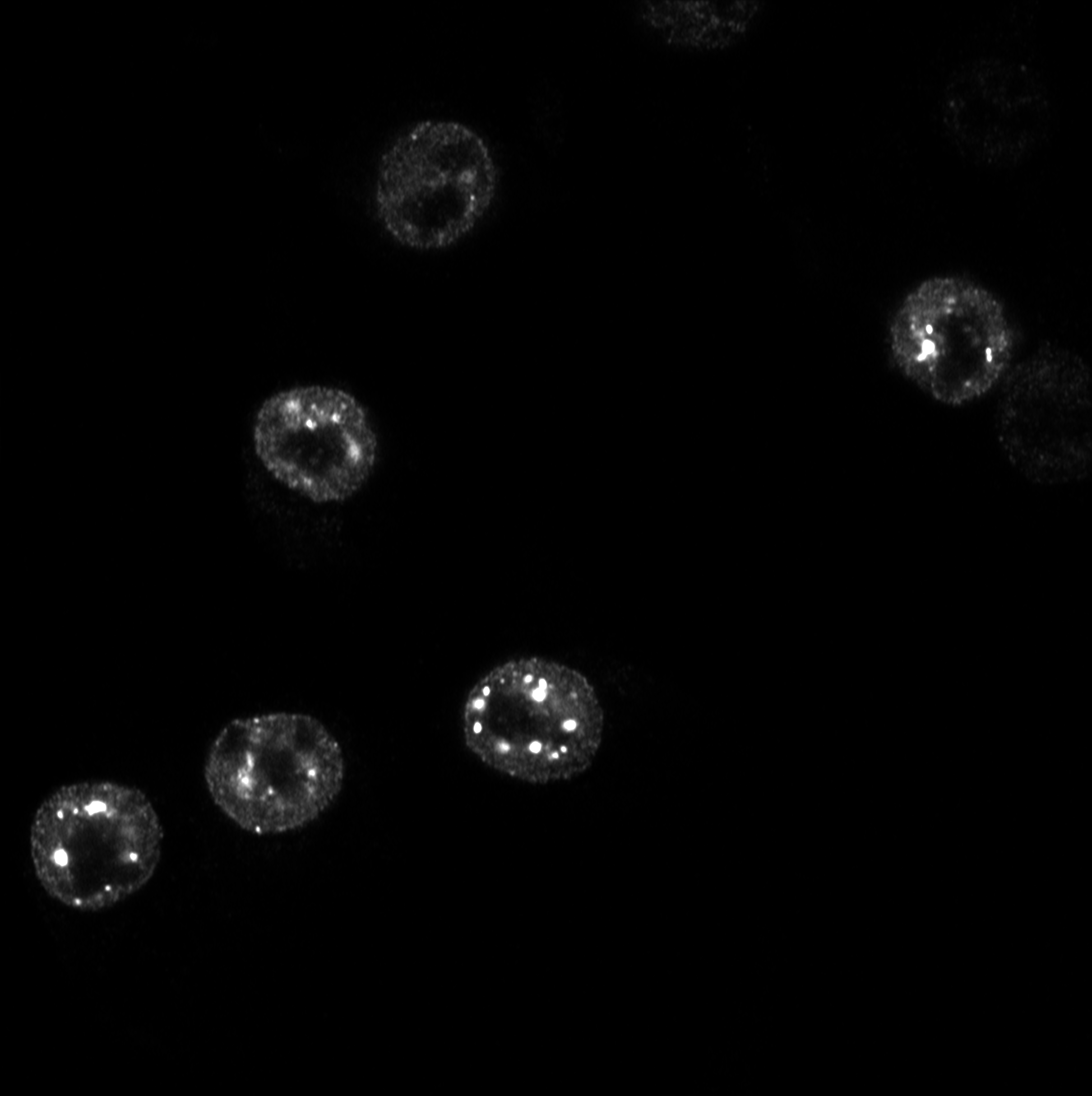

Supplement: Figure 4—figure supplement 1—source data 1. [file elife-89951-fig4-figsupp1-data1.zip › Figure 4-figure supplement 1-source data 1/IRF1+SPOP_EPOX_Figure 4-figure supplement 1-source data 1.tif]

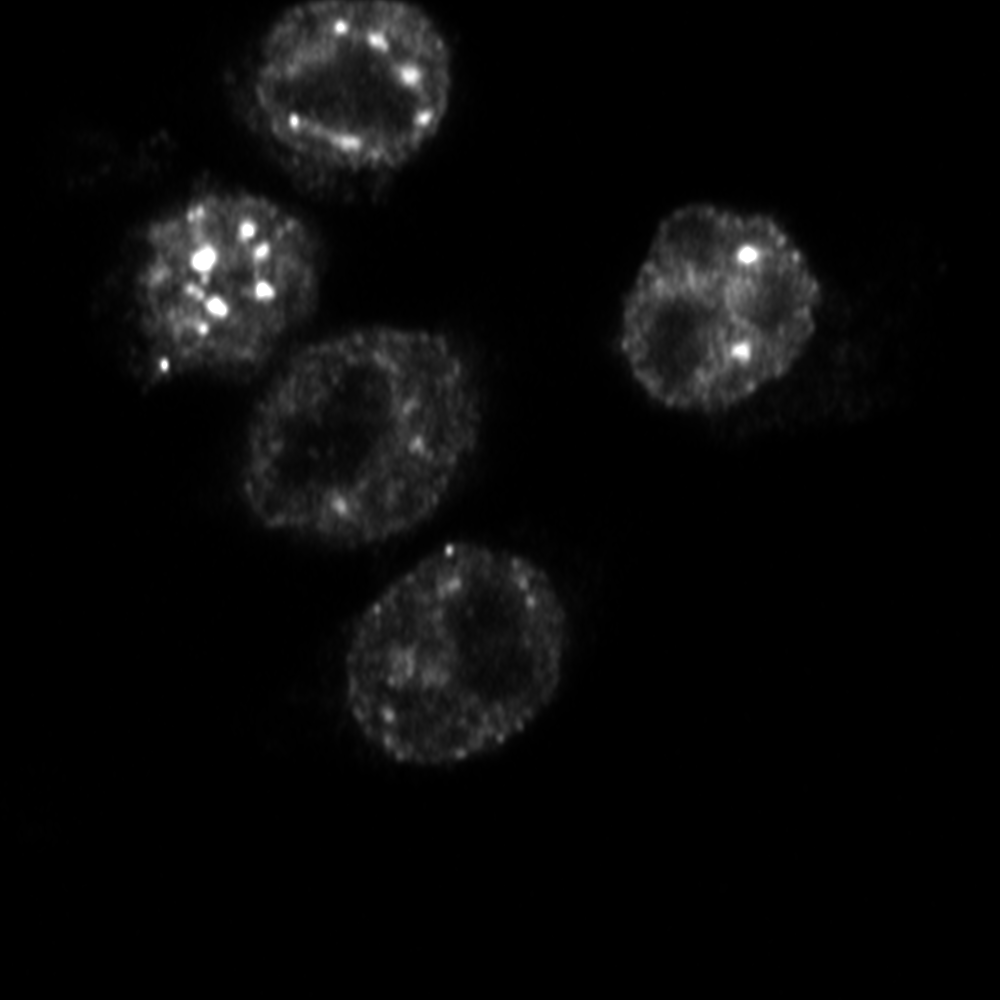

Supplement: Figure 4—figure supplement 1—source data 1. [file elife-89951-fig4-figsupp1-data1.zip › Figure 4-figure supplement 1-source data 1/SPOP_EPOX_Figure 4-figure supplement 1-source data 1.tif]

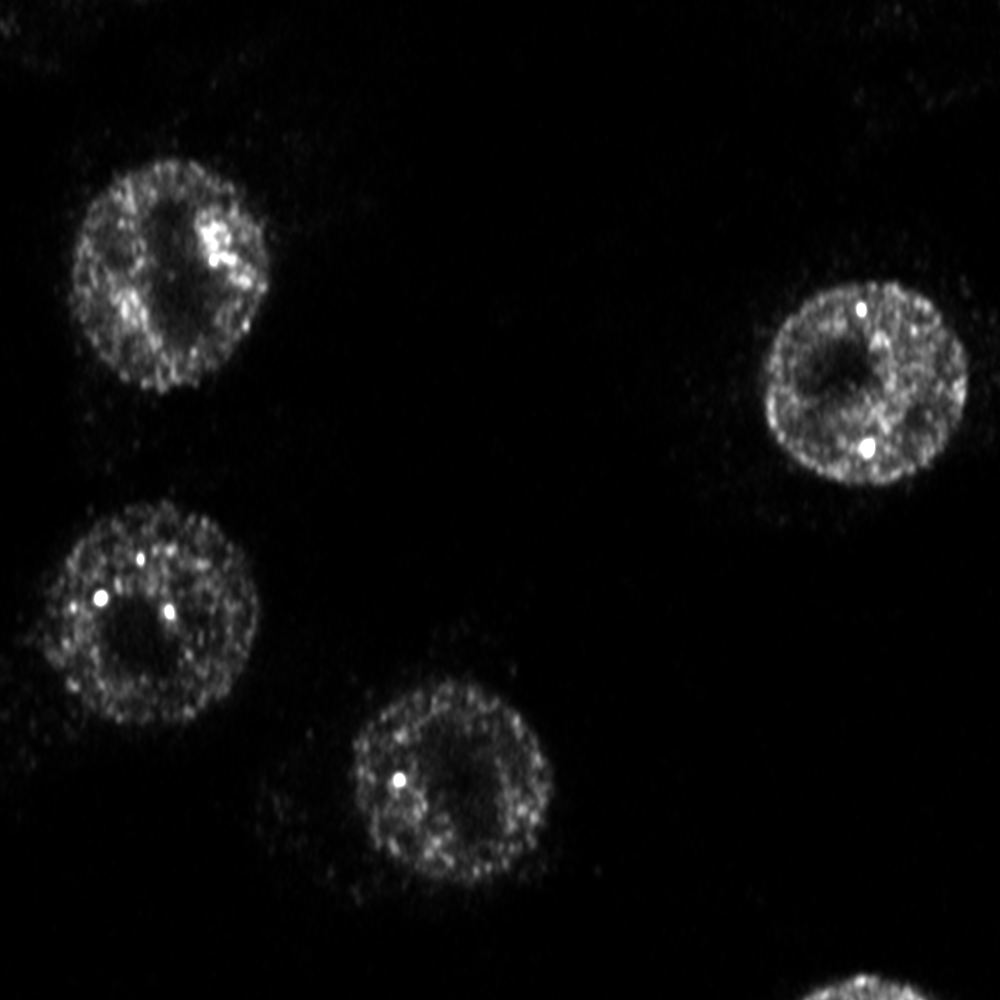

Supplement: Figure 4—figure supplement 1—source data 1. [file elife-89951-fig4-figsupp1-data1.zip › Figure 4-figure supplement 1-source data 1/SPOP_UNTR_Figure 4-figure supplement 1-source data 1.tif]

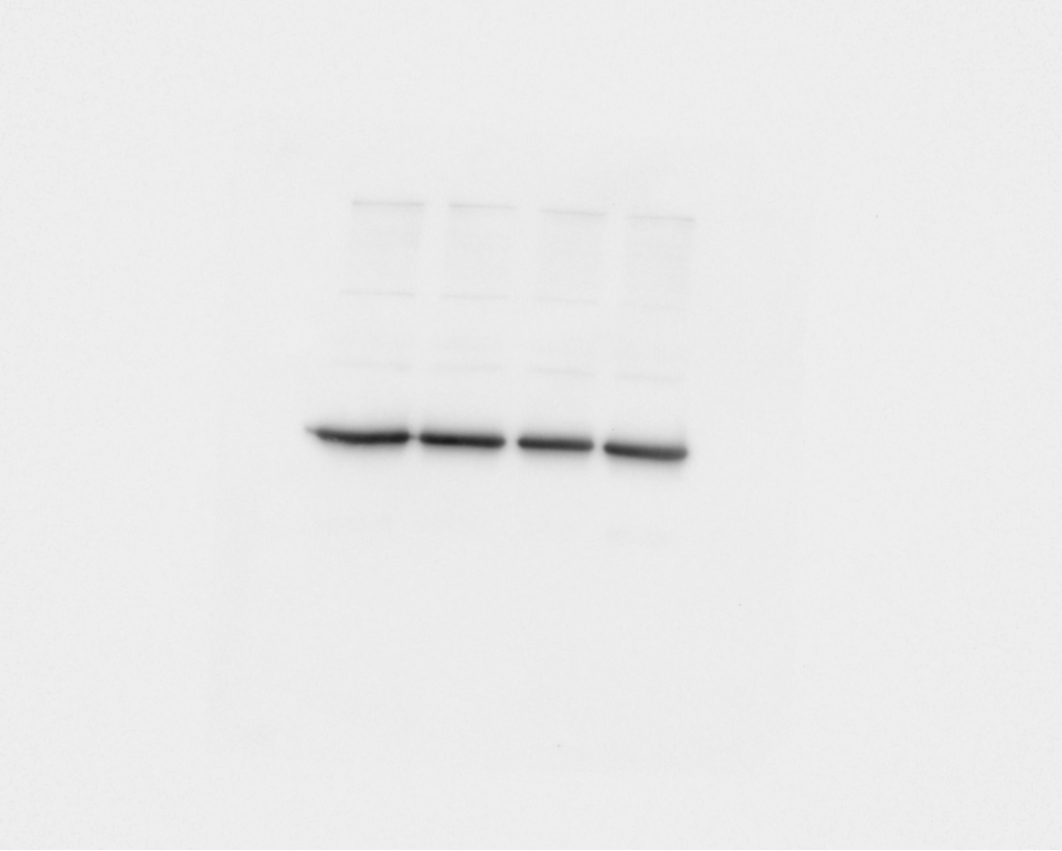

Supplement: Figure 4—figure supplement 1—source data 2. [file elife-89951-fig4-figsupp1-data2.zip › Figure 4-figure supplement 1-source data 2/ACTIN_Figure 4-figure supplement 1-source data 2/Versteeg 2023-07-28 15h10m42s 5.856s(Chemiluminescence).jpg]

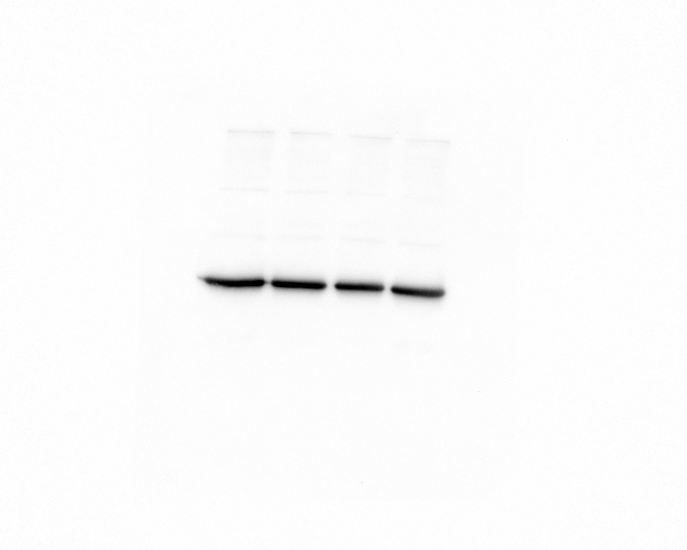

Supplement: Figure 4—figure supplement 1—source data 2. [file elife-89951-fig4-figsupp1-data2.zip › Figure 4-figure supplement 1-source data 2/ACTIN_Figure 4-figure supplement 1-source data 2/Versteeg 2023-07-28 15h10m42s 5.856s(Chemiluminescence).raw16.tif]

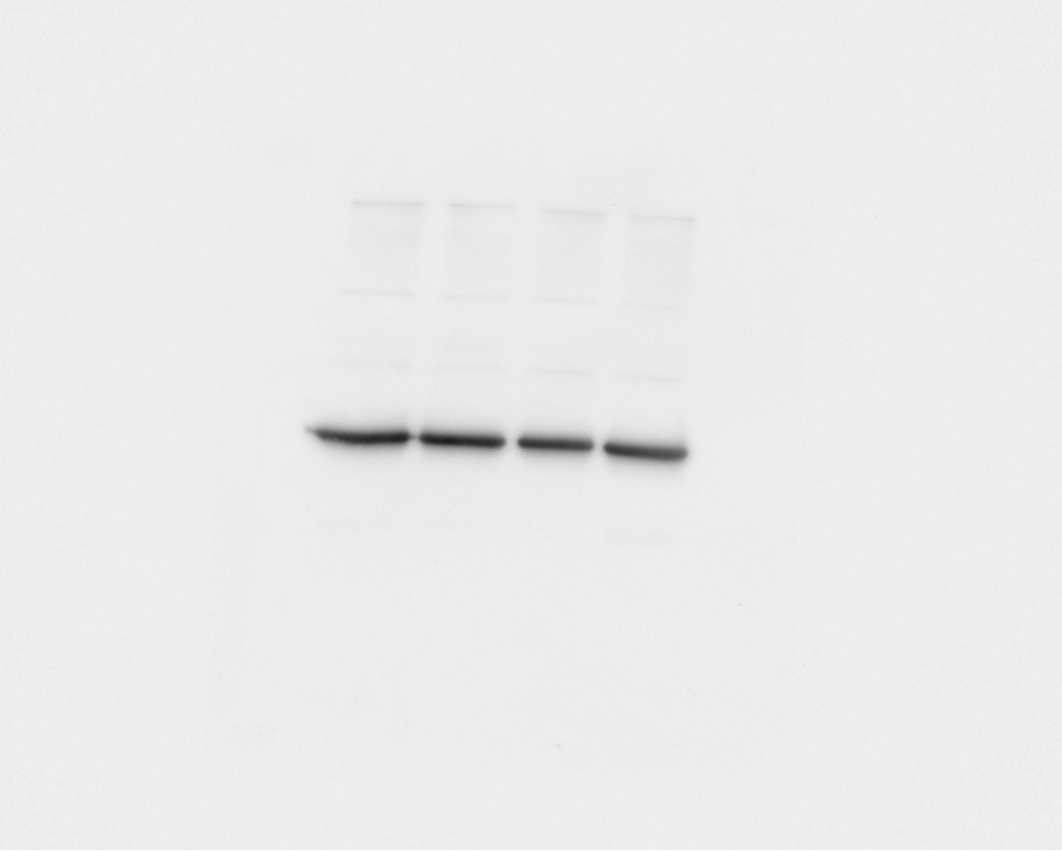

Supplement: Figure 4—figure supplement 1—source data 2. [file elife-89951-fig4-figsupp1-data2.zip › Figure 4-figure supplement 1-source data 2/ACTIN_Figure 4-figure supplement 1-source data 2/Versteeg 2023-07-28 15h10m42s 5.856s(Chemiluminescence).tif]

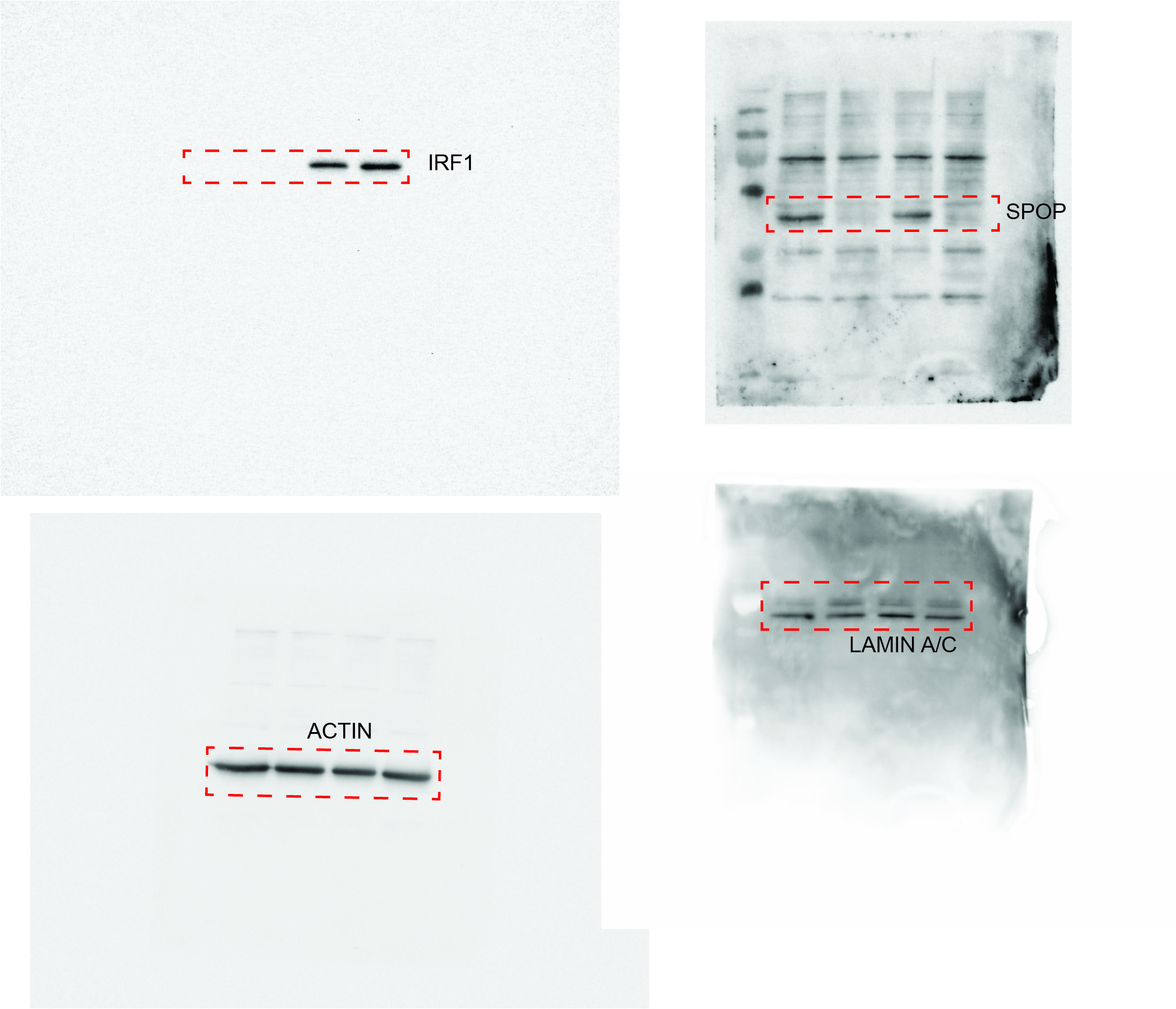

Supplement: Figure 4—figure supplement 1—source data 2. [file elife-89951-fig4-figsupp1-data2.zip › Figure 4-figure supplement 1-source data 2/Figure 4-figure supplement 1-source data 2.jpg]

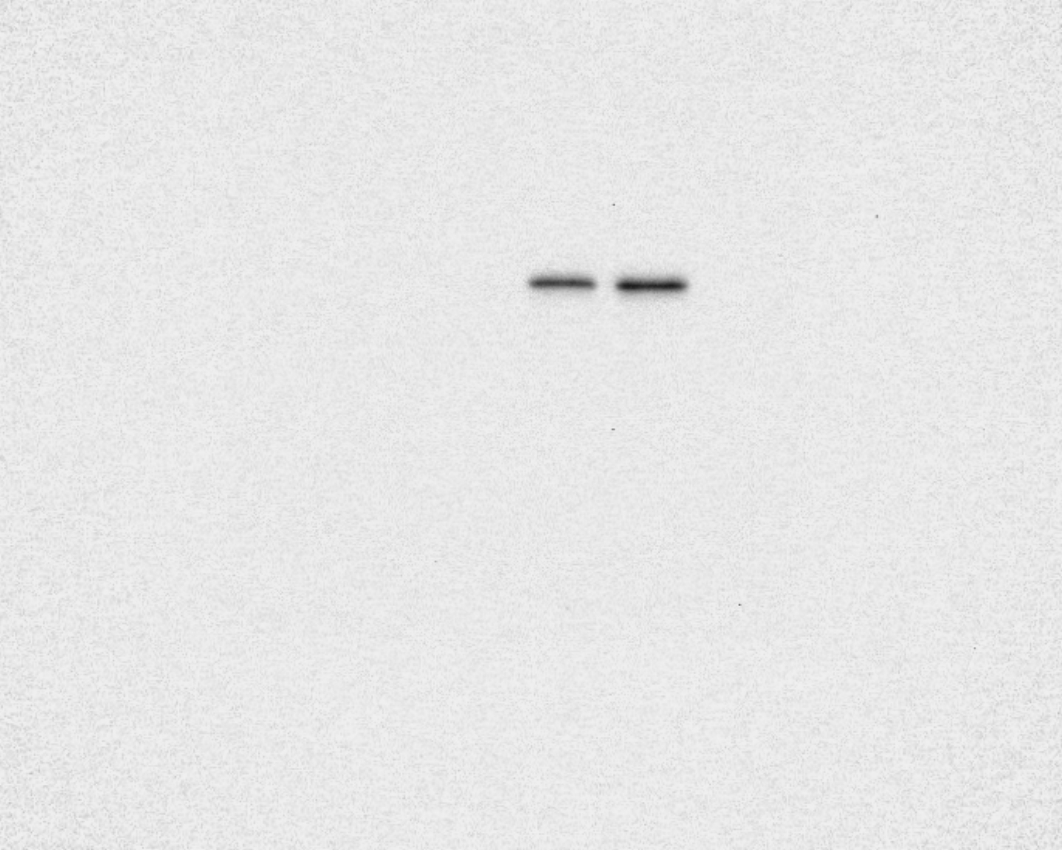

Supplement: Figure 4—figure supplement 1—source data 2. [file elife-89951-fig4-figsupp1-data2.zip › Figure 4-figure supplement 1-source data 2/IRF1_Figure 4-figure supplement 1-source data 2/Versteeg 2023-07-28 10h14m51s 135.244s(Chemiluminescence).jpg]

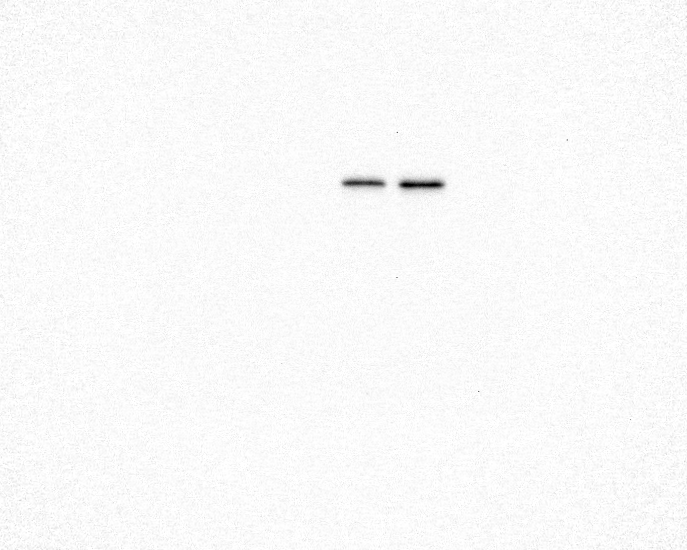

Supplement: Figure 4—figure supplement 1—source data 2. [file elife-89951-fig4-figsupp1-data2.zip › Figure 4-figure supplement 1-source data 2/IRF1_Figure 4-figure supplement 1-source data 2/Versteeg 2023-07-28 10h14m51s 135.244s(Chemiluminescence).raw16.tif]

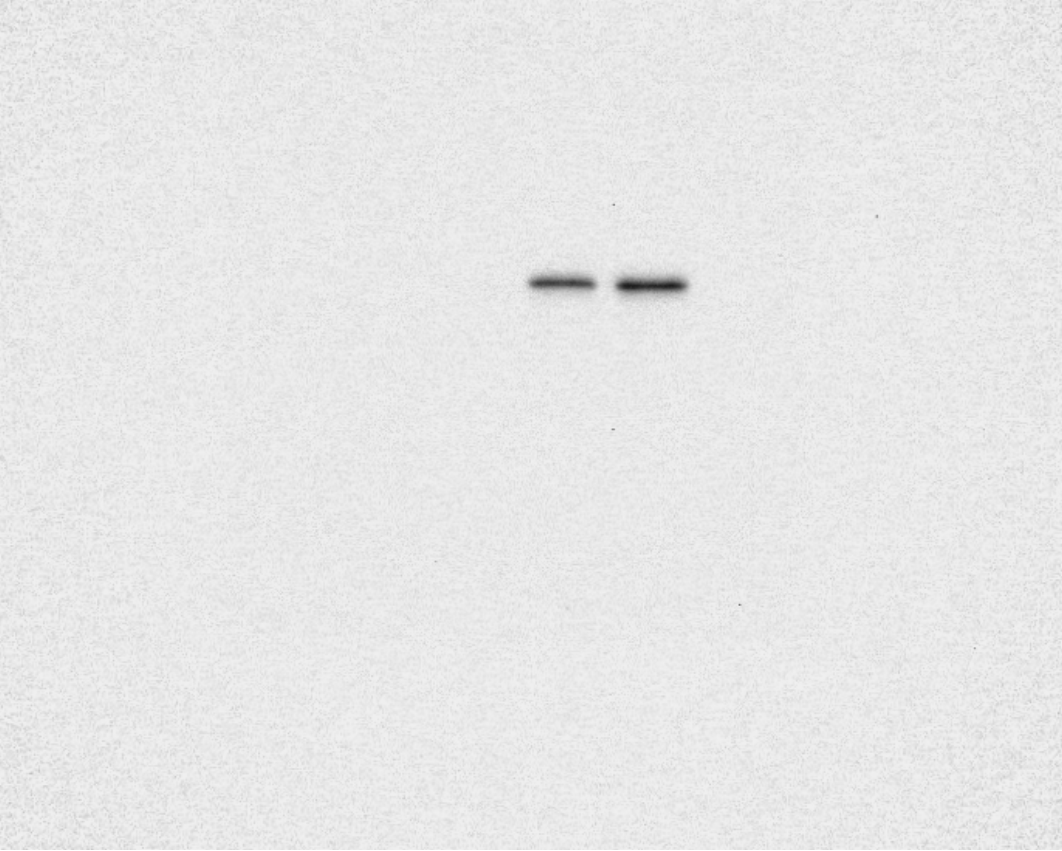

Supplement: Figure 4—figure supplement 1—source data 2. [file elife-89951-fig4-figsupp1-data2.zip › Figure 4-figure supplement 1-source data 2/IRF1_Figure 4-figure supplement 1-source data 2/Versteeg 2023-07-28 10h14m51s 135.244s(Chemiluminescence).tif]

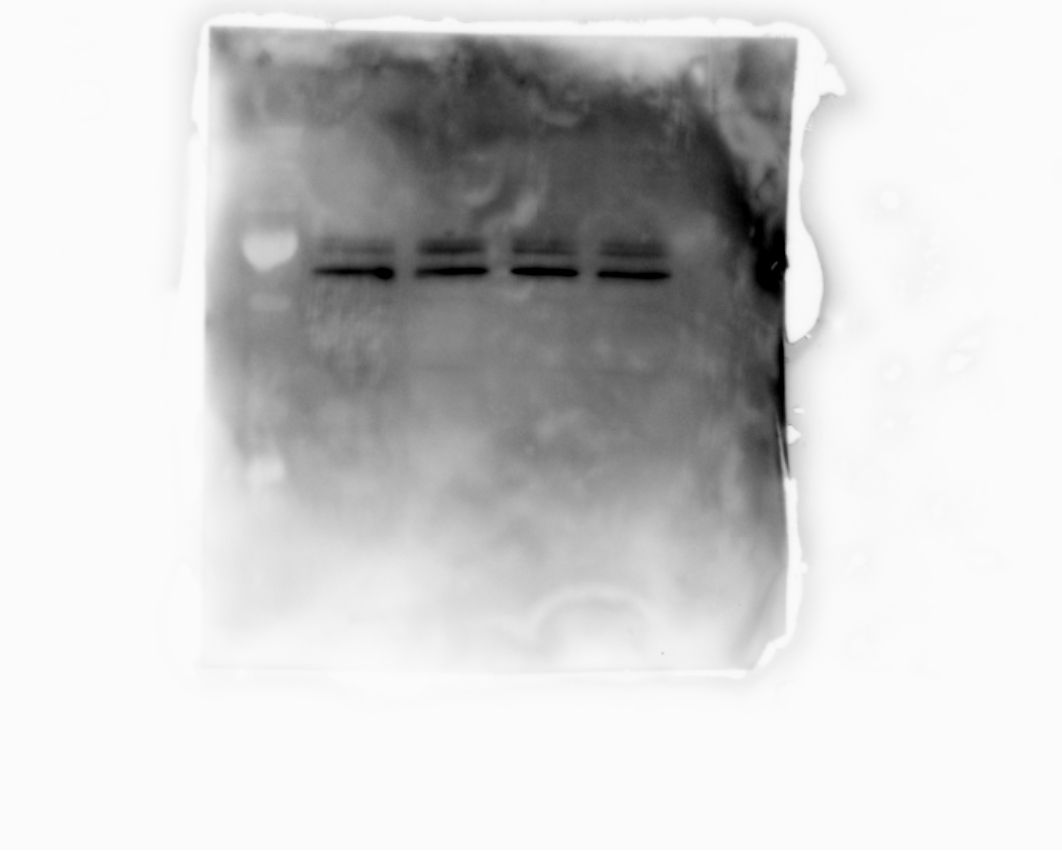

Supplement: Figure 4—figure supplement 1—source data 2. [file elife-89951-fig4-figsupp1-data2.zip › Figure 4-figure supplement 1-source data 2/LAMIN_Figure 4-figure supplement 1-source data 2/Versteeg 2023-07-28 13h26m38s 20.000s(Chemiluminescence).jpg]

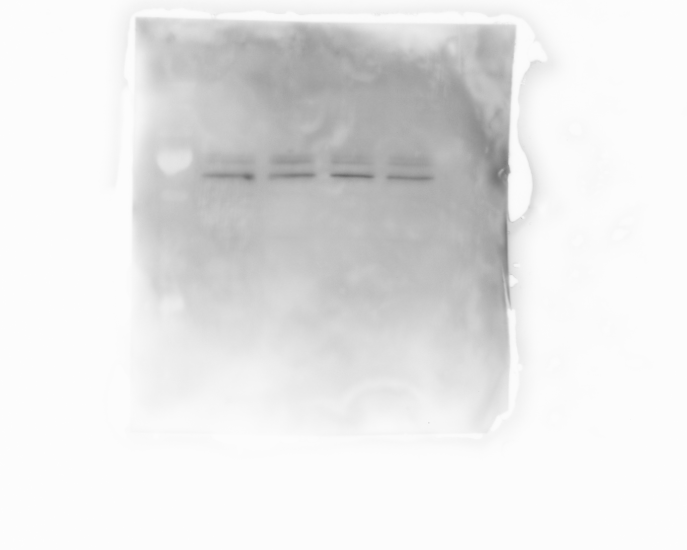

Supplement: Figure 4—figure supplement 1—source data 2. [file elife-89951-fig4-figsupp1-data2.zip › Figure 4-figure supplement 1-source data 2/LAMIN_Figure 4-figure supplement 1-source data 2/Versteeg 2023-07-28 13h26m38s 20.000s(Chemiluminescence).raw16.tif]

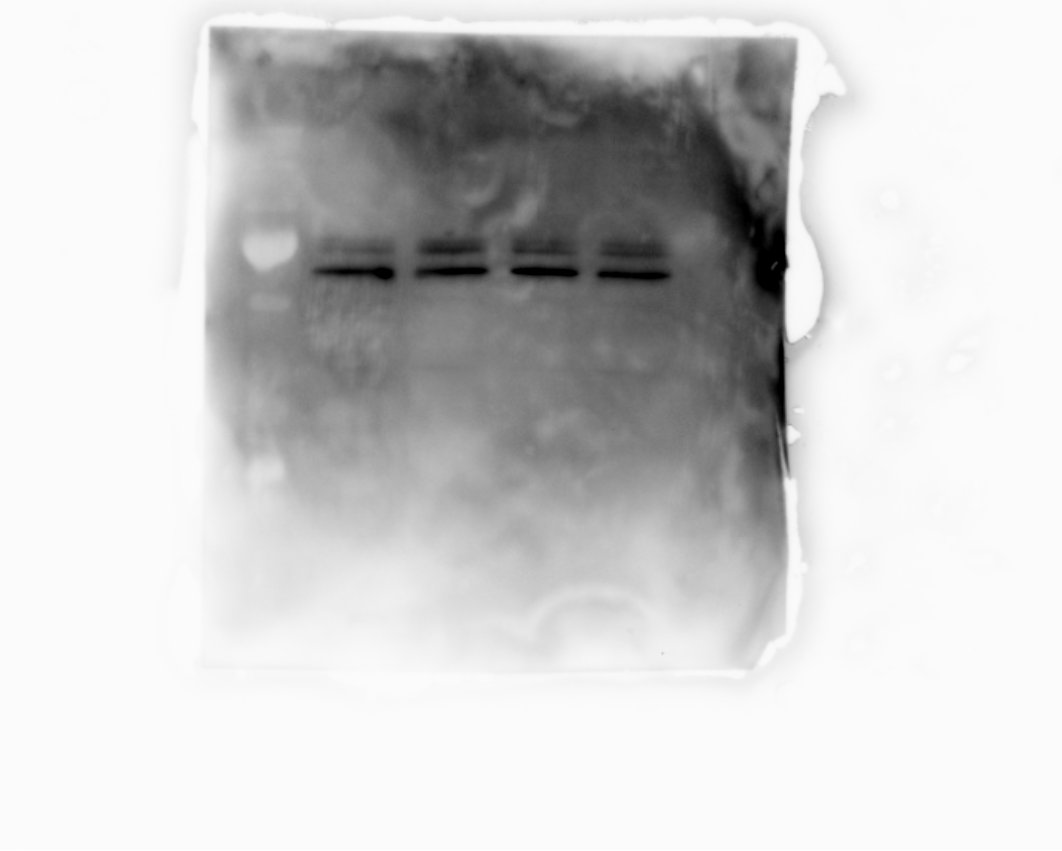

Supplement: Figure 4—figure supplement 1—source data 2. [file elife-89951-fig4-figsupp1-data2.zip › Figure 4-figure supplement 1-source data 2/LAMIN_Figure 4-figure supplement 1-source data 2/Versteeg 2023-07-28 13h26m38s 20.000s(Chemiluminescence).tif]

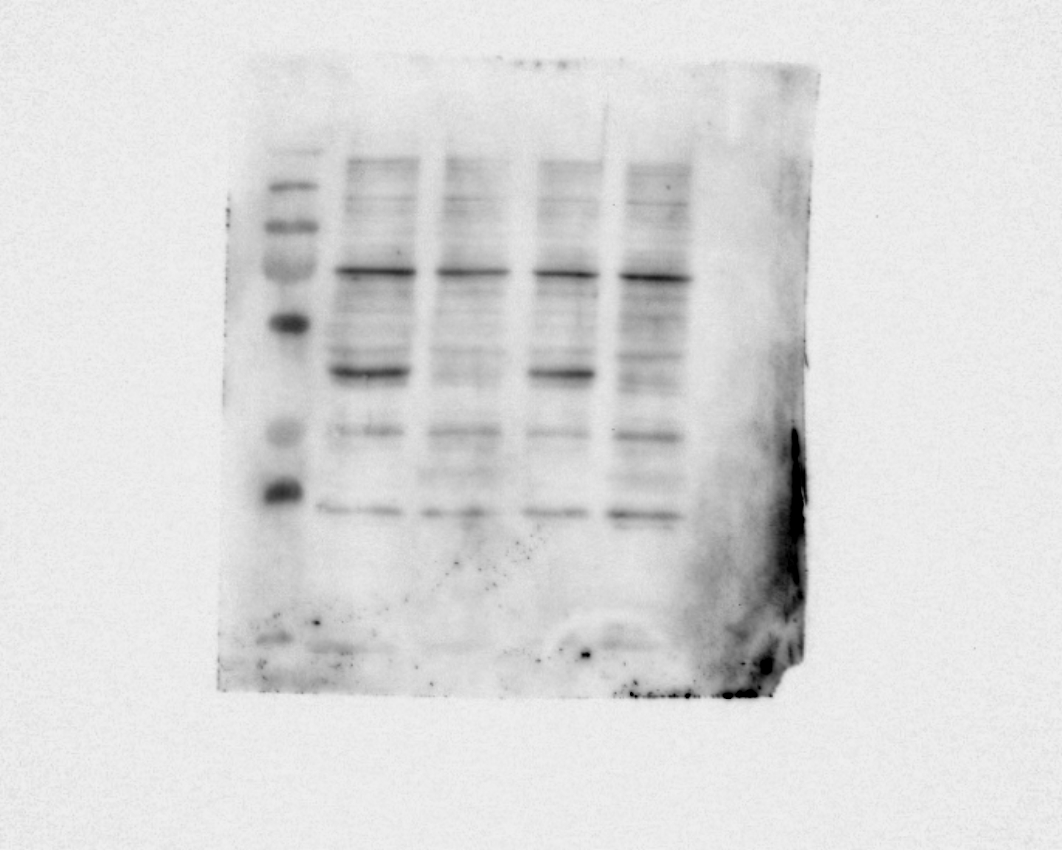

Supplement: Figure 4—figure supplement 1—source data 2. [file elife-89951-fig4-figsupp1-data2.zip › Figure 4-figure supplement 1-source data 2/SPOP_Figure 4-figure supplement 1-source data 2/Versteeg 2023-07-27 10h10m56s 55.918s(Chemiluminescence).jpg]

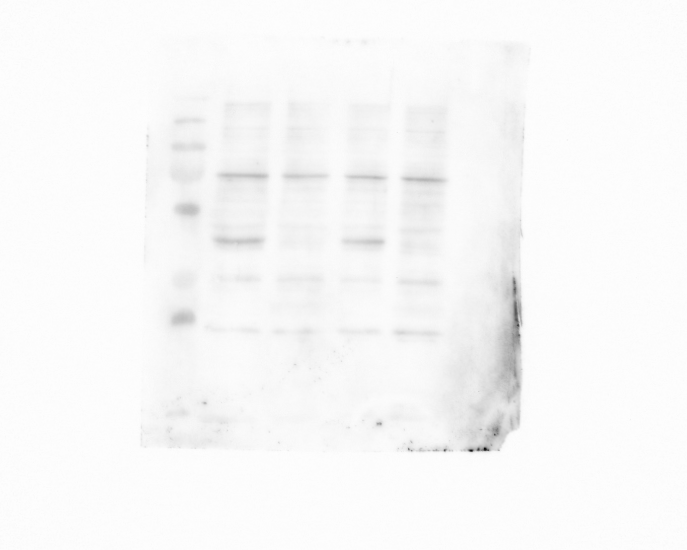

Supplement: Figure 4—figure supplement 1—source data 2. [file elife-89951-fig4-figsupp1-data2.zip › Figure 4-figure supplement 1-source data 2/SPOP_Figure 4-figure supplement 1-source data 2/Versteeg 2023-07-27 10h10m56s 55.918s(Chemiluminescence).raw16.tif]

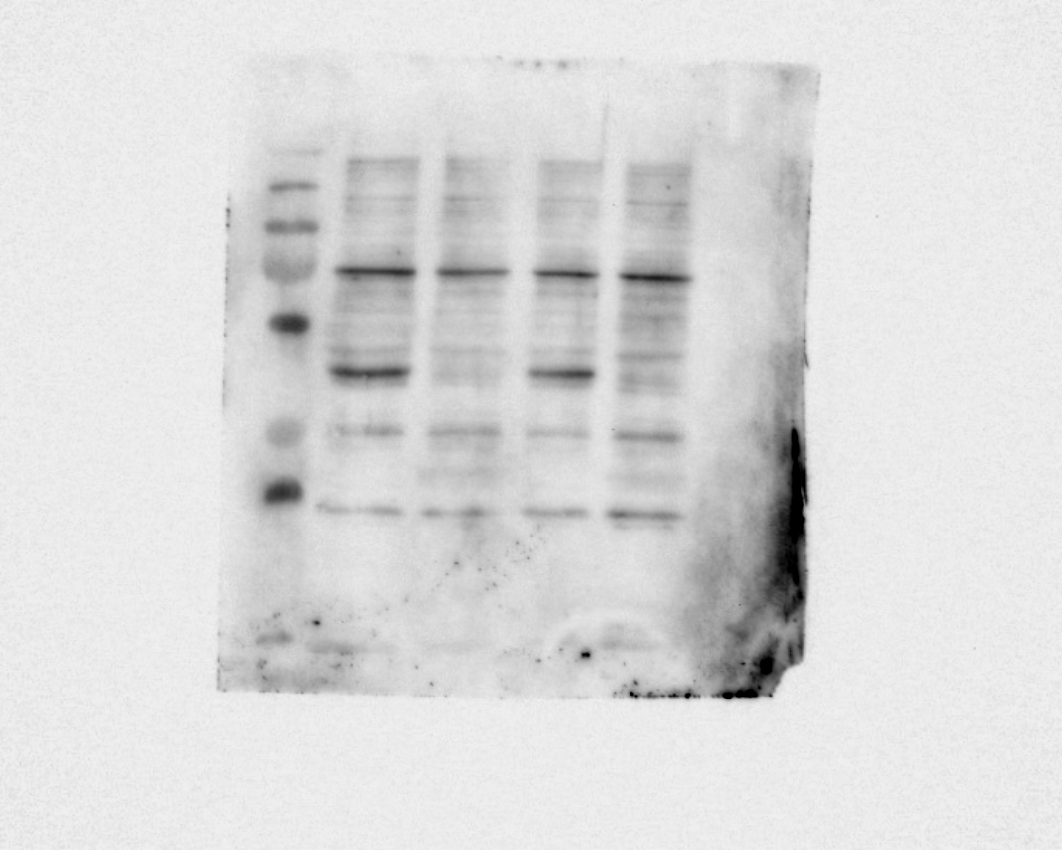

Supplement: Figure 4—figure supplement 1—source data 2. [file elife-89951-fig4-figsupp1-data2.zip › Figure 4-figure supplement 1-source data 2/SPOP_Figure 4-figure supplement 1-source data 2/Versteeg 2023-07-27 10h10m56s 55.918s(Chemiluminescence).tif]

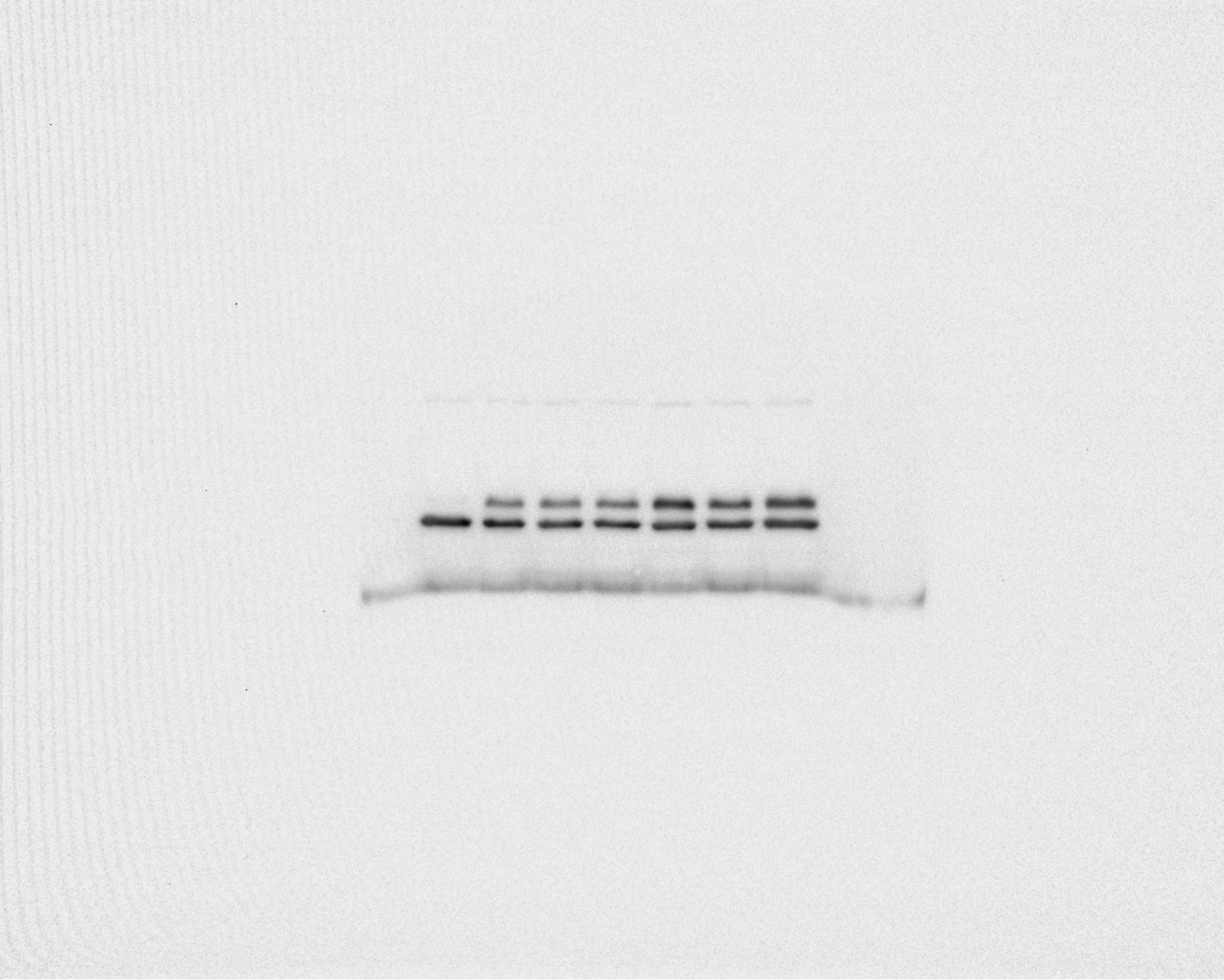

Supplement: Figure 4—figure supplement 1—source data 3. [file elife-89951-fig4-figsupp1-data3.zip › Figure 4-figure supplement 1-source data 3/ACTIN_Figure 4-figure supplement 1-source data 3/valentina 2022-05-25 13h33m15s(Chemiluminescence).jpg]

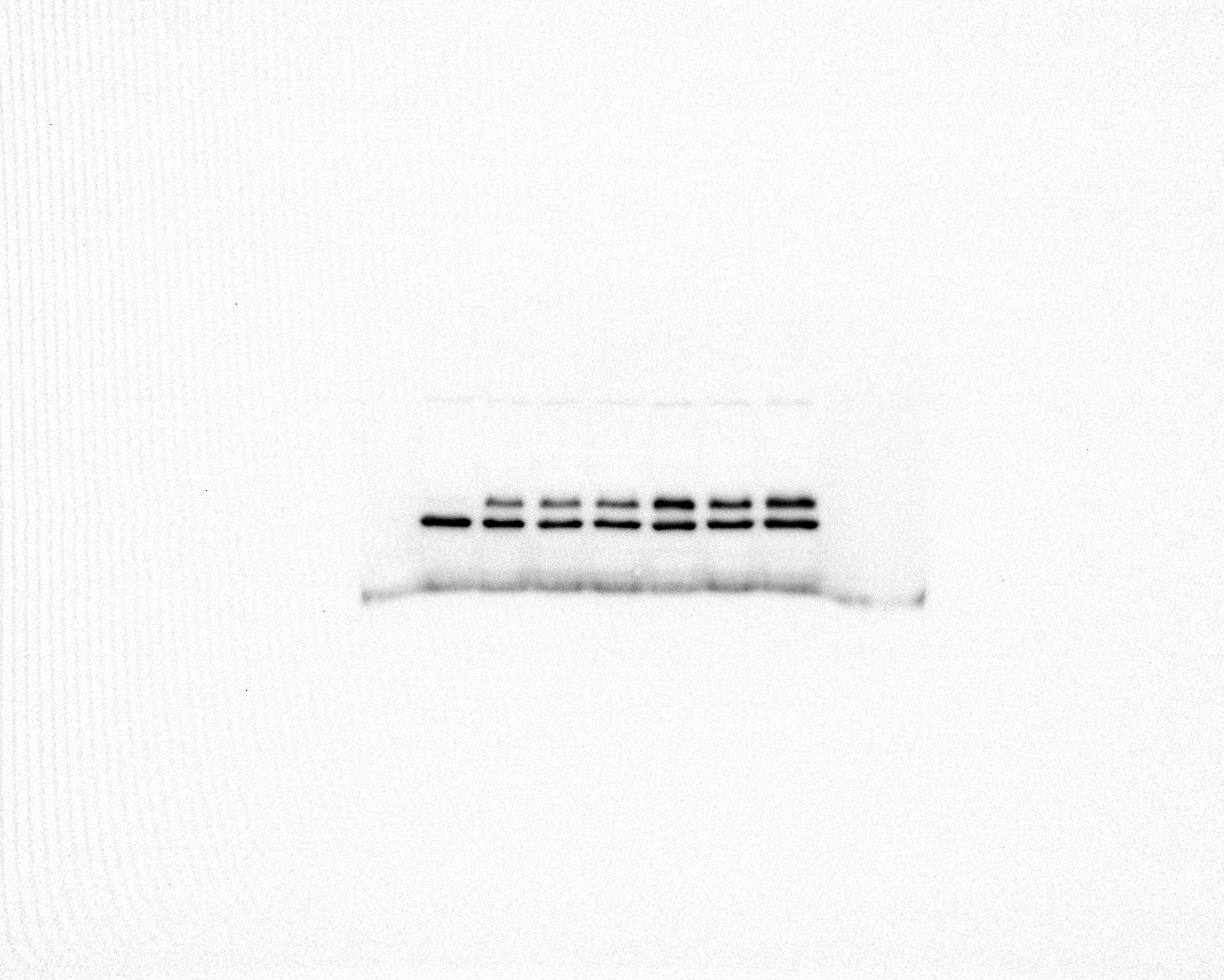

Supplement: Figure 4—figure supplement 1—source data 3. [file elife-89951-fig4-figsupp1-data3.zip › Figure 4-figure supplement 1-source data 3/ACTIN_Figure 4-figure supplement 1-source data 3/valentina 2022-05-25 13h33m15s(Chemiluminescence).raw16.tif]

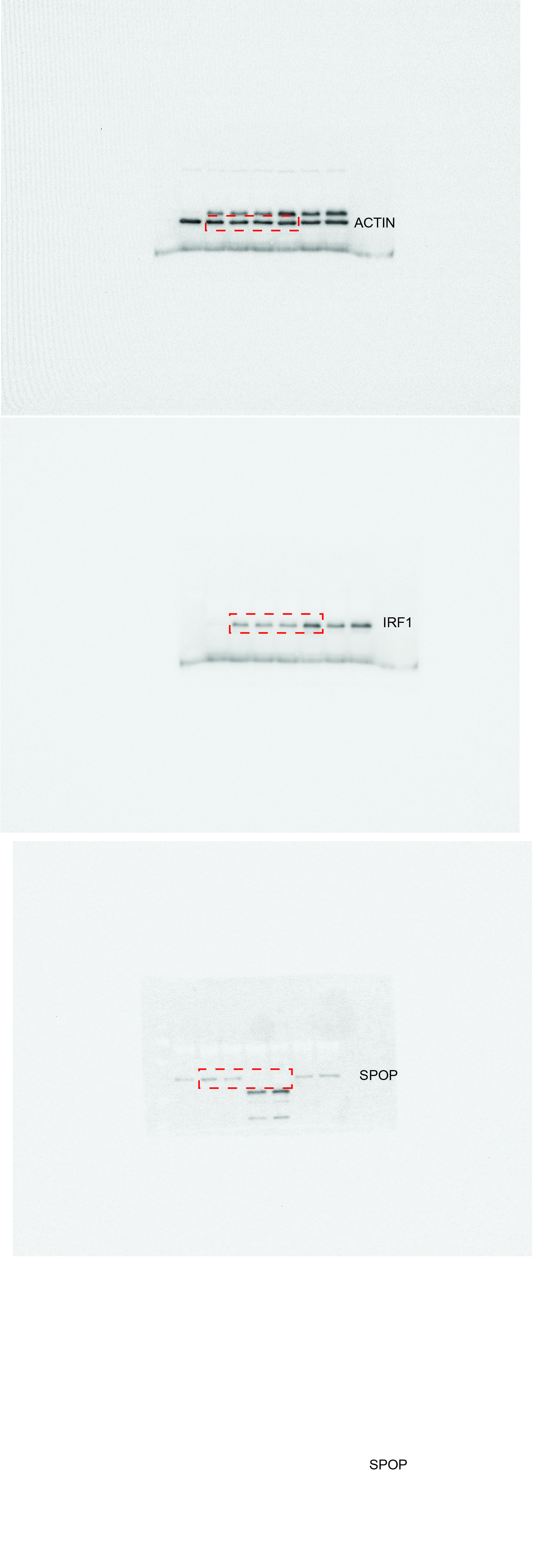

Supplement: Figure 4—figure supplement 1—source data 3. [file elife-89951-fig4-figsupp1-data3.zip › Figure 4-figure supplement 1-source data 3/Figure 4-figure supplement 1-source data 3.jpg]

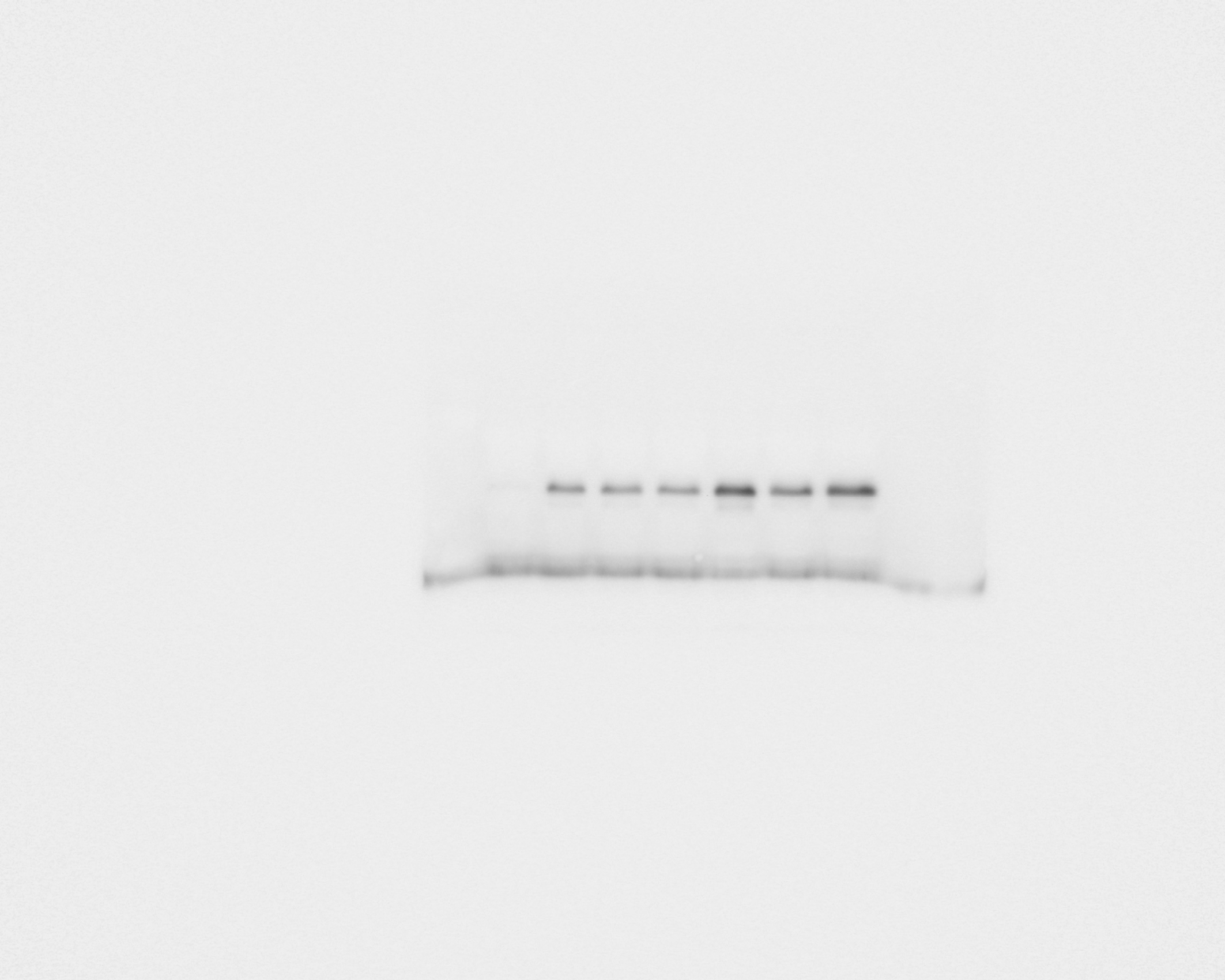

Supplement: Figure 4—figure supplement 1—source data 3. [file elife-89951-fig4-figsupp1-data3.zip › Figure 4-figure supplement 1-source data 3/IRF1_Figure 4-figure supplement 1-source data 3/Versteeg 2022-05-25 11h05m22s 7.048s(Chemiluminescence).jpg]

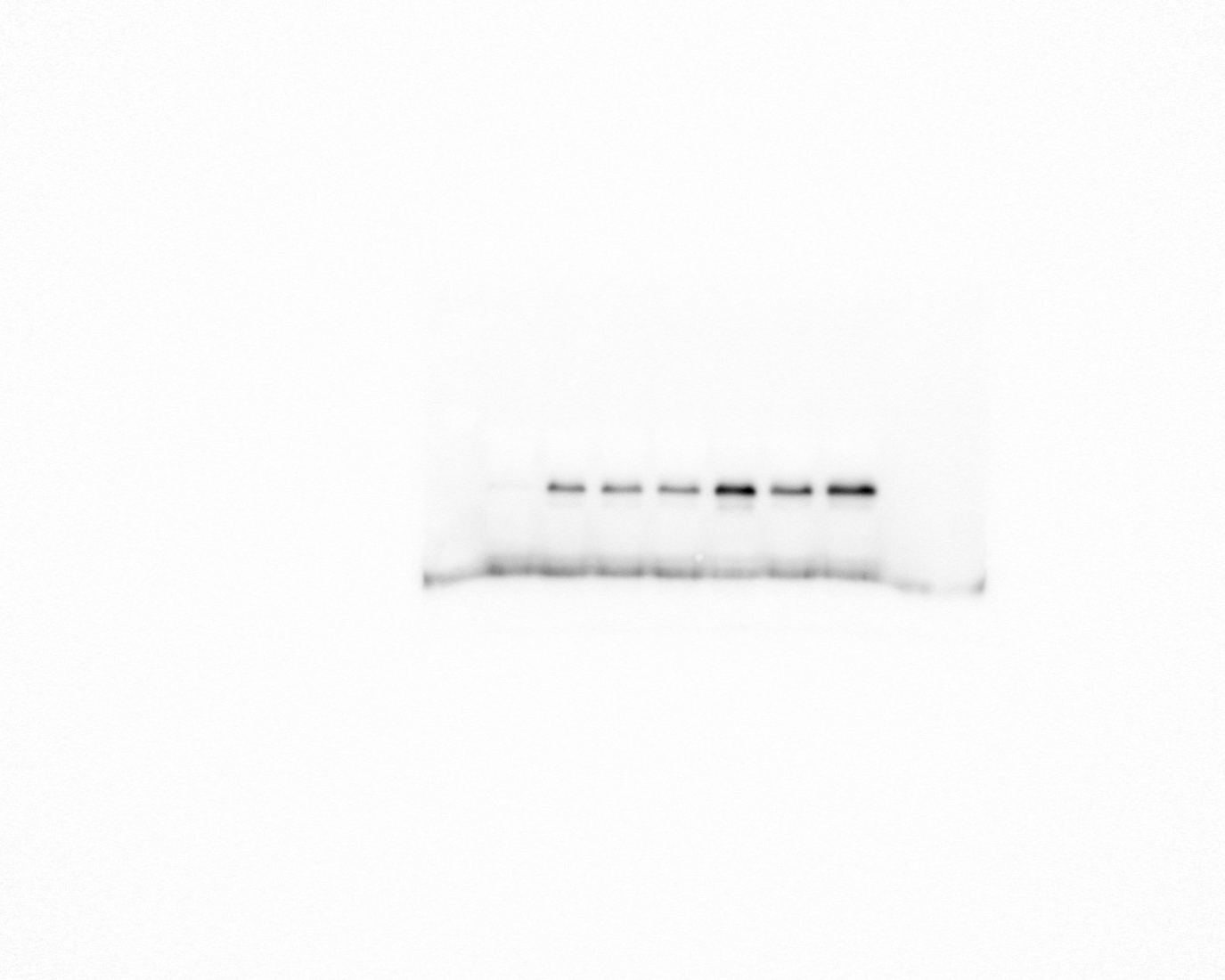

Supplement: Figure 4—figure supplement 1—source data 3. [file elife-89951-fig4-figsupp1-data3.zip › Figure 4-figure supplement 1-source data 3/IRF1_Figure 4-figure supplement 1-source data 3/Versteeg 2022-05-25 11h05m22s 7.048s(Chemiluminescence).raw16.tif]

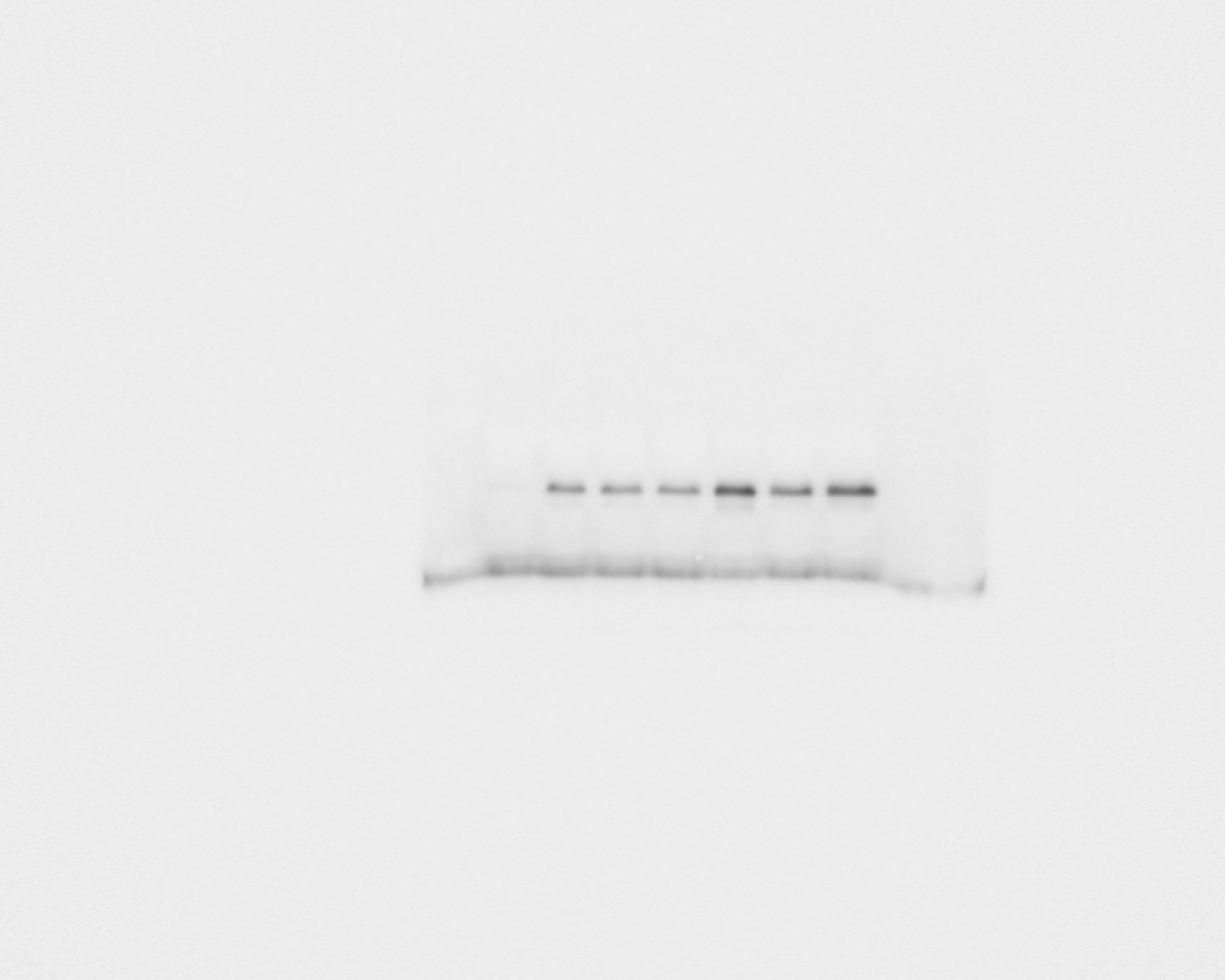

Supplement: Figure 4—figure supplement 1—source data 3. [file elife-89951-fig4-figsupp1-data3.zip › Figure 4-figure supplement 1-source data 3/IRF1_Figure 4-figure supplement 1-source data 3/Versteeg 2022-05-25 11h05m22s 7.048s(Chemiluminescence).tif]

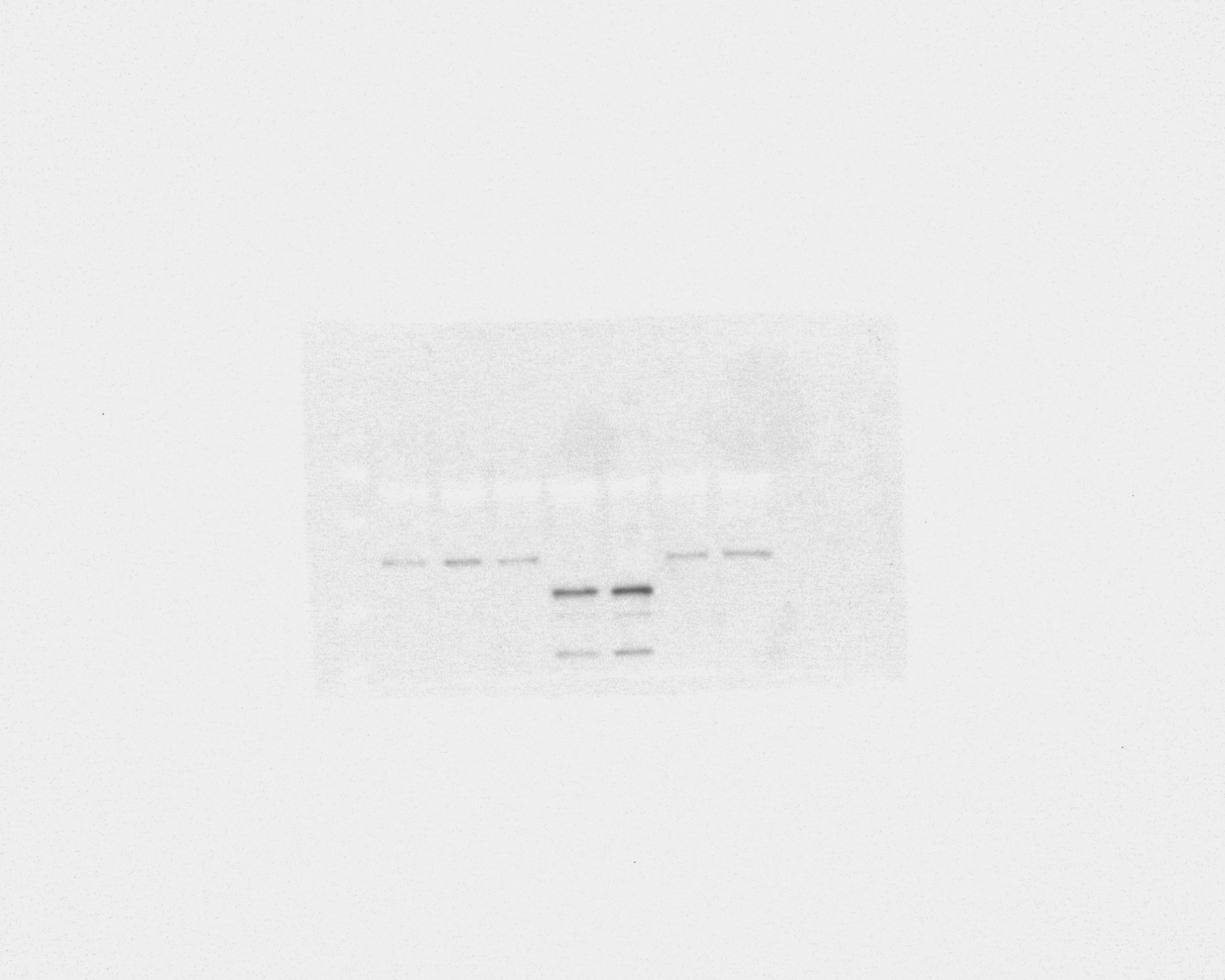

Supplement: Figure 4—figure supplement 1—source data 3. [file elife-89951-fig4-figsupp1-data3.zip › Figure 4-figure supplement 1-source data 3/SPOP_Figure 4-figure supplement 1-source data 3/Versteeg 2022-05-20 10h38m55s 299.990s(Chemiluminescence).jpg]

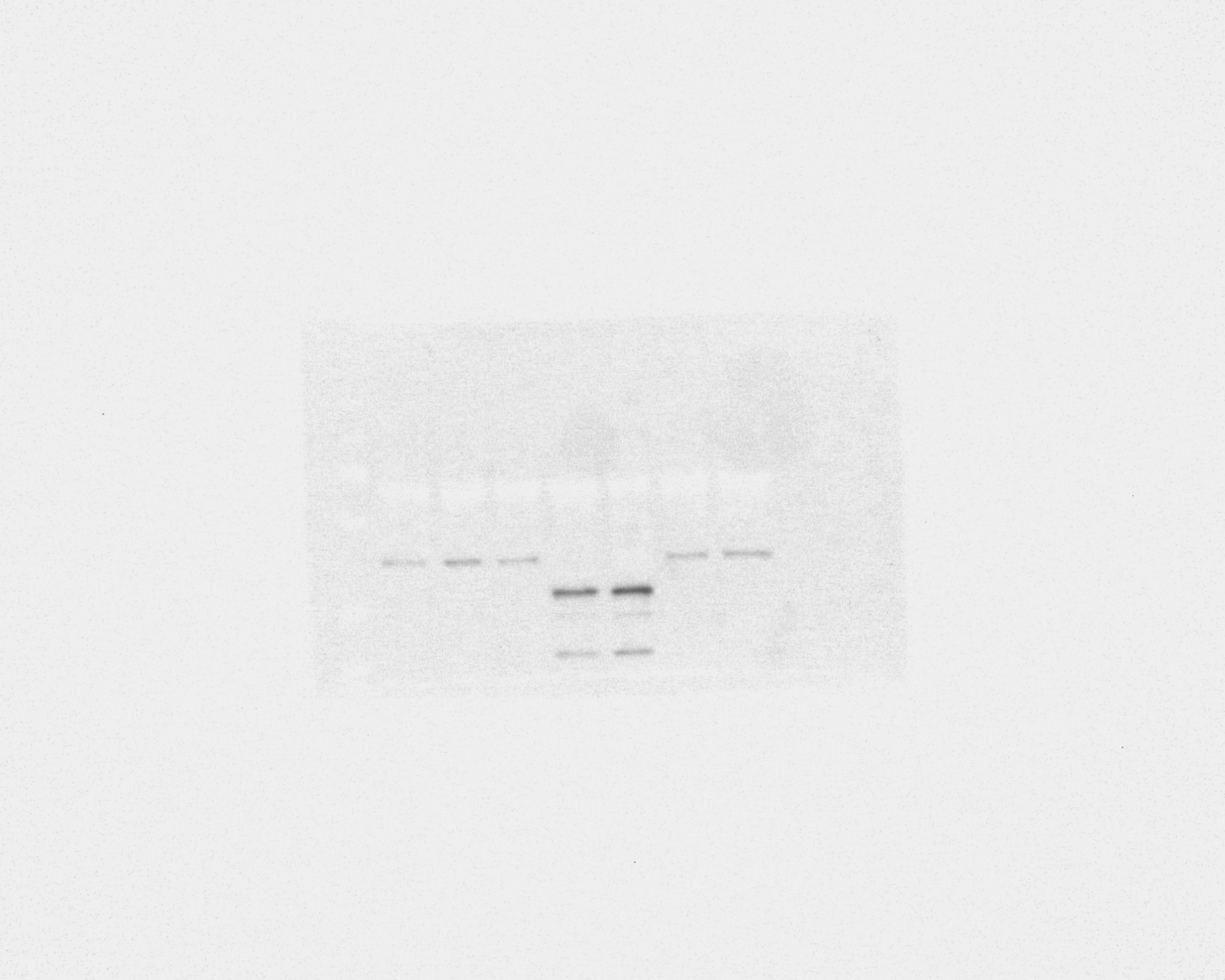

Supplement: Figure 4—figure supplement 1—source data 3. [file elife-89951-fig4-figsupp1-data3.zip › Figure 4-figure supplement 1-source data 3/SPOP_Figure 4-figure supplement 1-source data 3/Versteeg 2022-05-20 10h38m55s 299.990s(Chemiluminescence).tif]

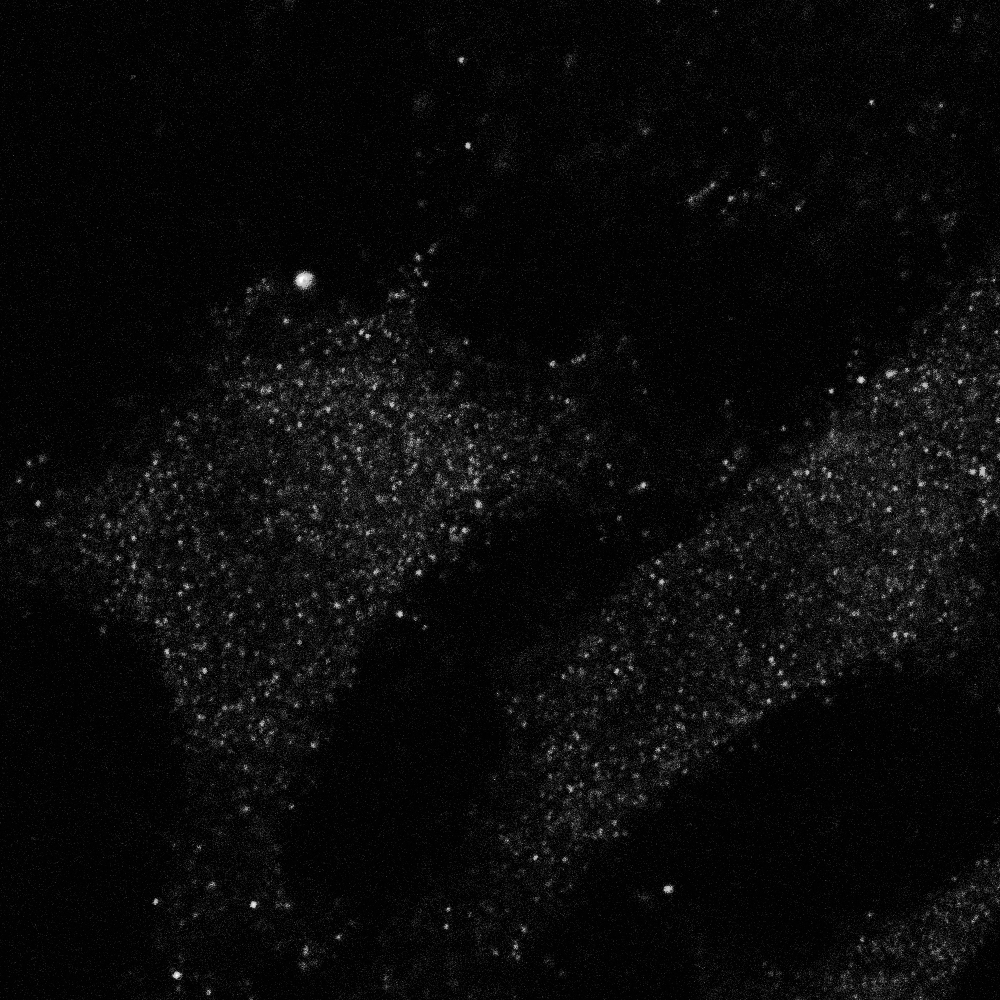

Supplement: Figure 4—figure supplement 2—source data 1. [file elife-89951-fig4-figsupp2-data1.zip › Figure 4-figure supplement 2-source data 1/IRF1_UNTR_Figure 4-figure supplement 2-source data 1.tif]

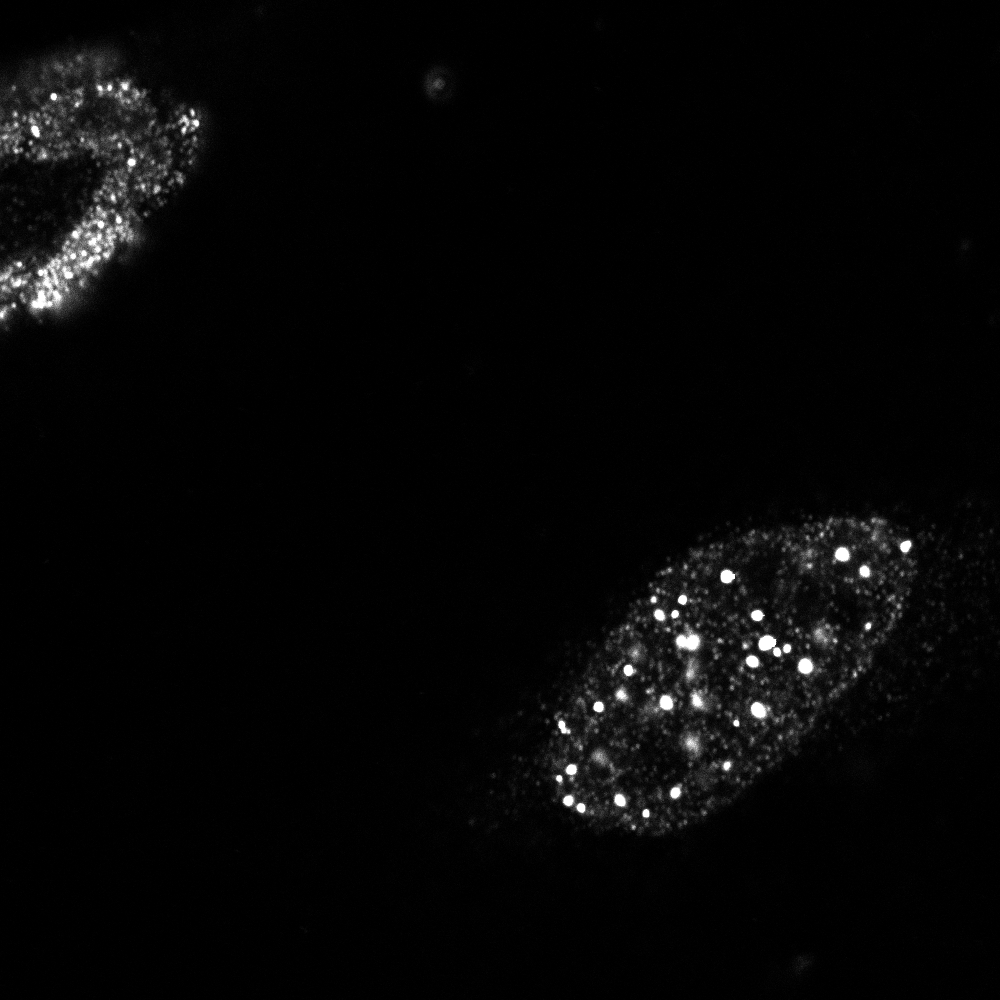

Supplement: Figure 4—figure supplement 2—source data 1. [file elife-89951-fig4-figsupp2-data1.zip › Figure 4-figure supplement 2-source data 1/IRF1+SPOP_EPOX_Figure 4-figure supplement 2-source data 1.tif]

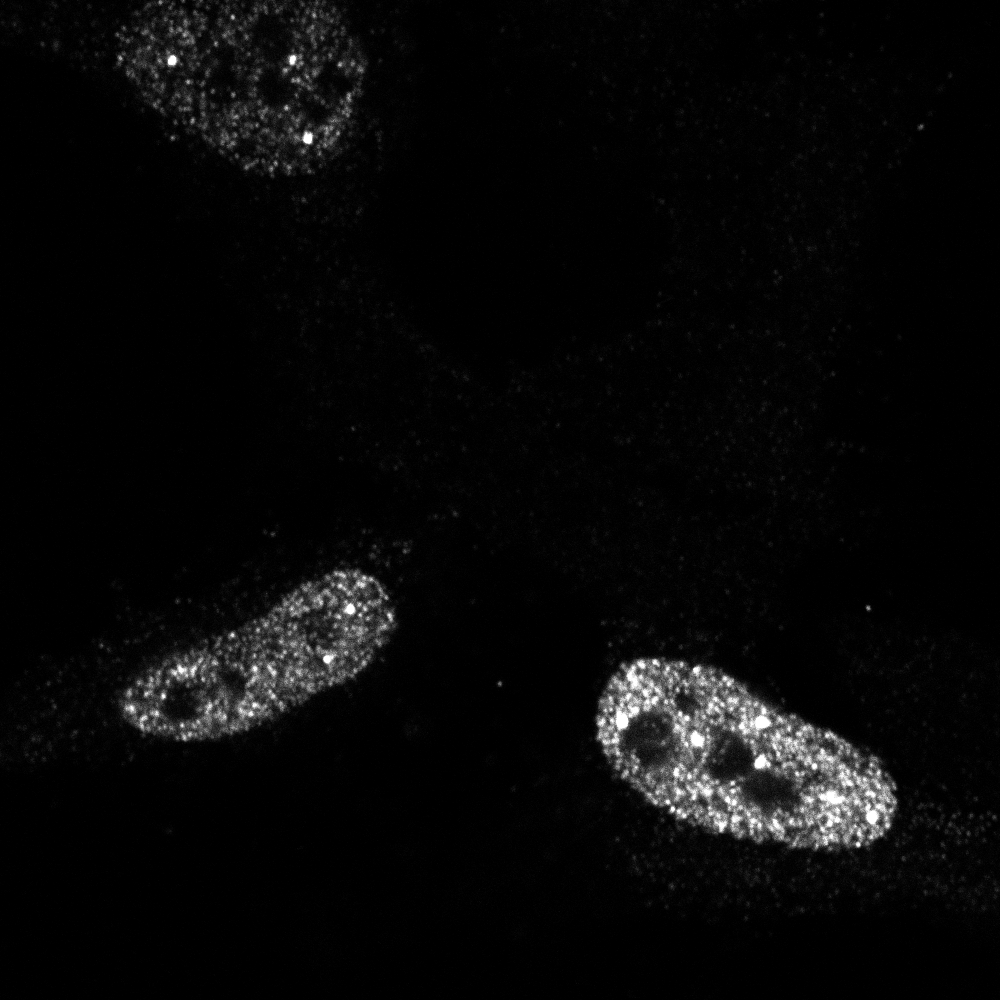

Supplement: Figure 4—figure supplement 2—source data 1. [file elife-89951-fig4-figsupp2-data1.zip › Figure 4-figure supplement 2-source data 1/SPOP_UNTR_Figure 4-figure supplement 2-source data 1.tif]

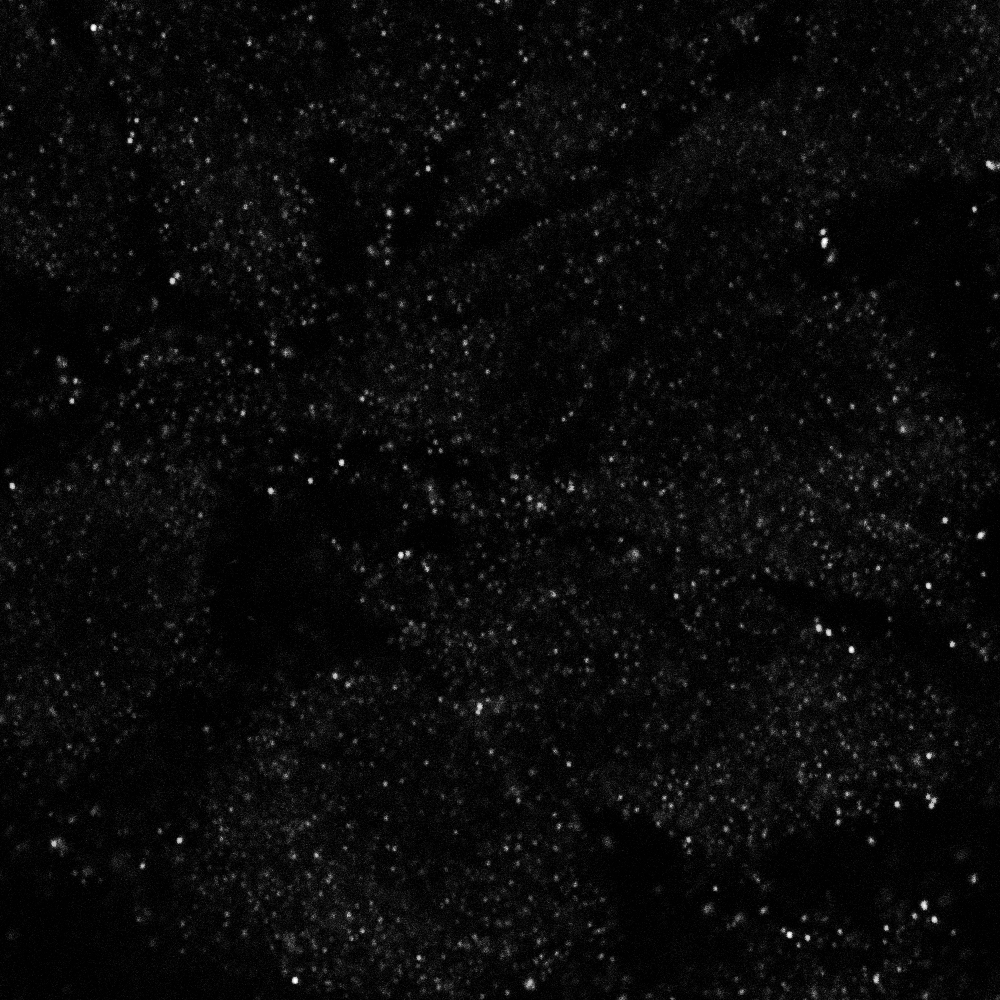

Supplement: Figure 4—figure supplement 2—source data 1. [file elife-89951-fig4-figsupp2-data1.zip › Figure 4-figure supplement 2-source data 1/Untransfected_UNTR_Figure 4-figure supplement 2-source data 1.tif]

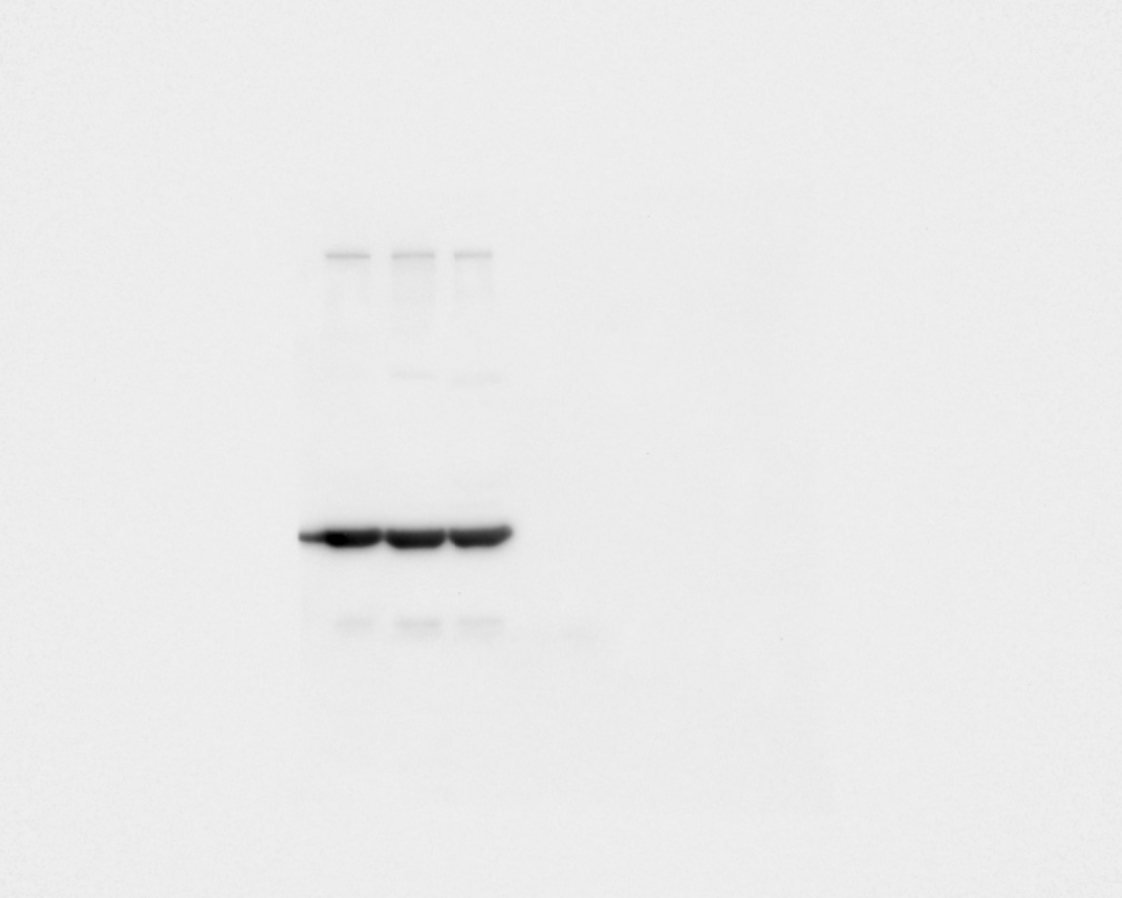

Supplement: Figure 5—figure supplement 1—source data 1. [file elife-89951-fig5-figsupp1-data1.zip › Figure 5-figure supplement 1-source data 1/ACTIN_Figure 5-figure supplement 1-source data 1/Versteeg 2023-03-13 14h18m15s 13.204s(Chemiluminescence).jpg]

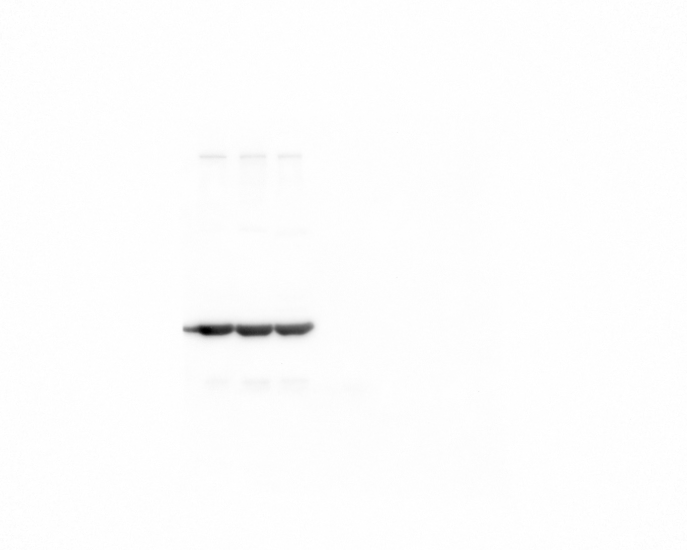

Supplement: Figure 5—figure supplement 1—source data 1. [file elife-89951-fig5-figsupp1-data1.zip › Figure 5-figure supplement 1-source data 1/ACTIN_Figure 5-figure supplement 1-source data 1/Versteeg 2023-03-13 14h18m15s 13.204s(Chemiluminescence).raw16.tif]

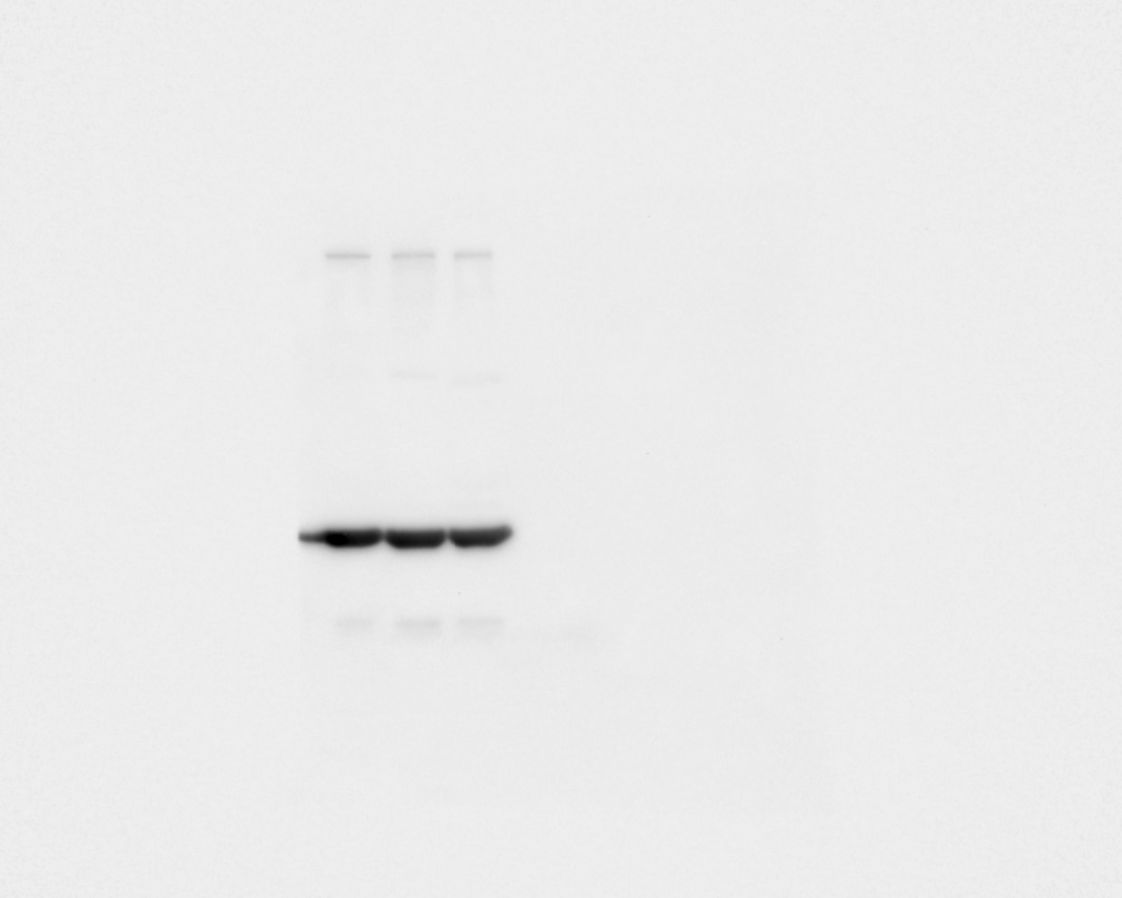

Supplement: Figure 5—figure supplement 1—source data 1. [file elife-89951-fig5-figsupp1-data1.zip › Figure 5-figure supplement 1-source data 1/ACTIN_Figure 5-figure supplement 1-source data 1/Versteeg 2023-03-13 14h18m15s 13.204s(Chemiluminescence).tif]

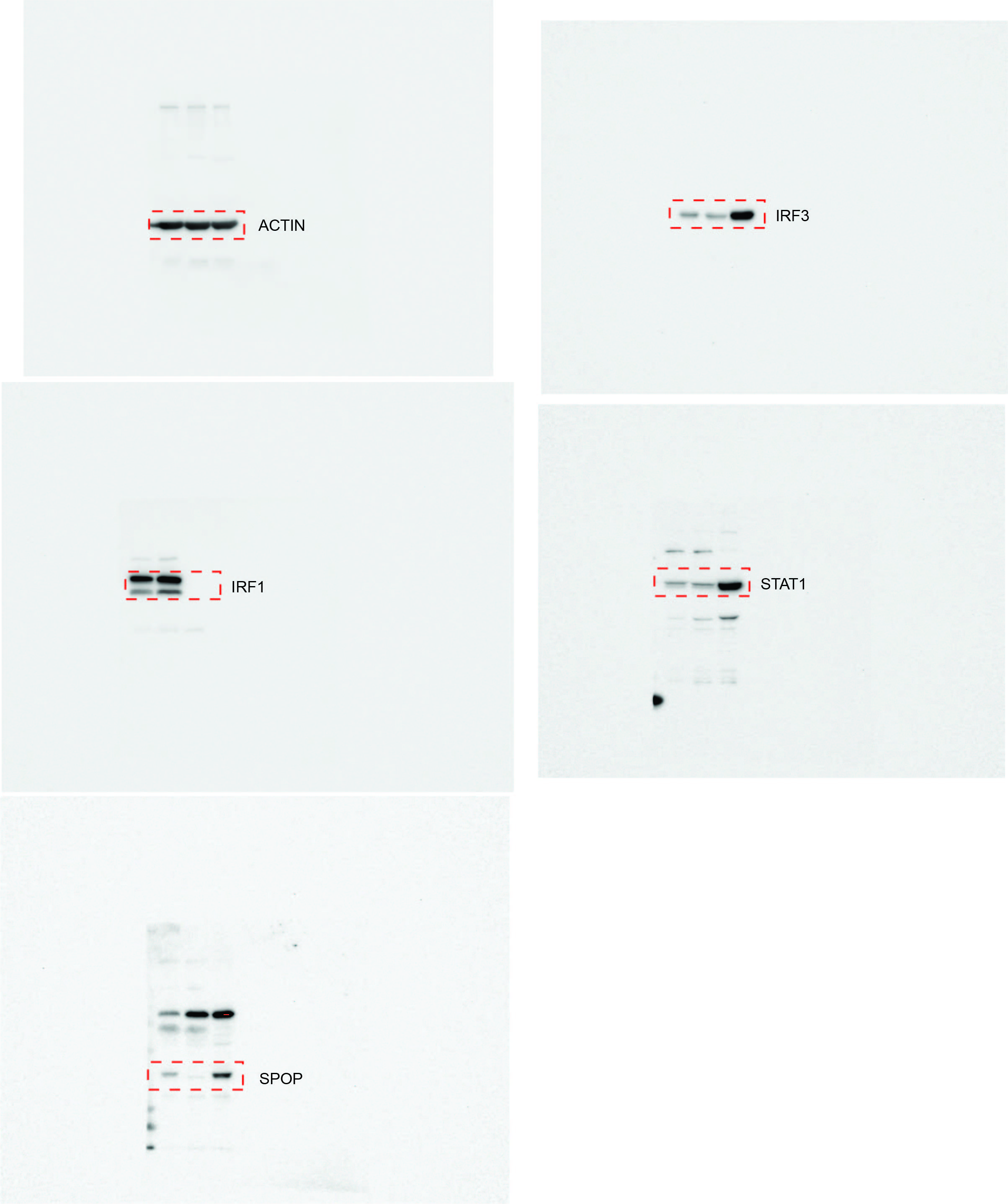

Supplement: Figure 5—figure supplement 1—source data 1. [file elife-89951-fig5-figsupp1-data1.zip › Figure 5-figure supplement 1-source data 1/Figure 5-figure supplement 1-source data 1.jpg]

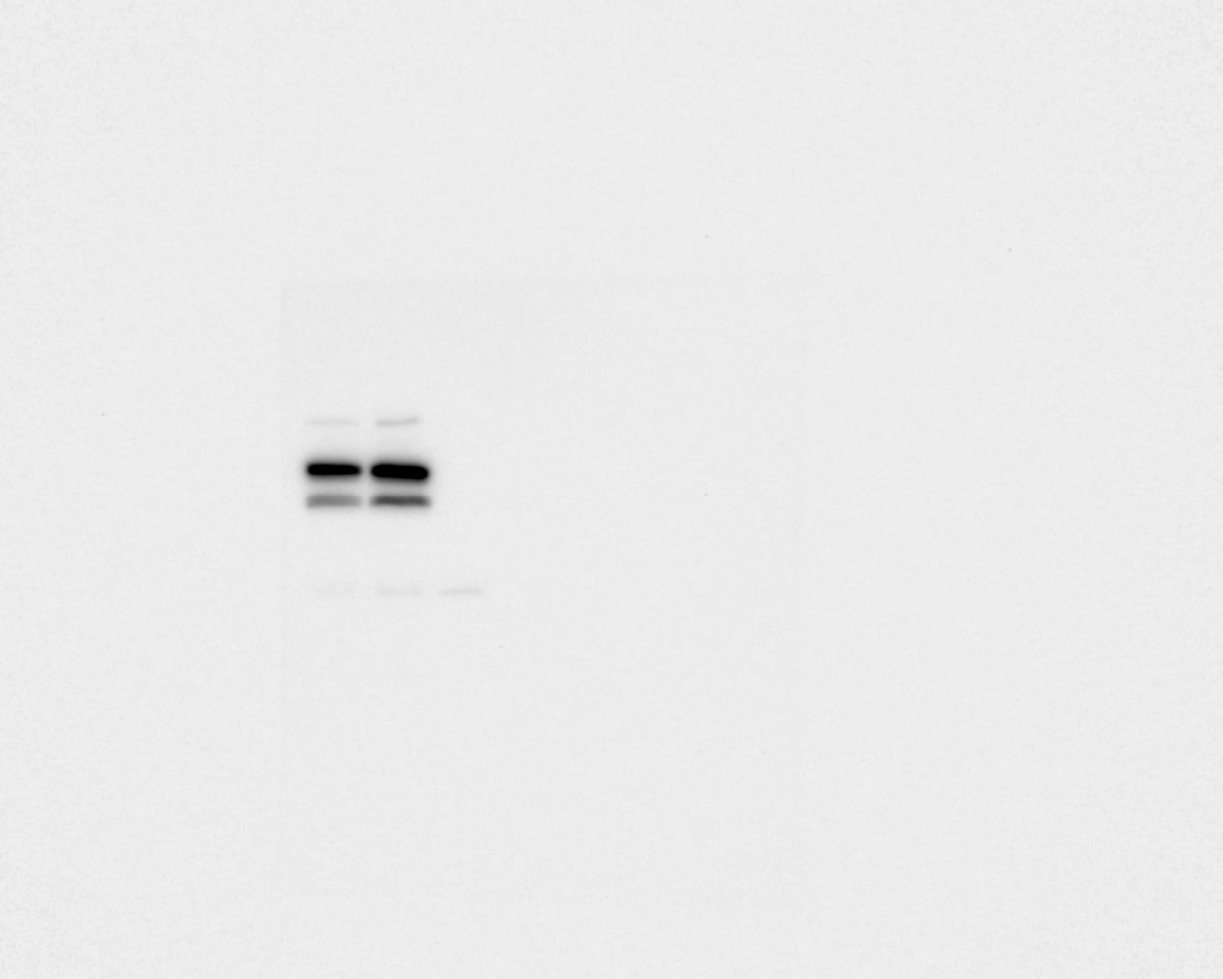

Supplement: Figure 5—figure supplement 1—source data 1. [file elife-89951-fig5-figsupp1-data1.zip › Figure 5-figure supplement 1-source data 1/IRF1_Figure 5-figure supplement 1-source data 1/Versteeg 2023-03-10 09h26m41s 25.408s(Chemiluminescence).jpg]

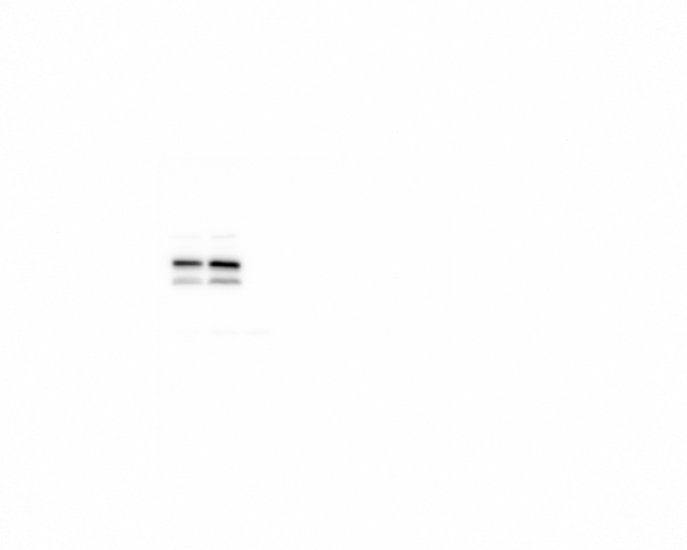

Supplement: Figure 5—figure supplement 1—source data 1. [file elife-89951-fig5-figsupp1-data1.zip › Figure 5-figure supplement 1-source data 1/IRF1_Figure 5-figure supplement 1-source data 1/Versteeg 2023-03-10 09h26m41s 25.408s(Chemiluminescence).raw16.tif]

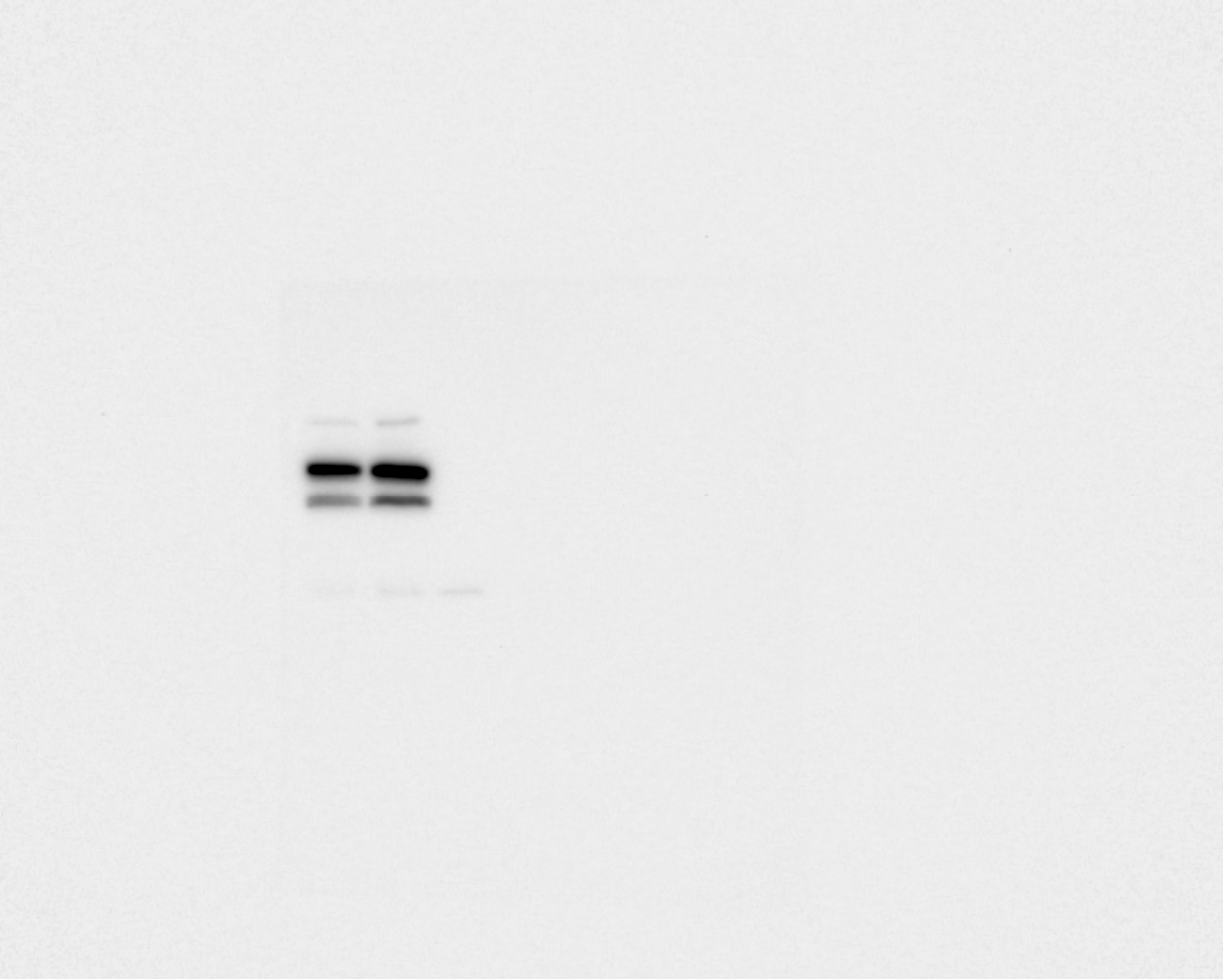

Supplement: Figure 5—figure supplement 1—source data 1. [file elife-89951-fig5-figsupp1-data1.zip › Figure 5-figure supplement 1-source data 1/IRF1_Figure 5-figure supplement 1-source data 1/Versteeg 2023-03-10 09h26m41s 25.408s(Chemiluminescence).tif]

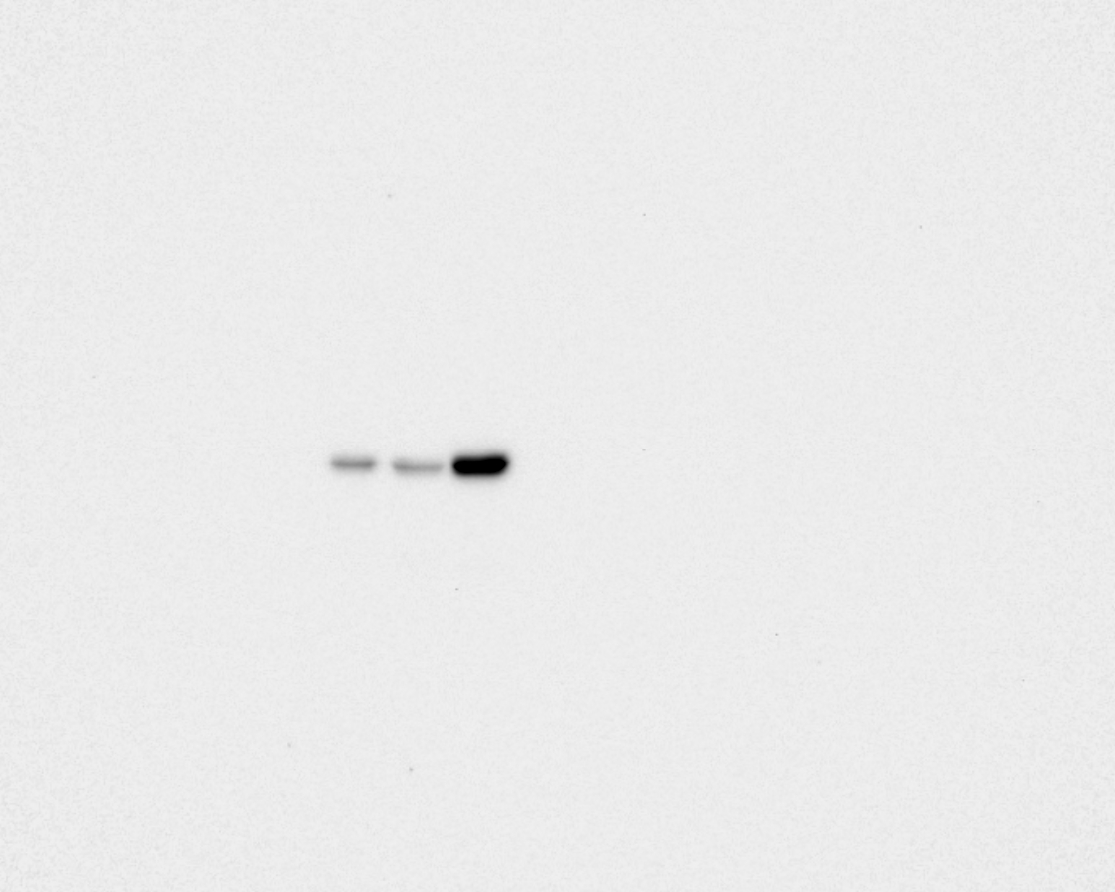

Supplement: Figure 5—figure supplement 1—source data 1. [file elife-89951-fig5-figsupp1-data1.zip › Figure 5-figure supplement 1-source data 1/IRF3_Figure 5-figure supplement 1-source data 1/Versteeg 2023-03-13 09h57m18s 43.714s(Chemiluminescence).jpg]

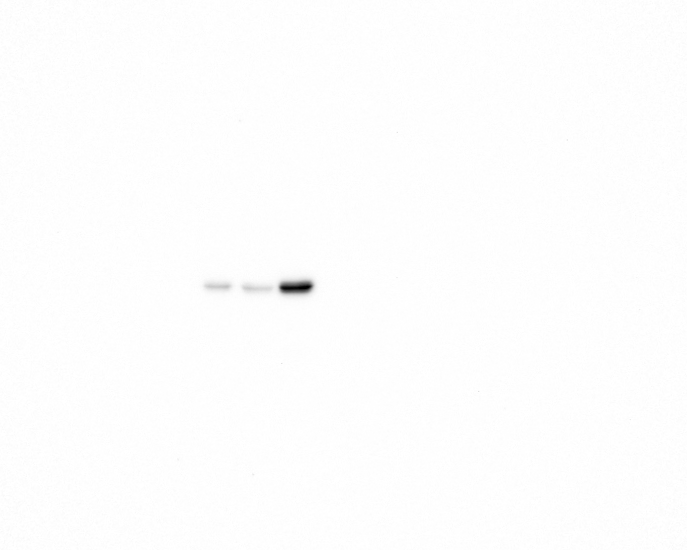

Supplement: Figure 5—figure supplement 1—source data 1. [file elife-89951-fig5-figsupp1-data1.zip › Figure 5-figure supplement 1-source data 1/IRF3_Figure 5-figure supplement 1-source data 1/Versteeg 2023-03-13 09h57m18s 43.714s(Chemiluminescence).raw16.tif]

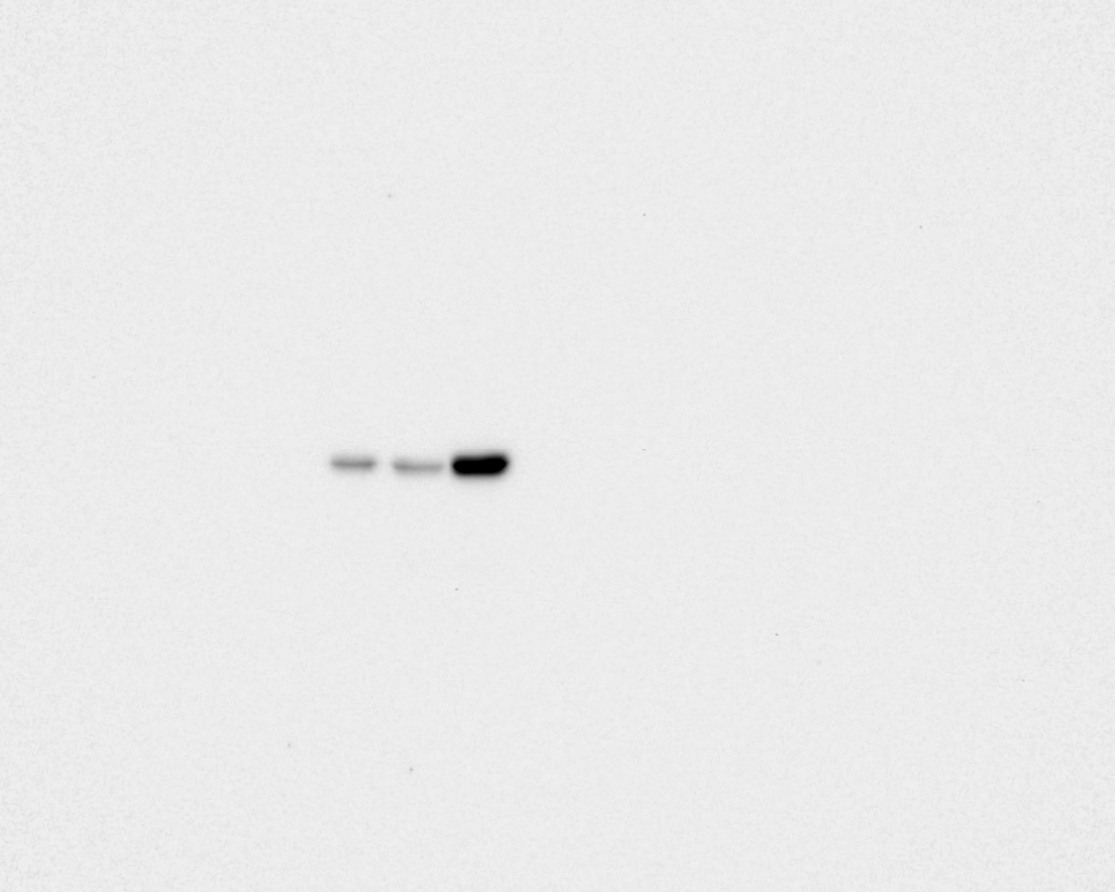

Supplement: Figure 5—figure supplement 1—source data 1. [file elife-89951-fig5-figsupp1-data1.zip › Figure 5-figure supplement 1-source data 1/IRF3_Figure 5-figure supplement 1-source data 1/Versteeg 2023-03-13 09h57m18s 43.714s(Chemiluminescence).tif]

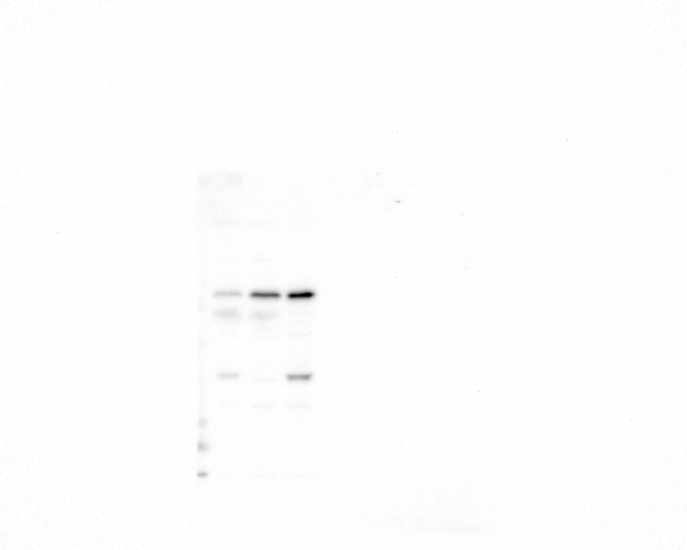

Supplement: Figure 5—figure supplement 1—source data 1. [file elife-89951-fig5-figsupp1-data1.zip › Figure 5-figure supplement 1-source data 1/SPOP_Figure 5-figure supplement 1-source data 1/Versteeg 2023-03-09 13h31m26s 116.938s(Chemiluminescence).raw16.tif]

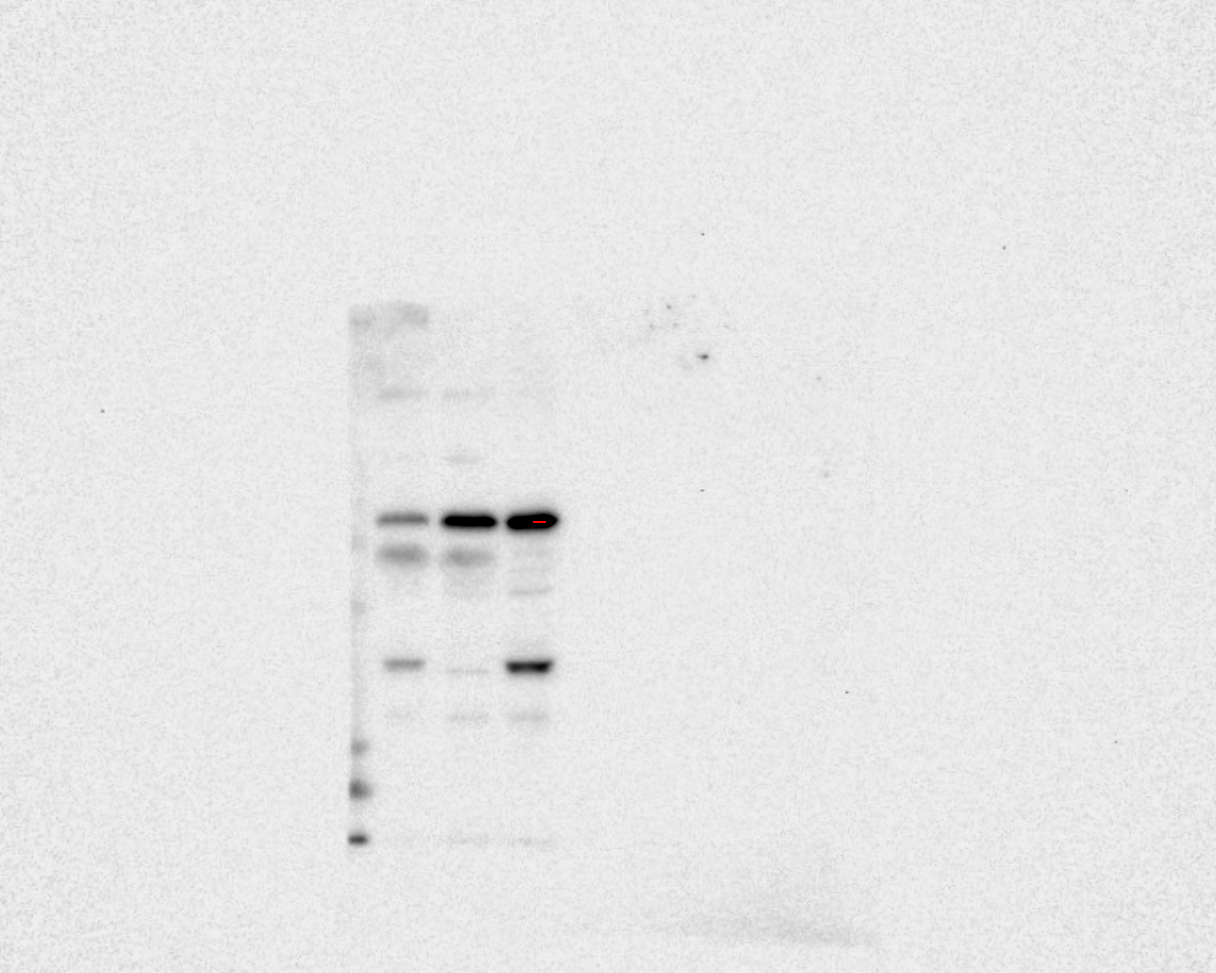

Supplement: Figure 5—figure supplement 1—source data 1. [file elife-89951-fig5-figsupp1-data1.zip › Figure 5-figure supplement 1-source data 1/SPOP_Figure 5-figure supplement 1-source data 1/Versteeg 2023-03-09 13h31m26s 116.938s(Chemiluminescence).tif]

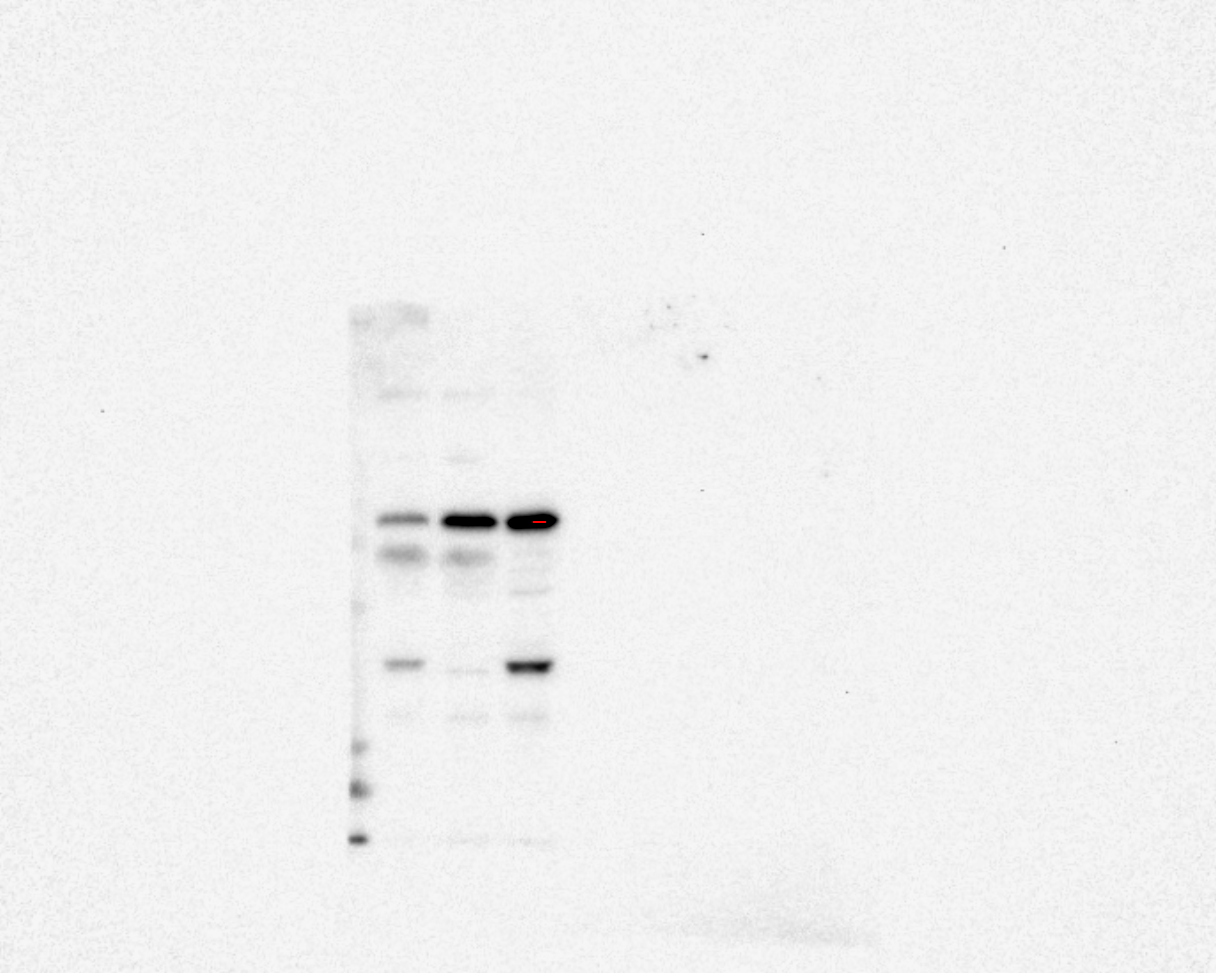

Supplement: Figure 5—figure supplement 1—source data 1. [file elife-89951-fig5-figsupp1-data1.zip › Figure 5-figure supplement 1-source data 1/SPOP_Figure 5-figure supplement 1-source data 1/Versteeg 2023-03-09 13h31m26s 116.938s(Chemiluminescence)_Adj.tif]

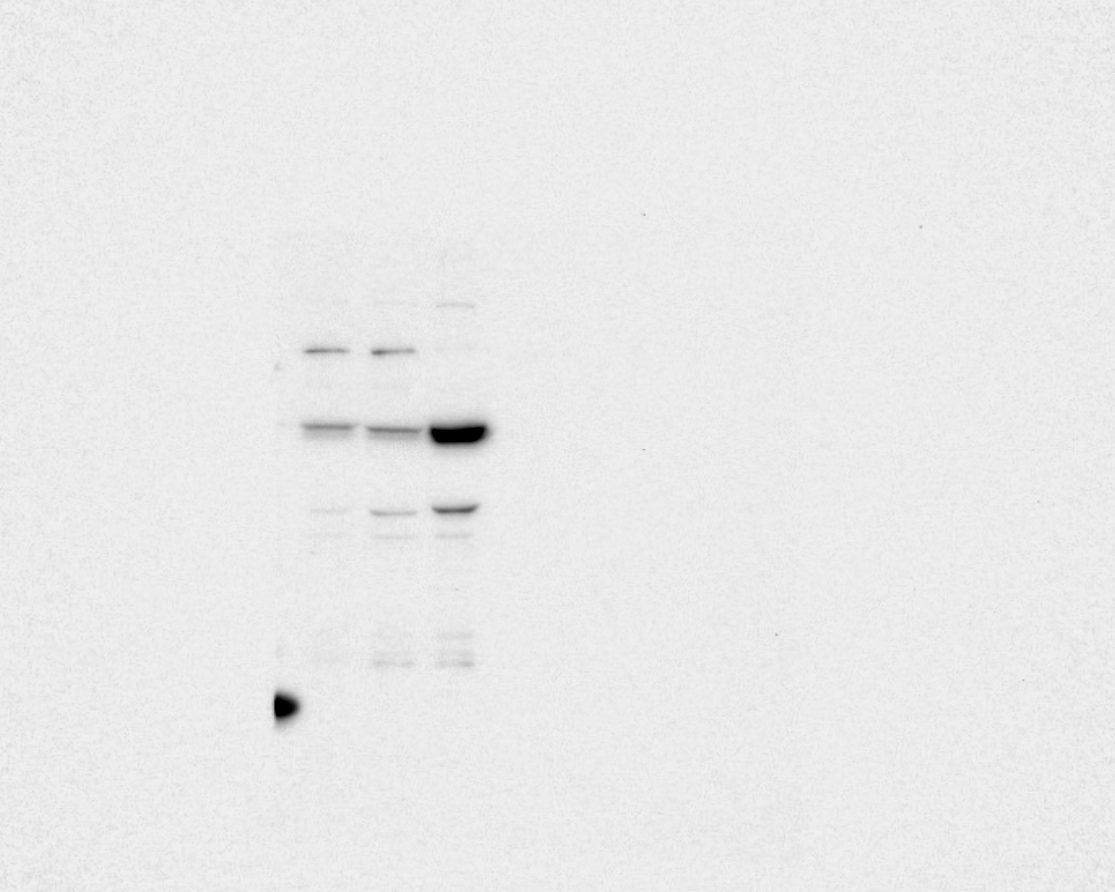

Supplement: Figure 5—figure supplement 1—source data 1. [file elife-89951-fig5-figsupp1-data1.zip › Figure 5-figure supplement 1-source data 1/STAT1_Figure 5-figure supplement 1-source data 1/Versteeg 2023-03-11 15h21m34s 68.122s(Chemiluminescence).jpg]

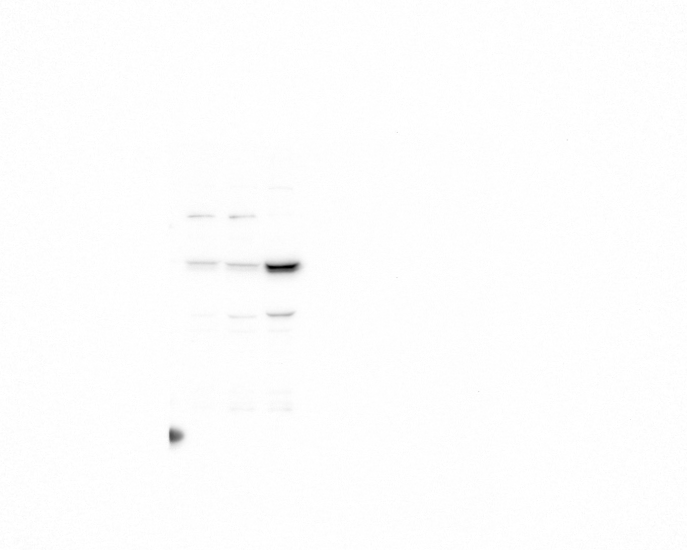

Supplement: Figure 5—figure supplement 1—source data 1. [file elife-89951-fig5-figsupp1-data1.zip › Figure 5-figure supplement 1-source data 1/STAT1_Figure 5-figure supplement 1-source data 1/Versteeg 2023-03-11 15h21m34s 68.122s(Chemiluminescence).raw16.tif]

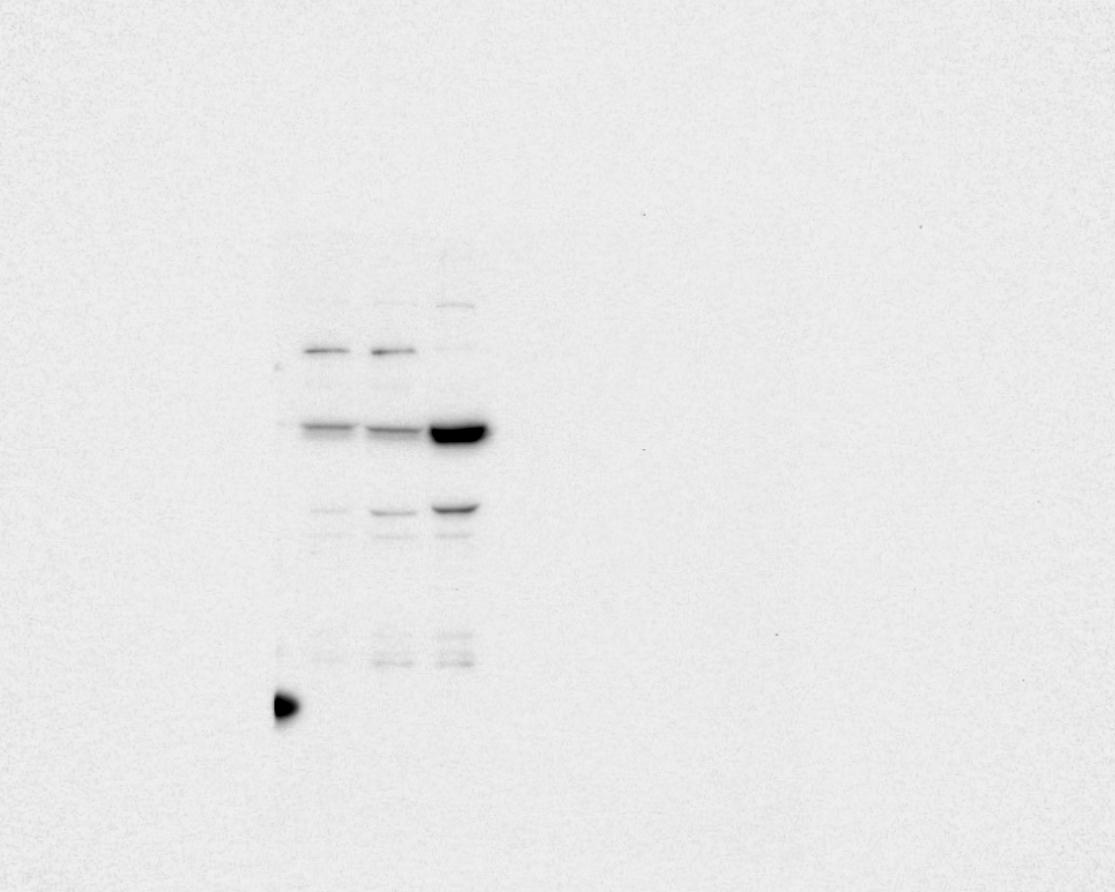

Supplement: Figure 5—figure supplement 1—source data 1. [file elife-89951-fig5-figsupp1-data1.zip › Figure 5-figure supplement 1-source data 1/STAT1_Figure 5-figure supplement 1-source data 1/Versteeg 2023-03-11 15h21m34s 68.122s(Chemiluminescence).tif]

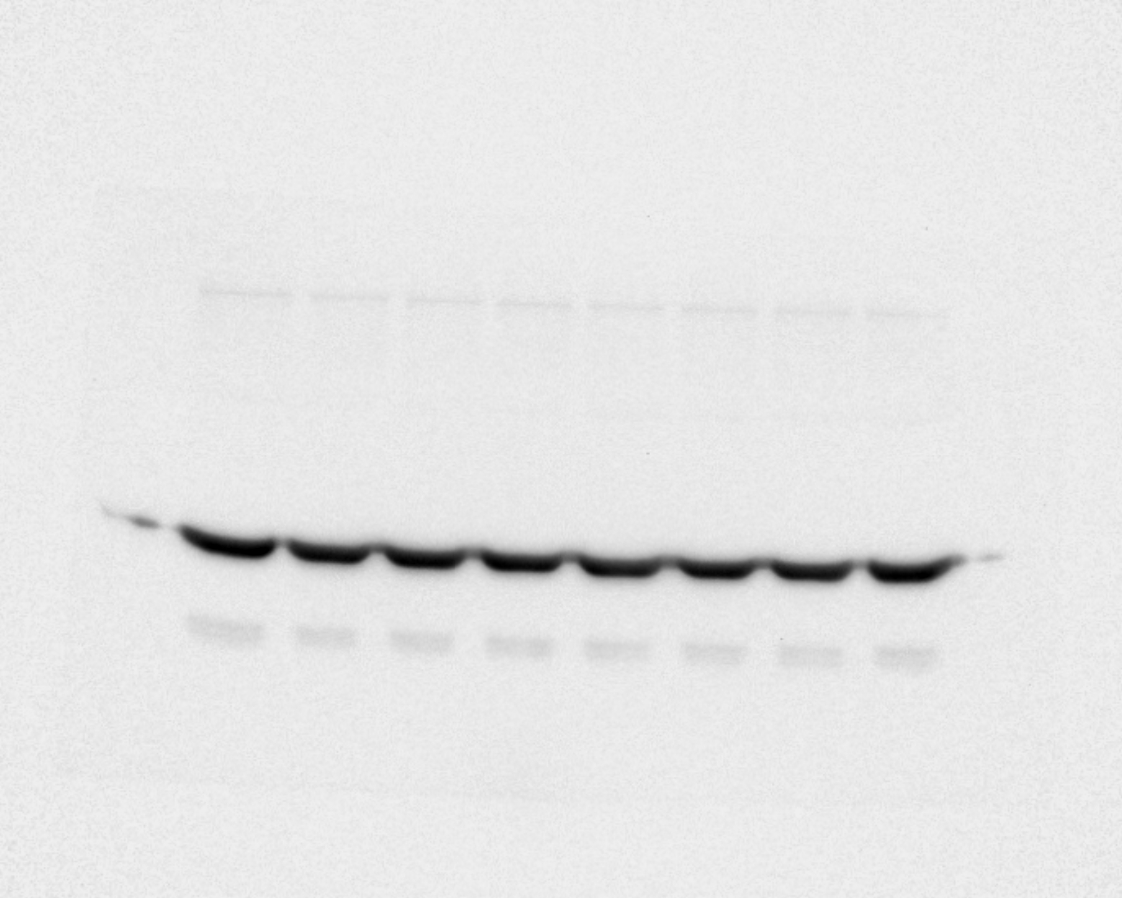

Supplement: Figure 5—figure supplement 1—source data 2. [file elife-89951-fig5-figsupp1-data2.zip › Figure 5-figure supplement 1-source data 2/ACTIN_Figure 5-figure supplement 1-source data 2/Versteeg 2023-03-24 15h18m18s 37.549s(Chemiluminescence).jpg]

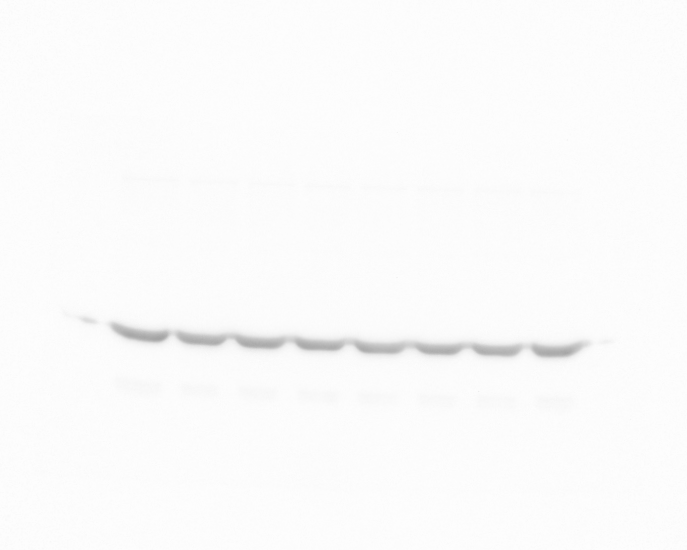

Supplement: Figure 5—figure supplement 1—source data 2. [file elife-89951-fig5-figsupp1-data2.zip › Figure 5-figure supplement 1-source data 2/ACTIN_Figure 5-figure supplement 1-source data 2/Versteeg 2023-03-24 15h18m18s 37.549s(Chemiluminescence).raw16.tif]

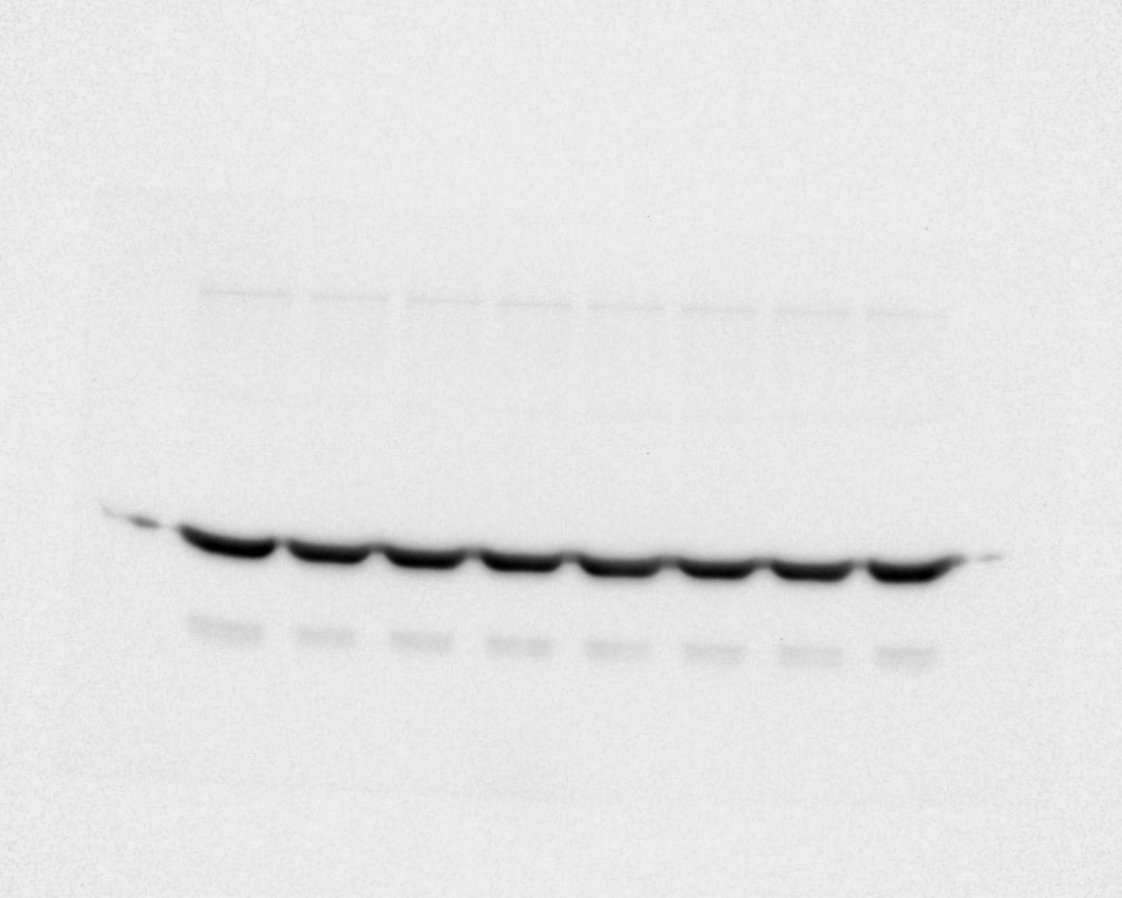

Supplement: Figure 5—figure supplement 1—source data 2. [file elife-89951-fig5-figsupp1-data2.zip › Figure 5-figure supplement 1-source data 2/ACTIN_Figure 5-figure supplement 1-source data 2/Versteeg 2023-03-24 15h18m18s 37.549s(Chemiluminescence).tif]

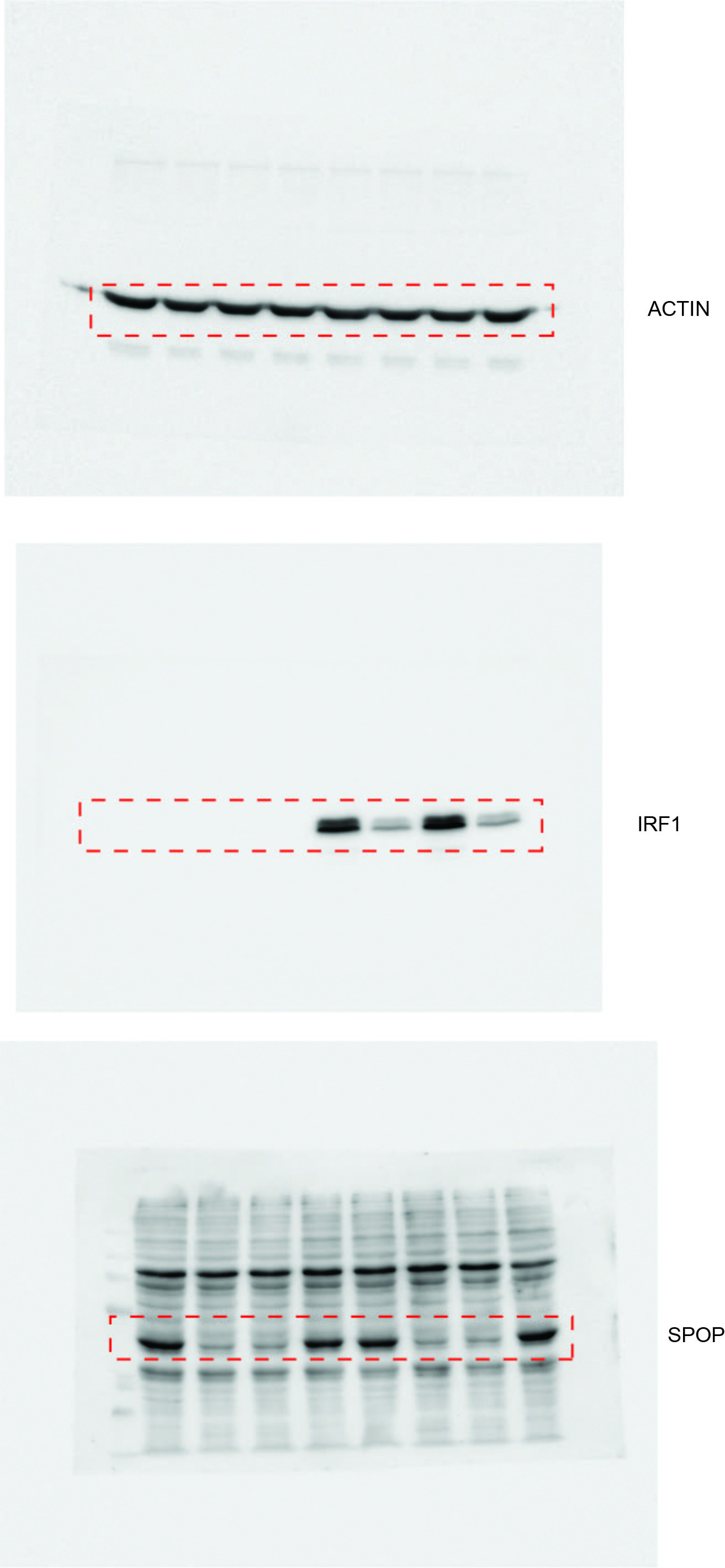

Supplement: Figure 5—figure supplement 1—source data 2. [file elife-89951-fig5-figsupp1-data2.zip › Figure 5-figure supplement 1-source data 2/Figure 5-figure supplement 1-source data 2.jpg]

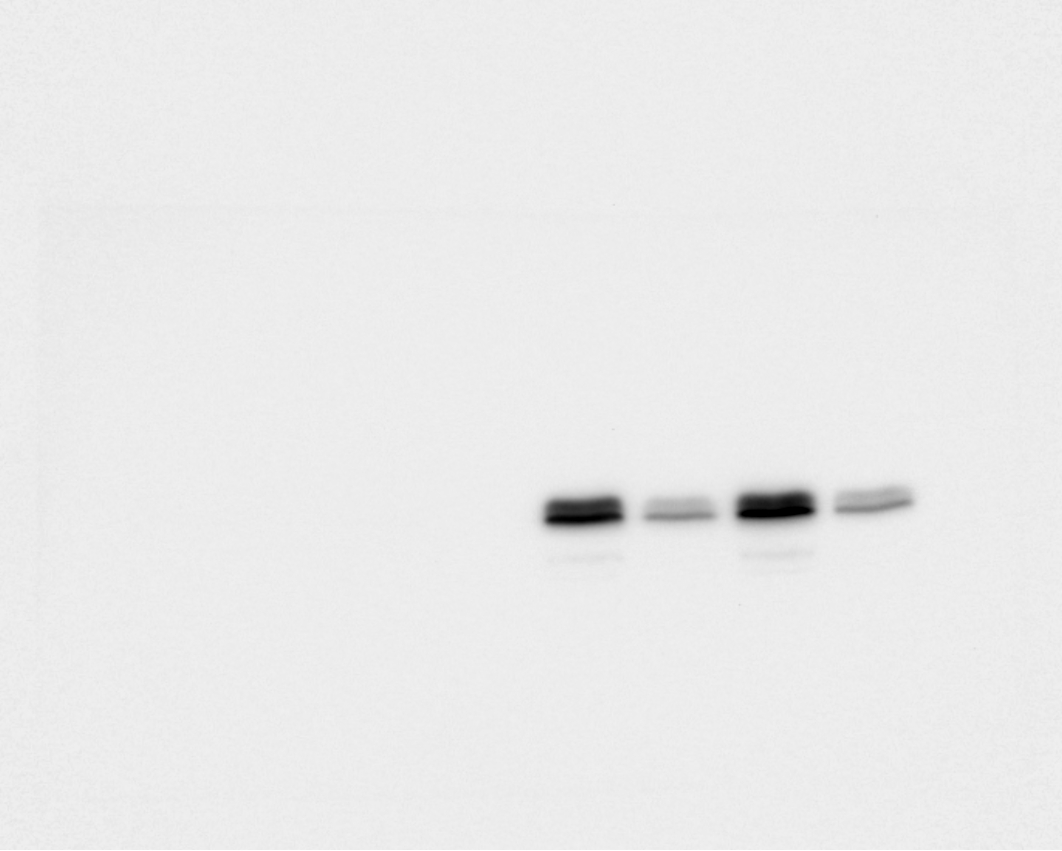

Supplement: Figure 5—figure supplement 1—source data 2. [file elife-89951-fig5-figsupp1-data2.zip › Figure 5-figure supplement 1-source data 2/IRF1_Figure 5-figure supplement 1-source data 2/Versteeg 2023-03-23 09h04m51s 19.306s(Chemiluminescence).jpg]

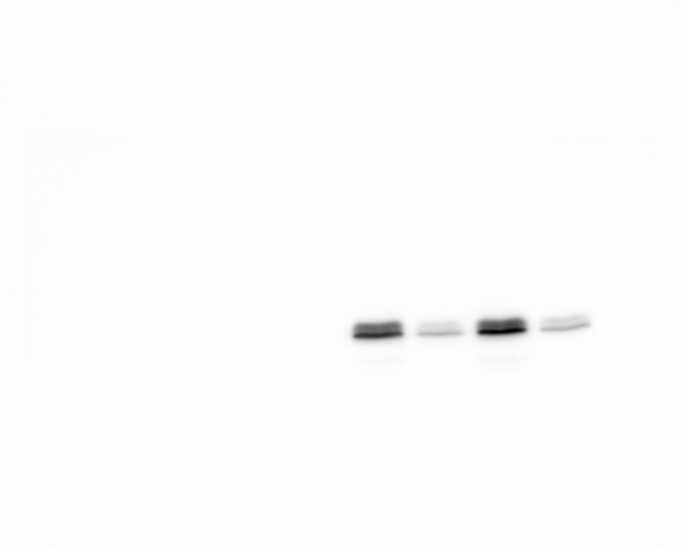

Supplement: Figure 5—figure supplement 1—source data 2. [file elife-89951-fig5-figsupp1-data2.zip › Figure 5-figure supplement 1-source data 2/IRF1_Figure 5-figure supplement 1-source data 2/Versteeg 2023-03-23 09h04m51s 19.306s(Chemiluminescence).raw16.tif]

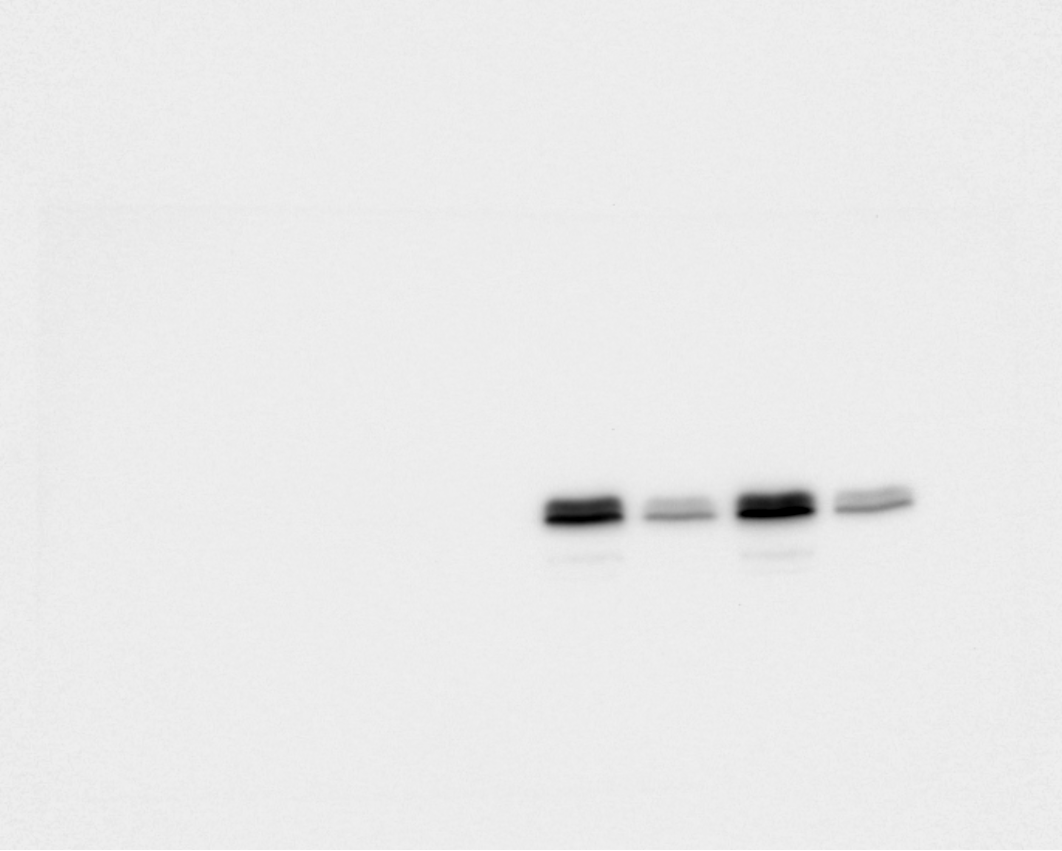

Supplement: Figure 5—figure supplement 1—source data 2. [file elife-89951-fig5-figsupp1-data2.zip › Figure 5-figure supplement 1-source data 2/IRF1_Figure 5-figure supplement 1-source data 2/Versteeg 2023-03-23 09h04m51s 19.306s(Chemiluminescence).tif]

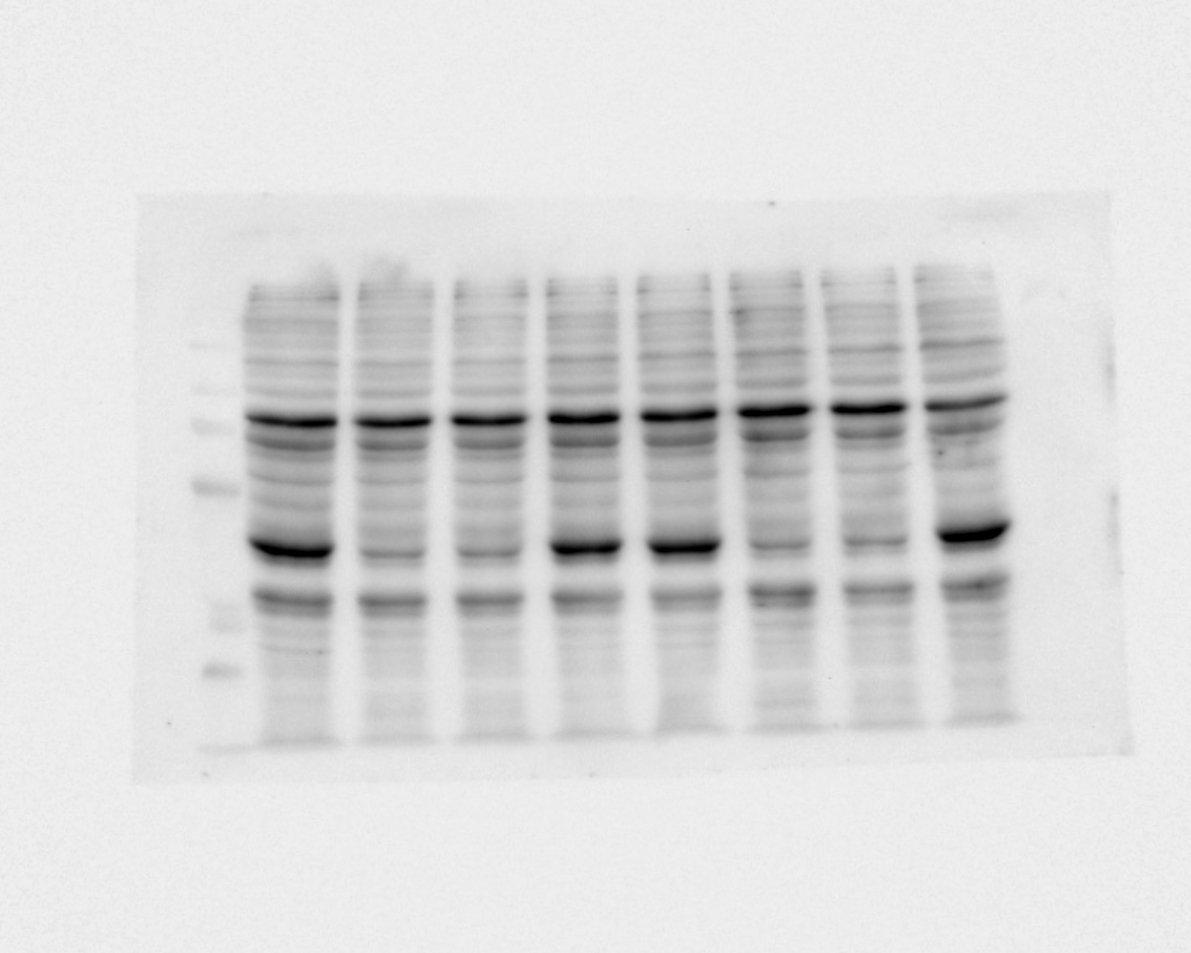

Supplement: Figure 5—figure supplement 1—source data 2. [file elife-89951-fig5-figsupp1-data2.zip › Figure 5-figure supplement 1-source data 2/SPOP_Figure 5-figure supplement 1-source data 2/Versteeg 2023-03-24 13h11m23s 25.408s(Chemiluminescence).jpg]

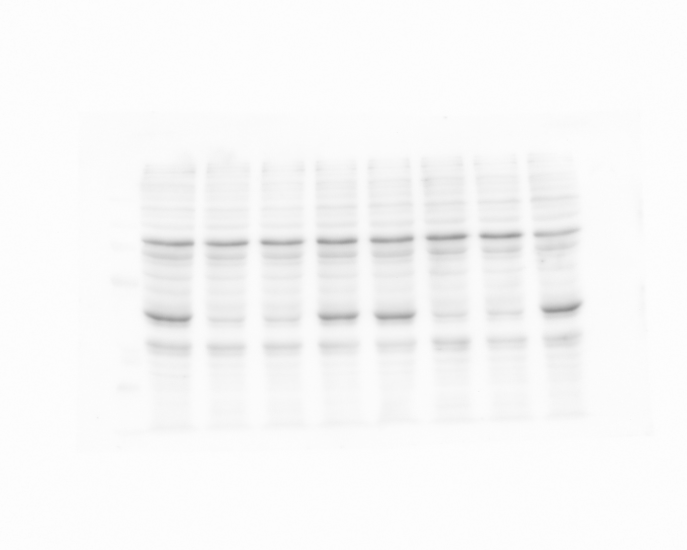

Supplement: Figure 5—figure supplement 1—source data 2. [file elife-89951-fig5-figsupp1-data2.zip › Figure 5-figure supplement 1-source data 2/SPOP_Figure 5-figure supplement 1-source data 2/Versteeg 2023-03-24 13h11m23s 25.408s(Chemiluminescence).raw16.tif]

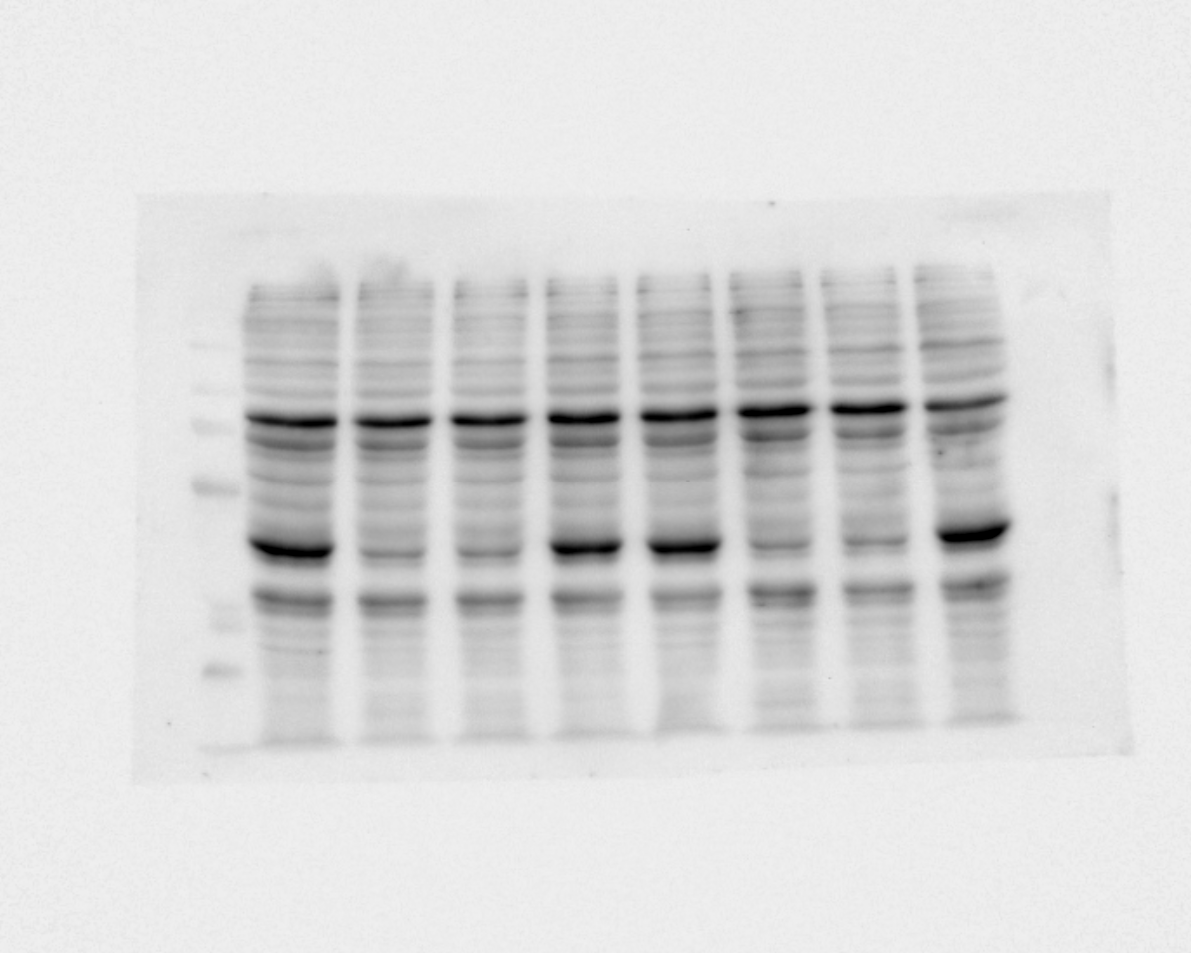

Supplement: Figure 5—figure supplement 1—source data 2. [file elife-89951-fig5-figsupp1-data2.zip › Figure 5-figure supplement 1-source data 2/SPOP_Figure 5-figure supplement 1-source data 2/Versteeg 2023-03-24 13h11m23s 25.408s(Chemiluminescence).tif]
